# Design, Synthesis, In Silico Studies and Inhibitory Activity towards Bcr-Abl, BTK and FLT3-ITD of New 2,6,9-Trisubstituted Purine Derivatives as Potential Agents for the Treatment of Leukaemia

**DOI:** 10.3390/pharmaceutics14061294

**Published:** 2022-06-17

**Authors:** Jeanluc Bertrand, Hana Dostálová, Vladimír Kryštof, Radek Jorda, Thalía Delgado, Alejandro Castro-Alvarez, Jaime Mella, David Cabezas, Mario Faúndez, Christian Espinosa-Bustos, Cristian O. Salas

**Affiliations:** 1 Departamento de Química Orgánica, Facultad de Química y de Farmacia, Pontificia Universidad Católica de Chile, Avenida Vicuña Mackenna 4860, Santiago 7820436, Chile; jgbertrand@uc.cl (J.B.); tdelgado@uc.cl (T.D.); 2 Department of Experimental Biology, Palacký University Olomouc, Šlechtitelů 27, 783 71 Olomouc, Czech Republic; hana.dostalova@upol.cz (H.D.); radek.jorda@upol.cz (R.J.); 3 Institute of Molecular and Translational Medicine, Faculty of Medicine and Dentistry, Palacký University Olomouc, Hněvotínská 5, 779 00 Olomouc, Czech Republic; 4 Departamento de Ciencias Preclínicas, Facultad de Medicina, Universidad de La Frontera, Manuel Montt 112, Temuco 4780000, Chile; qf.alec.astro@gmail.com; 5 Instituto de Química y Bioquímica, Facultad de Ciencias, Universidad de Valparaíso, Avenida Gran Bretaña 1111, Valparaíso 2360102, Chile; jaime.mella@uv.cl (J.M.); david.cg172012@gmail.com (D.C.); 6 Facultad de Farmacia, Centro de Investigación Farmacopea Chilena, Universidad de Valparaíso, Avenida Gran Bretaña 1093, Valparaíso 2360102, Chile; 7 Departamento de Farmacia, Facultad de Química y de Farmacia, Pontificia Universidad Católica de Chile, Avenida Vicuña Mackenna 4860, Santiago 7820436, Chile; mfaundeza@uc.cl (M.F.); ccespino@uc.cl (C.E.-B.)

**Keywords:** leukaemia, purine derivatives, tyrosine kinases inhibitors, 3D-QSAR, molecular docking

## Abstract

We report 31 new compounds designed, synthesized and evaluated on Bcr-Abl, BTK and FLT3-ITD as part of our program to develop 2,6,9-trisubstituted purine derivatives as inhibitors of oncogenic kinases. The design was inspired by the chemical structures of well-known kinase inhibitors and our previously developed purine derivatives. The synthesis of these purines was simple and used a microwave reactor for the final step. Kinase assays showed three inhibitors with high selectivity for each protein that were identified: **4f** (IC_50_ = 70 nM for Bcr-Abl), **5j** (IC_50_ = 0.41 μM for BTK) and **5b** (IC_50_ = 0.38 μM for FLT-ITD). The 3D-QSAR analysis and molecular docking studies suggested that two fragments are potent and selective inhibitors of these three kinases: a substitution at the 6-phenylamino ring and the length and volume of the alkyl group at N-9. The N-7 and the *N*-methyl-piperazine moiety linked to the aminophenyl ring at C-2 are also requirements for obtaining the activity. Furthermore, most of these purine derivatives were shown to have a significant inhibitory effect in vitro on the proliferation of leukaemia and lymphoma cells (HL60, MV4-11, CEM, K562 and Ramos) at low concentrations. Finally, we show that the selected purines (**4i**, **5b** and **5j**) inhibit the downstream signalling of the respective kinases in cell models. Thus, this study provides new evidence regarding how certain chemical modifications of purine ring substituents provide novel inhibitors of target kinases as potential anti-leukaemia drugs.

## 1. Introduction

Leukaemia, lymphoma and myeloma are blood malignancies of the lymph haematological system; they originate in the bone marrow or lymph system through haematopoiesis [1]. Hematologic malignancies are principally characterized by the cell progenitor from which the dangerous cell type is determined: myeloid leukaemia, lymphoid leukaemia, lymphoma and multiple myeloma. Leukaemia diseases include different classifications where “acute” and “chronic” refer to the grade of maturation of the cancer blood cells. Here, myeloid leukaemia is categorised by acute myelogenous leukaemia (AML) and chronic myeloid leukaemia (CML). Similarly, lymphoid leukaemia is classified as acute lymphoblastic leukaemia (ALL) and chronic lymphocytic leukaemia (CLL) [2,3].

**Figure 1:**
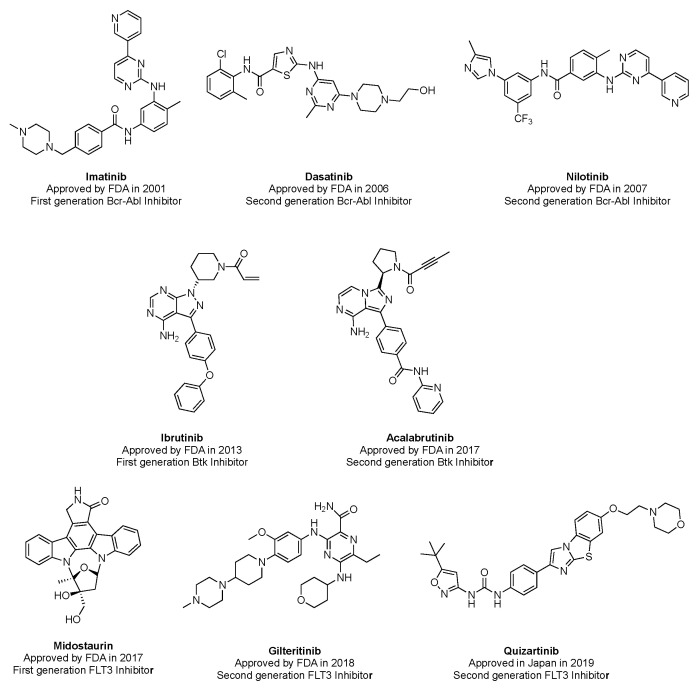
Chemical structures of some TKIs used in the treatment of leukaemia.

Currently, various treatments are available to treat these haematological diseases [4]. Some drugs can treat more than one type of leukaemia, particularly tyrosine kinase inhibitors (TKIs, Figure 1) [5,6,7]. These drugs have been very effective when a mutation and other hereditary deviations cause hyperactivation of several signalling pathways. They generally promote survival and cell proliferation. In CML, a broad majority of cases and 20–30% of ALL are due to chromosomal translocation between chromosomes 9 and 22 [t(9, 22)], thus, forming the Philadelphia chromosome (Ph) that encodes the fusion protein break-point cluster region–Abelson (Bcr-Abl) with tyrosine kinase activity deregulated; this, in turn, drives survival and proliferation through multiple downstream signalling pathways [8,9]. 

Despite the increase in overall survival promoted by imatinib (first generation of Bcr-Abl inhibitor), dasatinib and nilotinib (second generation of Bcr-Abl inhibitors), more than 25% of patients must modify the treatment due to resistance [10,11,12,13]. The most common resistances are based on an amino acid substitution in the kinase domain where T315I (Ile replaces Thr at position 315 of Bcr-Abl) appear in 2% to 20% of patients with CML; they, in turn, develop resistance to imatinib, dasatinib and nilotinib [14,15].

Another tyrosine kinase that has major implications in haematological malignancies is Bruton tyrosine kinase (BTK) [16,17]. Patients with loss-of-function mutations in the BTK gene develop XLA, which is an immune deficiency of B-cells in the blood and lymphoid system characterized by a lack of maturation and development of B lymphocytes [18,19,20]. However, chronic activation of the BTK signalling activated by antigen binding to a B-cell receptor (BCR) can promote haematological malignancies [16,17]. 

BCR stimulation triggers BTK phosphorylation, thus, promoting the activation of several downstream signalling pathways, including MAPK, PI3K/Akt/mTOR and NF-κB. Hence, BTK mediates B-cell proliferation and survival, and thus targeting this pathway is a novel target in B-cell malignancies [21]. Ibrutinib and acalabrutinib (Figure 1) are small molecules that inhibit covalently BTK by targeting Cys481; they have been approved for the treatment of CLL [22,23]. Nevertheless, Cys481 mutations to Ser or Thr, lacking the Michael donor group thiol, and causing covalent drug resistance, have been identified [23,24,25].

AML is the most common leukaemia type (32% of all types of leukaemia patients) [26]; 30% of AML patients elicited a mutation in Fms-like tyrosine kinase 3 receptor (FLT3) [27]. The *FLT3* gene transcript is a transmembrane tyrosine kinase receptor FLT3, which binds with an extracellular ligand, dimerises and then is autophosphorylated to activate several growth, proliferative, differentiation and survival downstream signalling pathways [28]. 

The most common mutation in FLT3 is the internal tandem duplication (ITD), which promotes ligand-independent dimerization of the kinase, thus, causing it to adopt a stable and active conformation with increased tyrosine kinase activity [29]. Midostaurine and gilteritinib are FLT3-ITD inhibitors approved by the Food and Drugs Administration (FDA) in 2017 and 2018, respectively; quizartinib was approved in 2019 in Japan, Figure 1. However, toxicity problems due to their non-specificity toward tyrosine kinases and the appearance of resistance mutations over its administration present a challenge to overcome [30,31,32,33].

**Figure 2:**
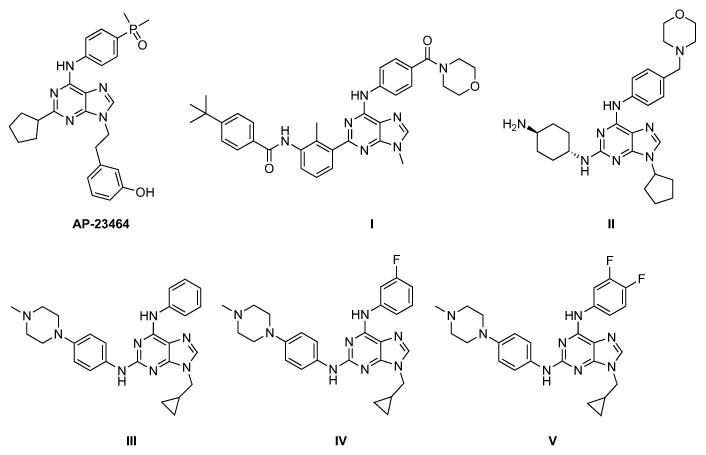
Some examples of purine derivatives reported as TKIs inhibitors.

To overcome the limitations of current clinical TKIs, several Bcr-Abl, BTK and FLT3-ITD inhibitors have been developed; most are nitrogenous heterocyclic compounds [7,34,35]. Of the nitrogen-heterocycles used for TKIs, the purine fragment is of particular interest [36,37] because the purine ring is considered a “privilege scaffold” in medicinal chemistry [38,39]. Azam et al. [40] developed **AP23464** (Figure 2) as a potent Bcr-Abl inhibitor (IC_50_ < 1 nM). Likewise, Shi et al. [41] synthesized and evaluated some 2,6,9-trisubstituted purine derivatives as reversible inhibitors of BTK. 

Compound **I** (Figure 2) showed the most promising activity (IC_50_ = 8 nM). Gucký et al. [42] optimized purine derivatives in the aniline fragment to convert potent CKD-2 inhibitors into new FLT3-ITD inhibitors. Here, **II** (Figure 2) showed an interesting inhibitory activity on FLT3-ITD with an IC_50_ value of 2 nM. For **AP23464**, [43] **I** [41] and **II** [42] were found to have important interactions between the N-7 and the NH aniline substitution on the C-6 of the purine ring to stabilize the inhibitor-kinase complexes in the ATP binding site.

Our goal is to develop new Bcr-Abl and BTK inhibitors based on derivatives of 2,6,9-trisubstituted purine derivatives. We obtained new compounds that inhibit both kinases [44]. Of the 13 new compounds, **III**, **IV** and **V** depicted the best results as Bcr-Abl and BTK inhibitors (Figure 2). These results allowed us to identify compound **V**, which was an effective inhibitor of Bcr-Abl and BTK (IC_50_ values of 40 nM and 0.58 μM, respectively) [44]. Furthermore, the substitution of a fluorine atom in the aniline fragment (on C-6) at *meta*- or *para*-positions was shown to be important for the inhibition of these kinases. 

From the docking studies of **V**, the main interactions in both bonding pockets indicated that hydrogen bonds between N-7/NH (purine moiety) with NH/CO (M318 for Bcr-Abl and M477 for BTK) and the salt bridge or hydrogen bond between NH^+^-piperazine with the respective residue (D325 for Bcr-Abl and N526 for BTK) stabilized the **V**-kinase complexes. Docking studies suggested that the hydrophobic pocket in both kinases is an interesting point to explore. In addition, QSAR analysis showed that the *meta*-position in the aniline fragment could be modified more freely for new inhibitors of Bcr-Abl and BTK. **V** was highly cytotoxic on leukaemia cancer cell lines (K562, CEM, Ramos and MV4-11 cells) with IC_50_ values between 1.24 and 7.62 μM. We also demonstrated that **V** decreased the phosphorylation of some kinases downstream of both targets in K562 and Ramos cells.

**Figure 3:**
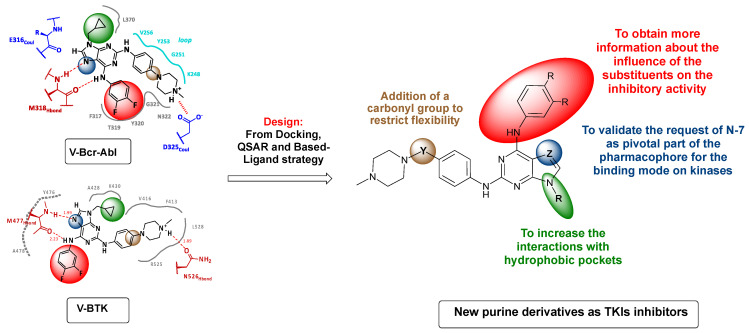
Design of new TKIs based on 2,6,9-trisubstituted purines considering chemical structures of compounds **AP-23464** and **I**–**V** and docking results of **V** with Bcr-Abl/BTK.

In this work, continuing our program to develop 2,6,9-trisubstituted purine derivatives with antitumoral activity [44,45,46,47], we report a new set of 31 purine derivatives with interesting effects on Bcr-Abl, BTK and FLT3-ITD activities. In the design of our compounds, we considered our previous results and the chemical structures of the purine-based kinase inhibitors (Figure 3). First, the aminophenyl fragment on C-6 was mainly substituted with some electron-withdrawing groups (EWG) in *ortho*, *meta-* and/or *para*-positions. 

This modification allowed us to obtain more information about the influence of the substituents on inhibitory activity. Second, modifications in the volume and length of the alkyl group on N-9 were considered to increase the hydrophobic interactions in the binding cavity of these kinases. To validate the key role of the N-7/NH moiety to stabilize the **V**-kinase complexes, an analogue of **V** was proposed in which the N-7 was replaced by CH. To conclude this design, the *N*-methyl-arylpiperazine fragment on C-2 was conserved in these new purine derivatives due to the important interaction that **V** had with both kinase cavities. 

Other kinase inhibitors exhibit antitumoral properties (as well as the Cdk inhibitor milciclib [48] and the Axl inhibitor SGI-7079 [49]) and contain this moiety. However, a carbonyl group was added between both rings to understand the importance of flexibility in the *N*-methyl-arylpiperazine fragment. Finally, the cytotoxic effect of these new purine derivatives on some cancer cell lines related to haematological malignancies was assayed; we also studied the inhibition of these kinases in cell lines and performed rigorous in silico studies, including 3D-QSAR and docking studies, to understand the structure–activity relationship of these ligands on these kinases and the selectivity between them.

## 2. Materials and Methods

### 2.1. Chemistry

The reagents and chemicals used in this work were obtained from Sigma Aldrich (St. Louis, MO, USA). Those compounds that had already been published previously were synthesised according to the respective procedures and this was indicated with the relevant references. The purity of all synthesised compounds was checked by NMR, TLC and HRMS. In the case of TLC, silica gel 60 F_254_ 25 aluminium foils 20 × 20 C (purchased from Merck, Burlington, CA, USA) were used as the stationary phase, and the mobile phase is specified in each reaction procedure. In the NMR spectra, the chemical shifts of each signal are reported in parts per million (ppm) and the coupling constants (*J*) in hertz (*Hz*). For the multiplicity of the NMR signals, they are expressed as s (singlet), d (doublet), t (triplet) and dd (doublet doublet), as appropriate.

The following instruments were used for the identification and characterisation of each of the synthesised compounds: (1) Kofler Thermogerate apparatus (Reichert, Werke A.G., Wien, Vienna, Austria) for the determination of the melting points (mp), which are expressed in degrees Celsius (°C) without corrections; (2) BRUKER AVANCE III HD-400 spectrometers [400 MHz (^1^H) and 100 MHz (^13^C)] or 200 MHz [200 MHz (^1^H) and 50 MHz (^13^C)] to obtain the ^1^H and ^13^C NMR spectra (tetramethylsilane, TMS, was used as internal reference); and (3) BRUKER COMPACT QTOF MS + Elute UHPLC with a constant nebuliser temperature of 250 °C, for the acquisition of HRMS-ESI data. This methodology was used for the final products. For this, samples dissolved in acetonitrile were injected directly into the ESI source through an injection valve and by means of a syringe pump with a flow rate of 5 µL min^−1^. 

Measurements were performed in positive ion mode, with a scanning range of *m*/*z* 300.00–1510.40 and a resolution of 140,000; (4) LC-MS experiments were conducted on an UHPLC Eksigent1 coupled with MS detector ABSciex1, Triple Quad 4500 model equipment. This methodology was used for reaction intermediates. The samples were directly injected by using a syringe, and the data was collected in a range of 100.0–600.0 Da, at 200 Da s^−1^ and positive polarity.

#### 2.1.1. General Procedures for the Synthesis of Compounds **2a**–**2e** and **2a’**–**2e’**

In a reaction flask, 2,6-dichloro-9*H*-purine (**1**, 2.0 g, 1.10 mol), the respective alkyl halide (1.58 mol) and K_2_CO_3_ (3.36 g, 3.17 mol) were suspended in DMF (20 mL). The mixture was stirred for 12 h, at room temperature. Then, the suspension was filtered, and the solvent was removed under vacuum. The crude product was purified by column chromatography on silica gel, using acetone/dichloromethane (5:95) as the mobile phase to yield the products **2a**–**2e** and **2a’**–**2e’**. R_f_ values of each compound of this series was calculated using the mentioned mobile phase.

2,6-Dichloro-9-(cyclopropylmethyl)-9*H*-purine, (**2a**): White solid, yield 51%. The analytical data corresponded to literature [44].

2,6-Dichloro-7-(cyclopropylmethyl)-7*H*-purine, (**2a’**): White solid, yield 18%. The analytical data corresponded to literature [44].

2,6-Dichloro-9-isopentyl-9*H*-purine, (**2b**): White solid, yield 49%. The analytical data corresponded to literature [46].

2,6-Dichloro-7-isopentyl-7*H*-purine, (**2b’**): White oil, yield 15%. The analytical data corresponded to literature [46].

2,6-Dichloro-9-pentyl-9*H*-purine, (**2c**): White solid, yield 50%, The analytical data corresponded to literature [46].

2,6-Dichloro-7-pentyl-7*H*-purine, (**2c’**): Yellow oil, yield 15%. The analytical data corresponded to literature [46].

2,6-Dichloro-9-hexyl-9*H*-purine, (**2d**): White solid, yield 45%. The analytical data corresponded to literature [46].

2,6-Dichloro-7-hexyl-7*H*-purine, (**2d’**): Yellow oil, yield 23%. The analytical data corresponded to literature [46].

2,6-Dichloro-9-octyl-9*H*-purine, (**2e**): White solid, yield 93%, mp 43.5–47.3 °C, R_f_ = 0.6. ^1^H NMR (400 MHz, CDCl_3_) δ 8.09 (s, 1H), 4.25 (t, *J* = 7.3 Hz, 2H), 1.99–1.78 (m, 2H), 1.41–1.08 (m, 11H), 0.86 (dd, *J* = 8.1, 5.6 Hz, 3H). ^13^C NMR (101 MHz, CDCl_3_) δ 153.32, 153.02, 151.84, 145.85, 130.88, 77.16, 44.81, 31.78, 29.89, 29.14, 29.01, 26.68, 22.68, 14.15. ESI/MS for (C_13_H_18_Cl_2_N_4_ [M + H]^+^). Calcd: 301.1. Found: 300.8.

2,6-Dichloro-7-octyl-7*H*-purine, (**2e´**): White solid, yield 5%, mp 64.2–65.9 °C, R_f_ = 0.5. ^1^H NMR (400 MHz, CDCl_3_) δ 8.23 (s, 1H, CH), 4.45 (t, *J* = 11.9 Hz, 2H, CH_2_), 1.99 (d, *J* = 6.3 Hz, 2H, CH_2_), 1.28 (m, 10H, 5CH_2_), 0.93–0.77 (m, 3H, CH_3_). ^13^C NMR (101 MHz, CDCl_3_) δ 161.08, 154.91, 147.68, 144.60, 126.64, 44.81, 31.78, 29.89, 29.14, 29.01, 26.68, 22.68, 14.15. ESI/MS for (C_13_H_18_Cl_2_N_4_ [M + H]^+^). Calcd: 301.1. Found: 300.8.

#### 2.1.2. General Procedures for the Synthesis of Compounds **3a**–**3ad**

In a reaction flask, **2a**–**2e** (0.411 mmol), the respective aniline (0.411 mmol) DIPEA (0.15 mL, 0.822 mmol) were dissolved in *n*-butanol (20 mL). The mixture was stirred for 12 h, at 110 °C. Then, the suspension was cooled at room temperature, and the solvent was removed under vacuum. The crude product was purified by column chromatography on silica gel, using acetone/dichloromethane (10:90) as the mobile phase to yield products **3a**–**3ad**. The Rf values of each compound of this series were calculated using the mentioned mobile phase.

2-Chloro-9-(cyclopropylmethyl)-*N*-(2-fluorophenyl)-9*H*-purin-6-amine, (**3a**): White solid, yield 61%, mp 135–138 °C, R_f_ = 0.3. ^1^H NMR (400 MHz, CDCl_3_) *δ* 8.58 (dd, *J* = 8.1, 7.2 Hz, 1H, CH), 7.97 (s, 2H, NH, CH), 7.21 (t, *J* = 7.7 Hz, 1H, CH), 7.18–7.11 (m, 1H, CH), 7.11–7.04 (m, 1H, CH), 4.05 (d, *J* = 7.3 Hz, 2H, CH_2_), 1.42–1.26 (m, 1H, CH), 0.69 (q, *J* = 5.6 Hz, 2H, CH_2_), 0.46 (q, *J* = 5.1 Hz, 2H, CH_2_). ^13^C NMR (101 MHz, CDCl_3_) *δ* 154.03, 153.26 (d, ^1^*J_C-F_* = 244.5 Hz, CF), 152.16, 152.04, 151.10, 140.89, 126.62 (d, ^2^*J_C-F_* = 9.9 Hz), 124.70 (d, ^4^*J_C-F_* = 3.8 Hz, CH), 124.25 (d, ^3^*J_C-F_* = 7.6 Hz, CH), 115.14 (d, ^2^*J_C-F_* = 19.1 Hz, CH), 48.88, 11.18, 4.51 (2C). ^19^F RMN (376 MHz, CDCl_3_) δ −130.50 (s, 1F). ESI/MS for (C_15_H_13_ClFN_5_ [M + H]^+^): Calcd: 318.1. Found: 318.1.

2-Chloro-*N*-(3-chlorophenyl)-9-(cyclopropylmethyl)-9*H*-purin-6-amine, (**3b**): Brown solid, yield 73%, mp 140–141 °C, R_f_ = 0.3. ^1^H NMR (400 MHz, CDCl_3_) *δ* 8.20 (s, 1H, NH), 7.91 (s, 1H. CH), 7.85 (t, *J* = 2.0 Hz, 1H. CH), 7.66 (dd, *J* = 8.2, 1.4 Hz, 1H. CH), 7.28 (t, *J* = 8.1 Hz, 1H. CH), 7.08 (dd, *J* = 8.0, 1.1 Hz, 1H. CH), 4.03 (t, *J* = 6.3 Hz, 2H. CH_2_), 1.36–1.25 (m, 1H. CH), 0.69 (q, *J* = 5.8 Hz, 2H. CH_2_), 0.45 (q, *J* = 5.0 Hz, 2H. CH_2_). ^13^C NMR (101 MHz, CDCl_3_) *δ* 153.92, 152.03, 150.93, 140.80, 139.39, 134.63, 130.06, 123.85, 120.15, 119.20, 118.21, 48.74, 11.03, 4.39 (2C). ESI/MS for (C_15_H_13_Cl_2_N_5_ [M + H]^+^): Calcd: 334.1. Found: 333.7.

2-Chloro-9-(cyclopropylmethyl)-*N*-(*m*-tolyl)-9*H*-purin-6-amine, (**3c**): White solid, yield 80%, mp 115–117 °C, R_f_ = 0.3. ^1^H NMR (400 MHz, CDCl_3_) *δ* 7.99 (s, 1H, NH), 7.81 (s, 1H, CH), 7.55 (d, *J* = 8.0 Hz, 1H, CH), 7.43 (s, 1H, CH), 7.19 (t, *J* = 7.8 Hz, 1H, CH), 6.87 (d, *J* = 7.5 Hz, 1H, CH), 3.94 (d, *J* = 7.3 Hz, 2H, CH_2_), 2.30 (s, 3H, CH_3_), 1.23 (dtd, *J* = 15.2, 7.5, 2.7 Hz, 1H, CH), 0.60 (q, *J* = 5.6 Hz, 2H, CH_2_), 0.36 (q, *J* = 5.2 Hz, 2H, CH_2_). ^13^C NMR (101 MHz, CDCl_3_) *δ* 154.18, 152.46, 150.77, 140.52, 139.02, 138.08, 129.03, 124.99, 121.06, 119.16, 117.66, 48.74, 21.69, 11.13, 4.45 (2C). ESI/MS for (C_16_H_16_ClN_5_ [M + H]^+^): Calcd: 314.1. Found: 314.0.

2-Chloro-9-(cyclopropylmethyl)-*N*-(3-(trifluoromethyl)phenyl)-9*H*-purin-6-amine, (**3d**): White solid, yield 45%, mp 125–127 °C, R_f_ = 0.3. ^1^H NMR (400 MHz, CDCl_3_) *δ* 8.40 (s, 1H, NH), 8.06 (s, 1H, CH), 8.05 (d, *J* = 7.6 Hz, 1H, CH), 7.94 (s, 1H, CH), 7.50 (t, *J* = 7.9 Hz, 1H, CH), 7.37 (d, *J* = 7.8 Hz, 1H, CH), 4.04 (d, *J* = 7.3 Hz, 2H, CH_2_), 1.38–1.27 (m, 1H, CH), 0.70 (q, *J* = 5.9 Hz, 2H, CH_2_), 0.46 (q, *J* = 5.0 Hz, 2H, CH_2_). ^13^C NMR (101 MHz, CDCl_3_) *δ* 153.99, 152.02, 151.00, 140.79, 138.80, 131.43 (q, ^2^*J_C-F_* = 32.4 Hz, C-CF_3_), 129.64, 123.91 (d, ^1^*J_C-F_* = 272.4 Hz, CF_3_), 123.19, 120.31 (q, ^3^*J_C-F_* = 3.7 Hz, CH), 119.07, 116.84 (q, ^3^*J_C-F_* = 4.0 Hz, CH), 48.79, 11.00, 4.38 (2C). ^19^F NMR (376 MHz, CDCl_3_) *δ* −62.79 (s, 3F). ESI/MS for (C_16_H_13_ClF_3_N_5_ [M + H]^+^): Calcd: 368.1. Found: 367.8.

2-Chloro-9-(cyclopropylmethyl)-*N*-(3-(trifluoromethoxy)phenyl)-9*H*-purin-6-amine, (**3e**): Brown solid, yield 68%, mp 137–138 °C, R_f_ = 0.3. ^1^H NMR (400 MHz, CDCl_3_) *δ* 8.41 (s, 1H, NH), 7.94 (s, 1H, CH), 7.85 (s, 1H, CH), 7.66 (dd, *J* = 8.2, 1.6 Hz, 1H, CH), 7.37 (t, *J* = 8.2 Hz, 1H, CH), 6.97 (d, *J* = 8.2 Hz, 1H, CH), 4.04 (d, *J* = 7.3 Hz, 2H, CH_2_), 1.40–1.25 (m, 1H, CH), 0.69 (q, *J* = 5.7 Hz, 2H, CH_2_), 0.45 (q, *J* = 5.1 Hz, 2H, CH_2_). ^13^C NMR (101 MHz, CDCl_3_) *δ* 154.01, 151.95, 150.95, 149.64 (d, ^3^*J_C-F_* = 1.8 Hz, C-OCF_3_), 140.75, 139.65, 121.83, 121.28, 120.50 (q, ^1^*J_C-F_* = 257.5 Hz, CF_3_), 119.22, 119.00, 118.12, 115.77, 112.78, 48.80, 10.99, 4.30 (2C). ^19^F NMR (376 MHz, CDCl_3_) *δ* −57.68 (s, 3F). ESI/MS for (C_16_H_13_ClF_3_N_5_O [M + H]^+^): Calcd: 384.1. Found: 383.9.

3-((2-Chloro-9-(cyclopropylmethyl)-9*H*-purin-6-yl)amino)benzonitrile, (**3f**): White solid, yield 74%, mp 145–146 °C, R_f_ = 0.2. ^1^H NMR (400 MHz, CDCl_3_) *δ* 8.62 (s, 1H, NH), 8.22–8.16 (m, 1H, CH), 8.00 (dd, *J* = 8.4, 1.3 Hz, 1H, CH), 7.98 (s, 1H, CH), 7.45 (t, *J* = 8.0 Hz, 1H, CH), 7.36 (d, *J* = 7.7 Hz, 1H, CH), 4.05 (d, *J* = 7.3 Hz, 2H, CH_2_), 1.46–1.16 (m, 1H, CH), 0.70 (q, *J* = 5.8 Hz, 2H, CH_2_), 0.46 (q, *J* = 5.0 Hz, 2H, CH_2_). ^13^C NMR (101 MHz, CDCl_3_) *δ* 154.04, 151.85, 151.13, 141.02, 139.34, 130.01, 127.15, 124.23, 123.09, 119.01, 118.76, 113.09, 48.98, 11.09, 4.52 (2C). ESI/MS for (C_16_H_13_ClN_6_ [M + H]^+^): Calcd: 325.1. Found: 324.9.

2-Chloro-9-(cyclopropylmethyl)-*N*-(3-nitrophenyl)-9*H*-purin-6-amine, (**3g**): Yellow solid, yield 66%, mp 150–151 °C, R_f_ = 0.2. ^1^H NMR (400 MHz, CDCl_3_) *δ* 8.66 (s, 1H, CH), 8.39 (s, 1H, NH), 8.20 (dd, *J* = 8.2, 1.4 Hz, 1H, CH), 7.98 (s, 1H, CH), 7.95 (dd, *J* = 8.2, 1.3 Hz, 1H, CH), 7.54 (t, *J* = 8.2 Hz, 1H, CH), 4.06 (d, *J* = 7.3 Hz, 2H, CH_2_), 1.43–1.28 (m, 1H, CH), 0.70 (q, *J* = 5.7 Hz, 2H, CH_2_), 0.47 (q, *J* = 5.1 Hz, 2H, CH_2_). ^13^C NMR (101 MHz, CDCl_3_) *δ* 153.87, 151.84, 151.24, 148.70, 141.23, 139.43, 129.90, 125.63, 119.26, 118.26, 114.77, 48.86, 11.02, 4.42. ESI/MS for (C_15_H_13_ClN_6_O_2_ [M + H]^+^): Calcd: 345.8. Found: 345.0.

2-Chloro-9-(cyclopropylmethyl)-*N*-(4-(trifluoromethoxy)phenyl)-9*H*-purin-6-amine, (**3h**): White solid, yield 48%, mp 137–139 °C, R_f_ = 0.3. ^1^H NMR (400 MHz, CDCl_3_) *δ* 8.10 (s, 1H, NH), 7.92 (s, 1H, CH), 7.82 (d, *J* = 9.0 Hz, 2H, 2CH), 7.24 (d, *J* = 8.7 Hz, 2H, 2CH), 4.04 (d, *J* = 7.3 Hz, 2H, CH_2_), 1.40–1.26 (m, 1H, CH), 0.70 (q, *J* = 5.5 Hz, 2H, CH_2_), 0.46 (q, *J* = 5.2 Hz, 2H, CH_2_). ^13^C NMR (101 MHz, CDCl_3_) *δ* 154.11, 152.22, 151.03, 145.17 (d, ^3^*J_C-F_* = 1.9 Hz, C-OCF_3_), 140.85, 136.99, 122.00 (2C), 121.35 (2C), 120.67 (d, ^1^*J_C-F_* = 256.8 Hz, CF_3_), 119.28, 48.88, 11.18, 4.52 (2C). ^19^F NMR (376 MHz, CDCl_3_) *δ* −58.09 (s, 3F). ESI/MS for (C_16_H_13_ClF_3_N_5_O [M + H]^+^): Calcd: 384.1. Found: 383.8.

4-((2-Chloro-9-(cyclopropylmethyl)-9*H*-purin-6-yl)amino)benzonitrile, (**3i**): White solid, yield 18%, mp 151–152 °C, R_f_ = 0.2. ^1^H NMR (400 MHz, DMSO-*d*_6_) *δ* 10.75 (s, 1H, NH), 8.47 (s, 1H, CH), 8.13 (d, *J* = 8.9 Hz, 2H, 2CH), 7.82 (d, *J* = 8.8 Hz, 2H, 2CH), 4.05 (d, *J* = 7.3 Hz, 2H, CH_2_), 1.38–1.29 (m, 1H, CH), 0.59–0.53 (m, 2H, CH_2_), 0.49–0.44 (m, 2H, CH_2_). ^13^C NMR (101 MHz, DMSO-*d*_6_) δ 151.95, 151.75, 151.33, 143.51, 143.01, 133.00 (2C), 120.50 (2C), 119.35, 119.30, 104.57, 47.86, 11.20, 3.89 (2C). ESI/MS for (C_16_H_13_ClN_6_ [M + 3H-CNPh-Cl]^+^): Calcd: 191.1. Found: 191.1.

2-Chloro-9-(cyclopropylmethyl)-*N*-(4-nitrophenyl)-9*H*-purin-6-amine, (**3j**): Yellow solid, yield 21%, mp 155–156 °C, R_f_ = 0.2. ^1^H NMR (400 MHz, DMSO-*d*_6_) *δ* 10.85 (s, 1H, NH), 8.41 (s, 1H, CH), 8.15 (dd, *J* = 9.6, 2.4 Hz, 2H, 2CH), 8.09 (dd, *J* = 9.6, 2.3 Hz, 2H, 2CH), 3.97 (d, *J* = 7.3 Hz, 2H, CH_2_), 1.25 (ddt, *J* = 10.4, 7.5, 3.7 Hz, 1H, CH), 0.51–0.45 (m, 2H, CH_2_), 0.38 (q, *J* = 4.9 Hz, 2H, CH_2_). ^13^C NMR (101 MHz, DMSO-*d*_6_) *δ* 151.82, 151.49, 151.46, 145.56, 143.18, 141.78, 124.62 (2C), 119.93 (2C), 119.47, 47.82, 11.11, 3.83 (2C). ESI/MS for (C_15_H_13_ClN_6_O_2_ [M + H]^+^): Calcd: 345.1. ESI/MS for (C_15_H_13_ClN_6_O_2_ [M + 3H − NO_2_Ph; − Cl]^+^): Calcd: 191.1. Found: 191.1.

Ethyl 4-((2-chloro-9-(cyclopropylmethyl)-9*H*-purin-6-yl)amino)benzoate, (**3k**): White solid, yield 45%, mp 130–133 °C, R_f_ = 0.2. ^1^H NMR (400 MHz, CDCl_3_) *δ* 8.23 (s, 1H, NH), 8.06 (d, *J* = 8.8 Hz, 2H, 2CH), 7.93 (s, 1H, CH), 7.88 (d, *J* = 8.8 Hz, 2H, 2CH), 4.36 (q, *J* = 7.1 Hz, 2H, CH_2_), 4.04 (d, *J* = 7.3 Hz, 2H, CH_2_), 1.39 (t, *J* = 7.1 Hz, 3H, CH_3_), 1.31 (ddt, *J* = 10.4, 7.5, 4.0 Hz, 1H, CH), 0.69 (q, *J* = 5.9 Hz, 2H, CH_2_), 0.45 (q, *J* = 5.0 Hz, 2H, CH_2_). ^13^C NMR (101 MHz, CDCl_3_) *δ* 166.29, 153.97, 151.96, 151.18, 142.47, 141.10, 130.99 (2C), 125.49, 119.58 (2C), 119.17, 60.93, 48.88, 14.49, 11.16, 4.51 (2C). ESI/MS for (C_18_H_18_ClN_5_O_2_ [M + H]^+^): Calcd: 372.1). ESI/MS for (C_18_H_18_ClN_5_O_2_ [M + 3H + Na − OCH_2_CH_3_]^+^): Calcd: 352.1. Found: 352.1.

2-Chloro-*N*-(4-chloro-3-fluorophenyl)-9-(cyclopropylmethyl)-9*H*-purin-6-amine, (**3l**): White solid, yield 57%, mp 140–142 °C, R_f_ = 0.2. ^1^H NMR (400 MHz, CDCl_3_) δ 8.31 (s, 1H, NH), 7.93 (s, 1H, CH), 7.90 (d, *J* = 2.4 Hz, 1H, CH), 7.40–7.30 (m, 2H, 2CH), 4.04 (d, *J* = 7.3 Hz, 2H, CH_2_), 1.32 (tdd, *J* = 7.5, 6.2, 2.7 Hz, 1H, CH), 0.70 (q, *J* = 5.9 Hz, 2H, CH_2_), 0.46 (q, *J* = 4.9 Hz, 2H, CH_2_). ^13^C NMR (101 MHz, CDCl_3_) δ 158.15 (d, ^1^*J_C-F_* = 247.1 Hz), 154.00, 151.91, 151.09, 141.00, 138.41 (d, ^3^*J_C-F_* = 10.0 Hz), 130.62, 119.28, 116.22 (d, ^4^*J_C-F_* = 3.4 Hz), 115.32 (d, ^2^*J_C-F_* = 18.0 Hz), 108.72 (d, ^2^*J_C-F_* = 26.4 Hz), 48.92, 11.15, 4.52 (2C). ^19^F NMR (376 MHz, CDCl_3_) δ −112.99 (s, 1F). ESI/MS for (C_15_H_12_Cl_2_FN_5_ [M + H]^+^): Calcd: 352.1. Found: 352.1.

2-Chloro-*N*-(3-chloro-4-fluorophenyl)-9-(cyclopropylmethyl)-9*H*-purin-6-amine, (**3m**): White solid, yield 86%, mp 140–143 °C, R_f_ = 0,3. ^1^H NMR (400 MHz, CDCl_3_) *δ* 8.15 (s, 1H, NH), 7.95 (s, 1H, CH), 7.90 (dd, *J* = 6.4, 2.7 Hz, 1H, CH), 7.66–7.58 (m, 1H, CH), 7.14 (t, *J* = 8.8 Hz, 1H, CH), 4.04 (d, *J* = 7.3 Hz, 2H, CH_2_), 1.38–1.26 (m, 1H, CH), 0.70 (q, *J* = 5.5 Hz, 2H, CH_2_), 0.46 (q, *J* = 5.1 Hz, 2H, CH_2_). ^13^C NMR (101 MHz, CDCl_3_) *δ* 154.65 (d, ^1^*J_C-F_* = 246.1 Hz, CF), 154.08, 151.93, 150.88, 140.65, 134.77 (d, ^4^*J_C-F_* = 3.3 Hz), 122.39, 121.14 (d, ^2^*J_C-F_* = 18.7 Hz), 120.05 (d, ^3^*J_C-F_* = 6.8 Hz, CH), 118.77, 116.71 (d, ^2^*J_C-F_* = 22.1 Hz, CH), 48.83, 11.01, 4.41 (2C). ^19^F NMR (376 MHz, CDCl_3_) *δ* −121.09 (s, 1F). ESI/MS for (C_15_H_12_Cl_2_FN_5_ [M + H]^+^): Calcd: 351.0. Found: 352.0.

2-Chloro-9-(cyclopropylmethyl)-*N*-(3,4-dichlorophenyl)-9*H*-purin-6-amine, (**3n**): Brown solid, yield 66%, mp 124–126 °C, R_f_ = 0.3. ^1^H NMR (400 MHz, CDCl_3_) *δ* 8.34 (s, 1H, NH), 7.95 (d, *J* = 2.5 Hz, 1H, CH), 7.93 (s, 1H, CH), 7.63 (dt, *J* = 11.1, 5.5 Hz, 1H, CH), 7.38 (d, *J* = 8.8 Hz, 1H, CH), 4.03 (d, *J* = 7.3 Hz, 2H, CH_2_), 1.31 (ddd, *J* = 9.4, 6.1, 3.8 Hz, 1H, CH), 0.69 (q, *J* = 5.6 Hz, 2H, CH_2_), 0.45 (q, *J* = 5.1 Hz, 2H, CH_2_). ^13^C NMR (101 MHz, CDCl_3_) *δ* 154.04, 151.86, 151.08, 141.01, 137.86, 132.78, 130.63, 127.05, 121.81, 119.60, 119.10, 48.94, 11.10, 4.52 (2C). ESI/MS for (C_15_H_12_Cl_3_N_5_ [M + H]^+^): Calcd: 368.0. Found: 367.9.

2-Chloro-9-(cyclopropylmethyl)-*N*-(3-nitro-4-(trifluoromethoxy)phenyl)-9*H*-purin-6-amine, (**3o**): Yellow solid, yield 38%, mp 136–138 °C, R_f_ = 0.2. ^1^H NMR (400 MHz, CDCl_3_) *δ* 9.16 (s, 1H, NH), 8.48 (d, *J* = 2.7 Hz, 1H, CH), 8.12 (dd, *J* = 9.1, 2.8 Hz, 1H, CH), 7.98 (s, 1H, CH), 7.39 (dd, *J* = 9.0, 0.9 Hz, 1H, CH), 4.04 (d, *J* = 7.3 Hz, 2H, CH_2_), 1.38–1.28 (m, 1H, CH), 0.68 (q, *J* = 5.7 Hz, 2H, CH_2_), 0.45 (q, *J* = 5.1 Hz, 2H, CH_2_). ^13^C NMR (101 MHz, CDCl_3_) *δ* 153.78, 151.61, 151.40, 142.79, 141.57, 138.25, 136.06 (d, ^3^*J_C-F_* = 2.1 Hz, C-OCF_3_), 124.78, 124.23, 120.34 (d, ^1^*J_C-F_* = 260.2 Hz, CF_3_), 119.22, 116.55, 49.00, 11.00, 4.46 (2C). ^19^F NMR (376 MHz, CDCl_3_) *δ* −57.93 (s, 3F). ESI/MS for (C_16_H_12_ClF_3_N_6_O_3_ [M + H]^+^): Calcd: 429.1. Found: 428.9.

2-Chloro-9-isopentyl-*N*-phenyl-9*H*-purin-6-amine, (**3p**): Brown solid, yield 88%, mp 110.1–113.8 °C, R_f_ = 0.3. ^1^H NMR (400 MHz, CDCl_3_) δ 7.96 (s, 1H, NH), 7.84 (s, 1H, CH), 7.78 (d, *J* = 8.6 Hz, 2H, 2CH), 7.39 (t, *J* = 8.0 Hz, 2H, 2CH), 7.14 (td, *J* = 7.6, 0.9 Hz, 1H, CH), 4.27–4.12 (m, 2H, CH_2_), 1.79 (dt, *J* = 7.4, 5.5 Hz, 2H, CH_2_), 1.69–1.53 (m, 1H, CH), 0.99 (d, *J* = 6.6 Hz, 6H, 2CH_3_). ^13^C NMR (101 MHz, CDCl_3_) δ 154.41, 154.39, 152.32, 150.80, 140.58, 138.16, 129.27 (2C), 124.22, 120.38 (2C), 42.67, 38.81, 25.79, 22.40 (2C). ESI/MS for (C_16_H_18_ClN_5_ [M + H]^+^): Calcd: 316.1. Found: 316.1.

2-Chloro-*N*-(3-fluorophenyl)-9-isopentyl-9*H*-purin-6-amine, (**3q**): White solid, yield 50%, mp 136.2–138.7 °C, R_f_ = 0.4. ^1^H NMR (400 MHz, CDCl_3_) δ 8.00 (s, 1H, NH), 7.82 (s, 1H, CH) 7.79 (td, *J* = 2.2, 2.4Hz, 1H, CH), 7.40 (dd, *J* = 8.2, 1.2 Hz, 1H, CH), 7.31 (td, *J* = 8.2, 6.5 Hz, 1H, CH), 6.82 (td, J = 8.2, 2.2 Hz, 1H, CH), 4.28–4.17 (m, 2H, CH_2_), 1.80 (dd, *J* = 14.9, 7.1 Hz, 2H, CH_2_), 1.61 (dp, *J* = 13.3, 6.7 Hz, 1H, CH), 0.99 (t, *J* = 5.1 Hz, 6H, 2CH_3_). ^13^C NMR (101 MHz, CDCl_3_) δ 163.07 (d, ^1^*J_C-F_* = 244.6 Hz), 151.83, 150.79, 140.63 (d, ^3^*J_C-F_* = 8.4 Hz), 139.68, 139.59, 130.16 (d, ^3^*J_C-F_* = 9.5 Hz), 118.73, 115.38 (d, ^4^*J_C-F_* = 2.8 Hz), 110.62 (d, ^2^*J_C-F_* = 19.7 Hz), 107.52 (d, ^2^*J_C-F_* = 26.6 Hz), 42.59, 38.65, 25.65, 22.25 (2C). ^19^F NMR (376 MHz, CDCl_3_) δ −111.11 (s, 1F). ESI/MS for (C_16_H_17_ClFN_5_ [M + H]^+^): Calcd: 334.1. Found: 334.1.

2-Chloro-*N*-(3,4-difluorophenyl)-9-isopentyl-9*H*-purin-6-amine, (**3r**): Yellow solid, yield 67%, mp 141.3–144.1 °C, R_f_ = 0.5. ^1^H NMR (400 MHz, CDCl_3_) δ 8.22 (s, 1H, NH), 7.86 (ddd, *J* = 12.3, 7.1, 2.6 Hz, 1H, CH), 7.76 (s, 1H, CH), 7.31–7.26 (m, 1H, CH), 7.11 (dd, *J* = 18.6, 9.0 Hz, 1H, CH), 4.24–4.12 (m, 2H, CH_2_), 1.77 (dd, *J* = 14.9, 7.1 Hz, 2H, CH_2_), 1.58 (dhept, *J* = 13.4, 6.6 Hz, 1H, CH), 0.97 (d, *J* = 6.6 Hz, 6H, 2CH_3_). ^13^C NMR (101 MHz, CDCl_3_) δ 154.02, 152.09, 151.04, 150.18 (dd, ^1,2^*J_C-F_* = 246.9, 13.3 Hz), 146.88 (dd, ^1,2^*J_C-F_* = 245.2, 12.8 Hz), 141.18, 134.81 (dd, ^3,4^*J_C-F_* = 8.9, 3.1 Hz), 119.20, 117.36 (d, ^1^*J_C-F_* = 18.2 Hz), 115.99 (dd, ^3,4^*J_C-F_* = 5.8, 3.6 Hz), 110.06 (d, ^2^*J_C-F_* = 22.0 Hz), 42.60, 38.76, 25.73, 22.32 (2C). ^19^F NMR (376 MHz, CDCl_3_) δ −135.33 (d, *J_F-F_* = 21.8 Hz, 1F), -143.21 (d, *J_F-F_* = 21.8 Hz, 1F). ESI/MS for (C_16_H_16_ClF_2_N_5_ [M + H]^+^): Calcd: 352.8. Found: 352.1.

2-Chloro-9-pentyl-*N*-phenyl-9*H*-purin-6-amine, (**3s**): Brown solid, yield 72%, mp 103.5–106.3 °C, R_f_ = 0.6. ^1^H NMR (400 MHz, CDCl_3_) δ 8.05 (s, 1H, NH), 7.79 (s, 1H, CH), 7.77 (d, *J* = 3.0 Hz, 2H, 2CH), 7.39 (t, *J* = 8.0 Hz, 2H, 2CH), 7.13 (t, *J* = 7.4 Hz, 1H, CH), 4.17 (t, *J* = 7.3 Hz, 2H, CH_2_), 1.96–1.85 (m, 2H, CH_2_), 1.40–1.25 (m, 4H, 2CH_2_), 0.90 (t, *J* = 7.0 Hz, 3H, CH_3_). ^13^C NMR (101 MHz, CDCl_3_) δ 154.32, 152.38, 150.85, 140.81, 138.16, 129.27 (2C), 124.19, 120.37 (2C), 119.10, 44.30, 29.82, 28.84, 22.27, 14.01. ESI/MS for (C_16_H_18_ClN_5_ [M + H]^+^): Calcd: 316.1. Found: 316.4.

2-Chloro-*N*-(3-fluorophenyl)-9-pentyl-9*H*-purin-6-amine, (**3t**): Yellow solid, yield 40%, mp 118.9–123.7 °C, R_f_ = 0.6. ^1^H NMR (200 MHz, CDCl_3_) δ 8.21 (s, 1H, NH), 7.85–7.76 (m, 2H, 2CH), 7.39 (t, *J* = 8.9 Hz, 1H, CH), 7.32 (dd, *J* = 8.4, 2.1 Hz, 1H, CH), 6.91–6.75 (m, 1H, CH), 4.20 (t, *J* = 7.3 Hz, 2H, CH_2_), 2.03–1.81 (m, 2H, CH_2_), 1.44–1.26 (m, 4H, 2CH_2_), 0.91 (t, *J* = 6.6 Hz, 3H, CH_3_). ^13^C NMR (50 MHz, CDCl_3_) δ 163.15 (d, ^1^*J_C-F_* = 244.6 Hz), 154.07, 152.13, 151.01, 141.24, 139.83 (d, ^3^*J_C-F_* = 11.1 Hz), 130.23 (d, ^3^*J_C-F_* = 9.5 Hz), 119.30, 115.53 (d, ^4^*J_C-F_* = 2.9 Hz), 110.63 (d, ^2^*J_C-F_* = 21.5 Hz), 107.65 (d, ^2^*J_C-F_* = 26.7 Hz), 44.27, 29.77, 28.79, 22.22, 13.96. ^19^F NMR (188 MHz, CDCl_3_) δ −111.12 (s, 1F). ESI/MS for (C_16_H_17_ClFN_5_ [M + H]^+^): Calcd: 334.1. Found: 334.1.

2-Chloro-*N*-(3,4-difluorophenyl)-9-pentyl-9*H*-purin-6-amine, (**3u**): Brown solid, yield 72%, mp 139.1–142.7 °C, R_f_ = 0.4. ^1^H NMR (400 MHz, CDCl_3_) δ 8.40 (s, 1H, NH), 7.87 (ddd, *J* = 12.4, 7.1, 2.6 Hz, 1H, CH), 7.76 (s, 1H, CH), 7.35–7.27 (m, 1H, CH), 7.11 (dd, *J* = 18.7, 8.9 Hz, 1H, CH), 4.16 (t, *J* = 7.3 Hz, 2H, CH_2_), 1.94–1.80 (m, 2H, CH_2_), 1.43–1.21 (m, 4H, 2CH_2_), 0.88 (t, *J* = 7.0 Hz, 3H, CH_3_). ^13^C NMR (101 MHz, CDCl_3_) δ 154.04, 152.07, 150.98, 150.16 (dd, ^1,2^*J_C-F_* = 246.8, 13.3 Hz), 146.83 (dd, ^1,2^*J_C-F_* = 245.1, 12.9 Hz), 141.18, 134.90 (dd, ^3,4^*J_C-F_* = 8.9, 3.1 Hz), 119.10, 117.33 (d, ^2^*J_C-F_* = 17.1 Hz), 115.92 (dd, ^3,4^*J_C-F_* = 5.8, 3.6 Hz), 109.96 (d, ^2^*J_C-F_* = 22.0 Hz), 44.27, 29.75, 28.77, 22.21, 13.93. ^19^F NMR (376 MHz, CDCl_3_) δ −135.43 (d, *J* = 21.8 Hz, 1F), −143.34 (d, *J* = 21.8 Hz, 1F). ESI/MS for (C_16_H_16_ClF_2_N_5_ [M + H]^+^): Calcd: 351.1. Found: 351.8.

2-Chloro-9-hexyl-*N*-phenyl-9*H*-purin-6-amine, (**3v**): Yellow solid, yield 71%, mp 115.1–118.9 °C, R_f_ = 0.4. ^1^H NMR (400 MHz, CDCl_3_) δ 8.06 (s, 1H, NH), 7.81 (s, 1H, CH), 7.79 (dd, *J* = 8.6, 0.9 Hz, 2H, 2CH), 7.41–7.37 (m, 2H, 2CH), 7.13 (t, *J* = 7.4 Hz, 1H, CH), 4.18 (t, *J* = 7.3 Hz, 2H, CH_2_), 1.96–1.81 (m, 2H, CH_2_), 1.39–1.27 (m, 6H, 3CH_2_), 0.88 (t, *J* = 7.0 Hz, 3H, CH_3_). ^13^C NMR (101 MHz, CDCl_3_) δ 154.44, 154.42, 152.27, 150.76, 140.60, 138.15, 129.26 (2C), 124.22, 120.38 (2C), 44.38, 31.31, 30.08, 26.40, 22.59, 14.08. ESI/MS for (C_17_H_20_ClN_5_ [M + H]^+^): Calcd: 330.1. Found: 330.2.

2-Chloro-*N*-(3-fluorophenyl)-9-hexyl-9*H*-purin-6-amine, (**3w**): Yellow solid, yield 78%, mp 129.2–133.5 °C, R_f_ = 0.5. ^1^H NMR (400 MHz, CDCl_3_) δ 8.27 (s, 1H, NH), 7.66 (dt, *J* = 10.1, 2.2 Hz, 1H, CH), 7.64 (s, 1H, CH), 7.27 (dd, *J* = 8.1, 1.3 Hz, 1H, CH), 7.15 (td, *J* = 8.1, 4.1 Hz, 1H, CH), 6.67 (td, *J* = 8.3, 2.3 Hz, 1H, CH), 4.03 (t, *J* = 7.3 Hz, 2H, CH_2_), 1.73 (dd, *J* = 14.2, 7.1 Hz, 2H, CH_2_), 1.26–1.07 (m, 6H, 3CH_2_), 0.74 (t, *J* = 6.9 Hz, 3H, CH_3_). ^13^C NMR (101 MHz, CDCl_3_) δ 163.14 (d, ^1^*J_C-F_* = 244.4 Hz), 154.06, 152.13, 150.98, 141.12, 139.93 (d, ^3^*J_C-F_* = 11.1 Hz), 130.20 (d, ^3^*J_C-F_* = 9.5 Hz), 119.24, 115.51 (d, ^4^*J_C-F_* = 2.9 Hz), 110.55 (d, ^2^*J_C-F_* = 21.4 Hz), 107.60 (d, ^2^*J_C-F_* = 26.7 Hz), 44.27, 31.26, 30.04, 26.36, 22.55, 14.03. ^19^F NMR (376 MHz, CDCl_3_) δ −111.19 (s, 1F). ESI/MS for (C_17_H_19_ClFN_5_ [M + H]^+^): Calcd: 348.1. Found: 348.1.

2-Chloro-*N*-(3,4-difluorophenyl)-9-hexyl-9*H*-purin-6-amine, (**3x**): Brown solid, yield 82%, mp 147.3–150.1 °C, R_f_ = 0.5. ^1^H NMR (400 MHz, CDCl_3_) δ 7.95 (s, 1H, NH), 7.89 (ddd, *J* = 12.3, 7.1, 2.7 Hz, 1H, CH), 7.78 (s, 1H, CH), 7.31 (dt, *J* = 11.6, 3.9 Hz, 1H, CH), 7.14 (dd, *J* = 18.6, 8.9 Hz, 1H, CH), 4.18 (t, *J* = 7.3 Hz, 2H, CH_2_), 1.94–1.82 (m, 2H, CH_2_), 1.36–1.24 (m, 6H, 3CH_2_), 0.87 (t, *J* = 7.0 Hz, 3H, CH_3_). ^13^C NMR (101 MHz, CDCl_3_) δ 154.05, 152.11, 151.08, 150.27 (dd, ^1,2^*J_C-F_* = 247.1, 13.3 Hz), 146.96 (dd, ^1,2^*J_C-F_* = 245.3, 12.9 Hz), 141.33, 134.77 (dd, ^2,3^*J_C-F_* = 9.0, 3.1 Hz), 119.30, 117.45 (d, ^2^*J_C-F_* = 18.3 Hz), 115.93 (dd, ^3,4^*J_C-F_* = 5.8, 3.6 Hz), 110.05 (d, ^2^*J_C-F_* = 22.0 Hz), 44.31, 31.29, 30.09, 26.39, 22.57, 14.05. ^19^F NMR (376 MHz, CDCl_3_) δ −135.29 (d, *J* = 21.8 Hz, 1F), −143.19 (d, *J* = 21.8 Hz,1F). ESI/MS for (C_17_H_18_ClF_2_N_5_ [M + H]^+^): Calcd: 366.1. Found: 365.8.

2-Chloro-9-octyl-*N*-phenyl-9*H*-purin-6-amine, (**3y**): Yellow solid, yield 61%, mp 126.2–129.7 °C, R_f_ = 0.4. ^1^H NMR (400 MHz, CDCl_3_) δ 8.03 (s, 1H, NH), 7.79 (s, 1H, CH), 7.78 (d, *J* = 5.9 Hz, 2H, 2CH), 7.43–7.33 (m, 2H, 2CH), 7.13 (t, *J* = 7.4 Hz, 1H, CH), 4.17 (t, *J* = 7.3 Hz, 2H, CH_2_), 1.88 (p, *J* = 7.3 Hz, 2H, CH_2_), 1.44–1.07 (m, 10H, 5CH_2_), 0.87 (t, *J* = 6.9 Hz, 3H, CH_3_). ^13^C NMR (101 MHz, CDCl_3_) δ 154.34, 152.34, 150.80, 140.72, 138.18, 129.25 (2C), 124.18, 120.38 (2C), 118.99, 44.33, 31.83, 30.12, 29.21, 29.11, 26.73, 22.72, 14.19. ESI/MS for (C_19_H_24_ClN_5_ [M + H]^+^): Calcd: 358.2. Found: 358.2.

2-Chloro-*N*-(3-fluorophenyl)-9-octyl-9*H*-purin-6-amine, (**3z**): Yellow solid, yield 83%, mp 147.6–150.8 °C, R_f_ = 0.5. ^1^H NMR (200 MHz, CDCl_3_) δ 8.32 (s, 1H, NH), 7.88–7.75 (m, 2H, 2CH), 7.40 (t, *J* = 6.8 Hz, 1H, CH), 7.32 (dd, *J* = 8.0, 1.6 Hz, 1H, CH), 6.90–6.76 (m, 1H, CH), 4.20 (t, *J* = 7.3 Hz, 2H, CH_2_), 1.99–1.82 (m, 2H, CH_2_), 1.31 (d, *J* = 12.0 Hz, 10H, 5CH_2_), 0.89 (t, *J* = 6.3 Hz, 3H, CH_3_). ^13^C NMR (50 MHz, CDCl_3_) δ 163.14 (d, ^1^*J_C-F_* = 244.6 Hz), 154.14, 152.05, 150.95, 141.13, 139.83 (d, ^3^*J* = 11.1 Hz), 130.22 (d, ^3^*J_C-F_* = 9.5 Hz), 119.07, 115.54 (d, ^3^*J_C-F_* = 2.9 Hz), 110.63 (d, ^2^*J_C-F_* = 21.4 Hz), 107.66 (d, ^2^*J_C-F_* = 26.7 Hz), 44.32, 31.78, 30.06, 29.16, 29.06, 26.68, 22.68, 14.14. ^19^F NMR (188 MHz, CDCl_3_) δ −111.12 (s, 1F). ESI/MS for (C_19_H_23_ClFN_5_ [M + H]^+^): Calcd: 376.2. Found: 375.8.

2-Chloro-*N*-(3,4-difluorophenyl)-9-octyl-9*H*-purin-6-amine, (**3aa**): White solid, yield 71%, mp 168.2–171.2 °C, R_f_ = 0.6. ^1^H NMR (400 MHz, CDCl_3_) δ 8.29 (s, 1H, NH), 7.89 (ddd, *J* = 12.3, 7.1, 2.6 Hz, 1H, CH), 7.78 (s, 1H, CH), 7.36–7.29 (m, 1H, CH), 7.12 (q, *J* = 9.2 Hz, 1H, CH), 4.17 (t, *J* = 7.3 Hz, 2H, CH_2_), 1.96–1.79 (m, 2H, CH_2_), 1.27 (dd, *J* = 18.4, 10.5 Hz, 10H, 5CH_2_), 0.86 (t, *J* = 6.8 Hz, 3H, CH_3_). ^13^C NMR (101 MHz, CDCl_3_) δ 153.97, 151.94, 150.87, 150.08 (dd, ^1,2^*J_C-F_* = 246.9, 13.3 Hz), 146.75 (dd, ^1,2^*J_C-F_* = 245.2, 12.5 Hz), 141.03, 134.77 (d, ^3^*J_C-F_* = 5.7 Hz), 118.96, 117.25 (d, ^2^*J_C-F_* = 18.3 Hz), 115.78 (dd, ^3,4^*J_C-F_* = 5.7, 3.5 Hz), 109.84 (d, ^2^*J_C-F_* = 22.0 Hz), 44.19, 31.68, 29.96, 29.05, 28.95, 26.58, 22.57, 14.03. ^19^F NMR (376 MHz, CDCl_3_) δ -135.40 (d, *J* = 21.8 Hz, 1F), −143.32 (d, *J* = 21.8 Hz, 1F). ESI/MS for (C_19_H_22_ClF_2_N_5_ [M + H]^+^): Calcd: 394.2. Found: 394.0.

2-Chloro-9-(cyclopropylmethyl)-*N*-phenyl-9*H*-purin-6-amine, (**3ab**): White solid, yield 85%. The analytical data corresponded to literature [44].

2-Chloro-9-(cyclopropylmethyl)-*N*-(3-fluorophenyl)-9*H*-purin-6-amine, (**3ac**): White solid, yield 76%. The analytical data corresponded to literature [44].

2-Chloro-9-(cyclopropylmethyl)-*N*-(3,4-difluorophenyl)-9*H*-purin-6-amine, (**3ad**): White solid, yield 77%. The analytical data corresponded to literature [44].

#### 2.1.3. General Procedures for the Synthesis of Compounds **4a**–**4o**

Derivatives **3a**–**3o** (0.52 mmol) were dissolved in dioxane (2 mL) in a tube for MW reactor. Then, 4-(4-methylpiperazin-1-yl)aniline (110 mg, 0.57 mmol), Pd(OAc)_2_ (24 mg, 0.1 mmol), Xantphos (120 mg, 0.2 mmol) and 2 M K_2_CO_3_ (aq) (1 mL, 0.2 mmol) were added, and the reaction mixture was stirred for 1 h at 100 °C. Then, the suspension was cooled at room temperature and filtered on celite, and filtrate was added H_2_O (30 mL). The mixture was extracted with EtOAc, and the organic layer was dried by anhydrous Na_2_SO_4_ and removed under vacuum. The crude product was purified by column chromatography on silica gel using methanol/dichloromethane (10:90) as the mobile phase to yield products **4a**–**4o**. The Rf values of each compound of this series were calculated using the mentioned mobile phase.

9-(Cyclopropylmethyl)-*N*^6^-(2-fluorophenyl)-*N*^2^-(4-(4-methylpiperazin-1-yl)phenyl)-9*H*-purine-2,6-diamine, (**4a**): Green solid, yield 61%, mp 149–152 °C, R_f_ = 0.3. ^1^H NMR (400 MHz, CDCl_3_) *δ* 8.53 (td, *J* = 8.5, 1.8 Hz, 1H, CH), 7.68 (s, 2H, NH, CH), 7.50 (d, *J* = 8.9 Hz, 2H, 2CH), 7.12–7.03 (m, 2H, 2CH), 7.00–6.93 (m, 1H, CH), 6.90 (d, *J* = 8.9 Hz, 2H, 2CH), 6.90 (s, 1H, NH), 3.91 (d, *J* = 7.2 Hz, 2H, CH_2_), 3.21–3.12 (m, 4H, 2CH_2_), 2.66–2.57 (m, 4H, 2CH_2_), 2.35 (s, 3H, CH_3_), 1.34–1.23 (m, 1H, CH), 0.67–0.61 (m, 2H, CH_2_), 0.42 (m, 2H, CH_2_). ^13^C NMR (101 MHz, CDCl_3_) *δ* 156.70, 153.01 (d, ^1^*J*_C-F_ = 243.9 Hz, CF), 151.87, 151.57, 146.81, 138.17, 133.20, 127.49 (d, ^2^*J* = 9.8 Hz), 124.09 (d, ^4^*J_C-F_* = 3.7 Hz, CH), 122.95 (d, *J* = 7.5 Hz, CH), 122.22, 121.13 (2C), 117.01 (2C), 115.71, 114.86 (d, ^2^*J_C-F_* = 19.2 Hz, CH), 55.15 (2C), 49.93 (2C), 48.13, 46.03, 11.10, 4.29 (2C). ^19^F NMR (376 MHz, CDCl_3_) *δ* −130.72 (s, 1F). ESI/MS for (C_26_H_29_FN_8_ [M + H]^+^): Calcd: 473.2572. Found: 473.2578.

*N*^6^-(3-Chlorophenyl)-9-(cyclopropylmethyl)-*N*^2^-(4-(4-methylpiperazin-1-yl)phenyl)-9*H*-purine-2,6-diamine, (**4b**): Green solid, yield 50%, mp 152–153 °C, R_f_ = 0.3. ^1^H NMR (400 MHz, CDCl_3_) *δ* 7.98 (t, *J* = 2.0 Hz, 1H, CH), 7.93 (s, 1H, NH), 7.69 (s, 1H, CH), 7.53 (d, *J* = 8.9 Hz, 2H, 2CH), 7.44 (dd, *J* = 8.2, 1.3 Hz, 1H, CH), 7.19 (t, *J* = 8.1 Hz, 1H, CH), 7.02 (s, 1H, NH), 7.00 (ddd, *J* = 8.0, 2.0, 0.9 Hz, 1H, CH), 6.95 (d, *J* = 9.0 Hz, 2H, 2CH), 3.93 (d, *J* = 7.2 Hz, 2H, CH_2_), 3.23–3.14 (m, 4H, 2CH_2_), 2.67–2.57 (m, 4H, 2CH_2_), 2.36 (s, 3H, CH_3_), 1.31 (ddt, *J* = 14.6, 6.7, 4.6 Hz, 1H, CH), 0.67 (q, *J* = 5.9 Hz, 2H, CH_2_), 0.44 (q, *J* = 4.9 Hz, 2H, CH_2_). ^13^C NMR (101 MHz, CDCl_3_) *δ* 156.78, 151.83, 151.48, 146.85, 140.35, 138.03, 134.45, 132.99, 129.70, 122.80, 121.06, 119.93, 117.93, 117.10, 115.27, 55.21, 49.92, 48.14, 46.11, 11.05, 4.30. ESI/MS for (C_26_H_29_ClN_8_ [M + H]^+^): Calcd: 489.2276. Found: 489.2279.

9-(Cyclopropylmethyl)-*N*^2^-(4-(4-methylpiperazin-1-yl)phenyl)-*N*^6^-(*m*-tolyl)-9*H*-purine-2,6-diamine, (**4c**): Brown solid, yield 66%, mp 135–137 °C, R_f_ = 0.3. ^1^H NMR (400 MHz, CDCl_3_) *δ* 7.69 (s, 1H, CH), 7.63 (s, 1H, NH), 7.55 (d, *J* = 8.8 Hz, 2H, CH), 7.54 (m, 2H, 2CH), 7.20 (t, *J* = 7.9 Hz, 1H, CH), 6.94 (s, 1H, NH), 6.93 (d, *J* = 9.0 Hz, 2H, CH), 6.88 (d, *J* = 7.5 Hz, 1H, CH), 3.94 (d, *J* = 7.2 Hz, 2H, CH_2_), 3.26–3.13 (m, 4H, 2CH_2_), 2.67–2.63 (m, 4H, 2CH_2_), 2.40 (s, 3H, CH_3_), 2.33 (s, 3H, CH_3_), 1.31 (m, 1H, CH), 0.67 (q, *J* = 5.8 Hz, 2H, CH_2_), 0.45 (q, *J* = 5.0 Hz, 2H, CH_2_). ^13^C NMR (101 MHz, CDCl_3_) *δ* 156.90, 152.33, 151.36, 146.66, 139.04, 138.80, 137.90, 133.58, 128.78, 124.03, 120.99 (2C), 120.89, 117.54, 117.26 (2C), 115.46, 55.25 (2C), 50.00 (2C), 48.22, 46.08, 21.78, 11.21, 4.41 (2C). ESI/MS for (C_27_H_32_N_8_ [M + H]^+^): Calcd: 469.2823. Found: 469.2833.

9-(Cyclopropylmethyl)-*N^2^*-(4-(4-methylpiperazin-1-yl)phenyl)-*N^6^*-(3-(trifluoromethyl)phenyl)-9*H*-purine-2,6-diamine, (**4d**): Brown solid, yield 51%, mp 134–135 °C, R_f_ = 0.3. ^1^H NMR (400 MHz, CDCl_3_) δ 8.19 (s, 1H), 7.97 (s, 1H, NH), 7.91 (d, *J* = 8.2 Hz, 1H, CH), 7.69 (s, 1H, CH), 7.52 (d, *J* = 8.9 Hz, 2H, 2CH), 7.36 (t, *J* = 7.9 Hz, 1H, CH), 7.27 (d, *J* = 5.8 Hz, 1H, CH), 7.10 (s, 1H, NH), 6.92 (d, *J* = 9.0 Hz, 2H, CH), 3.93 (d, *J* = 7.2 Hz, 2H, CH_2_), 3.22–3.13 (m, 4H, 2CH_2_), 2.65–2.55 (m, 4H, 2CH_2_), 2.37 (s, 3H, CH_3_), 1.34–1.24 (m, 1H, CH), 0.66 (q, *J* = 5.8 Hz, 2H, CH_2_), 0.44 (q, *J* = 5.0 Hz, 2H, CH_2_). ^13^C NMR (101 MHz, CDCl_3_) δ 156.83, 151.99, 151.66, 146.94, 139.81, 138.28, 133.12, 131.19 (d, ^2^*J_C-F_* = 32.1 Hz), 129.34, 124.15 (dd, ^1^*J_C-F_* = 797.4, 525.0 Hz), 123.31, 121.03 (2C), 119.39 (d, ^3^*J_C-F_* = 3.9 Hz), 117.13 (2C), 116.70 (d, ^3^*J_C-F_* = 3.9 Hz), 115.37, 55.27 (2C), 49.99 (2C), 48.27, 46.16, 11.12, 4.39 (2C). ^19^F NMR (376 MHz, CDCl_3_) δ −62.48 (s, 3F, CF_3_). ESI/MS for (C_27_H_29_F_3_N_8_ [M + H]^+^): Calcd: 523.2540. Found: 523.2552.

9-(Cyclopropylmethyl)-*N*^2^-(4-(4-methylpiperazin-1-yl)phenyl)-*N*^6^-(3-(trifluoromethoxy)phenyl)-9*H*-purine-2,6-diamine, (**4e**): Red solid, yield 63%, mp 161–162 °C, R_f_ = 0.3. ^1^H NMR (400 MHz, CDCl_3_) *δ* 8.09 (s, 1H, NH), 7.86 (s, 1H, CH), 7.69 (s, 1H, CH), 7.54 (m, 3H, 3CH), 7.27 (t, *J* = 8.2 Hz, 1H, CH), 7.01 (s, 1H, NH), 6.94 (d, *J* = 8.9 Hz, 2H, 2CH), 6.89 (d, *J* = 8.2 Hz, 1H, CH), 3.93 (d, *J* = 7.1 Hz, 2H, CH_2_), 3.23–3.14 (m, 4H, 2CH_2_), 2.67–2.58 (m, 4H, 2CH_2_), 2.37 (s, 3H, CH_3_), 1.30 (dt, *J* = 8.1, 5.1 Hz, 1H, CH), 0.66 (q, *J* = 5.4 Hz, 2H, CH_2_), 0.44 (q, *J* = 4.9 Hz, 2H, CH_2_). ^13^C NMR (101 MHz, CDCl_3_) *δ* 156.82, 151.93, 151.62, 149.66 (d, ^3^*J_C-F_* = 1.5 Hz, COCF_3_), 146.98, 140.78, 138.23, 133.10, 129.81, 121.08 (2C), 120.64 (d, ^1^*J*_C-F_ = 257.2 Hz, CF_3_), 118.05, 117.10 (2C), 115.41, 114.66, 112.71, 55.29 (2C), 49.97 (2C), 48.28, 46.18, 11.15, 4.40 (2C). ^19^F NMR (376 MHz, CDCl_3_) *δ* −57.49 (s, 3F, CF_3_). ESI/MS for (C_27_H_29_F_3_N_8_O [M + H]^+^): Calcd: 539.2489. Found: 539.2496.

3-((9-(Cyclopropylmethyl)-2-((4-(4-methylpiperazin-1-yl)phenyl)amino)-9*H*-purin-6-yl)amino)benzonitrile, (**4f**): Red solid, yield 62%, mp 159–160 °C, R_f_ = 0.2. ^1^H NMR (400 MHz, CDCl_3_) δ 8.30 (s, 1H, NH), 8.26 (s, 1H, CH), 7.71 (s, 1H, CH), 7.70 (d, *J* = 6.2 Hz, 1H, 1CH), 7.53 (d, *J* = 8.8 Hz, 2H, 2CH), 7.34–7.27 (m, 1H, CH), 7.25 (d, *J* = 7.2 Hz, 1H, CH), 7.17 (s, 1H, NH), 6.97 (d, *J* = 8.9 Hz, 2H, 2CH), 3.93 (d, *J* = 7.1 Hz, 2H, CH_2_), 3.25–3.17 (m, 4H, 2CH_2_), 2.64–2.52 (m, 4H, 2CH_2_), 2.35 (s, 3H, CH_3_), 1.37–1.25 (m, 1H, CH), 0.66 (q, *J* = 5.4 Hz, 2H, CH_2_), 0.44 (q, *J* = 4.9 Hz, 2H, CH_2_). ^13^C NMR (101 MHz, CDCl_3_) δ 156.82, 151.74, 151.69, 147.12, 140.20, 138.38, 132.80, 129.57, 126.11, 123.84, 122.75, 121.21 (2C), 119.09, 117.04 (2C), 115.28, 112.77, 6, 55.29 (2C), 49.89 (2C), 48.29, 46.22, 11.10, 4.41 (2C). ESI/MS for (C_27_H_29_N_9_ [M + H]^+^): Calcd: 480.2619. Found: 480.2614.

9-(Cyclopropylmethyl)-*N*^2^-(4-(4-methylpiperazin-1-yl)phenyl)-*N*^6^-(3-nitrophenyl)-9*H*-purine-2,6-diamine, (**4g**): Orange solid, yield 40%, mp 165–166 °C, R_f_ = 0.2. ^1^H NMR (400 MHz, CDCl_3_) *δ* 8.66 (s, 1H, CH), 8.30 (s, 1H, NH), 7.96 (dd, *J* = 8.1, 1.2 Hz, 1H, CH), 7.83 (dd, *J* = 8.1, 1.7 Hz, 1H, CH), 7.73 (s, 1H, NH), 7.53 (d, *J* = 8.9 Hz, 2H, 2CH), 7.38 (t, *J* = 8.2 Hz, 1H, CH), 7.09 (s, 1H, NH), 6.93 (d, *J* = 8.9 Hz, 2H, 2CH), 3.95 (d, *J* = 7.2 Hz, 2H, CH_2_), 3.25–3.16 (m, 4H, 2CH_2_), 2.70–2.61 (m, 4H, 2CH_2_), 2.39 (s, 3H, CH_3_), 1.32 (m, 1H, CH), 0.68 (q, *J* = 5.5 Hz, 2H, CH_2_), 0.46 (q, *J* = 5.0 Hz, 2H, CH_2_). ^13^C NMR (101 MHz, CDCl_3_) *δ* 156.76, 151.86, 151.73, 148.74, 147.01, 140.50, 138.53, 132.98, 129.48, 125.57, 121.24 (2C), 117.32, 117.12 (2C), 115.39, 114.66, 55.22 (2C), 49.90 (2C), 48.35, 46.11, 11.14, 4.44 (2C). ESI/MS for (C_26_H_29_N_9_O_2_ [M + H]^+^): Calcd: 500.2517. Found: 500.2521.

9-(Cyclopropylmethyl)-*N*^2^-(4-(4-methylpiperazin-1-yl)phenyl)-*N*^6^-(4-(trifluoromethoxy)phenyl)-9*H*-purine-2,6-diamine, (**4h**): White solid, yield 39%, mp 161–162 °C, R_f_ = 0.2. ^1^H NMR (400 MHz, CDCl_3_) δ 7.80 (s, 1H, NH), 7.74 (d, *J* = 9.0 Hz, 2H, 2CH), 7.69 (s, 1H, CH), 7.53 (d, *J* = 8.9 Hz, 2H, 2CH), 7.14 (d, *J* = 8.6 Hz, 2H, 2CH), 6.97 (s, 1H, NH), 6.93 (d, *J* = 8.9 Hz, 2H, 2CH), 3.94 (d, *J* = 7.2 Hz, 2H, CH_2_), 3.23–3.14 (m, 4H, 2CH_2_), 2.66–2.55 (m, 4H, 2CH_2_), 2.37 (s, 3H, CH_3_), 1.39–1.26 (m, 1H, CH), 0.67 (q, *J* = 5.6 Hz, 2H, CH_2_), 0.45 (q, *J* = 5.0 Hz, 2H, CH_2_). ^13^C NMR (101 MHz, CDCl_3_) δ 156.89, 152.01, 151.63, 147.08, 144.44 (d, ^3^*J_C-F_* = 1.7 Hz), 138.20, 137.93, 133.13, 121.68 (2C), 121.31 (2C), 121.27 (2C), 120.71 (d, ^1^*J_C-F_* = 256.5 Hz), 116.98 (2C), 115.38, 55.34 (2C), 50.08 (2C), 48.28, 46.27, 11.19, 4.42 (2C). ^19^F NMR (376 MHz, CDCl_3_) δ −58.08 (s, 3F). ESI/MS for (C_27_H_29_F_3_N_8_O [M + H]^+^): Calcd: 539.2489. Found: 539.2496.

4-((9-(Cyclopropylmethyl)-2-((4-(4-methylpiperazin-1-yl)phenyl)amino)-9*H*-purin-6-yl)amino)benzonitrile, (**4i**): White solid, yield 49%, mp 171–172 °C, R_f_ = 0.2. ^1^H NMR (400 MHz, CDCl_3_) *δ* 8.07 (s, 1H, NH), 7.87 (d, *J* = 8.7 Hz, 2H, 2CH), 7.72 (s, 1H, CH), 7.54 (d, *J* = 6.0 Hz, 2H, 2CH), 7.52 (d, *J* = 6.2 Hz, 2H, 2CH), 6.98 (s, 1H, NH), 6.95 (d, *J* = 8.9 Hz, 2H, 2CH), 3.95 (d, *J* = 7.2 Hz, 2H, CH_2_), 3.27–3.17 (m, 4H, 2CH_2_), 2.70–2.58 (m, 4H, 2CH_2_), 2.38 (s, 3H, CH_3_), 1.39–1.28 (m, 1H, CH), 0.68 (q, *J* = 5.7 Hz, 2H, CH_2_), 0.46 (q, *J* = 5.0 Hz, 2H, CH_2_). ^13^C NMR (101 MHz, CDCl_3_) *δ* 156.84, 151.98, 151.42, 147.34, 143.48, 138.65, 133.19, 132.77 (2C), 121.64 (2C), 119.48 (2C), 116.94 (2C), 115.68, 105.12, 55.28 (2C), 49.94 (2C), 48.36, 46.21, 11.18, 4.45 (2C). ESI/MS for (C_27_H_29_N_9_ [M + H]^+^): Calcd: 480.2619. Found: 480.2619.

9-(Cyclopropylmethyl)-*N*^2^-(4-(4-methylpiperazin-1-yl)phenyl)-*N*^6^-(4-nitrophenyl)-9*H*-purine-2,6-diamine, (**4j**): Yellow solid, yield 60%, mp 162–163 °C, R_f_ = 0.2. ^1^H NMR (400 MHz, DMSO-*d*_6_) *δ* 10.34 (s, 1H, NH), 9.02 (s, 1H, NH), 8.31 (d, *J* = 9.0 Hz, 2H, 2CH), 8.10 (d, *J* = 9.3 Hz, 2H, 2CH), 8.08 (s, 1H, CH), 7.58 (d, *J* = 8.6 Hz, 2H, 2CH), 6.89 (d, *J* = 9.0 Hz, 2H, 2CH), 3.95 (d, *J* = 7.1 Hz, 2H, CH_2_), 3.13–2.98 (m, 4H, 2CH_2_), 2.46–2.41 (m, 4H, 2CH_2_), 2.19 (s, 3H, CH_3_), 1.37–1.25 (m, 1H), 0.57–0.50 (m, 2H, CH_2_), 0.47–0.42 (m, 2H, CH_2_). ^13^C NMR (101 MHz, DMSO-*d*_6_) *δ* 156.18, 152.01, 150.97, 146.92, 146.11, 140.69, 139.66, 133.11, 124.42 (2C), 120.83 (2C), 119.32 (2C), 115.87 (2C), 115.07, 54.73 (2C), 48.93 (2C), 47.19, 45.75, 11.28, 3.80 (2C). ESI/MS for (C_26_H_29_N_9_O_2_ [M + H]^+^): Calcd: 500.2517. Found: 500.2519.

Ethyl-4-((9-(cyclopropylmethyl)-2-((4-(4-methylpiperazin-1-yl)phenyl)amino)-9*H*-purin-6-yl)amino)benzoate, (**4k**): Green solid, yield 60%, mp 138–139 °C, R_f_ = 0.2. ^1^H NMR (400 MHz, CDCl_3_) δ 8.03 (s, 1H, NH), 7.98 (d, *J* = 8.7 Hz, 2H, 2CH), 7.82 (d, *J* = 8.8 Hz, 2H, 2CH), 7.71 (s, 1H, CH), 7.55 (d, *J* = 8.9 Hz, 2H, 2CH), 7.02 (s, 1H, NH), 6.95 (d, *J* = 9.0 Hz, 2H, 2CH), 4.35 (q, *J* = 7.1 Hz, 2H, CH_2_), 3.94 (d, *J* = 7.2 Hz, 2H, CH_2_), 3.26–3.15 (m, 4H, 2CH_2_), 2.68–2.55 (m, 4H, 2CH_2_), 2.37 (s, 3H, CH_3_), 1.38 (t, *J* = 7.1 Hz, 3H, CH_3_), 1.35–1.26 (m, H, CH), 0.67 (q, *J* = 5.8 Hz, 2H, CH_2_), 0.45 (q, *J* = 5.0 Hz, 2H, CH_2_). ^13^C NMR (101 MHz, CDCl_3_) δ 166.48 (CO), 156.86, 151.73 (2C), 147.11, 143.55, 138.33, 133.05, 130.77 (2C), 124.36, 121.34 (2C), 118.89 (2C), 117.03 (2C), 115.66, 60.76, 55.30 (2C), 50.03 (2C), 48.29, 46.23, 14.51, 11.18, 4.42 (2). ESI/MS for (C_29_H_34_N_8_O_2_ [M + H]^+^): Calcd: 527.2877. Found: 527.2877.

*N*^6^-(4-Chloro-3-fluorophenyl)-9-(cyclopropylmethyl)-*N*^2^-(4-(4-methylpiperazin-1-yl)phenyl)-9*H*-purine-2,6-diamine, (**4l**): Yellow solid, yield 48%, mp 149–150 °C, R_f_ = 0.3. ^1^H NMR (400 MHz, CDCl_3_) *δ* 8.01 (dd, *J* = 11.5, 2.2 Hz, 1H, CH), 8.00 (s, 1H, NH), 7.69 (s, 1H, CH), 7.52 (d, *J* = 8.9 Hz, 2H, 2CH), 7.22 (dd, *J* = 17.9, 9.9 Hz, 1H, CH), 7.17 (dd, *J* = 8.9, 2.2 Hz, 1H, CH), 7.01 (s, 1H, NH), 6.96 (d, *J* = 9.0 Hz, 2H, 2CH), 3.93 (d, *J* = 7.2 Hz, 2H, CH_2_), 3.26–3.14 (m, 4H, 2CH_2_), 2.69–2.54 (m, 4H, 2CH_2_), 2.36 (s, 3H, CH_3_), 1.35–1.27 (m, 1H, CH), 0.67 (q, *J* = 5.9 Hz, 2H, CH_2_), 0.45 (q, *J* = 4.9 Hz, 2H, CH_2_). ^13^C NMR (101 MHz, CDCl_3_) *δ* 158.11 (d, ^1^*J_C-F_* = 246.1 Hz, CF), 156.97, 151.70, 147.31, 139.43 (d, ^3^*J_C-F_* = 10.3 Hz), 138.25, 132.84, 130.22, 121.61 (2C), 117.09 (2C), 115.83 (d, ^4^*J_C-F_* = 3.3 Hz), 115.37, 113.95 (d, ^2^*J_C-F_* = 18.2 Hz), 108.50 (d, ^2^*J_C-F_* = 26.4 Hz), 55.34 (2C), 50.03 (2C), 48.30, 46.27, 11.17, 4.43 (2C). ^19^F NMR (376 MHz, CDCl_3_) *δ* −113.80 (s, 1F). ESI/MS for (C_26_H_28_ClFN_8_ [M + H]^+^): Calcd: 507.2182. Found: 507.2191.

*N*^6^-(3-Chloro-4-fluorophenyl)-9-(cyclopropylmethyl)-*N*^2^-(4-(4-methylpiperazin-1-yl)phenyl)-9*H*-purine-2,6-diamine, (**4m**): Green solid, yield 66%, mp 153–155 °C, R_f_ = 0.3. ^1^H NMR (400 MHz, CDCl_3_) *δ* 8.02 (dd, *J* = 6.6, 2.6 Hz, 1H, CH), 7.99 (s, 1H, CH), 7.93 (s, 1H, NH), 7.69 (s, 1H, CH), 7.51 (d, *J* = 8.9 Hz, 2H, 2CH), 7.39 (ddd, *J* = 8.9, 3.9, 2.9 Hz, 1H, CH), 7.03 (t, *J* = 8.8 Hz, 1H, CH), 7.02 (s, 1H, NH), 6.94 (d, *J* = 9.0 Hz, 1H, CH), 3.93 (d, *J* = 7.2 Hz, 2H, CH_2_), 3.24–3.16 (m, 4H, 2CH_2_), 2.69–2.58 (m, 4H, 2CH_2_), 2.38 (s, 3H, CH_3_), 1.–1.26 (m, 1H, CH), 0.70–0.64 (q, *J* = 5.8 Hz, 2H, CH_2_), 0.44 (q, *J* = 5.0 Hz, 2H, CH_2_). ^13^C NMR (101 MHz, CDCl_3_) *δ* 156.91, 154.08 (d, ^1^*J_C-F_* = 244.3 Hz), 151.92, 151.62, 146.96, 138.18, 135.93 (d, ^4^*J_C-F_* = 3.1 Hz), 133.08, 122.17, 121.24 (2C), 120.88 (d, ^2^*J_C-F_* = 18.4 Hz), 119.74 (d, ^3^*J_C-F_* = 6.6 Hz), 117.20 (2C), 116.43 (d, ^2^*J_C-F_* = 21.9 Hz), 115.21, 55.27 (2C), 49.94 (2C), 48.27, 46.15, 11.18, 4.42 (2C). ^19^F NMR (376 MHz, CDCl_3_) *δ* −122.82 (s, 1F). ESI/MS for (C_26_H_28_ClFN_8_ [M + H]^+^): Calcd: 507.2182. Found: 507.2189.

9-(Cyclopropylmethyl)-*N*^6^-(3,4-dichlorophenyl)-*N*^2^-(4-(4-methylpiperazin-1-yl)phenyl)-9*H*-purine-2,6-diamine, (**4n**): Green solid, yield 49%, mp 132–133 °C, R_f_ = 0.3. ^1^H NMR (400 MHz, CDCl_3_) *δ* 8.08 (d, *J* = 2.5 Hz, 1H, CH), 7.92 (s, 1H, NH), 7.69 (s, 1H, CH), 7.51 (d, *J* = 8.9 Hz, 2H, 2CH), 7.41 (dd, *J* = 8.8, 2.5 Hz, 1H, CH), 7.29 (d, *J* = 8.7 Hz, 1H, CH), 7.02 (s, 1H, NH), 6.96 (d, *J* = 9.0 Hz, 2H, 2CH), 3.93 (d, *J* = 7.2 Hz, 2H, CH_2_), 3.25–3.15 (m, 4H, 2CH_2_), 2.68–2.57 (m, 4H, 2CH_2_), 2.37 (s, 3H, CH_3_), 1.31 (tdd, *J* = 10.3, 7.5, 3.7 Hz, 1H, CH), 0.67 (q, *J* = 5.9 Hz, 2H, CH_2_), 0.45 (q, *J* = 4.9 Hz, 2H, CH_2_). ^13^C NMR (101 MHz, CDCl_3_) *δ* 156.91, 151.73, 147.11, 138.86, 138.31, 132.93, 132.59, 130.29, 125.80, 121.49, 121.38, 119.26, 117.19, 115.39, 55.32, 49.98, 48.29, 46.23, 11.18, 4.44. ESI/MS for (C_26_H_28_Cl_2_N_8_ [M + H]^+^): Calcd: 523.1887. Found: 523.1888.

9-(Cyclopropylmethyl)-*N*^2^-(4-(4-methylpiperazin-1-yl)phenyl)-*N*^6^-(3-nitro-4-(trifluoromethoxy)phenyl)-9*H*-purine-2,6-diamine, (**4o**): Red solid, yield 39%, mp 144–145 °C, R_f_ = 0.2. ^1^H NMR (400 MHz, CDCl_3_) *δ* 8.54 (d, *J* = 2.7 Hz, 1H, CH), 8.50 (s, 1H, NH), 7.81 (dd, *J* = 9.0, 2.7 Hz, 1H, CH), 7.73 (s, 1H, CH), 7.47 (d, *J* = 8.9 Hz, 2H. 2CH), 7.26 (dd, *J* = 8.9, 0.8 Hz, 1H, CH), 7.07 (s, 1H, NH), 6.92 (d, *J* = 9.0 Hz, 2H, 2CH), 3.94 (d, *J* = 7.2 Hz, 2H, CH_2_), 3.24–3.12 (m, 4H, 2CH_2_), 2.64–2.55 (m, 4H, 2CH_2_), 2.36 (s, 3H, CH_3_), 1.31 (dqd, *J* = 15.3, 7.6, 5.0 Hz, 1H, CH), 0.68 (q, *J* = 5.8 Hz, 2H, CH_2_), 0.45 (q, *J* = 5.0 Hz, 2H, CH_2_). ^13^C NMR (101 MHz, CDCl_3_) *δ* 156.92, 152.10, 151.41, 147.50, 143.09, 139.06, 138.75, 135.24 (d, ^3^*J_C-F_* = 2.1 Hz, C-OCF_3_), 132.41, 124.19, 123.80, 121.81 (2C), 120.44 (d, ^1^*J_C-F_* = 260.0 Hz, CF_3_), 116.86 (2C), 116.13, 115.31, 55.32 (2C), 49.84 (2C), 48.38, 46.27, 11.11, 4.44 (2C). ^19^F NMR (376 MHz, CDCl_3_) *δ* −57.95 (s, 3F, CF_3_). ESI/MS for (C_27_H_28_F_3_N_9_O_3_ [M + H]^+^): Calcd: 584.2340. Found: 584.2339.

#### 2.1.4. General Procedures for the Synthesis of Final Compounds **5a**–**5l**

Derivatives **5a**–**5l** were obtained from **3s**–**3ad** using the same procedure described in 2.1.4. The crude product was purified by column chromatography on silica gel, using methanol/dichloromethane (10:90) as the mobile phase to yield the respective products. The Rf values of each compound of this series were calculated using the mentioned mobile phase.

9-Isopentyl-*N*^2^-(4-(4-methylpiperazin-1-yl)phenyl)-*N*^6^-phenyl-9*H*-purine-2,6-diamine, (**5a**): Brown solid, yield 52%, mp 153.1–155.2 °C, R_f_ = 0.4. ^1^H NMR (400 MHz, CDCl_3_) δ 7.74 (d, *J* = 7.7 Hz, 2H, 2CH), 7.68 (s, 1H, NH), 7.58 (d, *J* = 8.8 Hz, 2H, 2CH), 7.57 (s, 1H, CH), 7.32 (t, *J* = 7.9 Hz, 2H, CH), 7.06 (t, *J* = 7.4 Hz, 1H, CH), 6.97 (s, 1H, NH), 6.93 (d, *J* = 9.0 Hz, 2H, 2CH), 4.16–4.06 (m, 2H, CH_2_), 3.24–3.15 (m, 4H, 2CH_2_), 2.68–2.59 (m, 4H, 2CH_2_), 2.38 (s, 3H, CH_3_), 1.80 (dd, *J* = 14.8, 7.0 Hz, 2H, CH_2_), 1.70–1.57 (m, 1H, CH), 1.00 (d, *J* = 6.6 Hz, 6 H, 2CH_3_). ^13^C NMR (101 MHz, CDCl_3_) δ 156.80, 152.29, 151.40, 146.79, 139.17, 138.12, 133.55, 128.95 (2C), 123.13, 120.88 (2C), 120.32 (2C), 117.15 (2C), 115.53, 55.33 (2C), 50.17 (2C), 46.22, 41.99, 38.88, 25.74, 22.48 (2C). ESI/MS for (C_27_H_34_N_8_ [M + H]^+^). Calcd: 471.2979. Found: 471.2979.

*N*^6^-(3-Fluorophenyl)-9-isopentyl-*N*^2^-(4-(4-methylpiperazin-1-yl)phenyl)-9*H*-purine-2,6-diamine, (**5b**): Brown solid, yield 20%, mp 162.1–164.9 °C, R_f_ = 0.5. ^1^H NMR (400 MHz, CDCl_3_) δ 7.97 (s, 1H, NH), 7.85 (d, *J* = 11.5 Hz, 1H, CH), 7.57 (s, 1H, CH), 7.56 (d, *J* = 9.0 Hz, 2H, 2CH), 7.23–7.14 (m, 3H, 2CH, NH), 6.94 (d, *J* = 9.0 Hz, 2H, 2CH), 6.75–6.66 (m, 1H, CH), 4.16–4.02 (m, 2H, CH_2_), 3.24–3.13 (m, 4H, 2CH_2_), 2.67–2.56 (m, 4H, 2CH_2_), 2.37 (s, 3H, CH_3_), 1.78 (dd, *J* = 14.8, 7.0 Hz, 2H, CH_2_), 1.62 (tt, *J* = 13.3, 6.5 Hz, 1H, CH), 0.98 (d, *J* = 6.6 Hz, 6H, 2CH_3_). ^13^C NMR (101 MHz, CDCl_3_) δ 163.12 (d, ^1^*J_C-F_* = 243.5 Hz), 156.84, 151.93, 151.55, 146.97, 140.85 (d, ^3^*J_C-F_* = 11.3 Hz), 138.34, 133.20, 129.77 (d, ^3^*J_C-F_* = 9.6 Hz), 121.21 (2C), 117.13 (2C), 115.43, 115.15 (d, ^4^*J_C-F_* = 2.5 Hz), 109.36 (d, ^2^*J_C-F_* = 21.5 Hz), 107.42 (d, ^2^*J_C-F_* = 26.8 Hz), 55.24 (2C), 50.00 (2C), 46.14, 41.98, 38.79, 25.70, 22.42 (2C). ^19^F NMR (376 MHz, CDCl_3_) δ −111.76 (s, 1F). ESI/MS for (C_27_H_33_FN_8_ [M + H]^+^). Calcd: 489.2885. Found: 489.2896.

*N*^6^-(3,4-Difluorophenyl)-9-isopentyl-*N*^2^-(4-(4-methylpiperazin-1-yl)phenyl)-9*H*-purine-2,6-diamine, (**5c**): Brown solid, yield 31%, mp 151.2–153.7 °C, R_f_ = 0.6. ^1^H NMR (400 MHz, CDCl_3_) δ 7.96 (ddd, *J* = 12.7, 7.2, 2.5 Hz, 1H, CH), 7.67 (s, 1H, NH), 7.54 (s, 1H, CH), 7.51 (d, *J* = 8.9 Hz, 2H, 2CH), 7.11 (d, *J* = 9.0 Hz, 1H, CH), 7.03 (dd, *J* = 18.4, 9.0 Hz, 1H, CH), 6.92 (d, *J* = 9.0 Hz, 2H, 2CH), 6.90 (s, 1H, NH), 4.13–4.04 (m, 2H, CH_2_), 3.19–3.14 (m, 4H, 2CH_2_), 2.63–2.55 (m, 4H, CH_2_), 2.34 (s, 3H, CH_3_), 1.77 (dd, *J* = 14.8, 7.0 Hz, 2H, CH_2_), 1.61 (tt, *J* = 13.3, 6.7 Hz, 1H, CH), 0.97 (d, *J* = 6.6 Hz, 6H, 2CH_3_). ^13^C NMR (101 MHz, CDCl_3_) δ 156.89, 151.86, 151.62, 150.16 (dd, ^1,2^*J_C-F_* = 246.0, 13.2 Hz), 147.21, 146.25 (dd, ^1,2^*J_C-F_* = 243.8, 13.3 Hz), 138.39, 135.83 (dd, ^3,4^*J_C-F_* = 9.0, 2.6 Hz), 133.02, 121.43 (2C), 117.13 (2C), 117.07 (d, ^2^*J*_C-F_ = 17.6 Hz), 116.98, 115.32 (d, ^3^*J_C-F_* = 7.2 Hz), 109.74 (d, ^2^*J_C-F_* = 22.2 Hz), 55.34 (2C), 50.07 (2C), 46.26, 42.05, 38.87, 25.76, 22.48 (2C). ^19^F NMR (376 MHz, CDCl_3_) δ −136.04 (d, *J* = 22.0 Hz, 1F), -144.94 (d, *J* = 22.0 Hz, 1F). ESI/MS for (C_27_H_32_F_2_N_8_ [M + H]^+^). Calcd: 507.2791. Found: 507.2793.

*N*^2^-(4-(4-Methylpiperazin-1-yl)phenyl)-9-pentyl-*N*^6^-phenyl-9*H*-purine-2,6-diamine, (**5d**): Brown solid, yield 22%, mp 138.5–141.3 °C, R_f_ = 0.5. ^1^H NMR (400 MHz, CDCl_3_) δ 7.75 (d, *J* = 7.7 Hz, 2H, 2CH), 7.68 (s, 1H, NH), 7.58 (s, 1H, CH), 7.56 (d, *J* = 3.4 Hz, 2H, 2CH), 7.36–7.29 (m, 2H, 2CH), 7.06 (t, *J* = 7.4 Hz, 1H, CH), 6.94 (s, 1H, NH), 6.92 (d, *J* = 3.9 Hz, 2H, 2CH), 4.09 (t, *J* = 7.2 Hz, 2H, CH_2_), 3.24–3.15 (m, 4H, 2CH_2_), 2.68–2.60 (m, 4H, 2CH_2_), 2.39 (s, 3H, CH_3_), 1.96–1.85 (m, 2H, CH_2_), 1.35 (ddd, *J* = 12.7, 10.3, 5.1 Hz, 4H, 2CH_2_), 0.91 (t, *J* = 7.0 Hz, 3H, CH_3_). ^13^C NMR (101 MHz, CDCl_3_) δ 156.83, 152.29, 151.43, 146.77, 139.17, 138.26, 133.56, 128.96 (2C), 123.14, 120.94 (2C), 120.34 (2C), 117.19 (2C), 115.52, 55.30 (2C), 50.12 (2C), 46.16, 43.72, 29.68, 28.95, 22.31, 14.06. ESI/MS for (C_27_H_34_N_8_ [M + H]^+^). Calcd: 471.2979. Found: 471.2977.

*N*^6^-(3-Fluorophenyl)-*N*^2^-(4-(4-methylpiperazin-1-yl)phenyl)-9-pentyl-9*H*-purine-2,6-diamine, (**5e**): Brown solid, yield 25%, mp 147.1–149.3 °C, R_f_ = 0.6. ^1^H NMR (400 MHz, CDCl_3_) δ 7.89 (s, 1H, NH), 7.88 (d, *J* = 11.3 Hz, 1H, CH), 7.56 (s, 1H, CH), 7.55 (d, *J* = 9.1 Hz, 2H, 2CH), 7.25–7.19 (m, 2H, 2CH), 7.01 (s, 1H, NH), 6.95 (d, *J* = 8.9 Hz, 2H, 2CH), 6.75–6.69 (m, 1H, CH), 4.07 (t, *J* = 7.2 Hz, 2H, CH_2_), 3.23–3.16 (m, 4H, 2CH_2_), 2.65–2.55 (m, 4H, 2CH_2_), 2.36 (s, 3H, CH_3_), 1.97–1.83 (m, 2H, CH_2_), 1.41–1.28 (m, 4H, 2CH_2_), 0.90 (t, *J* = 6.9 Hz, 3H, CH_3_). ^13^C NMR (101 MHz, CDCl_3_) δ 163.18 (d, ^1^*J_C-F_* = 243.6 Hz), 156.90, 151.95, 151.60, 147.11, 140.87 (d, ^3^*J_C-F_* = 11.3 Hz), 138.44, 133.10, 129.84 (d, ^3^*J_C-F_* = 9.6 Hz), 121.35 (2C), 117.14 (2C), 115.48, 115.13 (d, ^4^*J_C-F_* = 2.8 Hz), 109.42 (d, ^2^*J_C-F_* = 21.6 Hz), 107.43 (d, ^2^*J_C-F_* = 26.8 Hz), 55.33 (2C), 50.08 (2C), 46.25, 43.71, 29.64, 28.92, 22.28, 14.04. ^19^F NMR (376 MHz, CDCl_3_) δ −111.72 (s, 1F). ESI/MS for (C_27_H_33_FN_8_ [M + H]^+^). Calcd: 489.2885. Found: 489.2884.

*N*^6^-(3,4-Difluorophenyl)-*N*^2^-(4-(4-methylpiperazin-1-yl)phenyl)-9-pentyl-9*H*-purine-2,6-diamine, (**5f**): Red solid, yield 65%, mp 172.5–174.6 °C, R_f_ = 0.4. ^1^H NMR (400 MHz, CDCl_3_) δ 7.99 (ddd, *J* = 12.8, 7.3, 2.4 Hz, 1H, CH), 7.94 (s, 1H, NH), 7.56 (s, 1H, CH), 7.52 (d, *J* = 8.9 Hz, 2H, 2CH), 7.14 (d, *J* = 8.9 Hz, 1H, CH), 7.04 (dd, *J* = 18.6, 9.0 Hz, 1H, CH), 6.98 (s, 1H, NH), 6.95 (d, *J* = 9.0 Hz, 2H, 2CH), 4.07 (t, *J* = 7.2 Hz, 2H, CH_2_), 3.24–3.14 (m, 4H, 2CH_2_), 2.65–2.57 (m, 4H, 2CH_2_), 2.36 (s, 3H, CH_3_), 1.96–1.84 (m, 2H, CH_2_), 1.45–1.27 (m, 4H, 2CH_2_), 0.90 (t, *J* = 7.0 Hz, 3H, CH_3_). ^13^C NMR (101 MHz, CDCl_3_) δ 156.94, 151.86, 151.62, 150.11 (dd, ^1,2^*J_C-F_* = 245.5, 13.1 Hz), 147.22, 146.19 (dd, ^1,2^*J_C-F_* = 243.3, 13.2 Hz), 138.45, 135.90 (dd, ^3^*J_C-F_* = 9.3, 2.9 Hz), 132.98, 121.50 (2C), 117.10 (2C), 117.01 (d, ^2^*J_C-F_* = 19.6 Hz), 115.31 (dd, ^3,4^*J_C-F_* = 5.4, 2.2 Hz), 109.71 (d, ^2^*J_C-F_* = 22.2 Hz), 55.33 (2C), 50.05 (2C), 46.25, 43.74, 29.65, 28.92, 22.29, 14.04. ^19^F NMR (376 MHz, CDCl_3_) δ -136.13 (d, *J* = 22.0 Hz, 1F), −145.02 (d, *J* = 22.1 Hz, 1F). ESI/MS for (C_27_H_32_F_2_N_8_ [M + H]^+^). Calcd: 507.2791. Found: 507.2788.

9-Hexyl-*N*^2^-(4-(4-methylpiperazin-1-yl)phenyl)-*N*^6^-phenyl-9*H*-purine-2,6-diamine, (**5g**): Brown solid, yield 20%, mp 155.8–160.0 °C, R_f_ = 0.6. ^1^H NMR (400 MHz, CDCl_3_) δ 7.81 (s, 1H, NH), 7.75 (d, *J* = 7.9 Hz, 2H, 2CH), 7.57 (d, *J* = 7.0 Hz, 2H, 2CH), 7.55 (s, 1H, CH), 7.32 (t, *J* = 7.8 Hz, 2H, 2CH), 7.06 (t, *J* = 7.4 Hz, 1H, CH), 6.95 (s, 1H, NH), 6.93 (d, *J* = 9.0 Hz, 2H, 2CH), 4.08 (t, *J* = 7.2 Hz, 2H, CH_2_), 3.22–3.16 (m, 4H, 2CH_2_), 2.66–2.60 (m, 4H, 2CH_2_), 2.38 (s, 3H, CH_3_), 1.90 (m, 2H, CH_2_), 1.43–1.18 (m, 6H, 3CH_2_), 0.88 (t, *J* = 6.9 Hz, 3H, CH_3_). ^13^C NMR (101 MHz, CDCl_3_) δ 156.83, 152.27, 151.39, 146.78, 139.20, 138.18, 133.51, 128.93 (2C), 123.12, 120.93 (2C), 120.38 (2C), 117.15 (2C), 115.39, 55.26 (2C), 50.11 (2C), 46.14, 43.75, 31.38, 29.93, 26.48, 22.61, 14.13. ESI/MS for (C_28_H_36_N_8_ [M + H]^+^). Calcd: 485.3136. Found: 485.3137.

*N*^6^-(3-Fluorophenyl)-9-hexyl-*N*^2^-(4-(4-methylpiperazin-1-yl)phenyl)-9*H*-purine-2,6-diamine, (**5h**): Yellow solid, yield 50%, mp 147.1–149.9 °C, R_f_ = 0.6. ^1^H NMR (400 MHz, CDCl_3_) δ 7.89 (s, 1H, NH), 7.91–7.85 (m, 1H, CH), 7.56 (s, 1H, CH), 7.55 (d, *J* = 9.9 Hz, 2H, 2CH), 7.21 (m, 2H, 2CH), 7.02 (s, 1H, CH), 6.95 (d, *J* = 9.0 Hz, 2H, 2CH), 6.78–6.68 (m, 1H, CH), 4.08 (t, *J* = 7.2 Hz, 2H, CH_2_), 3.24–3.15 (m, 4H, 2CH_2_), 2.65–2.56 (m, 4H, 2CH_2_), 2.37 (s, 3H, CH_3_), 1.88 (m, 2H, CH_2_), 1.38–1.27 (m, 6H, 3CH_2_), 0.88 (t, *J* = 7.0 Hz, 3H, CH_3_). ^13^C NMR (101 MHz, CDCl_3_) δ 163.19 (d, ^1^*J_C-F_* = 243.6 Hz), 156.90, 151.96, 151.60, 147.09, 140.87 (d, ^3^*J_C-F_* = 11.3 Hz), 138.45, 133.13, 129.85 (d, ^3^*J_C-F_* = 9.6 Hz), 121.34 (2C), 117.16 (2C), 115.50, 115.13 (d, ^4^*J_C-F_* = 2.6 Hz), 109.43 (d, ^2^*J_C-F_* = 21.6 Hz), 107.43 (d, ^2^*J_C-F_* = 26.8 Hz), 55.33 (2C), 50.08 (2C), 46.25, 43.74, 31.37, 29.92, 26.48, 22.61, 14.12. ^19^F NMR (376 MHz, CDCl_3_) δ −111.71 (s, 1F). ESI/MS for (C_28_H_35_FN_8_ [M + H]^+^). Calcd: 503.3041. Found: 503.3046.

*N*^6^-(3,4-Difluorophenyl)-9-hexyl-*N*^2^-(4-(4-methylpiperazin-1-yl)phenyl)-9*H*-purine-2,6-diamine, (**5i**): Yellow solid, yield 45%, mp 170.2–174.0 °C, R_f_ = 0.5. ^1^H NMR (400 MHz, CDCl_3_) δ 7.98 (ddd, *J* = 12.8, 7.3, 2.5 Hz, 1H, CH), 7.94 (s, 1H, NH), 7.55 (s, 1H, CH), 7.52 (d, *J* = 8.9 Hz, 2H, 2CH), 7.17–7.10 (m, 1H, CH), 7.03 (q, *J* = 8.8 Hz, 1H, CH), 7.02 (s, 1H, NH), 6.94 (d, *J* = 9.0 Hz, 2H, 2CH), 4.07 (t, *J* = 7.2 Hz, 2H, CH_2_), 3.24–3.18 (m, 4H, 2CH_2_), 2.69–2.49 (m, 4H, 2CH_2_), 2.37 (s, 3H, CH_3_), 1.97–1.78 (m, 2H, CH_2_), 1.40–1.23 (m, 6H, 3CH_2_), 0.88 (t, *J* = 7.0 Hz, 3H, CH_3_). ^13^C NMR (101 MHz, CDCl_3_) δ 156.92, 151.86, 151.61, 150.10 (dd, ^1,2^*J_C-F_* = 245.7, 13.0 Hz), 147.19, 146.18 (dd, ^1,2^*J_C-F_* = 243.6, 13.1 Hz), 138.44, 135.89 (dd, ^3,4^*J_C-F_* = 9.2, 2.9 Hz), 133.00, 121.46 (2C), 117.10 (2C), 117.00 (d, ^2^*J_C-F_* = 20.7 Hz), 115.38, 115.31 (dd, ^3,4^*J_C-F_* = 5.8, 2.2 Hz), 109.71 (d, ^2^*J_C-F_* = 22.2 Hz), 55.32 (2C), 50.03 (2C), 46.24, 43.76, 31.36, 29.92, 26.47, 22.61, 14.11. ^19^F NMR (376 MHz, CDCl_3_) δ -136.14 (d, *J* = 22.0 Hz, 1F), −145.02 (d, *J* = 22.0 Hz, 1F). ESI/MS for (C_28_H_34_F_2_N_8_ [M + H]^+^). Calcd: 521.2947. Found: 521.2943.

*N*^2^-(4-(4-Methylpiperazin-1-yl)phenyl)-9-octyl-*N*^6^-phenyl-9*H*-purine-2,6-diamine, (**5j**): Yellow solid, yield 30%, mp 171.3–173.5 °C, R_f_ = 0.2. ^1^H NMR (400 MHz, CDCl_3_) δ 7.66 (s, 1H, NH), 7.66 (d, *J* = 7.6 Hz, 2H, 2CH), 7.48 (d, *J* = 7.8 Hz, 2H, 2CH), 7.47 (s, 1H, CH), 7.27–7.20 (m, 2H, 2CH), 6.98 (t, *J* = 7.4 Hz, 1H, CH), 6.91 (s, 1H, NH), 6.85 (d, *J* = 9.0 Hz, 2H, 2CH), 3.99 (t, *J* = 7.2 Hz, 2H, CH_2_), 3.18–3.05 (m, 4H, 2CH_2_), 2.59–2.47 (m, 4H, 2CH_2_), 2.28 (s, 3H, CH_3_), 1.93–1.72 (m, 2H, CH_2_), 1.35–1.10 (m, 10H, 5CH_2_), 0.79 (t, *J* = 6.9 Hz, 3H, CH_3_). ^13^C NMR (101 MHz, CDCl_3_) δ 156.82, 152.28, 151.42, 146.83, 139.19, 138.23, 133.47, 128.92 (2C), 123.10, 120.91 (2C), 120.33 (2C), 117.08 (2C), 115.50, 55.35 (2C), 50.18 (2C), 46.26, 43.71, 31.89, 29.99, 29.26, 29.18, 26.83, 22.73, 14.20. ESI/MS for (C_30_H_40_N_8_ [M + H]^+^). Calcd: 513.3449. Found: 513.3444.

*N*^6^-(3-Fluorophenyl)-*N*^2^-(4-(4-methylpiperazin-1-yl)phenyl)-9-octyl-9*H*-purine-2,6-diamine, (**5k**): Brown solid, yield 20%, mp 181.2–183.6 °C, R_f_ = 0.5. ^1^H NMR (400 MHz, CDCl_3_) δ 7.89 (s, 1H, NH), 7.87 (dd, *J* = 11.4, 9.6 Hz, 1H, CH), 7.56 (s, 1H, CH), 7.55 (d, *J* = 9.4 Hz, 2H, 2CH), 7.22 (d, *J* = 1.8 Hz, 2H, 2CH), 7.05 (s, 1H, NH), 6.95 (d, *J* = 8.7 Hz, 2H, 2CH), 6.72 (ddd, *J* = 9.2, 4.8, 2.2 Hz, 1H, CH), 4.07 (t, *J* = 6.5 Hz, 2H, CH_2_), 3.24–3.14 (m, 4H, 2CH_2_), 2.69–2.53 (m, 4H, 2CH_2_), 2.36 (s, 3H, CH_3_), 1.95–1.83 (m, 2H, CH_2_), 1.41–1.19 (m, 10H, 5CH_2_), 0.87 (t, *J* = 6.8 Hz, 3H, CH_3_). ^13^C NMR (101 MHz, CDCl_3_) δ 163.08 (d, ^1^*J_C-F_* = 243.4 Hz), 156.78, 151.84, 151.49, 146.99, 140.76 (dd, ^3^*J_C-F_* = 11.3), 138.34, 132.99, 129.73 (d, ^3^*J_C-F_* = 9.6 Hz), 121.20 (2C), 117.01 (2C), 115.38, 115.03 (d, ^4^*J_C-F_* = 1,0 Hz), 109.31 (d, ^2^*J_C-F_* = 21.6 Hz), 107.32 (d, ^2^*J_C-F_* = 26.7 Hz), 55.24 (2C), 49.98 (2C), 46.16, 43.62, 31.77, 29.85, 29.14, 29.06, 26.71, 22.61, 14.08. ^19^F NMR (376 MHz, CDCl_3_) δ −111.72 (s, 1F). ESI/MS for (C_30_H_39_FN_8_ [M + H]^+^). Calcd: 531.3354. Found: 531.3356.

*N*^6^-(3,4-Difluorophenyl)-*N*^2^-(4-(4-methylpiperazin-1-yl)phenyl)-9-octyl-9*H*-purine-2,6-diamine, (**5l**): Brown solid, yield 52%, mp 192.0–194.1 °C, R_f_ = 0.5. ^1^H NMR (400 MHz, CDCl_3_) δ 7.96 (s, 1H, NH), 7.96 (ddd, *J* = 12.7, 7.2, 2.5 Hz, 1H, CH), 7.54 (s, 1H, CH), 7.51 (d, *J* = 8.9 Hz, 2H, 2CH), 7.11 (d, *J* = 9.0 Hz, 1H, CH), 7.03 (dd, *J* = 18.4, 9.0 Hz, 1H, CH), 6.92 (d, *J* = 9.0 Hz, 2H, 2CH), 6.90 (s, 1H, NH), 4.05 (d, *J* = 6.4 Hz, 2H, CH_2_), 3.18–3.17 (m, 4H, 2CH_2_), 2.61–2.59 (m, 4H, 2CH_2_), 2.35 (s, 3H, CH_3_), 1.88–1.85 (m, 2H, CH_2_), 1.32–1.24 (m, 10H, 5CH_2_), 0.85 (t, *J* = 6.9 Hz, 3H, CH_3_). ^13^C NMR (101 MHz, CDCl_3_) δ 156.89, 151.86, 151.62, 150.16 (dd, ^1,2^*J_C-F_* = 246.0, 13.2 Hz), 147.21, 146.25 (dd, ^1,2^*J_C-F_* = 243.8, 13.3 Hz), 138.39, 135.83 (dd, ^3,4^*J_C-F_* = 9.0, 2.6 Hz), 133.02, 121.43 (2C), 117.13 (2C), 117.07 (d, ^2^*J*_C-F_ = 17.6 Hz), 116.98, 115.32 (d, ^3^*J_C-F_* = 7.2 Hz), 109.74 (d, ^2^*J_C-F_* = 22.2 Hz), 55.34 (2C), 50.07 (2C), 46.26, 42.05, 31.80, 30.08, 29.18, 29.07, 26.70, 22.69, 14.15. ^19^F NMR (376 MHz, CDCl_3_) δ -136.37 (d, *J* = 21.8 Hz, 1F), −144.29 (d, *J* = 21.8 Hz, 1F). ESI/MS for (C_30_H_38_F_2_N_8_ [M + H]^+^). Calcd: 549.3260. Found: 549.3265.

#### 2.1.5. (4-Aminophenyl) (4-Methylpiperazin-1-yl)Methanone, **7**

To a solution of 4-nitrobenzoyl chloride (**6**, 1.00 g, 10.78 mmol), we added 1-methylpiperazine (1.08 g, 10.78 mmol) and triethylamine (4.5 mL, 32 mmol) in DMF (50 mL), and then the mixture was stirred at room temperature for 2 h. Then, the reaction mixture was poured into 400 mL of cold water and filtered, and the solid was dried in the oven at 80 °C for 2 h to give a product as an orange solid with a yield of 99%, mp 123–124 °C. ^1^H NMR (400 MHz, CDCl_3_) δ 8.26 (d, *J* = 8.7 Hz, 2H, 2CH), 7.56 (d, *J* = 8.7 Hz, 2H, 2CH), 3.80 (s, 2H, CH_2_), 3.36 (s, 2H, CH_2_), 2.49 (s, 2H, CH_2_), 2.34 (s, 2H, CH_2_), 2.31 (s, 3H, CH_3_). ^13^C NMR (101 MHz, CDCl_3_) δ 168.03 (CO), 148.49, 142.09, 128.18 (2C), 124.00 (2C), 77.16, 55.26, 54.66, 47.64, 46.09, 42.26. ESI/MS for (C_12_H_15_N_3_O_3_ [M + H]^+^). Calcd: 250.12. Found: 249.8.

The reduction was performed with a solution of the nitro compound (0.60 g, 2.41 mmol) with SnCl_2_ × 2 H_2_O (3.26 g, 14.42 mmol) in ethanol (50 mL), and the mixture was stirred under reflux by 2 h. After completion of the reaction, the reaction mixture was filtered in Celite and concentrated to give the product as a pink solid, yield 98%, mp 135–137 °C. ^1^H NMR (400 MHz, CDCl_3_) δ 7.09 (d, *J* = 8.5 Hz, 2H, 2CH), 6.45 (d, *J* = 8.5 Hz, 2H, 2CH), 4.04 (s, 2H, NH_2_), 3.50 (s, 4H, 2CH_2_), 2.26 (s, 4H, 2CH_2_), 2.16 (s, 3H, CH_3_). ^13^C NMR (101 MHz, CDCl_3_) δ 170.67 (CO), 148.55, 128.98 (2C), 124.04, 113.71 (2C), 54.79 (4C), 45.78. ESI/MS for (C_12_H_17_N_3_O [M + H]^+^). Calcd: 220.1. Found: 220.1.

#### 2.1.6. General Procedures for the Synthesis of Final Compounds **8a**–**8c**

Derivatives **8a**–**8c** (0.52 mmol) were obtained from **3a**–**3c** (0.52 mmol) and **7** (114 mg, 0.52 mmol) using the same procedure described in 2.1.4. The crude product was purified by column chromatography on silica gel, using methanol/dichloromethane (10:90) as the mobile phase to yield the respective products. The Rf values of each compound of this series were calculated using the mentioned mobile phase.

(4-((9-(Cyclopropylmethyl)-6-(phenylamino)-9*H*-purin-2-yl)amino)phenyl)(4-methylpiperazin-1-yl)methanone, (**8a**): Pink solid, yield 54%, mp 164–165 °C, R_f_ = 0.4. ^1^H NMR (400 MHz, CDCl_3_) *δ* 7.89 (s, 1H, NH), 7.74 (s, 1H, CH), 7.72 (d, *J* = 8.6 Hz, 4H, 4CH), 7.38 (d, *J* = 8.6 Hz, 2H, 2CH), 7.34 (dd, *J* = 10.9, 5.2 Hz, 2H, 2CH), 7.32 (s, 1H, NH), 7.09 (t, *J* = 7.4 Hz, 1H, CH), 3.97 (d, *J* = 7.2 Hz, 2H, CH_2_), 3.67 (s, 4H, 2CH_2_), 2.42 (s, 4H, 2CH_2_), 2.32 (s, 3H, CH_3_), 1.33 (ddd, *J* = 7.8, 4.6, 2.9 Hz, 1H, CH), 0.68 (q, *J* = 5.9 Hz, 2H, CH_2_), 0.46 (q, *J* = 4.9 Hz, 2H, CH_2_). ^13^C NMR (101 MHz, CDCl_3_) *δ* 170.63 (C=O), 155.86, 152.39, 151.07, 142.10, 138.83, 138.48, 128.98 (2C), 128.50 (2C), 128.31, 123.61, 120.87 (2C), 118.05 (2C), 115.90, 70.69 (2C), 55.21 (2C), 48.38, 46.18, 11.19, 4.44 (2C). ESI/MS for (C_27_H_30_N_8_O [M + H]^+^): Calcd: 483.2615. Found: 483.2621.

(4-((9-(Cyclopropylmethyl)-6-((3-fluorophenyl)amino)-9*H*-purin-2-yl)amino)phenyl)(4-methylpiperazin-1-yl)methanone, (**8b**): Yellow solid, yield 55%, mp 150–151 °C, R_f_ = 0.3. ^1^H NMR (400 MHz, CDCl_3_) *δ* 8.04 (s, 1H, NH), 7.86 (d, *J* = 12.0 Hz, 1H, CH), 7.75 (s, 1H, CH), 7.71 (d, *J* = 8.5 Hz, 2H, 2CH), 7.41 (d, *J* = 8.5 Hz, 2H, 2CH), 7.40 (s, 1H, NH), 7.23 (m, 2H, 2CH), 6.77–6.72 (m, 1H, CH), 3.97 (d, *J* = 7.2 Hz, 2H, CH_2_), 3.67 (s, 4H, 2CH_2_), 2.42 (s, 4H, 2CH_2_), 2.32 (s, 3H, CH_3_), 1.32 (ddd, *J* = 15.5, 7.6, 4.9 Hz, 1H, CH), 0.68 (q, *J* = 5.5 Hz, 2H, CH_2_), 0.45 (q, *J* = 5.1 Hz, 2H, CH_2_). ^13^C NMR (101 MHz, CDCl_3_) *δ* 170.57 (C=O), 163.13 (d, ^1^*J_C-F_* = 243.6 Hz, CF), 155.81, 152.00, 151.24, 141.88, 140.58 (d, ^3^*J_C-F_* = 11.2 Hz), 138.72, 129.94 (d, ^3^*J_C-F_* = 9.5 Hz, CH), 128.63, 128.56 (2C), 118.31 (2C), 115.91, 115.55 (d, ^4^*J_C-F_* = 2.1 Hz, CH), 109.80 (d, ^2^*J* = 21.9 Hz, CH), 107.77 (d, ^2^*J_C-F_* = 26.6 Hz, CH), 70.55 (2C), 55.22 (2C), 48.41, 46.18, 11.15, 4.44 (2C). ^19^F NMR (376 MHz, CDCl_3_) *δ* −111.89 (s, 1F). ESI/MS for (C_27_H_29_FN8O [M + H]^+^): Calcd: 501.2125. Found: 501.2527.

(4-((9-(Cyclopropylmethyl)-6-((3,4-difluorophenyl)amino)-9*H*-purin-2-yl)amino)phenyl)(4-methylpiperazin-1-yl)methanone, (**8c**): Brown solid, yield 54%, mp 142–143 °C, R_f_ = 0.3. ^1^H NMR (400 MHz, CDCl_3_) *δ* 8.07 (s, 1H, NH), 7.95 (ddd, *J* = 12.6, 7.1, 2.5 Hz, 1H, CH), 7.75 (s, 1H, CH), 7.70 (d, *J* = 8.6 Hz, 2H, 2CH), 7.40 (d, *J* = 8.6 Hz, 2H, 2CH), 7.38 (s, 1H, NH), 7.15 (d, *J* = 8.7 Hz, 1H, CH), 7.11–6.98 (m, 1H, CH), 3.97 (d, *J* = 7.2 Hz, 2H, CH_2_), 3.69 (s, 4H, 2CH_2_), 2.44 (s, 4H, 2CH_2_), 2.33 (s, 3H, CH_3_), 1.32 (dtd, *J* = 14.9, 7.4, 2.5 Hz, 1H, CH), 0.72–0.65 (m, 2H, CH_2_), 0.46 (q, *J* = 5.0 Hz, 2H, CH_2_). ^13^C NMR (101 MHz, CDCl_3_) *δ* 170.54 (C=O), 155.79, 151.94, 151.25, 148.70 (dd, ^1^*J_C-F_* = 234.5, ^2^*J_C-F_* = 13.3 Hz, CF), 145.93 (dd, ^1,2^*J_C-F_* = 244.4, 19.9 Hz, CF), 141.85, 138.75, 135.54 (dd, ^3,4^*J* = 9.0, 2.8 Hz), 128.66, 128.55 (2C), 118.31 (2C), 117.11 (d, ^2^*J_C-F_* = 18.7 Hz, CH), 115.93 (dd, ^3,4^*J_C-F_* = 5.1, 2.9 Hz, CH), 115.71, 110.14 (d, ^2^*J_C-F_* = 21.8 Hz, CH), 70.47 (2C), 55.17 (2C), 48.43, 46.12, 11.16, 4.45 (2C). ^19^F NMR (376 MHz, CDCl_3_) *δ* -136.26 (d, ^3^*J_F-F_* = 21.9 Hz, 1F), −144.31 (d, ^3^*J_F-F_* = 21.9 Hz, 1F). ESI/MS for (C_27_H_28_F_2_N_8_O [M + H]^+^): Calcd: 519.2427. Found: 519.2435.

#### 2.1.7. Synthesis of 2,4-Dichloro-7-(Cyclopropylmethyl)-7*H*-Pyrrolo[2,3-*d*]Pyrimidine, **10**

To a solution of 2,4-dichloro-7*H*-pyrrolo[2,3-*d*]pyrimidine (**9**, 1.0 g, 5.3 mmol), (bromomethyl)cyclopropane (1.08 g, 7.9 mmol) and K_2_CO_3_ (2.25 g, 15.9 mmol) in DMF (20 mL) were added, then the mixture was stirred at room temperature by 12 h. The mixture was filtrated and evaporated. The residue was purified by silica gel chromatography using DCM as mobile phase to give a white solid, yield 82%, mp 41.2–44.7 °C, Rf = 0.5 (DCM). ^1^H NMR (400 MHz, CDCl_3_) δ 7.35 (d, *J* = 3.6 Hz, 1H, CH), 6.58 (d, *J* = 3.6 Hz, 1H, CH), 4.07 (d, *J* = 7.2 Hz, 2H, CH_2_), 1.35–1.15 (m, 1H, CH), 0.63 (qd, *J* = 6.0, 4.9 Hz, 2H, CH_2_), 0.42 (q, *J* = 4.9 Hz, 2H, CH_2_). ^13^C NMR (101 MHz, CDCl_3_) δ 152.54, 151.98, 151.78, 129.65, 116.43, 99.93, 49.82, 11.25, 4.24 (2C). ESI/MS for (C_10_H_9_Cl_2_N_3_ [M + H]^+^). Calcd: 242.0. Found: 241.9.

#### 2.1.8. 2-Chloro-7-(Cyclopropylmethyl)-*N*-Phenyl-7*H*-Pyrrolo[2,3-*d*]Pyrimidin-4-Amine, **11**

**11** was obtained starting from **10** (500 mg, 2.07 mmol) and aniline (193 mg, 2.07 mmol) using the same procedure described in 2.1.4. The crude product was purified by column chromatography on silica gel, using methanol/dichloromethane (R_f_ value was calculated using the mentioned mobile phase), to yield a yellow solid (88%), mp 81.2–84.9 °C, Rf = 0.4. ^1^H NMR (400 MHz, CDCl_3_) δ 7.52 (d, *J* = 7.7 Hz, 2H, 2CH), 7.40 (t, *J* = 7.9 Hz, 2H, 2CH), 7.25–7.20 (m, 1H, CH), 7.21 (s, 1H, NH), 6.98 (d, *J* = 3.6 Hz, 1H, CH), 5.88 (d, *J* = 3.6 Hz, 1H, CH), 4.00 (d, *J* = 7.1 Hz, 2H, CH_2_), 1.25–1.13 (m, 1H, CH), 0.59 (q, *J* = 5.9 Hz, 2H, CH_2_), 0.38 (q, *J* = 4.9 Hz, 2H, CH_2_). ^13^C NMR (101 MHz, CDCl_3_) δ 155.39, 153.09, 151.85, 138.31, 129.29 (2C), 125.57, 124.62, 123.78 (2C), 101.86, 99.21, 49.23, 11.38, 4.11 (2C). ESI/MS for (C_16_H_15_ClN4 [M + H]^+^). Calcd: 299.11. Found: 299.0.

#### 2.1.9. 7-(Cyclopropylmethyl)-N^2^-(4-(4-Methylpiperazin-1-yl)Phenyl)-N^4^-Phenyl-7*H*-pyrrolo[2,3-*d*]Pyrimidine-2,4-Diamine, **12**

**12** was obtained starting from **11** (100 mg, 0.34 mmol) and 4-(4-methylpiperazin-1-yl)aniline (59 mg, 0.34 mmol) using the same procedure described in 2.1.4. The crude product was purified by column chromatography on silica gel, using methanol/dichloromethane (10:90) as mobile phase to yield a yellow solid, yield 43%, mp 102.3–105.5 °C, R_f_ = 0.4. ^1^H NMR (400 MHz, CDCl_3_) δ 7.60 (d, *J* = 7.2 Hz, 2H, 2CH), 7.58 (d, *J* = 8.8 Hz, 2H, 2CH), 7.34 (t, *J* = 7.9 Hz, 2H, 2CH), 7.11 (t, *J* = 7.4 Hz, 1H), 6.92 (d, *J* = 9.0 Hz, 2H, 2CH), 6.83 (d, *J* = 3.6 Hz, 1H, CH), 6.80 (s, 1H, NH), 6.09 (d, *J* = 3.6 Hz, 1H), 3.95 (d, *J* = 7.0 Hz, 2H, CH_2_), 3.19–3.17 (m, 4H, 2CH_2_), 2.64–2.61 (m, 4H, 2CH_2_), 2.38 (s, 3H, CH_3_), 1.30–1.22 (m, 1H), 0.60 (q, *J* = 5.9 Hz, 2H, CH_2_), 0.41 (q, *J* = 4.8 Hz, 2H, CH_2_). ^13^C NMR (101 MHz, CDCl_3_) δ 156.20, 154.45, 152.53, 146.28, 139.43, 134.18, 128.98 (2C), 123.71, 121.97 (2C), 121.95, 120.31 (2C), 117.30 (2C), 98.28, 97.89, 55.34 (2C), 50.26 (2C), 48.85, 46.19, 11.46, 4.07 (2C). ESI/MS for (C_27_H_31_N_7_ [M + H]^+^). Calcd: 454.2714. Found: 454.2711.

### 2.2. Kinase Assays

Recombinant kinase inhibition was measured using standard radioassay. Bcr-Abl1 and Cdk-2/cyclin E kinases were produced in-house, FLT3-ITD and BTK were purchased from ProQinase. The kinases were assayed with suitable substrates (500 µM peptide GGEAIYAAPFKK for Abl, 1 mg/mL histone H1 for Cdk-2, 100 µM poly (Glu:Tyr; 4:1)) in the presence of ATP (1 µM for Bcr-Abl, 15 µM for Cdk-2 and BTK), 0.05 µCi [γ-^33^P]ATP and the test compound in reaction buffer (60 mM HEPES-NaOH, pH 7.5, 3 mM MgCl_2_, 3 mM MnCl_2_, 3 µM Na orthovanadate, 1.2 mM DTT, 2.5 µg/50 µL PEG20.000). The reactions were stopped by adding 3% aq. H_3_PO_4_. P-81 phosphocellulose (Whatman) was used to separate phosphorylated substrates. Kinase activity was quantified using a FLA-7000 digital image analyser. The concentration of the test compounds required to reduce the activity by 50% (IC_50_ value) was determined from dose–response curves.

### 2.3. 3D-QSAR

#### 2.3.1. Data Processing

CoMFA studies were performed on the whole set of 31 purine derivatives (Table S3). The biological activity IC_50_ was converted to pIC_50_ values (-log IC_50_, in molar concentration). Compounds were divided into training and test sets in an 80:20 ratio, respectively. In all cases, the biological pIC_50_ range was between 2 and 3 log units. CoMFA studies were achieved with Sybyl X-1.2 software installed in Windows 10 environment on a PC with an Intel Core i7 CPU. 

**Figure 4:**
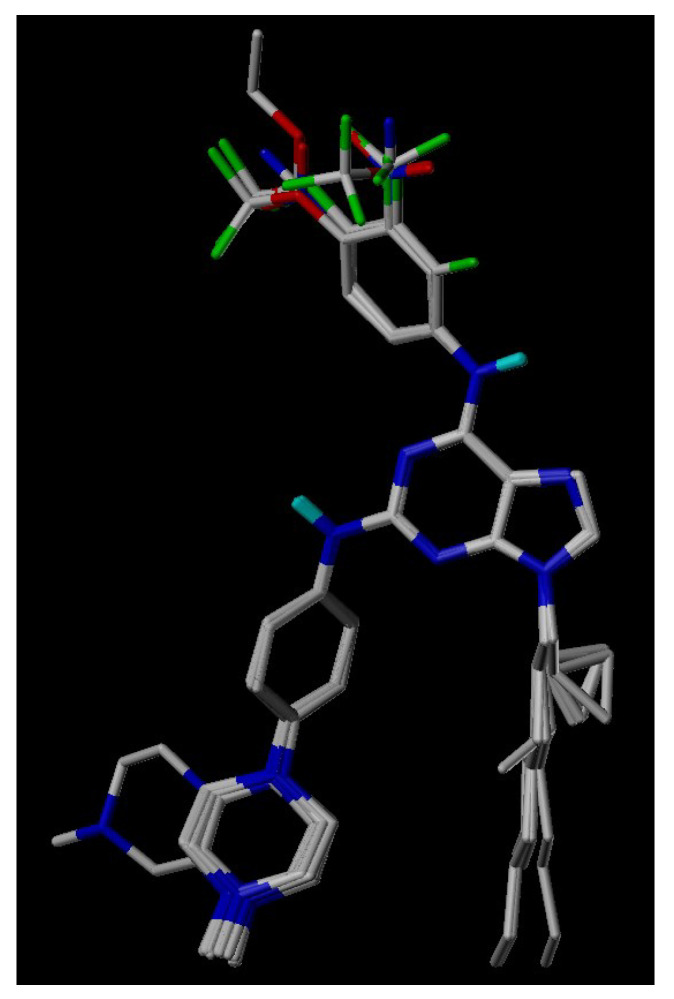
Superimposed structures of all compounds used in CoMFA models.

To obtain the best conformers for each purine, these were drawn in ChemDraw and exposed to a preliminary geometry optimization using MM2 molecular mechanics which was implemented in ChemBio3D software. Following this, compounds were minimized by the Powell method as implemented in Sybyl (1000 iterations and 0.005 Kcal/molÅ as termination gradient). Then, MMFF94 charges were assigned to each atom in every molecule. The minimized structures were superimposed with the Distill protocol (Figure 4).

#### 2.3.2. CoMFA Field Calculation

After the minimization of the molecules, their steric and electrostatic potentials were calculated for each one, within a virtual lattice of 2 Å separation. For this purpose, a probe atom was used (a *sp*^3^ carbon probe atom with a van der Waals radius of 1.52 Å and a charge of +1.0). 30.0 kcal/mol was the limit value for the visualization of both steric and electrostatic fields.

#### 2.3.3. Internal Validation and Partial Least Squares (PLS) Analysis

The statistical method of partial least squares (PLS) was used to search for correlations between the CoMFA variables (steric and electrostatic potentials) and the biological activity dependent variables). [50]. To find the best model, the cross-validation analysis was executing through Leave-One-Out (LOO) and sample distance PLS [SAMPLS]). From this analysis, *q*^2^ and *N* (optimal number of components) were obtained for each model. The value of *q*^2^ must be greater than 0.5 and is an indicator of the internal predictive capacity of the model. It is calculated from the following Equation (1):

(1) $$q^{2}=1-\frac{\sum {\left(y_{i}-y_{pred}\right)}^{2}}{\sum {\left(y_{i}-\bar{y}\right)}^{2}}$$

where $y_{i}$, $\bar{y}$ and $y_{pred}$ are the observed, mean and predicted activities in the training set, respectively.

#### 2.3.4. External Validation

The models were subjected to additional validation through the evaluation of the activity prediction for the test set. The method described by Golbraik [51,52] was used, which proposes that a model is adequate if it meets the following criteria:

(2) $$q^{2}>0.5$$

(3) $$r^{r}>0.6$$

(4) $$0.85\leq k\leq 1.15\;\mathrm{or}\;0.85\leq \;k^{\prime }\leq 1.15$$

Compliance with these values is essential to guarantee adequate external predictive capacity of the models [51].

### 2.4. Docking

Molecular modelling studies were performed using Schrodinger Suite 2021-1. All minimizations were performed utilizing the OPLS4 force field [53]. The X-ray crystal structures of Bcr-Abl (PDB ID: 2GQG; resolution: 2.40 Å) [54], BTK (PDB ID: 4OT5; resolution: 1.55 Å) [55] and FLT3 (PDB ID: 6JQR; resolution: 2.20 Å) [56] co-crystallized with their respective inhibitors were downloaded from the protein database (https://www.rcsb.org/ (accessed on 5 March 2021)). First, the water molecules and ligands that were not involved in binding to the active site were removed. Then, the enzyme structure was prepared using the Protein Preparation module, where the missing amino acids were added using the Prime program, polar amino acids were protonated at pH 7.0, and protein hydrogens were minimized [57,58].

The co-crystallized ligands were used to prepare the binding site grid for molecular docking. First, the “Standard Precision” docking method (SP) was used to generate 20 poses per ligand. Then, the best poses were refined with the “eXtra Precision” (XP) method of the Glide program [59,60]. The docking method was first validated by self-docking the co-crystallized ligand to the active site of the enzyme. This resulted in a docking pose with an RMSD less than 0.5 Å from the co-crystallized ligand pose (see Figure S2). The validated molecular docking protocol was then used to study the ligand-protein interactions for compounds **4f**, **5b** and **5j** at the enzyme active site and predict their binding pattern (For more details, see Supplementary Materials).

### 2.5. Cytotoxicity Assays

Cytotoxicity assays for the purines were quantified using the resazurin method, a dye to measure redox reactions occurring in the mitochondria of live cells. The cells were treated by tested compounds (in triplicate, six different doses) for 72 h. After this period, a resazurin (Sigma Aldrich, St. Louis, MO, USA) solution was added, and the fluorescence of resorufin corresponding to the live cell quantity was measured after 4 h at 544 nm/590 nm (excitation/emission) using a Fluoroskan Ascent microplate reader (Labsystems Oy, Vantaa, Finland). The drug concentration lethal to 50% of the cells (GI_50_) was calculated from the dose–response curves.

### 2.6. Flow Cytometry

Cell cycle effects were measured using flow cytometric determination of DNA content. MV4-11 and K562 cells were treated with tested compounds for 24 h. After the staining with propidium iodide, the DNA content was analysed by flow cytometry using a 488 nm laser (BD FACS Verse with software BD FACSuite^TM^, version 1.0.6.). The cell cycle distribution was analysed using ModFit LT (Verity Software House 5.0, Topsham, ME, USA).

### 2.7. Immunoblotting

Protein changes in the treated cells were analysed using immunoblotting. Briefly, cell lysates were separated on SDS-polyacrylamide gels and electroblotted onto nitrocellulose membranes. After blocking, overnight incubation with specific primary antibodies and incubation with peroxidase-conjugated secondary antibodies, the peroxidase activity was detected with Super-Signal West Pico reagents (Thermo Scientific, Waltham, MA, USA) using a CCD camera LAS-4000 (Fujifilm). The following specific antibodies were purchased from Cell Signalling: anti-FLT3 (clone 8F2), anti-phospho-FLT3 Y589/591 (clone 30D4), anti-STAT5 (clone D2O6Y), anti-phospho-STAT5 Y694, anti-ERK1/2, anti-phospho-ERK1/2 T202/Y204, anti-Akt (clone C67E7), anti-phospho-Akt S473 (clone D9E), anti-phospho-Bcr Y177, anti-CrkL (clone 32H4), anti-phospho-CrkL Y207, anti-BTK (clone C82B8), anti-phospho-BTK Y223, anti-PLCγ2, anti-phospho-PLCγ2 Y1217, and anti-PARP-1 (clone 46D11). Anti-α-tubulin (clone DM1A) was purchased from Sigma Aldrich.

### 2.8. Caspase-3/7 Assay

MV4-11 and K562 cells were kept in a 96-well plate were treated with increasing concentrations of compound **4i** and **5b** for 24 h. After this period, caspase-3/7 assay buffer (150 mM HEPES pH 7.4, 450 mM NaCl, 150 mM KCl, 30 mM MgCl_2_, 1.2 mM EGTA, 1.5% Nonidet P40, 0.3% CHAPS, 30% sucrose, 30 mM DTT, and 3 mM PMSF) containing 150 µM peptide substrate Ac-DEVD-AMC (Enzo Life Sciences, Farmingdale, NY, USA) was added, and after 2 h incubation, the fluorescence of AMC was measured using a Fluoroskan Ascent microplate reader (Labsystems) at 346 nm/442 nm (excitation/emission).

## 3. Results and Discussion

### 3.1. Chemistry

**Scheme 1:**
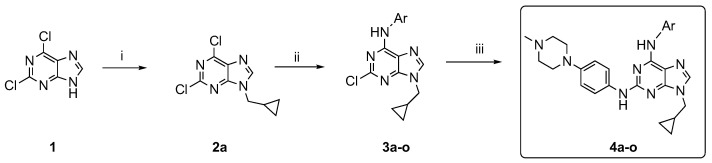
Synthesis of purine derivatives **4a**–**o.** Reagents and conditions: (i) (Bromomethyl)cyclopropane, K_2_CO_3_, DMF, 12 h, rt, 69%. (ii) Anilines, DIPEA, *n*-butanol, reflux, 12 h, 18–86% (iii) 4-(4-methylpiperazin-1-yl)aniline, 2 M K_2_CO_3_, Pd(OAc)_2,_ Xantphos, dioxane, MW, 100 °C, 2 h, 29–66%.

This work uses the simple and efficient synthesis methods reported by our group. We present here the synthesis of new 2,6,9-trisubstituted purines **4a**–**o**, **5a**–**l** and **9a**–**c** as described in Scheme 1 [44,45,46]. A total of 30 compounds were obtained using 2,6-dichloropurine (**1**) as a starting material. To achieve those derivatives modified in the aniline fragment on C-6, the first step was the alkylation of **1** with (bromomethyl)cyclopropane under basic conditions to give a mixture of N-9 and N-7 alkylated purine regioisomers **2a**–**2a’** in a 3:1 ratio in 61% yield for both compounds. Later, we used a regioselective nucleophilic substitution (S_N_Ar) at position C-6 of **2a** with anilines using *n*-butanol as the solvent and *N*,*N*-diisopropylethylamine as the base. The **3a**–**o** was obtained in various yields (18–86%) at 110 °C for 12 h. Finally, a Buchwald–Hartwig coupling reaction at C-2 of **3a**–**o** with 4-(4-methylpiperazin-1-yl)aniline was synthesized according to the literature [44], promoted by microwave and catalysed by palladium (II) yielded the purine derivatives **4a**–**o** in low to moderate yields (39–66%). The chemical structures of **4a**–**o** are shown in Table 1.

**Scheme 2:**
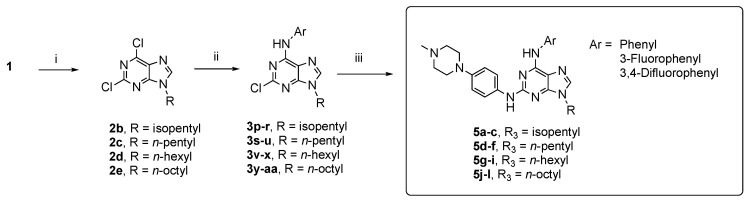
Synthesis of purine derivatives **5a**–**l**. Reagents and conditions*:* (i) Alkyl halides, K_2_CO_3_, DMF, 12 h, rt, 64–98% (ii) Anilines, DIPEA, *n*-butanol, reflux, 12 h, 40–83% (iii) 4-(4-methylpiperazin-1-yl)aniline, 2 M K_2_CO_3_, Pd(OAc)_2,_ Xantphos, dioxane, MW, 100 °C, 2 h, 20–65%.

For those purine derivatives with several hydrophobic alkyl groups in N-9 **5a**–**l**, we performed the same procedure described for **4a**–**o** (Scheme 2). **1** was alkylated with the respective alkyl halides and the yielded compounds **2b**–**e** (64–98%), which were substituted by aniline, 3-fluoraniline or 3,4-difluoraniline (to compare these purine derivatives with **III**, **IV** and **V**) to obtain **3p**–**aa** (40–83%). The final step was the microwave-assisted Buchwald–Hartwig coupling of 4-(4-methylpiperazin-1-yl)aniline with the **3p**–**aa** derivatives, thus, yielding the target compounds **5a**–**l** (20–65%).

**Scheme 3:**
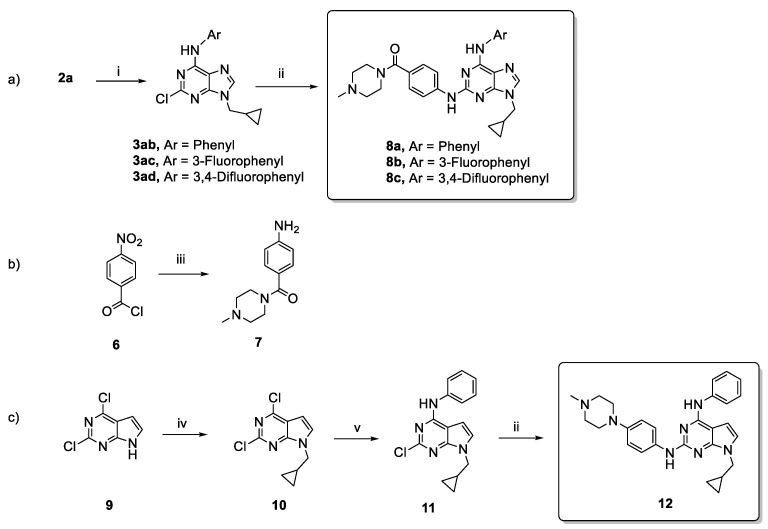
Synthesis of purine derivatives **8a**–**c** in the steps (**a**,**b**), and **12** in (**c**). Reagents and conditions: (i) Anilines, DIPEA, *n*-butanol, reflux, 12 h, 71–86%, (ii) **7** or 4-(4-methylpiperazin-1-yl)aniline, 2 M K_2_CO_3_, Pd(OAc)_2,_ Xantphos, dioxane, MW, 100 °C, 2 h, 43–55%, (iii) 1. *N*-methylpiperazine, K_2_CO_3_, DMF, 2 h, 99% 2. Hydrazine, Pd-C, ethanol, 70 °C, 2 h, 98%, (iv) (bromomethyl)cyclopropane, K_2_CO_3_, DMF, 12 h, rt, 82% and (v) aniline, 2 M K_2_CO_3_, Pd(OAc)_2,_ Xantphos, dioxane, MW, 100 °C, 1 h, 88%.

The synthesis of 2,6,9-trisubstituted purines that present a carbonyl group between *N*-piperazine and aniline moiety (Scheme 3a,b) was performed using the strategy above. Therefore, we started from **2a** and the respective S_N_Ar by aniline and then added the 3-fluoraniline or 3,4-difluoraniline and used **7** for the last step; compounds **8a**–**c** were thus obtained. **7** was synthesized from *p*-nitrobenzoyl chloride **6** as shown in Scheme 3. Finally, we synthesized a pyrrolo-pyrimidine bioisoster **12** of **V**. Scheme 3c shows that the synthetic strategy to obtain **12** considered the same three steps to yield the previous target compounds; however, 2,6-dichloro-pyrrolo[2,3-*d*]pyrimidine **9** was the starting material.

All synthesised compounds were purified by column chromatography, and their structures were established based on their spectral properties (^1^H NMR, ^13^C NMR, ^19^F NMR, MS and HRMS, see Experimental Section and Supplementary Materials for target compounds).

### 3.2. Kinase Inhibition

We screened all synthesized compounds for inhibitory activity against Abl, BTK and FLT3-ITD (Table 1). The serine/threonine kinase Cdk-2 was assayed in parallel to study the selectivity of tyrosine kinases.

Table 1 shows some interesting findings:

1. The Abl kinase was the most sensitive kinase to inhibition by several compounds at sub-micromolar concentrations (<1.0 μM, 48%) in contrast with BTK (42%) and FLT3-ITD (29%). In our previous work, most purine derivatives had low activity on Cdk-2/E, which may suggest selectivity towards these tyrosine kinases.
2. Although Abl did not find a more potent inhibitor than our hit compounds (**IV** and **V**), **4k** was the most active compound in the series (IC_50_ = 40 nM, similar to **IV** and **V**). For BTK some compounds with slightly better activity than **V** were recognized (with IC_50_ values < 0.58 μM: **5d**, **5e**, **5i** and **5j**), thus, highlighting **5h** (IC_50_ = 0.32 μM). Likewise, the purine derivatives could also be a starting point to develop FLT3-ITD inhibitors considering **5a** (IC_50_ = 0.23 μM).
3. Preliminary evidence about the selectivity of 2,6,9-trisubstituted purines to these kinases is revealed and illustrates the length of the alkyl chain on N-9 and the pattern of substitution on the aniline on C-6. However, in silico studies were conducted to explore this hypothesis in detail. The results are discussed in Section 3.3 and Section 3.4. This explains the selectivity among these kinases by purine derivatives.

A structure–activity relationship (SAR) analysis can be established for each target as follows:

1. The substitution pattern on the 6-phenylamino ring was found to significantly influence the inhibitory potency in these kinases especially those related to the size and position of the substituents. For Abl, the size of the substituents in the meta-position appeared to be more important than their electronic features in terms of activity. This effect led us to explain the results for **4f** versus **IV** or **4c** vs. **4d** (CH_3_ instead of CF_3_). In addition, the least active compounds (**4d** and **4e**) had the bulkiest substituents. Similar behaviour was observed for BTK and FLT3-ITD. A comparison of the substitution at the meta- vs. para- position for Abl indicates that the second one is more beneficial for the inhibition of this kinase. Nevertheless, the tendency for BTK or FLT3-ITD is clearer than Abl; the presence of substituents in the para increased the potency as shown in compounds **4g** vs. **4j** (NO_2_), **4f** vs. **4i** (CN) or **4e** vs. **4h** (OCF_3_). As expected, the presence of fluorine at the ortho-position diminished the inhibitory activity on Abl and BTK. In addition, any substituent in the 3,4-disubstituted derivatives replacing one or both fluorine atoms of **V** reduce the potency in three kinases: This was mainly observed when fluorine was simultaneously replaced by NO_2_ and OCF_3_ groups (**4o**).
2. The length and volume of the alkyl chain on N-9 dramatically influenced the inhibitory effect on kinases. A bulky (isopentyl, especially **5b**) or a linear alkyl chain longer than five carbons decreased the potency of Abl (for **5h**, **5j** and **5k** IC_50_ values = 100 μM). For BTK, a linear chain of five or six carbons interestingly increased the activity (depending on the aminophenyl moiety on C-6, it can be n-pentyl or n-hexyl, **5d**, **5e**, **5h** or **5i**). However, the best results were observed for purine derivatives with an isopentyl group for FLT3-ITD (**5a**, **5b** or **5c**). This is a very important finding that illustrates the selectivity of these three kinases.
3. The addition of CO between piperazine and aniline ring (C-2 purine) reduced the potency of Abl, BTK and FLT3-ITD.
4. Finally, as expected, the replacement of N-7 by CH, significantly reduced the potency, which underscores the importance of this atom.

### 3.3. D-Quantitative Structure–Activity Relationship (QSAR)

We next analysed the 3D-QSARs for each kinase to characterize the SAR information of the different targets observed previously and validate our design and propose a rational series of structural modifications that give rise to a new family of compounds. Here, the biological activity (IC_50_) was correlated with the steric and electrostatic potentials of these purines. The results can be visualized through coloured polyhedra around some fragments of these compounds; these indicate that changes in volume and electronic properties should be performed to improve biological activity. We next presented the results obtained for each kinase.

**Figure 5:**
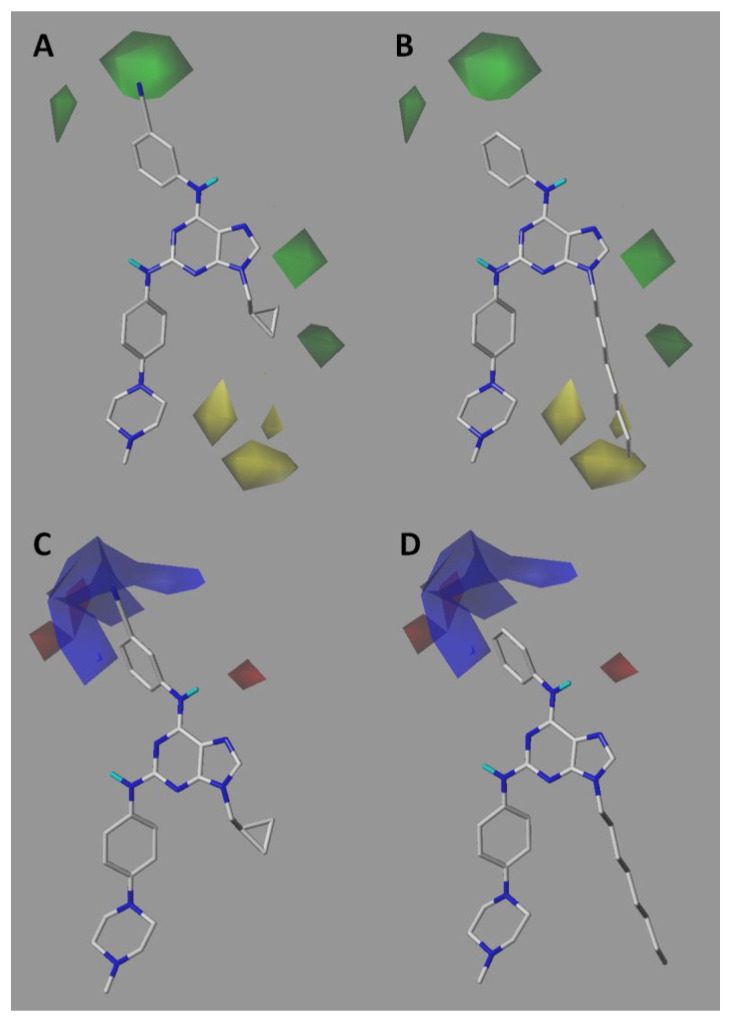
Steric (**A**,**B**) and electrostatic (**C**,**D**) contour maps in Bcr-Abl. The most active compound **4f** (**A**,**C**) is compared against the least active **5j** (**B**,**D**).

For Bcr-Abl, a steric contour map (Figure 5A,B) displays two green polyhedra on the *meta-* and *para*-positions of the aminophenyl fragment linked on C-6 of the purine ring. This means that the insertion of bulky groups at those positions is favourable for the inhibitory activity on Bcr-Abl as observed for **4k** (IC_50_ = 40 nM). The yellow polyhedra show that small groups are favourable.

This behaviour is observed for the most active purine derivative (**4f**, IC_50_ = 70 nM); it has a nitrile group in the *meta*-position of aminophenyl fragment immersed in the green polyhedron. The others have active compounds (**4c** IC_50_ = 0.10, **4b** IC_50_ = 0.12 and **4i** IC_50_ = 0.10 mM). The *ortho*-position does not seem to be favourable for the insertion of substituents perhaps due to the deleterious steric effect on the NH group of aminophenyl ring, which could prevent a correct interaction with the Met318 as shown by our docking studies. Two green polyhedra are also seen around the cyclopropyl ring. This means that the presence of short and bulky alkyl chains on the N-9 of the purine is favourable for biological activity. 

However, there are three yellow polyhedra beyond the length of the cyclopropyl group, which means that the increase in chain length on N-9 is not favourable for activity. In the electrostatic potential map (Figure 5C,D), the red polyhedra means that the presence of electron-rich atoms is favourable for activity. The blue colour indicates that the presence of electron-deficient groups is favourable for activity. A blue polyhedron can be seen for **4f** near the *meta-* and *para*-positions of the aminophenyl fragment on C-6. In **5j**, the lack of a substituent could be related to the low activity. This blue polyhedron means that the electron-withdrawing functional groups in the ring underlie its activity (**4k**).

**Figure 6:**
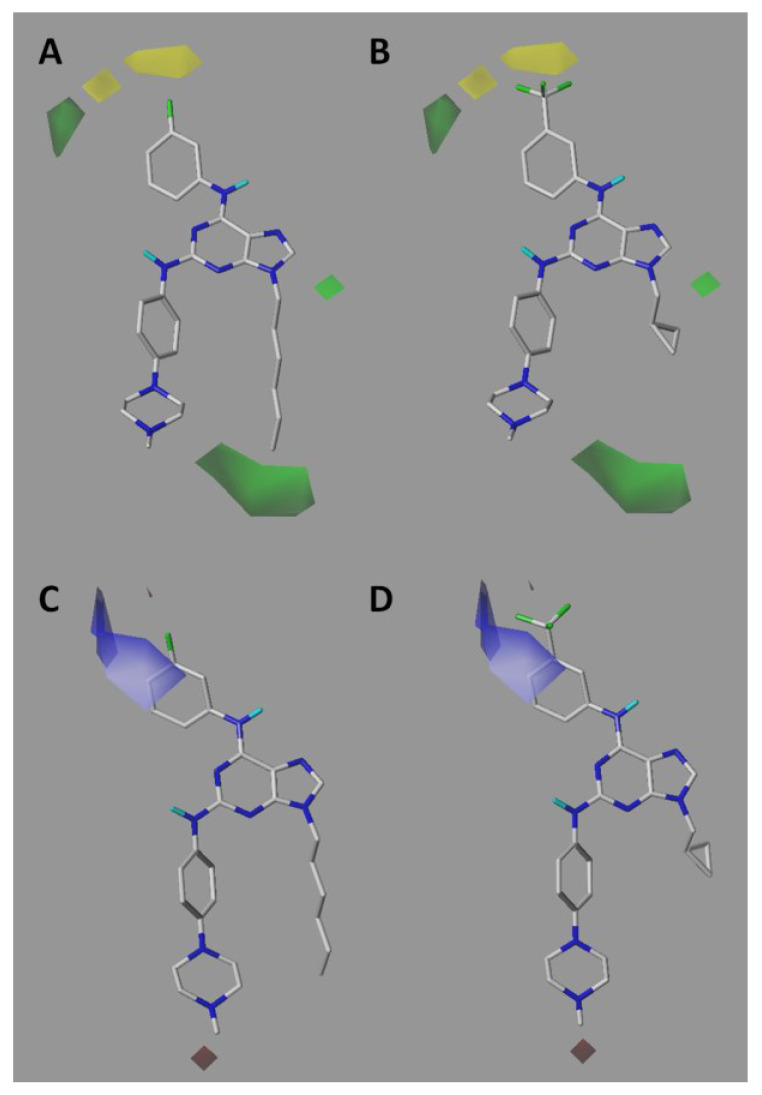
Steric (**A**,**B**) and electrostatic (**C**,**D**) contour maps in BTK. The most active compound **5h** (**A**,**C**) is compared against the least active **4d** (**B**,**D**).

For BTK, the steric contour map (Figure 6A,B) shows a large green polyhedron at the end of the alkyl chain. This means that, unlike Bcr-Abl, inserting long chains at that position is favourable for activity. This explains the lower activity of **4d** (IC_50_ = 22.68 mM, which has a short cyclopropyl ring instead of a long alkyl chain (**5h**, IC_50_ = 0.32 mM). 

A second green polyhedron is located some distance from the *para*-position of the aniline attached to the purine at C-6. This polyhedron intersects the OCF_3_ group of **4h** (IC_50_ = 0.60 mM) as well as the groups of **4i** (CN, IC_50_ = 0.91 mM) and **4l** (Cl, IC_50_ = 0.88 mM); thus, we suggest evaluating the presence of bulky functional groups at this position. Far from the *meta*-position, two yellow polyhedra intersect the trifluoromethyl group of the less active compound **4d**. The less active compounds **4a** (IC_50_ = 14.70 mM), **4m** (IC_50_ = 2.39 mM) and **8a** (IC_50_ = 6.01 mM) do not present functional groups in this position. The electrostatic contour map (Figure 6C,D) shows a large blue polyhedron in the *para*-position of the phenylamino fragment on C-6. This behaviour is the same as observed for Bcr-Abl.

**Figure 7:**
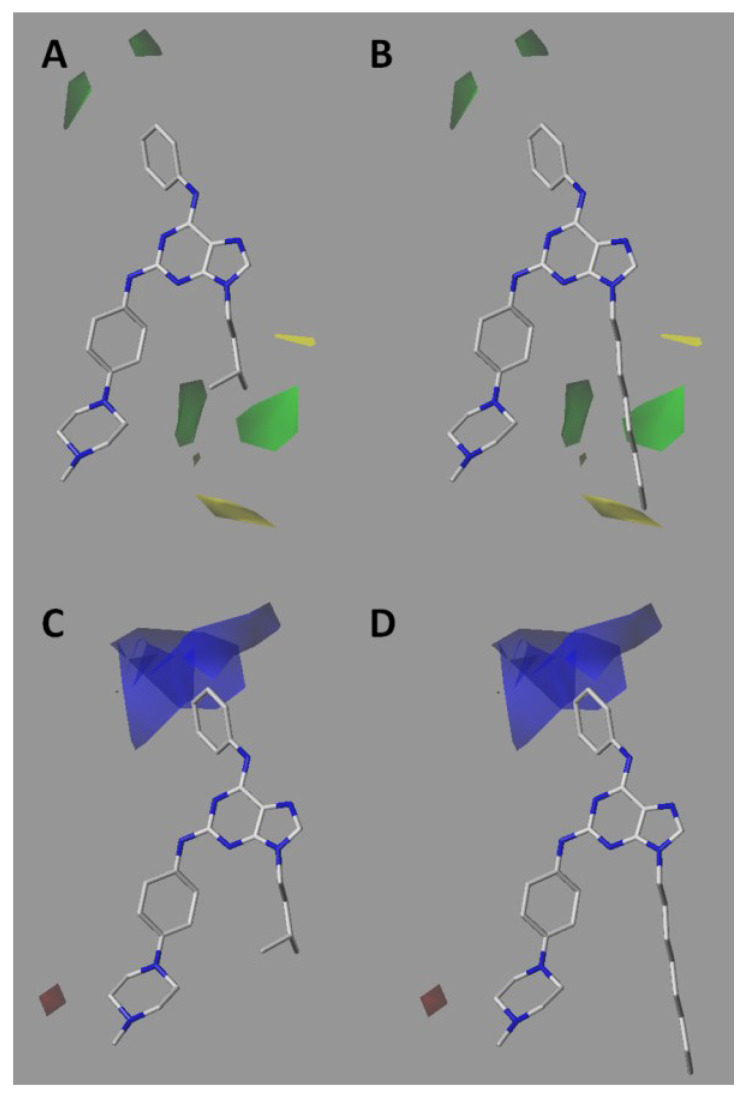
Steric (**A**,**B**) and electrostatic (**C**,**D**) contour maps in FLT3-ITD. The most active compound **5a** (**A**,**C**) is compared against the least active **5j** (**B**,**D**).

For the FLT3-ITD (Figure 7), the steric contour map shows two green polyhedra around the isopentyl group in N-9 of **5a**; the most active are on FLT3-ITD (IC_50_ = 0.23 mM). At the end of the octyl chain of compound **5j**, the least active of the series (IC_50_ = 20.28 mM) shows a yellow polyhedron. Therefore, we postulate that FLT3-ITD has greater freedom than Bcr-Abl but less than in BTK; this helps explore the bulky substitutions in N-9. 

These results are corroborated by the fact that active compounds **5b** (IC_50_ = 0.38 mM), **5d** (IC_50_ = 0.46 mM) and **5e** (IC_50_ = 0.50 mM) have chains that do not exceed the region delimited by the green polyhedra. There are also two green polyhedra in the *ortho*- and *para*-positions of the phenylamino fragment on C-6, which indicates that the presence of bulky substituents there might increase the inhibitory activity. The electrostatic contour map for FLT3-ITD (Figure 7A,B) indicates similar behaviour to Abl and BTK. There is a large blue polyhedron on the *meta-* and *para*-positions of the phenylamino fragment on C-6.

### 3.4. Molecular Docking Studies

Molecular docking simulations were performed to study the binding pattern of selected purine derivatives in the active sites of Bcr-Abl, BTK and FLT3. The molecular docking protocol was first validated by running self-docking of the co-crystalized ligand for the dasatinib-Bcr-Abl (PDB ID: 2GQG) [54], ligand 481-BTK (PDB ID: 4OT5) [55] and gliterinib-FLT3 (PDB ID: 6JQR) [56]. Autodocking validation reproduced the experimental binding mode of the co-crystallised ligand, thus, indicating that the docking protocol used is suitable for the intended docking analysis (Figure S2). The docking protocol demonstrates that the docking program succeeds in reproducing the main interactions between the co-crystallized ligands and their respective proteins (Figure S3).

According to biochemical studies, compounds **4f**, **5j** and **5b** showed potent inhibition against Bcr-Abl, BTK and FLT3, and these were also highly selective among these kinases. Therefore, the binding modes of these new purines were determined via in silico studies, using the protocols obtained with self-docking. The binding affinity scores are expressed in kcal/mol in Table 2 as well as those for the co-crystalized ligands.

Docking results for Bcr-Abl indicated that **4f** had binding energy of −67.64 kcal/mol, while **5b** and **5j** had energies of −57.32 and −58.47 kcal/mol, respectively. These values agree with the experimental results and show that **4f** is the most active compound on Bcr-Abl compared to **5b** and **5j**. **4f** had a high affinity for this kinase due to hydrogen bonds with residue M318 and favourable hydrophobic interactions with amino acids L248, G249, Y253 and V256. 

**Figure 8:**
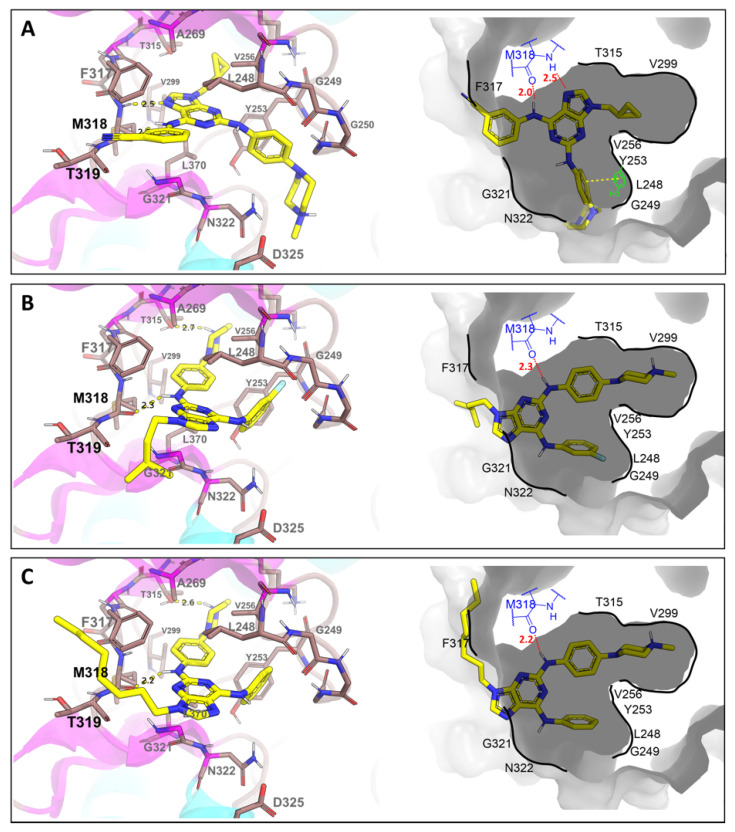
Representations of the binding modes of compounds **4f** (**A**), **5b** (**B**) and **5j** (**C**) at the Bcr-Abl active site. The image on the right is the three-dimensional representation of the best docking result for each compound. The image on the left is a top view of a diagram of ligand interactions with the binding site.

There are π–π stacking interactions between the piperazine-bound aromatic ring with the phenol of residue Y253 (Figure 8A). In contrast, **5b** and **5j** (Figure 8B,C) have an alkyl chain (isopentyl and *n*-octyl groups) that interacts unfavourably with residues L370 and Y253, thus, causing a different position to **4i**. Due to this, **5b** and **5j** only form a hydrogen bond with M318 through the N-H phenylamino fragment on C-6, which significantly reduces their hydrophobic interactions with amino acids L248, G249, Y253 and V256.

**Figure 9:**
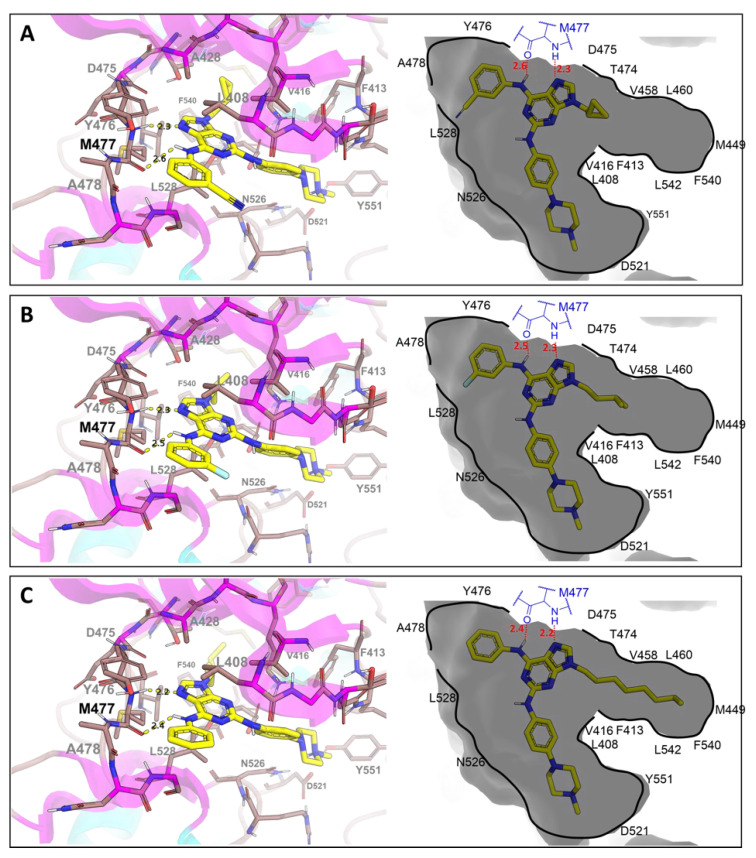
Representations of the binding modes of compounds **4f** (**A**), **5b** (**B**) and **5j** (**C**) at the BTK active site. The image on the right is the three-dimensional representation of the best docking result for each compound. The image on the left is a top view of a diagram of ligand interactions with the binding site.

In the case of BTK, the best docking results showed the same position for **4f**, **5b** and **5j** (Figure 9A–C); all these compounds formed hydrogen bonds with M477. The binding energy of these compounds matches the experimental values where compound **5j** is one of the most active with a binding energy of −86.08 kcal/mol. Figure 8C shows that the *n*-octyl radical interacts favourably with the narrow hydrophobic pocket in the enzyme active site. The interactions of amino acids F540 and L542 are important: These residues are in the innermost part of the hydrophobic pocket. Such interactions are not achievable with the alkyl radicals of **4f** and **5b** (binding energy −73.37 and 67.34 kcal/mol, respectively).

**Figure 10:**
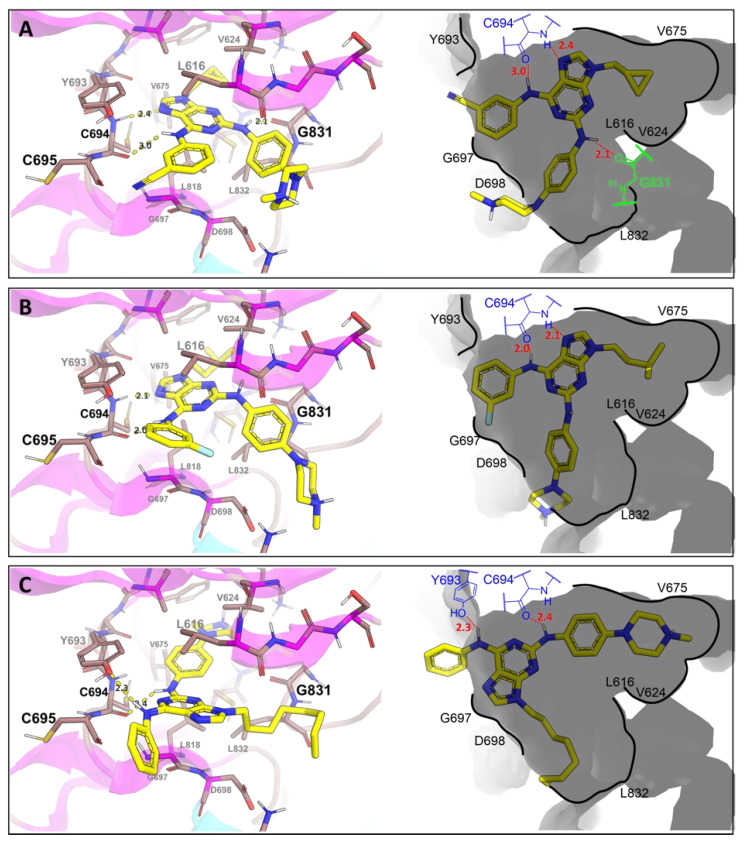
Representations of the binding modes of compounds **4f** (**A**), **5b** (**B**) and **5j** (**C**) at the FLT3 active site. The image on the right is the three-dimensional representation of the best docking result for each compound. The image on the left is a top view of a diagram of the ligand interactions with the binding site.

For FLT3 (Figure 10), we noted that **4f** established three hydrogen bonds as shown in Figure 10A. Two of these are with C694 through the secondary aromatic amine to the carbonyl (3.0 Å distance) and N-7 of the purine (2.4 Å distance). The third is between the carbonyl of G831 and the N-H of the phenylamino fragment on C-6 (2.1 Å distance). In contrast, the hydrophobic pocket in the FLT3 binding site appears to be slightly wider than Bcr-Abl. 

This explains why **5b** shows favouring hydrophobic interactions with residues V624, L616 and V675 to subsequently enhance hydrogen bonds with residue C694 (2.1 and 2.0 Å distance, Figure 10B). **5j** (Figure 10C) is completely different from **4f** and **5b** because it has a lower activity on FLT3-ITD. For **5j**, the piperazine is in the hydrophobic pocket, and the *n*-octyl is positioned in a side region to interact with the hydrophobic residues in that region. This orientation of **5j** caused the N-H phenylamino fragment on C-6 to generate a hydrogen bond with the hydroxyl group of Y693.

**Figure 11:**
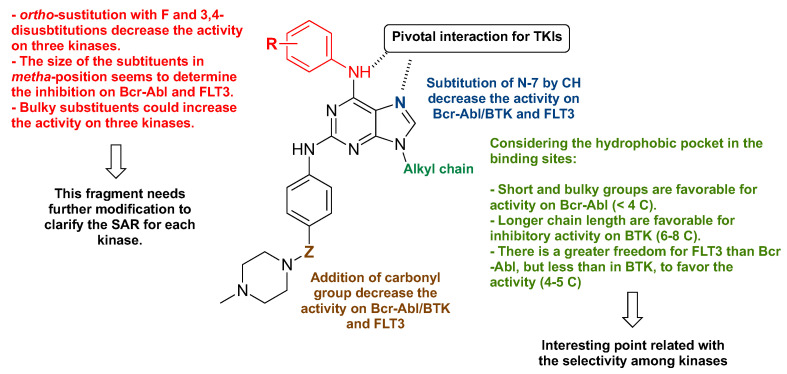
Summary of the main structure–activity relationships found in the present study.

Considering the proposed chemical modifications on **V** (Figure 3), Figure 11 summarises the results obtained via SAR and in silico studies (3D-QSAR and molecular docking) in this work.

### 3.5. Cytotoxic Activity against Cell Lines

We screened all synthesized compounds for cytotoxic activity against the cancer cell lines K562 (chronic myelogenous leukaemia expressing oncogenic kinase Bcr-Abl), HL-60 (acute promyelocytic leukaemia), MV4-11 (acute myelogenous leukaemia expressing oncogenic kinase FLT3-ITD), Ramos (non-Hodgkin’s lymphoma expressing oncogenic kinase BTK) and CEM (acute lymphoblastic leukaemia). These purine derivatives yielded micromolar GI_50_ values in all cell lines (Table 3). MV4-11 was the most sensitive cell line, and CEM was the least sensitive. In general, compound **4i** was the most cytotoxic on K562 and CEM cells (1.03 and 1.85 µM), **4j** on HL-60 and Ramos cells (0.42 and 1.30 µM) and **5a** on MV4-11 cells (0.73 µM). Interestingly, **4i**, **4j** and **5a** had high inhibition of Bcr-Abl, FLT3-ITD and BTK, which is consistent with the level of expression of these oncogenic kinases [61,62,63].

### 3.6. Inhibition of Kinases in Cell Lines

**Figure 12:**
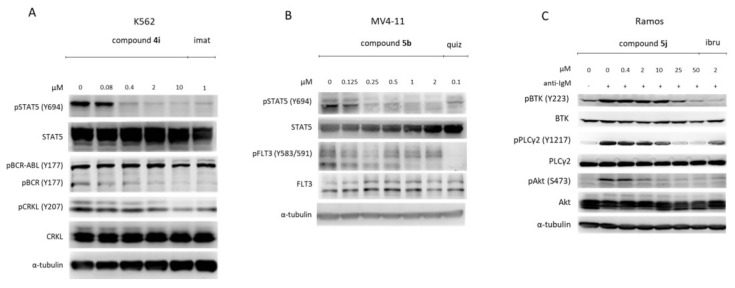
The effects of compounds **4i**, **5b** and **5j** in cell lines. K562 cells treated with **4i** (**A**), MV4-11 with **5b** (**B**) and Ramos with **5j** (**C**). Cells were treated with vehicle or the indicated concentration of the compound for 4 h. Imatinib (imat, 1 µM), quizartinib (quiz, 0.1 µM) and ibrutinib (ibru, 2 µM) were used as positive controls. Ramos cells were stimulated with anti-IgM (5 µg/mL) for 10 min before harvesting. The cells were lysed, and the proteins were detected by immunoblotting. Tubulin was used as the loading control.

Based on their inhibitory effects on recombinant kinases as well as cytotoxic effects in relevant cell line models, the purine derivatives **4i**, **5b** and **5j** were selected for further experiments to confirm the expected molecular mechanism of action. The candidate compounds were tested to determine changes in signalling pathways downstream of the oncogenic kinases in cells. Compound **4i** was investigated as a potential inhibitor of Bcr-Abl for downstream signalling on the K562 cell line, **5b** in MV4-11 cell line for the FLT3-ITD-dependent pathway and **5j** in Ramos cells for suppression of BTK-mediated oncogenic signalling. The cells were treated with increasing concentrations of the respective compounds for 4 h. In K562, treatment with **4i** inhibited autophosphorylation of Bcr (pBcr) starting at 0.08 µM (Figure 12A). 

Moreover, the compound dose-dependently decreased the level of phosphorylated STAT5 (pSTAT5) and CrkL (pCrkL), which are both direct substrates of the oncogenic Bcr-Abl kinase. MV4-11 cells were exposed to increasing concentrations of compound **5b**, thus, resulting in a clear suppression of the autophosphorylation of the oncogenic FLT3-ITD as seen in Figure 12B. In addition, **5b** inhibited the phosphorylation of STAT5, a key substrate in FLT3-ITD-mediated signalling. Finally, compound **5j** impaired the downstream signalling of oncogenic BTK in immunoglobulin M-activated Ramos cells. Treatment with **5j** decreased the levels of phosphorylated BTK in a dose-dependent manner as well as the downstream protein targets PLCγ2 and Akt (Figure 12C).

**Figure 13:**
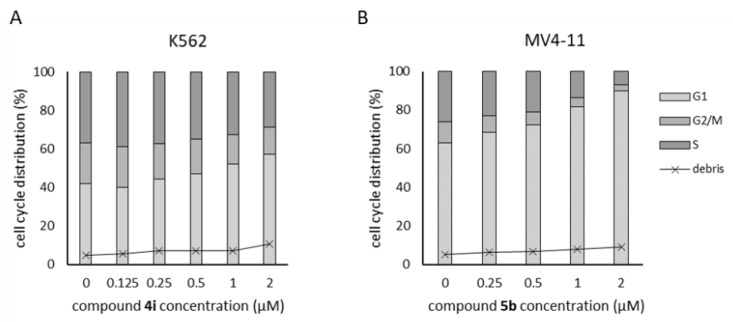
Cell cycle distribution in K562 (**A**) and MV4-11 (**B**) cells treated with **4i** and **5b**, respectively, for 24 h.

Flow cytometric analysis of the cell cycle was performed to determinate the anti-proliferative effect of the compounds. STAT5 is a protein involved in cell proliferation, and its inhibition causes arrest in the G1 phase [64]. In K562 and MV4-11 cell lines, compounds **4i** and **5b** effectively induced G1 arrest after 24 h exposure at sub-micromolar concentrations. MV4-11 cells responded more sensitively to the presence of compound **5b** versus **4i** in the cell cycle distribution in K562 (Figure 13A,B).

**Figure 14:**
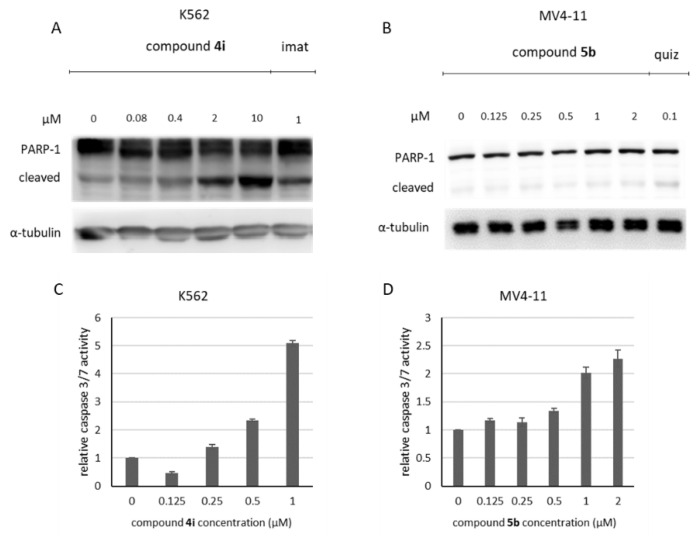
Detection of apoptotic marker PARP-1 and relative caspase 3/7 activity in cell lines treated with compounds **4i** and **5b**. Cleaved PARP-1 was detected in K562 treated with **4i** (**A**) and in MV4-11 treated with **5b** (**B**). Cells were treated for 4 h. Caspase 3/7 activity was measured in lysates of K562 (**C**) and MV4-11 cells (**D**) after 24 h treatment with the respective compounds.

Finally, we analysed the mechanism of cell death induced by the prepared compounds **4i** and **5b**. Proteolytic cleavage of PARP-1—a well-established protein involved in apoptosis—was analysed in K562 and MV4-11 treated with respective compounds for 4 h. In the case of the Bcr-Abl-dependent line K562, treatment with the purine derivative **4i** resulted in increased levels of the 89 kDa fragment of PARP-1 (Figure 14A). The cleavage of PARP-1 is an important event in the apoptotic mechanism of cell death, thus, revealing the mechanism of action [65]. The PARP-1 fragment was not detected in MV4-11 cells treated with **5b** because the compound was not effective in inducing apoptosis after 4 h of treatment (Figure 14B). 

To confirm these results, caspase 3/7 activity (responsible for PARP-1 cleavage) was further investigated. Here, 24-h treatment with **5b** and **4i** was performed in cell lines after which the relative activity of caspase 3/7 was determined in an assay based on a fluorogenic substrate. The K562 cells acted again more sensitively towards the presence of sub-micromolar doses of **4i**; there was a nearly five-fold increase in caspase 3/7 activity at 1 µM (Figure 14C). MV4-11 cells were less responsive—a higher concentration of **5b** was required for caspase induction (Figure 14D).

## 4. Conclusions

In this study, we demonstrated a new set of 2,6,9-trisubstituted purine derivatives with broad inhibitory activity on three leukaemia-related tyrosine kinases: Bcr-Abl, BTK and FLT-3-ITD. These three compounds were selected because of their effects on specific targets: **4f**, **5b** and **5j**. The potency and selectivity of these compounds were directly determined by the substitution pattern at C-6 and N-9 of the purine scaffold as well as by the presence of N-7 and the *N*-methyl-piperazine moiety. The 3D-QSAR analysis and molecular docking studies helped explain the different activities shown between these purines. The **4i**, **5b** and **5j** compounds showed cytotoxicity against cancer cell lines with high expression of these kinases. **4i** and **5b** could inhibit certain proteins found in the downstream signalling pathways of Bcr-Abl and FLT3-ITD, respectively. Therefore, these results demonstrated that synthetically accessible purine derivatives can be considered as candidate kinase inhibitors and potential anti-leukaemia drugs.

## Figures and Tables

**Table 1:**
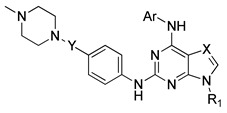
In vitro inhibition of compounds **4a**–**o**, **5a**–**l**, **8a**–**c** and **12** of the kinases Abl, BTK, FLT3-ITD and Cdk-2/Cyclin E.

					IC_50_ (µM) ^a^			
Compound	X	R	Ar	Y	Abl	BTK	FLT3-ITD	Cdk-2/E
**4a**	N	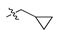	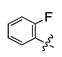	--	4.19 ± 0.83	14.70 ± 1.48	19.39 + 6.14	13.96 ± 2.33
**4b**	N	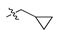	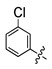	--	0.12 ± 0.03	1.28 ± 0.18	1.12 + 0.04	19.95 ± 2.07
**4c**	N	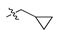	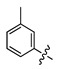	--	0.10 ± 0.00	1.30 ± 0.02	1.50 + 0.40	22.86 ± 2.08
**4d**	N	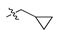	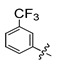	--	1.78 ± 0.07	22.68 ± 2.88	1.80 + 0.21	15.54 ± 1.70
**4e**	N	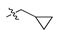	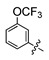	--	0.42 ± 0.10	4.12 ± 0.90	2.32 + 0.02	47.70 ± 19.41
**4f**	N	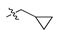	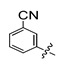	--	0.07 ± 0.01	1.43 ± 0.29	0.93 + 0.08	23.63 ± 1.15
**4g**	N	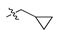	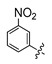	--	0.11 ± 0.01	1.36 ± 0.16	1.25 + 0.31	5.62 ± 0.52
**4h**	N	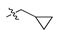	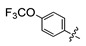	--	0.39 ± 0.08	0.60 ± 0.22	0.75 + 0.01	18.20 ± 2.05
**4i**	N	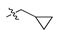	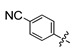	--	0.10 ± 0.03	0.91 ± 0.19	0.37 + 0.08	10.85 ± 1.60
**4j**	N	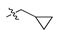	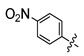	--	0.54 ± 0.02	0.94 ± 0.04	0.53 + 0.00	23.81 ± 6.05
**4k**	N	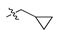	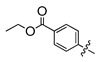	--	0.04 ± 0.01	1.16 ± 0.18	1.16 + 0.17	>50.00
**4l**	N	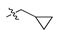	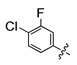	--	0.20 ± 0.05	0.88 ± 0.19	1.03 + 0.15	26.63 ± 3.37
**4m**	N	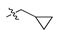	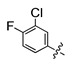	--	0.27 ± 0.05	2.39 ± 0.43	1.54 + 0.35	10.80 ± 3.84
**4n**	N	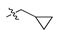	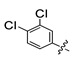	--	0.07 ± 0.01	3.90 ± 0.05	2.53 + 0.65	12.90 ± 1.70
**4o**	N	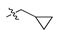	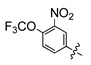	--	0.44 ± 0.12	5.03 ± 0.99	2.67 + 1.02	12.60 ± 2.12
**5a**	N	isopentyl	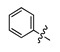	--	1.78 ± 0.20	2.22 ± 0.96	0.23 + 0.07	22.08 ± 0.38
**5b**	N	isopentyl	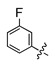	--	>50.00	3.78 ± 0.86	0.38 + 0.10	21.12 ± 1.91
**5c**	N	isopentyl	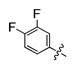	--	2.28 ± 0.80	1.89 ± 0.17	0.79 + 0.09	62.52 ± 15.01
**5d**	N	*n*-pentyl	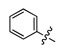	--	1.23 ± 0.02	0.42 ± 0.14	0.46 + 0.07	31.11 ± 2.91
**5e**	N	*n*-pentyl	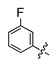	--	0.65 ± 0.07	0.36 ± 0.04	0.50 + 0.05	19.01 ± 8.98
**5f**	N	*n*-pentyl	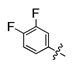	--	1.36 ± 0.50	0.93 ± 0.14	1.02 + 0.10	20.79 ± 3.18
**5g**	N	*n*-hexyl	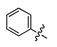	--	9.82 ± 1.87	0.91 ± 0.02	1.91 + 0.18	27.88 ± 1.04
**5h**	N	*n*-hexyl	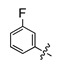	--	>100	0.32 ± 0.01	2.31 + 0.01	80.75 ± 12.48
**5i**	N	*n*-hexyl	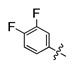	--	33.97 ± 1.63	0.46 ± 0.03	2.74 + 0.33	47.32 ± 31.58
**5j**	N	*n*-octyl	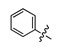	--	>100	0.41 ± 0.10	20.28 + 0.21	19.04 ± 1.55
**5k**	N	*n*-octyl	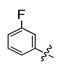	--	>100	0.97 ± 0.22	>20.00	25.58 ± 3.27
**5l**	N	*n*-octyl	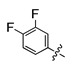	--	84.46 ± 5.90	0.58 ± 0.33	>20.00	32.19 ± 1.33
**8a**	N	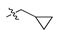	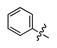	-CO-	1.21 ± 0.11	6.01 ± 0.72	1.95 + 0.11	31.42 ± 3.18
**8b**	N	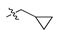	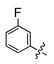	-CO-	0.57 ± 0.03	1.72 ± 0.19	1.60 + 0.42	0.57 ± 0.03
**8c**	N	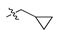	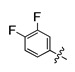	-CO-	0.94 ± 0.22	2.73 ± 0.17	1.81 + 0.02	14.15 ± 1.24
**12**	CH	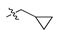	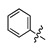	--	62.28 ± 12.11	>20.00	>20.00	46.24 ± 1.49

^a^ IC_50_ values were determined in at least three independent experiments in the range of 0.01–100 µM.

**Table 2:** Binding affinity scores (kcal/mol) for selected purines in the enzyme active sites.

	MM-GBSA		
Compound	Bcr-Abl	BTK	FLT3
**4f**	−67.64	−73.37	−60.17
**5b**	−57.32	−67.34	−74.05
**5j**	−58.47	−86.08	−61.34
**Dasatinib**	−71.75	―	―
**Lig-4OT5**	―	−89.00	―
**Gliterinib**	―	―	−75.71

**Table 3:** The in vitro cytotoxicity of compounds **4a**–**o**, **5a**–**l**, **8a**–**c** and **12** on cancer cell lines.

Compound	GI_50_ (µM) ^a^				
	K562	HL-60	MV4-11	Ramos	CEM
**4a**	21.32 ± 0.63	1.82 ± 0.35	6.47 ± 1.82	15.58 ± 0.88	17.12 ± 0.40
**4b**	1.99 ± 0.82	1.93 ± 1.05	1.94 ± 0.16	10.62 ± 3.08	19.05 ± 1.07
**4c**	2.86 ± 0.76	9.79 ± 0.87	1.42 ± 0.23	12.54 ± 0.48	16.45 ± 0.36
**4d**	4.51 ± 0.36	0.72 ± 0.16	2.84 ± 0.29	3.83 ± 0.10	23.78 ± 0.08
**4e**	5.18 ± 1.68	1.40 ± 0.13	2.88 ± 0.01	12.84 ± 0.03	19.53 ± 0.61
**4f**	3.68 ± 0.78	1.56 ± 0.88	1.05 ± 0.10	5.86 ± 3.64	13.44 ±1 0.08
**4g**	2.93 ± 0.59	8.51 ± 1.79	1.98 ± 0.46	4.87 ± 0.01	11.45 ± 3.93
**4h**	2.90 ± 0.91	1.08 ± 0.31	2.45 ± 0.09	11.73 ± 2.88	14.95 ± 0.92
**4i**	1.03 ± 0.21	3.17 ± 0.57	0.89 ± 0.07	1.49 ± 0.05	1.85 ± 0.10
**4j**	2.84 ± 1.04	0.42 ± 0.08	2.21 ± 0.71	1.30 ± 0.5	2.52 ± 0.29
**4k**	2.70 ± 1.31	1.93 ± 0.17	2.43 ± 0.59	12.34 ± 1.58	13.91 ± 1.12
**4l**	3.45 ± 1.61	3.22 ± 0.74	2.56 ± 0.64	11.18 ± 2.64	15.41 ± 0.55
**4m**	2.57 ± 0.52	0.86 ± 0.11	1.88 ± 0.06	7.52 ± 0.41	13.35 ± 1.58
**4n**	5.54 ± 1.56	1.52 ± 0.34	4.81 ± 1.54	8.02 ± 1.30	17.54 ± 0.08
**4o**	2.60 ± 1.25	1.68 ± 0.01	1.49 ± 0.33	4.46 ± 0.38	14.83 ± 1.32
**5a**	8.93± 5.52	1.52 ± 0.61	0.73 ± 0.25	10.07 ± 2.98	16.39 ± 2.41
**5b**	18.56 ± 3.73	2.04 ± 0.58	2.89 ± 0.95	18.69 ± 3.10	>25
**5c**	9.40 ± 0.78	1.59 ± 0.47	1.43 ± 0.56	8.87 ± 0.94	16.81 ± 3.34
**5d**	6.70 ± 2.48	3.98 ± 1.06	1.28 ± 0.38	13.78 ± 3.13	19.12 ± 3.39
**5e**	2.14 ± 0.80	1.31 ± 0.00	1.31 ± 0.71	11.61 ± 1.46	19.03 ± 3.89
**5f**	>25	3.61 ± 1.02	1.61 ± 0.63	>25	>25
**5g**	15.50 ± 4.98	6.11 ± 0.76	4.27 ± 1.45	22.53 ± 1.23	34.73 ± 1.37
**5h**	24.67 ± 0.41	8.77 ± 3.84	3.54 ± 1.53	>25	>25
**5i**	>25	3.91 ± 0.07	3.86 ± 1.08	>25	>25
**5j**	8.19 ± 0.06	1.25 ± 0.11	4.45 ± 1.48	14.25 ± 2.61	23.58 ± 2.01
**5k**	10.04 ± 6.31	2.16 ± 0.48	12.42 ± 3.93	15.74 ± 0.37	21.27 ± 0.31
**5l**	11.85 ± 2.19	1.51 ± 1.49	16.28 ± 4.99	18.85 ± 2.11	>25
**8a**	8.91 ± 1.64	6.64 ± 1.42	1.24 ± 0.21	16.57 ± 0.67	>25
**8b**	4.91 ± 0.01	1.08 ± 0.10	1.48 ± 0.54	7.07 ± 0.32	20.23 ± 3.73
**8c**	6.07 ± 0.08	2.39 ± 0.89	1.52 ± 0.31	14.69 ± 0.02	20.69 ± 4.07
**12**	6.38 ± 1.14	3.58 ± 0.65	8.28 ± 2.90	12.83 ± 0.61	15.51 ± 1.46

^a^ GI_50_ values were determined in three independent experiments in the range of 0.1 to 25 µM.

## Data Availability

Not applicable.

## Supplementary Materials

The following supporting information can be downloaded at: https://www.mdpi.com/article/10.3390/pharmaceutics14061294/s1, ^1^H-, ^13^C- and ^19^F-NMR and HRMS of selected compounds; Table S1: Statistical parameters for CoMFA analysis in Bcr-Abl, BTK and FLT3-ITD; Table S2: Summary of external validation parameters for CoMFA models; Table S3: Experimental and predicted pIC_50_ and residual values for analysed compounds according to CoMFA; Figure S1: Plots of experimental pIC_50_ versus predicted pIC_50_ for CoMFA in Bcr-Abl, BTK and FLT3-ITD; Figure S2: Co-crystallized ligands with their experimentally determined binding mode are shown in green, while the docking pose of the ligands from our self-docking protocol are shown in yellow; Figure S3: Schemes of the essential interactions reproduced by the self-docking poses. The important hydrogen bonds are amino acids M318, M477 and C694 for Abl (A), BTK (B) and FLT3 (C), respectively; Figure S4: Docking protocol for synthetized ligands; Figure S5: Graphic representation of the active site of Bcr-Abl protein, Table S4: The main energetic contributions per residues between the active site of Bcr-Abl with the synthesized ligands; Table S5: Prime MM-GBSA calculation of the docked complexes (ligands-Abl); Figure S6: Graphic representation of the active site of BTK protein; Table S6: Main energetic contributions per residues between the active site of BTK with the synthesized ligands; Table S7: Prime MM-GBSA calculation of the docked complexes (ligands-BTK); Figure S7: Graphic representation of the hydrophobic pocket of BTK protein and 5j structure in yellow; Table S8: The main energetic contributions per residues the hydrophobic pocket of BTK with synthetized ligands; Figure S8: Graphic representation of the active site of FLT3 protein; Table S9: The main energetic contributions per residues between the active site of FLT3 with the synthesized ligands; Table S10: Prime MM-GBSA calculation of the docked complexes (ligand-FLT3).

## References

[1] Méndez-Ferrer S., Bonnet D., Steensma D.P., Hasserjian R.P., Ghobrial I.M., Gribben J.G., Andreeff M., Krause D.S. (2020). Bone marrow niches in haematological malignancies. Nat. Rev. Cancer.

[2] Taylor J., Xiao W., Abdel-Wahab O. (2017). Diagnosis and classification of hematologic malignancies on the basis of genetics. Blood.

[3] Godley L.A., Shimamura A. (2017). Genetic predisposition to hematologic malignancies: Management and surveillance. Blood.

[4] Zeidner J.F., Karp J.E., Blackford A.L., Foster M.C., Dees E.C., Smith G., Ivy S.P., Harris P. (2016). Phase I Clinical Trials in Acute Myeloid Leukemia: 23-Year Experience From Cancer Therapy Evaluation Program of the National Cancer Institute. J. Nat. Cancer Inst..

[5] Rossari F., Minutolo F., Orciuolo E. (2018). Past, present, and future of Bcr-Abl inhibitors: From chemical development to clinical efficacy. J. Hematol. Oncol..

[6] Kannaiyan R., Mahadevan D. (2018). A comprehensive review of protein kinase inhibitors for cancer therapy. Expert Rev. Anticancer.

[7] Liang C., Tian D., Ren X., Ding S., Jia M., Xin M., Thareja S. (2018). The development of Bruton’s tyrosine kinase (BTK) inhibitors from 2012 to 2017: A mini-review. Eur. J. Med. Chem..

[8] Kanfar S.S., Chan S.M., Gupta V., Schimmer A.D., Schuh A.C., Sibai H., Yee K.W.L., Minden M.D. (2016). Outcomes of Adult Philadelphia Positive Acute Lymphoblastic Leukemia Patients Treated with Pediatric Multi-Agent Chemotherapy and Imatinib and the Impact of Residual Disease Monitoring on Survival. Blood.

[9] Quintas-Cardama A., Cortes J. (2009). Molecular biology of bcr-abl1-positive chronic myeloid leukemia. Blood.

[10] Nicolini F.E., Mauro M.J., Martinelli G., Kim D.W., Soverini S., Muller M.C., Hochhaus A., Cortes J., Chuah C., Dufva I.H. (2009). Epidemiologic study on survival of chronic myeloid leukemia and Ph(+) acute lymphoblastic leukemia patients with BCR-ABL T315I mutation. Blood.

[11] Quintas-Cardama A., Kantarjian H., Cortes J. (2007). Flying under the radar: The new wave of BCR-ABL inhibitors. Nat. Rev. Drug Discov..

[12] Azevedo A.P., Reichert A., Afonso C., Alberca M.D., Tavares P., Lima F. (2017). BCR-ABL V280G Mutation, Potential Role in Imatinib Resistance: First Case Report. Clin. Med. Insights Oncol..

[13] Weisberg E., Manley P.W., Cowan-Jacob S.W., Hochhaus A., Griffin J.D. (2007). Second generation inhibitors of BCR-ABL for the treatment of imatinib-resistant chronic myeloid leukaemia. Nat. Rev. Cancer.

[14] Nicolini F.E., Ibrahim A.R., Soverini S., Martinelli G., Muller M.C., Hochhaus A., Dufva I.H., Kim D.W., Cortes J., Mauro M.J. (2013). The BCR-ABLT315I mutation compromises survival in chronic phase chronic myelogenous leukemia patients resistant to tyrosine kinase inhibitors, in a matched pair analysis. Haematologica.

[15] O’Hare T., Walters D.K., Stoffregen E.P., Jia T., Manley P.W., Mestan J., Cowan-Jacob S.W., Lee F.Y., Heinrich M.C., Deininger M.W. (2005). In vitro activity of Bcr-Abl inhibitors AMN107 and BMS-354825 against clinically relevant imatinib-resistant Abl kinase domain mutants. Cancer Res..

[16] Mohamed A.J., Yu L., Backesjo C.M., Vargas L., Faryal R., Aints A., Christensson B., Berglof A., Vihinen M., Nore B.F. (2009). Bruton’s tyrosine kinase (Btk): Function, regulation, and transformation with special emphasis on the PH domain. Immunol. Rev..

[17] Pal Singh S., Dammeijer F., Hendriks R.W. (2018). Role of Bruton’s tyrosine kinase in B cells and malignancies. Mol. Cancer.

[18] Suri D., Rawat A., Singh S. (2016). X-linked Agammaglobulinemia. Indian J. Pediatr..

[19] Thomas J.D., Sideras P., Smith C.I., Vorechovsky I., Chapman V., Paul W.E. (1993). Colocalization of X-linked agammaglobulinemia and X-linked immunodeficiency genes. Science.

[20] Vetrie D., Vorechovsky I., Sideras P., Holland J., Davies A., Flinter F., Hammarstrom L., Kinnon C., Levinsky R., Bobrow M. (1993). The gene involved in X-linked agammaglobulinaemia is a member of the src family of protein-tyrosine kinases. Nature.

[21] Campbell R., Chong G., Hawkes E.A. (2018). Novel Indications for Bruton’s Tyrosine Kinase Inhibitors, beyond Hematological Malignancies. J. Clin. Med..

[22] Hallek M. (2019). Chronic lymphocytic leukemia: 2020 update on diagnosis, risk stratification and treatment. Am. J. Hematol..

[23] Byrd J.C., Harrington B., O’Brien S., Jones J.A., Schuh A., Devereux S., Chaves J., Wierda W.G., Awan F.T., Brown J.R. (2016). Acalabrutinib (ACP-196) in Relapsed Chronic Lymphocytic Leukemia. N. Engl. J. Med..

[24] Woyach J.A., Furman R.R., Liu T.-M., Ozer H.G., Zapatka M., Ruppert A.S., Xue L., Li D.H.-H., Steggerda S.M., Versele M. (2014). Resistance mechanisms for the Bruton’s tyrosine kinase inhibitor ibrutinib. N. Engl. J. Med..

[25] Liu L., Shi B., Wang X., Xiang H. (2018). Strategies to overcome resistance mutations of Bruton’s tyrosine kinase inhibitor ibrutinib. Fut. Med. Chem..

[26] Schiffer C.A. (2003). Hematopoietic growth factors and the future of therapeutic research on acute myeloid leukemia. N. Engl. J. Med..

[27] Gilliland D.G., Griffin J.D. (2002). The roles of FLT3 in hematopoiesis and leukemia. Blood.

[28] Takahashi S. (2011). Downstream molecular pathways of FLT3 in the pathogenesis of acute myeloid leukemia: Biology and therapeutic implications. J. Hematol. Oncol..

[29] Weisberg E., Boulton C., Kelly L.M., Manley P., Fabbro D., Meyer T., Gilliland D.G., Griffin J.D. (2002). Inhibition of mutant FLT3 receptors in leukemia cells by the small molecule tyrosine kinase inhibitor PKC412. Cancer Cell.

[30] Wu M., Li C., Zhu X. (2018). FLT3 inhibitors in acute myeloid leukemia. J. Hematol. Oncol..

[31] Kiyoi H., Kawashima N., Ishikawa Y. (2020). FLT3 mutations in acute myeloid leukemia: Therapeutic paradigm beyond inhibitor development. Cancer Sci..

[32] Tollkuci E., Tran T., Myers R. (2020). Gilteritinib administration in a hemodialysis patient. J. Oncol. Pharm. Pr..

[33] Yamaura T., Nakatani T., Uda K., Ogura H., Shin W., Kurokawa N., Saito K., Fujikawa N., Date T., Takasaki M. (2018). A novel irreversible FLT3 inhibitor, FF-10101, shows excellent efficacy against AML cells with FLT3 mutations. Blood.

[34] Larrosa-Garcia M., Baer M.R. (2017). FLT3 Inhibitors in Acute Myeloid Leukemia: Current Status and Future Directions. Mol. Cancer.

[35] Musumeci F., Schenone S., Grossi G., Brullo C., Sanna M. (2015). Analogs, formulations and derivatives of imatinib: A patent review. Expert Opin. Ther. Pat..

[36] Sharma S., Singh J., Ojha R., Singh H., Kaur M., Bedi P.M.S., Nepali K. (2016). Design strategies, structure activity relationship and mechanistic insights for purines as kinase inhibitors. Eur. J. Med. Chem..

[37] Laufer S.A., Domeyer D.M., Scior T.R., Albrecht W., Hauser D.R. (2005). Synthesis and biological testing of purine derivatives as potential ATP-competitive kinase inhibitors. J. Med. Chem..

[38] Legraverend M., Grierson D.S. (2006). The purines: Potent and versatile small molecule inhibitors and modulators of key biological targets. Bioorg. Med. Chem..

[39] Welsch M.E., Snyder S.A., Stockwell B.R. (2010). Privileged scaffolds for library design and drug discovery. Curr. Opin. Chem. Biol..

[40] Azam M., Nardi V., Shakespeare W.C., Metcalf C.A., Bohacek R.S., Wang Y., Sundaramoorthi R., Sliz P., Veach D.R., Bornmann W.G. (2006). Activity of dual SRC-ABL inhibitors highlights the role of BCR/ABL kinase dynamics in drug resistance. Proc. Natl. Acad. Sci. USA.

[41] Shi Q., Tebben A., Dyckman A.J., Li H., Liu C., Lin J., Spergel S., Burke J.R., McIntyre K.W., Olini G.C. (2014). Purine derivatives as potent Bruton’s tyrosine kinase (BTK) inhibitors for autoimmune diseases. Bioorg. Med. Chem. Lett..

[42] Gucky T., Reznickova E., Radosova Muchova T., Jorda R., Klejova Z., Malinkova V., Berka K., Bazgier V., Ajani H., Lepsik M. (2018). Discovery of N(2)-(4-Amino-cyclohexyl)-9-cyclopentyl- N(6)-(4-morpholin-4-ylmethyl-phenyl)- 9H-purine-2,6-diamine as a Potent FLT3 Kinase Inhibitor for Acute Myeloid Leukemia with FLT3 Mutations. J. Med. Chem..

[43] Wang Y., Shakespeare W.C., Huang W.S., Sundaramoorthi R., Lentini S., Das S., Liu S., Banda G., Wen D., Zhu X. (2008). Novel N9-arenethenyl purines as potent dual Src/Abl tyrosine kinase inhibitors. Bioorg. Med. Chem. Lett..

[44] Bertrand J., Dostalova H., Krystof V., Jorda R., Castro A., Mella J., Espinosa-Bustos C., Maria Zarate A., Salas C.O. (2020). New 2,6,9-trisubstituted purine derivatives as Bcr-Abl and Btk inhibitors and as promising agents against leukemia. Bioorg. Chem..

[45] Salas C.O., Zarate A.M., Kryštof V., Mella J., Faundez M., Brea J., Loza M.I., Brito I., Hendrychová D., Jorda R. (2020). Promising 2,6,9-Trisubstituted Purine Derivatives for Anticancer Compounds: Synthesis, 3D-QSAR, and Preliminary Biological Assays. Int. J. Mol. Sci..

[46] Calderon-Arancibia J., Espinosa-Bustos C., Canete-Molina A., Tapia R.A., Faundez M., Torres M.J., Aguirre A., Paulino M., Salas C.O. (2015). Synthesis and Pharmacophore Modelling of 2,6,9-Trisubstituted Purine Derivatives and Their Potential Role as Apoptosis-Inducing Agents in Cancer Cell Lines. Molecules.

[47] Zárate A.M., Espinosa-Bustos C., Guerrero S., Fierro A., Oyarzún-Ampuero F., Quest A.F.G., Di Marcotullio L., Loricchio E., Caimano M., Calcaterra A. (2021). A New Smoothened Antagonist Bearing the Purine Scaffold Shows Antitumour Activity In Vitro and In Vivo. Int. J. Mol. Sci..

[48] Brasca M.G., Amboldi N., Ballinari D., Cameron A., Casale E., Cervi G., Colombo M., Colotta F., Croci V., D’Alessio R. (2009). Identification of N,1,4,4-Tetramethyl-8-{[4-(4-methylpiperazin-1-yl)phenyl]amino}-4,5-dihydro-1H-pyrazolo [4,3-h]quinazoline-3-carboxamide (PHA-848125), a Potent, Orally Available Cyclin Dependent Kinase Inhibitor. J. Med. Chem..

[49] Byers L.A., Diao L., Wang J., Saintigny P., Girard L., Peyton M., Shen L., Fan Y., Giri U., Tumula P.K. (2013). An Epithelial–Mesenchymal Transition Gene Signature Predicts Resistance to EGFR and PI3K Inhibitors and Identifies Axl as a Therapeutic Target for Overcoming EGFR Inhibitor Resistance. Clin. Cancer Res..

[50] Clark M., Cramer III R.D., Van Opdenbosch N. (1989). Validation of the general purpose tripos 5.2 force field. J. Comput. Chem..

[51] Golbraikh A., Tropsha A. (2002). Beware of q2!. J. Mol. Graph. Model..

[52] Tropsha A. (2010). Best Practices for QSAR Model Development, Validation, and Exploitation. Mol. Infor..

[53] (2021). Schrödinger Release 2021-1.

[54] Tokarski J.S., Newitt J.A., Chang C.Y., Cheng J.D., Wittekind M., Kiefer S.E., Kish K., Lee F.Y., Borzillerri R., Lombardo L.J. (2006). The structure of Dasatinib (BMS-354825) bound to activated ABL kinase domain elucidates its inhibitory activity against imatinib-resistant ABL mutants. Cancer Res..

[55] Lou Y., Han X., Kuglstatter A., Kondru R.K., Sweeney Z.K., Soth M., McIntosh J., Litman R., Suh J., Kocer B. (2015). Structure-based drug design of RN486, a potent and selective Bruton’s tyrosine kinase (BTK) inhibitor, for the treatment of rheumatoid arthritis. J. Med. Chem..

[56] Kawase T., Nakazawa T., Eguchi T., Tsuzuki H., Ueno Y., Amano Y., Suzuki T., Mori M., Yoshida T. (2019). Effect of Fms-like tyrosine kinase 3 (FLT3) ligand (FL) on antitumor activity of gilteritinib, a FLT3 inhibitor, in mice xenografted with FL-overexpressing cells. Oncotarget.

[57] Jacobson M.P., Friesner R.A., Xiang Z., Honig B. (2002). On the role of the crystal environment in determining protein side-chain conformations. J. Mol. Biol..

[58] Jacobson M.P., Pincus D.L., Rapp C.S., Day T.J., Honig B., Shaw D.E., Friesner R.A. (2004). A hierarchical approach to all-atom protein loop prediction. Proteins.

[59] Friesner R.A., Banks J.L., Murphy R.B., Halgren T.A., Klicic J.J., Mainz D.T., Repasky M.P., Knoll E.H., Shelley M., Perry J.K. (2004). Glide: A new approach for rapid, accurate docking and scoring. 1. Method and assessment of docking accuracy. J. Med. Chem..

[60] Friesner R.A., Murphy R.B., Repasky M.P., Frye L.L., Greenwood J.R., Halgren T.A., Sanschagrin P.C., Mainz D.T. (2006). Extra precision glide: Docking and scoring incorporating a model of hydrophobic enclosure for protein-ligand complexes. J. Med. Chem..

[61] Lozzio C., Lozzio B. (1975). Human chronic myelogenous leukemia cell-line with positive Philadelphia chromosome. Blood.

[62] Quentmeier H., Reinhardt J., Zaborski M., Drexler H.G. (2003). FLT3 mutations in acute myeloid leukemia cell lines. Leukemia.

[63] Guo B., Kato R.M., Garcia-Lloret M., Wahl M.I., Rawlings D.J. (2000). Engagement of the human pre-B cell receptor generates a lipid raft-dependent calcium signaling complex. Immunity.

[64] Baśkiewicz-Masiuk M., Machaliński B. (2004). The role of the STAT5 proteins in the proliferation and apoptosis of the CML and AML cells. Eur. J. Haematol..

[65] Gobeil S., Boucher C.C., Nadeau D., Poirier G.G. (2001). Characterization of the necrotic cleavage of poly(ADP-ribose) polymerase (PARP-1): Implication of lysosomal proteases. Cell Death Differ..

