# Supplementary material for: Design, Synthesis, In Silico Studies and Inhibitory Activity towards Bcr-Abl, BTK and FLT3-ITD of New 2,6,9-Trisubstituted Purine Derivatives as Potential Agents for the Treatment of Leukaemia
Source: Pharmaceutics. 2022 Jun 17;14(6):1294. doi: 10.3390/pharmaceutics14061294 (PMC9228270; doi:10.3390/pharmaceutics14061294)
Supplement: Supplementary file 1 [file pharmaceutics-14-01294-s001.zip › pharmaceutics-1775028-supplementary.pdf]

Supplementary Materials for

Design, synthesis, in silico studies, and inhibitory activity towards Bcr-Abl, BTK, and FLT3-ITD of new 2,6,9-trisubstituted purine derivatives as potential agents for the treatment of leukaemia

Jeanluc Bertrand,[1,#] Hana Dostálová,[2,#] Vladimír Kryštof,[2,*] Radek Jorda,[2,3] Thalía Delgado,[1] Alejandro Castro-Alvarez,[4] Jaime Mella,[5,6] David Cabezas,[5] Mario Faúndez,[7] Christian Espinosa-Bustos[7] and Cristian O. Salas[1,*]

[1] Departamento de Química Orgánica, Facultad de Química y de Farmacia, Pontificia Universidad Católica de Chile, Avenida Vicuña Mackenna 4860, 702843, Santiago, Chile; jgbertrand@uc.cl (J.B.), tdelgado@uc.cl (T.D.)

[2] Department of Experimental Biology, Palacký University Olomouc, Šlechtitelů 27, 78371 Olomouc, Czech Republic; hana.dostalva@upol.cz (H.D.), radek.jorda@upol.cz (R.J.)

[3] Institute of Molecular and Translational Medicine, Faculty of Medicine and Dentistry, Palacký University Olomouc, Hněvotínská 5, 77900, Olomouc, Czech Republic.

[4] Departamento de Ciencias Preclínicas, Facultad de Medicina, Universidad de La Frontera, Manuel Montt 112, 4780000, Temuco, Chile; qf.alec.astro@gmail.com

[5] Instituto de Química y Bioquímica, Facultad de Ciencias, Universidad de Valparaíso, 2360102, Avenida Gran Bretaña 1111, 2360102, Valparaíso, Chile; jaime.mella@uv.cl (J.M.), david.cg172012@gmail.com (D.C.)

[6] Facultad de Farmacia, Centro de Investigación Farmacopea Chilena, Universidad de Valparaíso, Avenida Gran Bretaña 1093, 2360102, Valparaíso, Chile.

[7] Departamento de Farmacia, Facultad de Química y de Farmacia, Pontificia Universidad Católica de Chile, Avenida Vicuña Mackenna 4860, 702843, Santiago, Chile; mfaundez@uc.cl (M.F), ccespino@uc.cl (C.E-B)

Index

^{1}H-, ^{13}C-, and ^{19}F-NMR of selected compounds……..……………………………….2-43

HRMS of selected compounds…..………………….……………………………..44-59

3D-QSAR………………………………………………………………………60-62

Molecular Docking details…………………...……………………………………63-70

^{1}H NMR spectra of compound **10a**

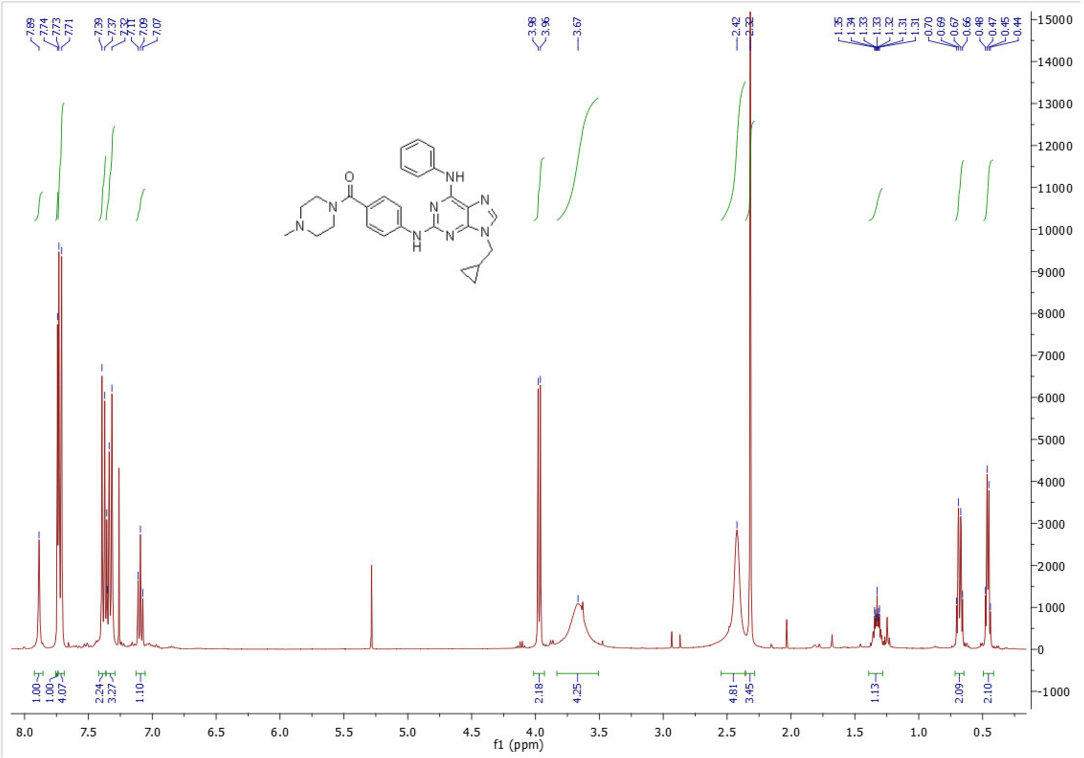

^{13}C NMR spectra of compound **10a**

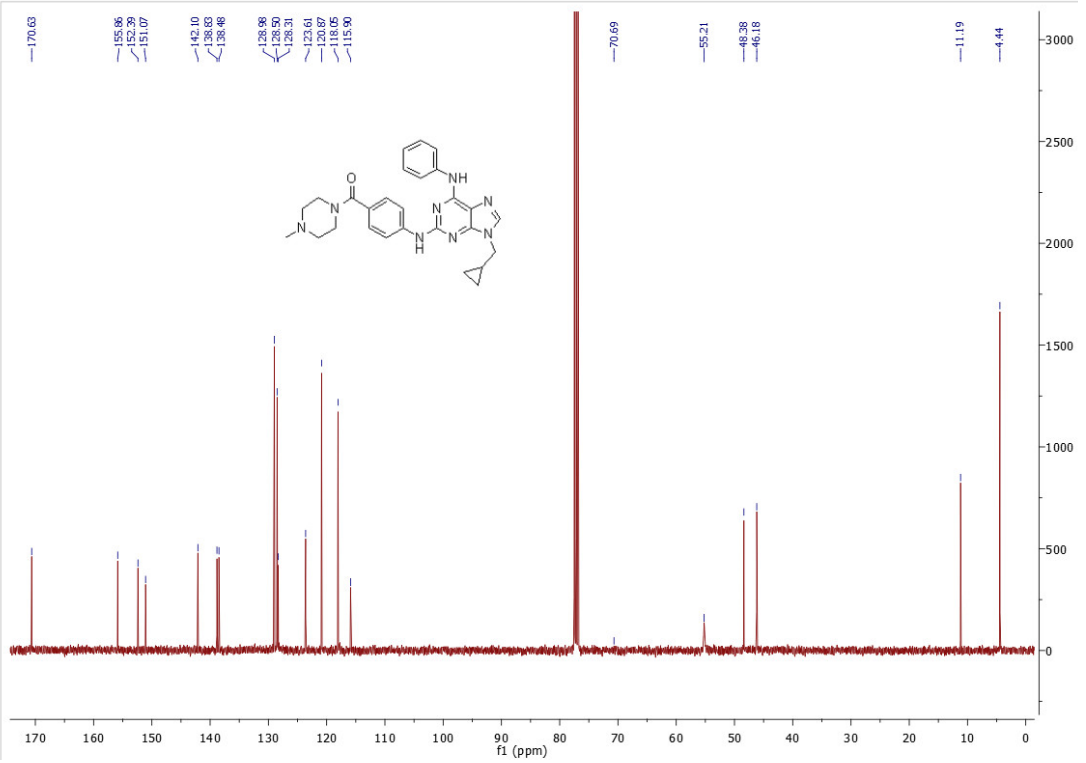

^{1}H NMR spectra of compound **10b**

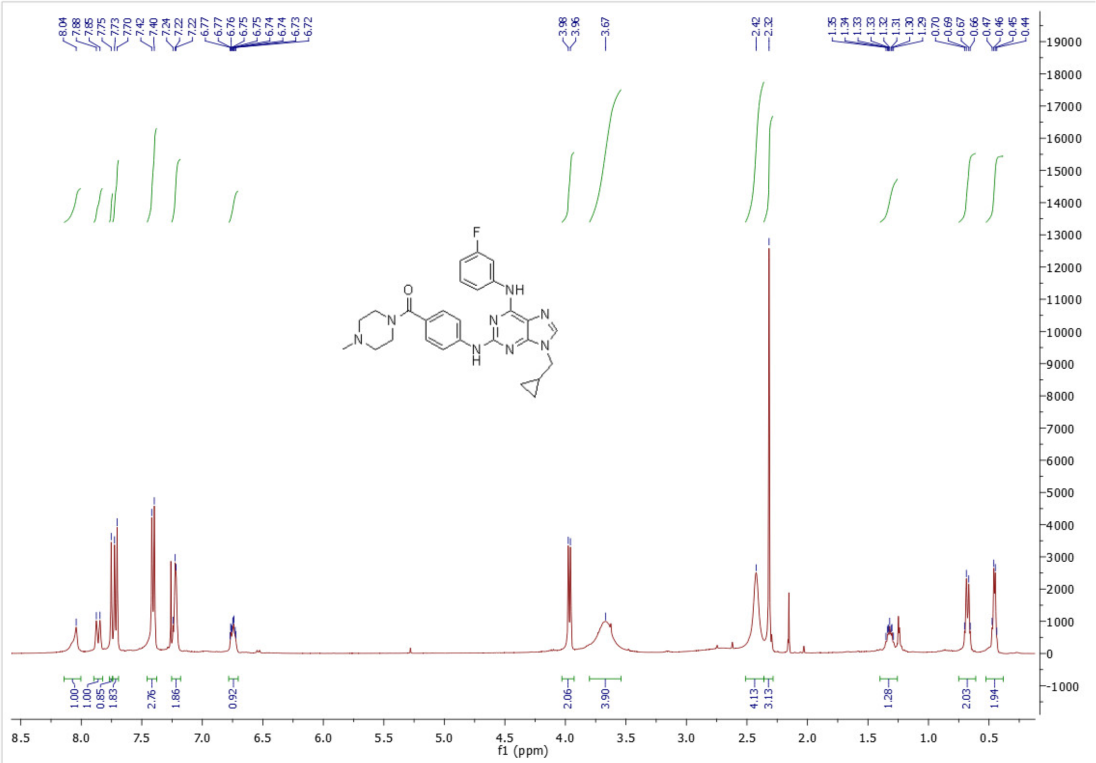

^{13}C NMR spectra of compound **10b**

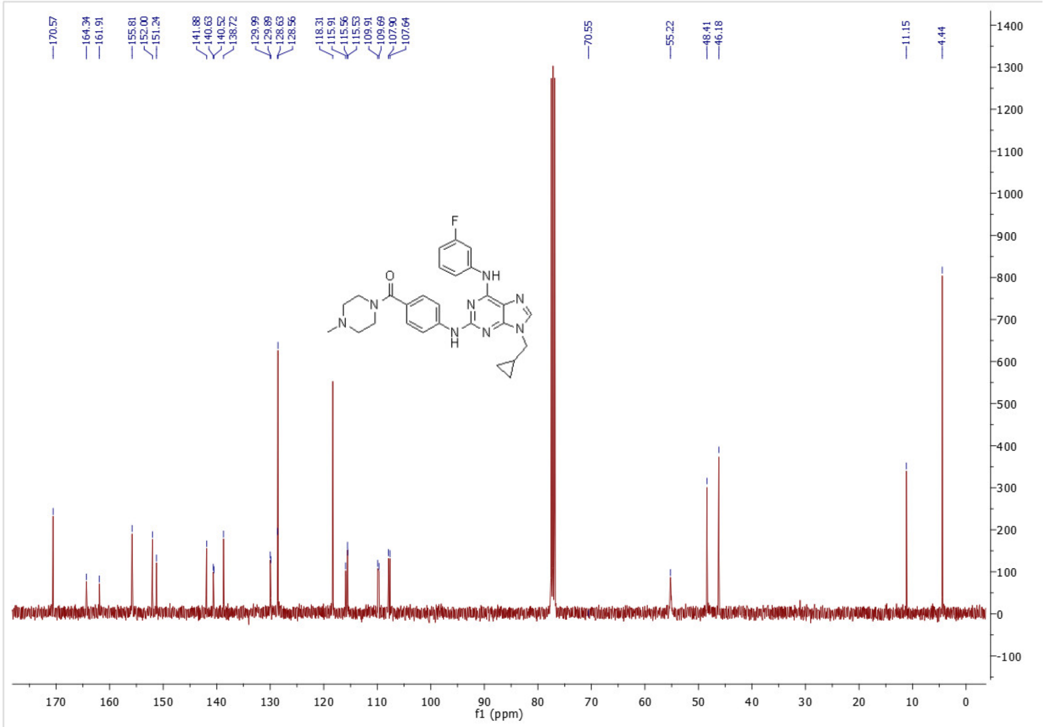

^{19}F NMR spectra of compound **10b**

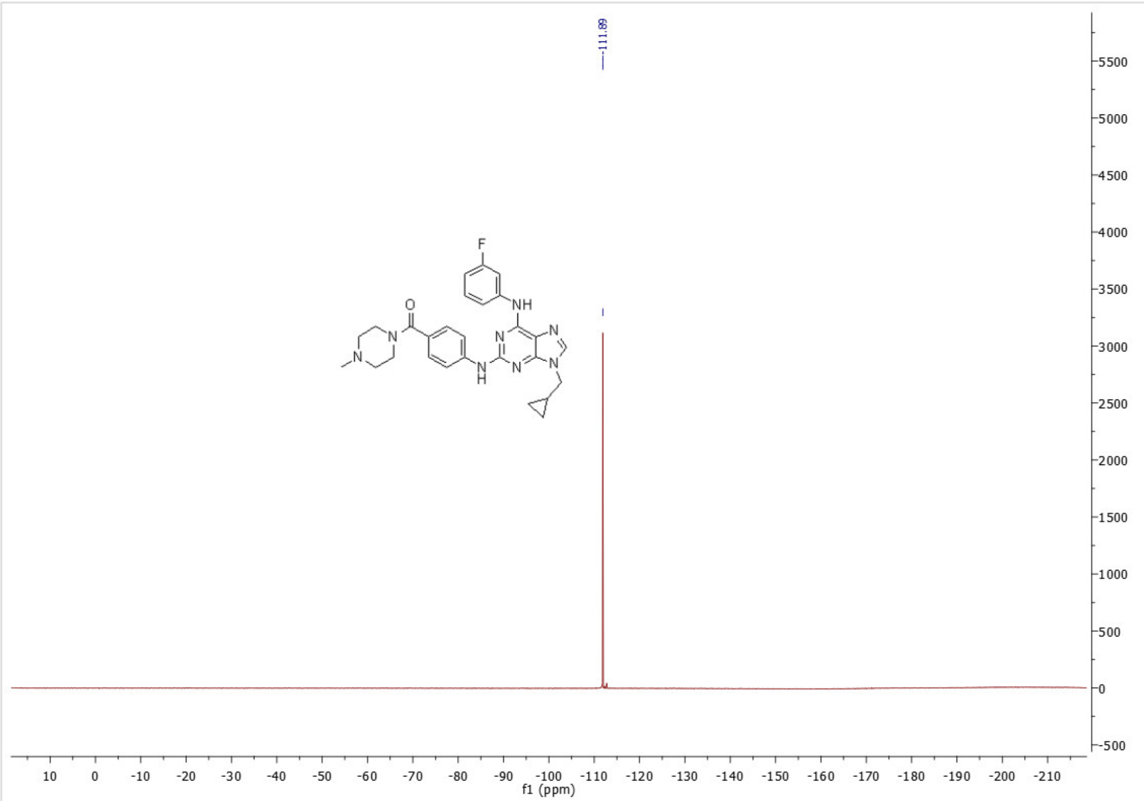

^{1}H NMR spectra of compound **10c**

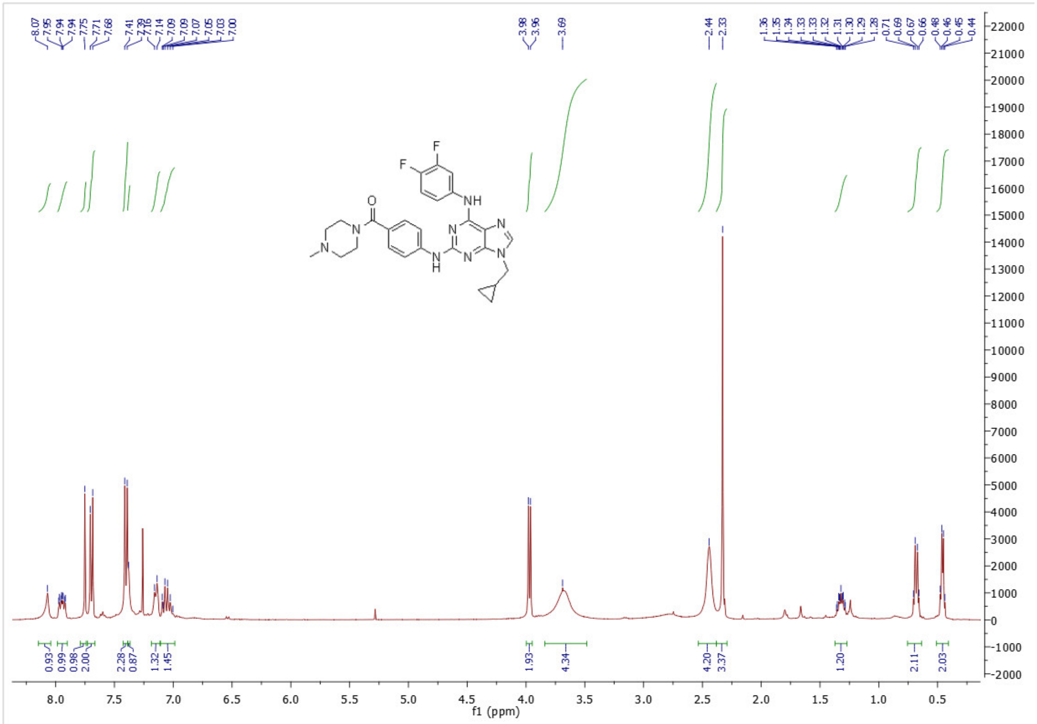

^{13}C NMR spectra of compound **10c**

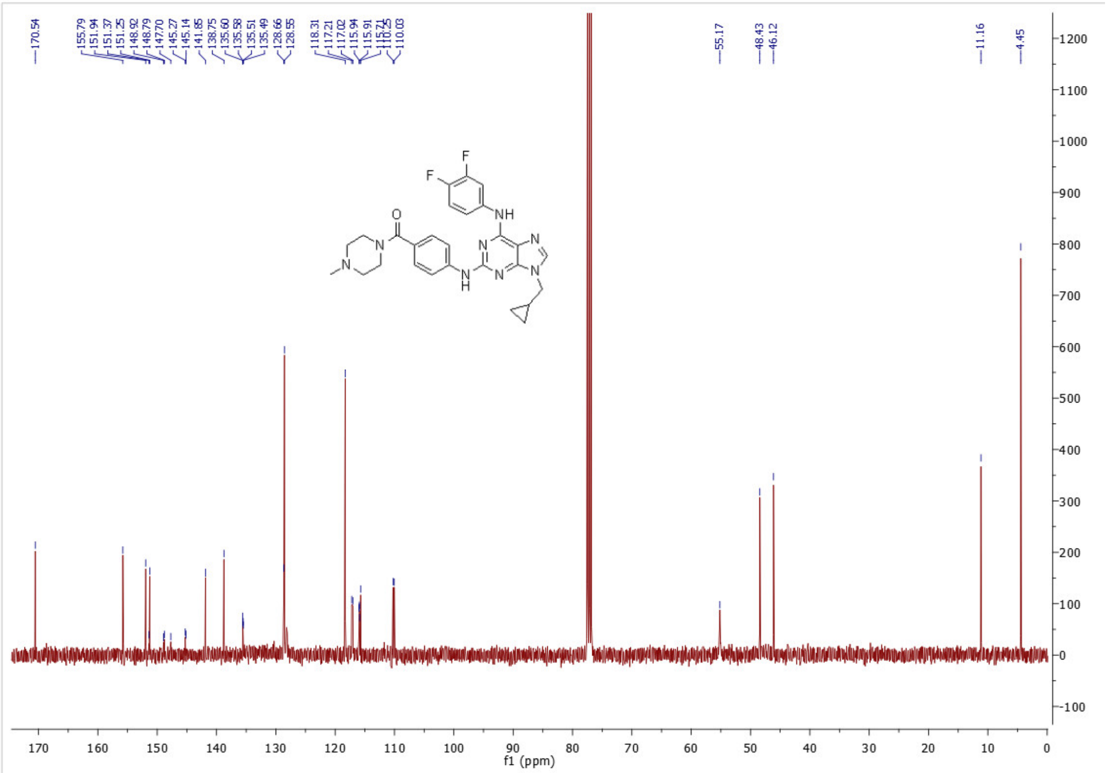

^{19}F NMR spectra of compound **10c**

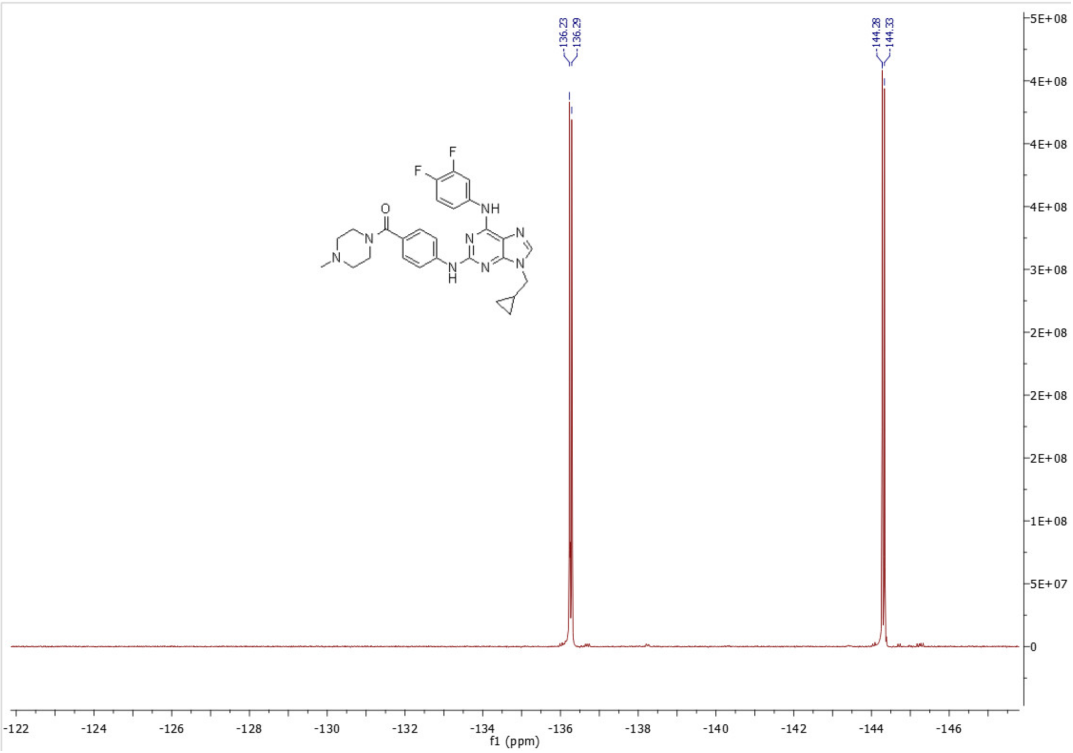

^{1}H NMR spectra of compound **11a**

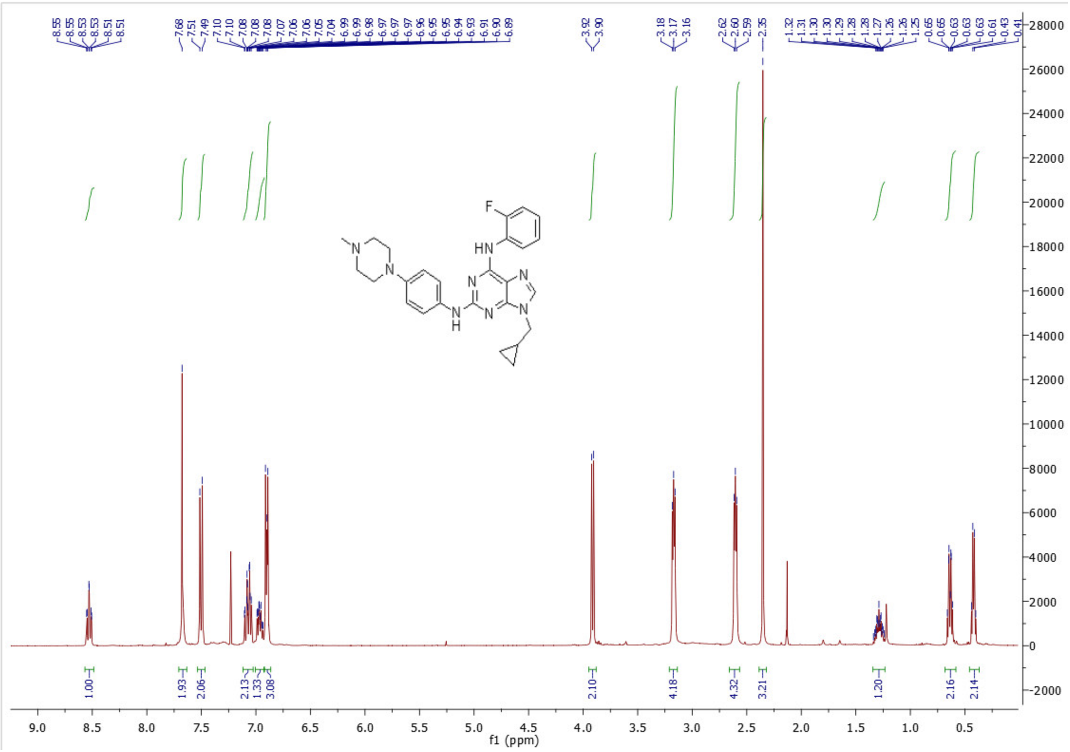

^{13}C NMR spectra of compound **11a**

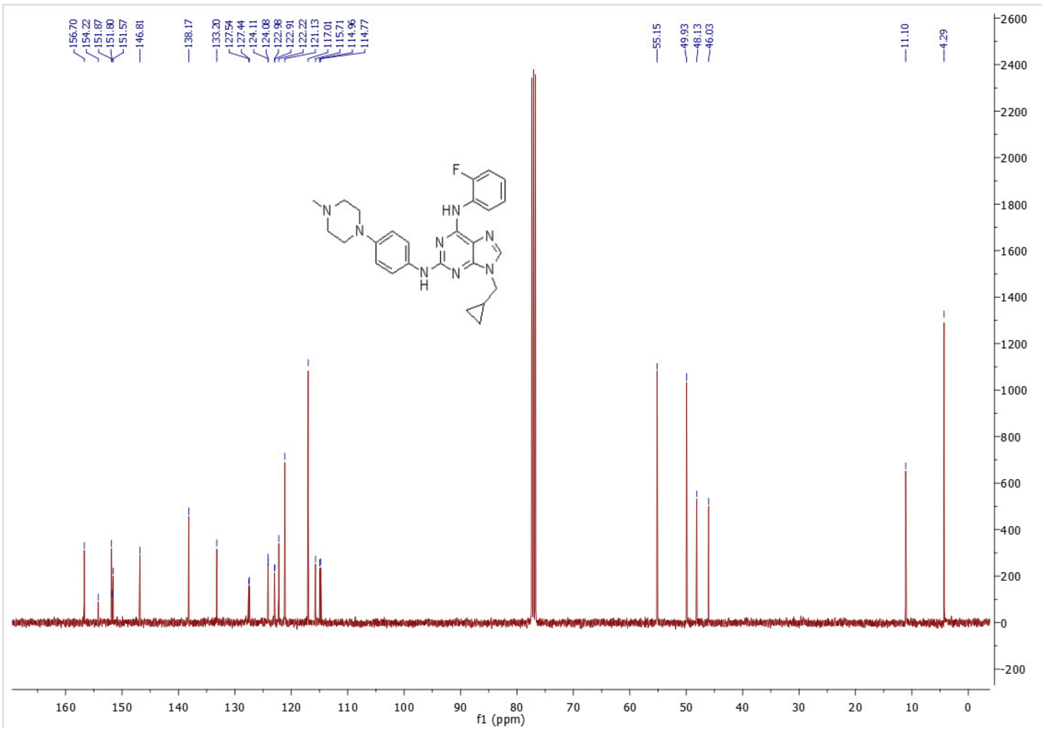

^{19}F NMR spectra of compound **11a**

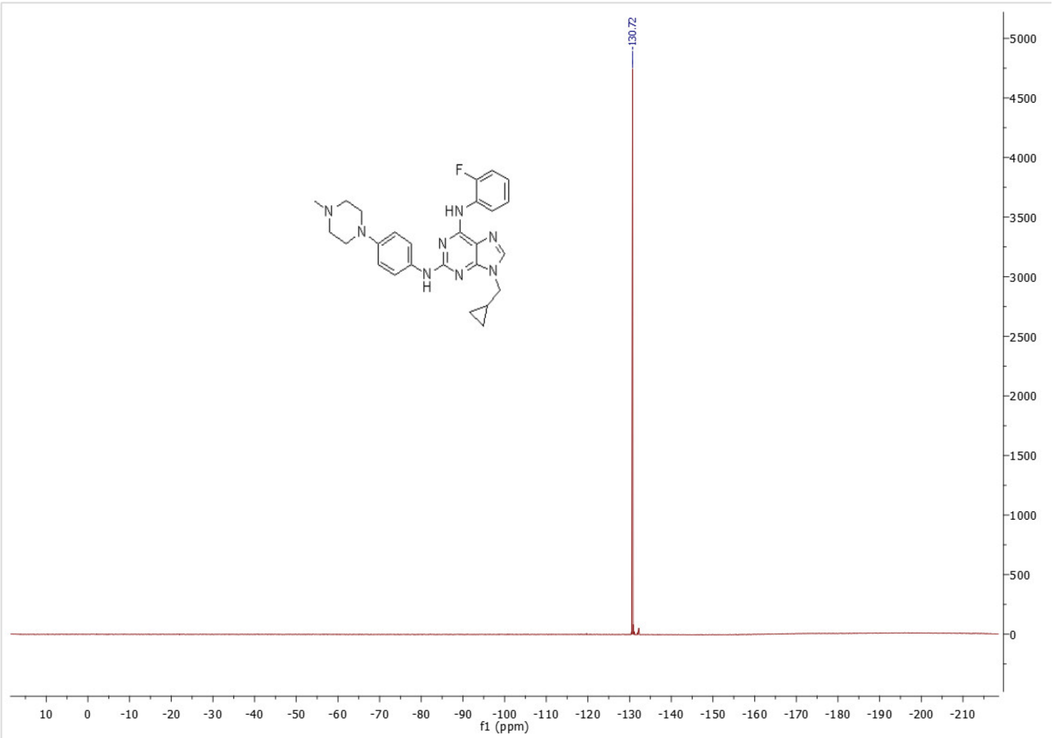

^{1}H NMR spectra of compound **11b**

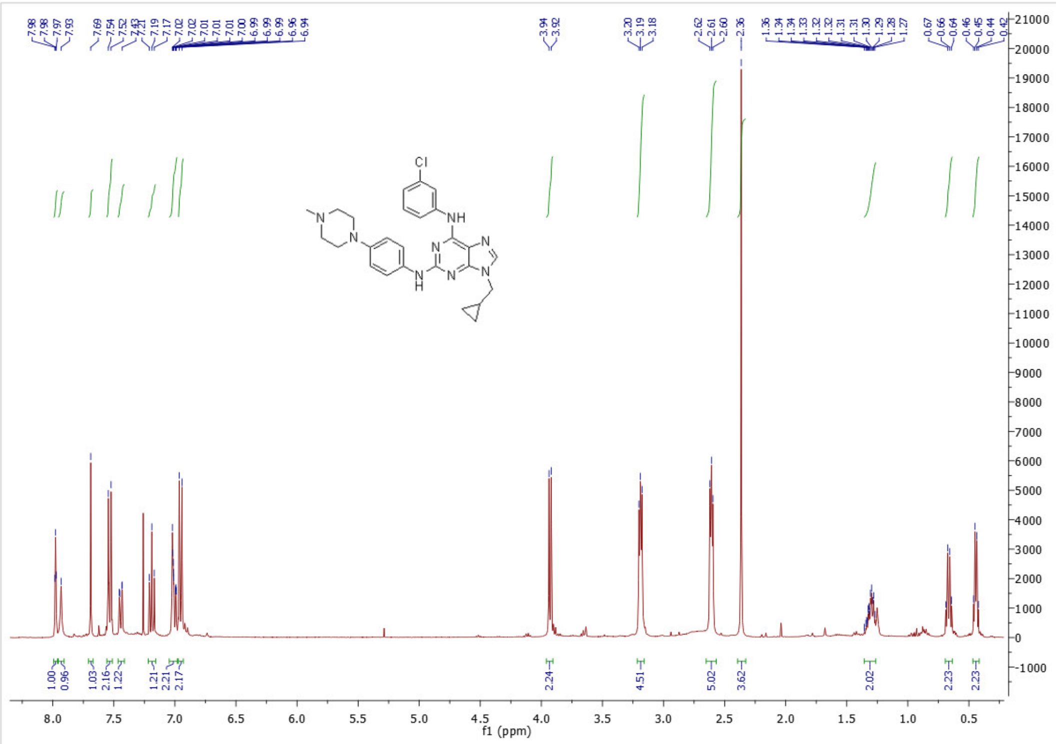

^{13}C NMR spectra of compound **11b**

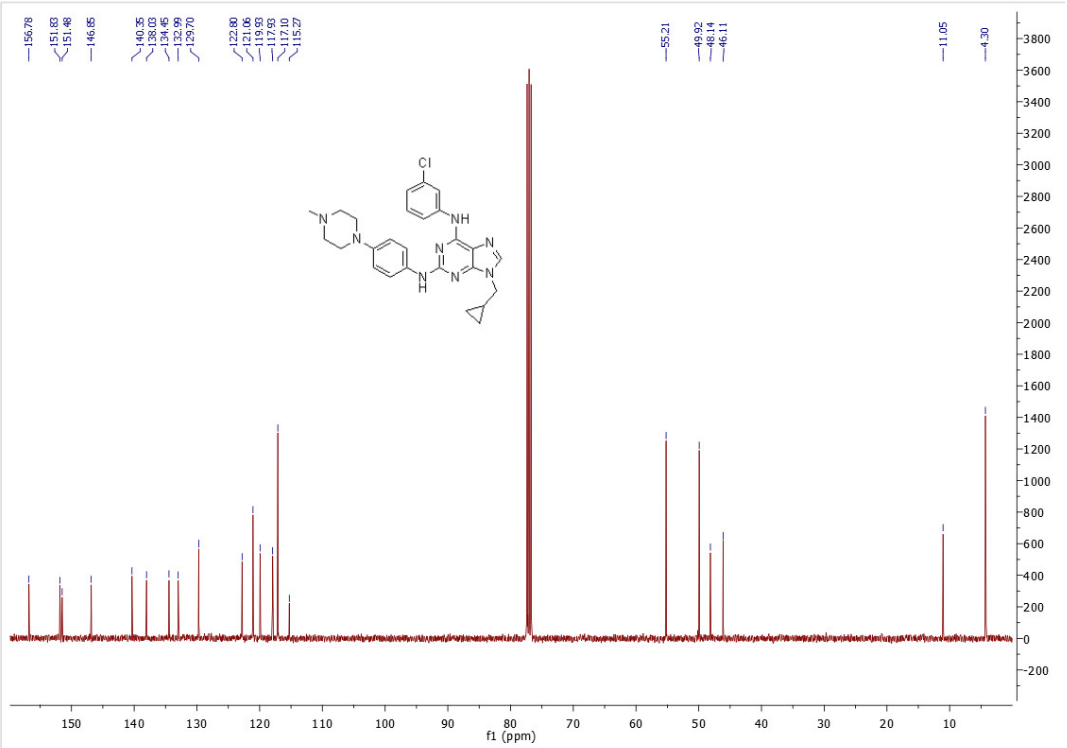

^{1}H NMR spectra of compound **11c**

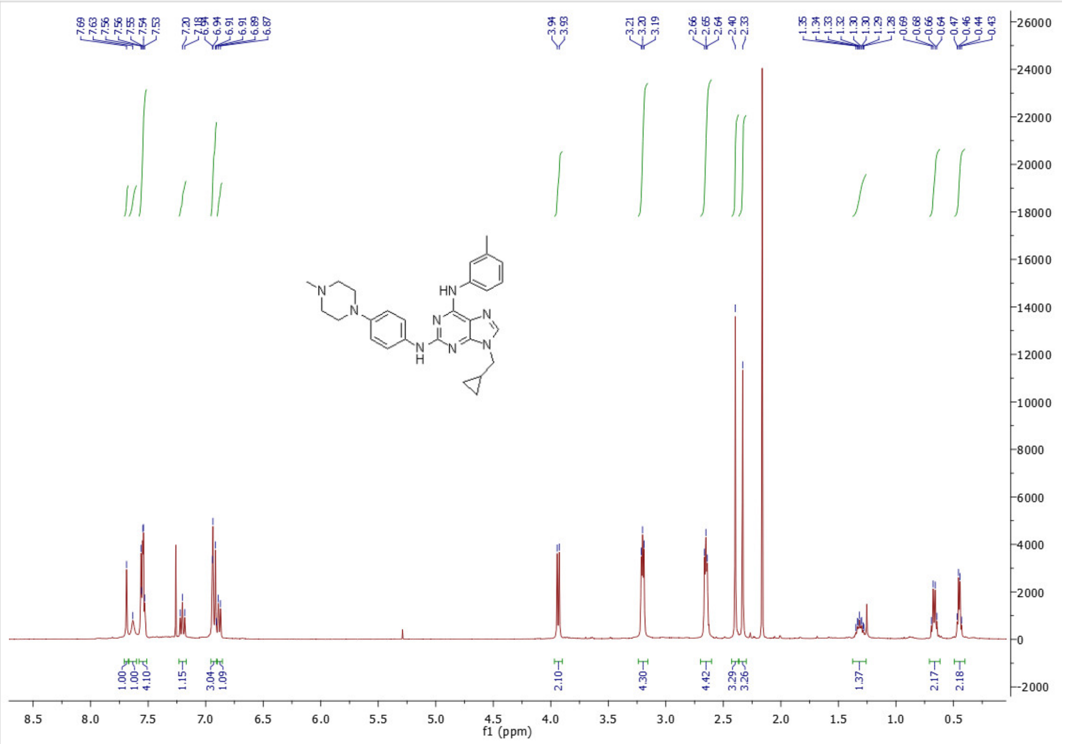

^{13}C NMR spectra of compound **11c**

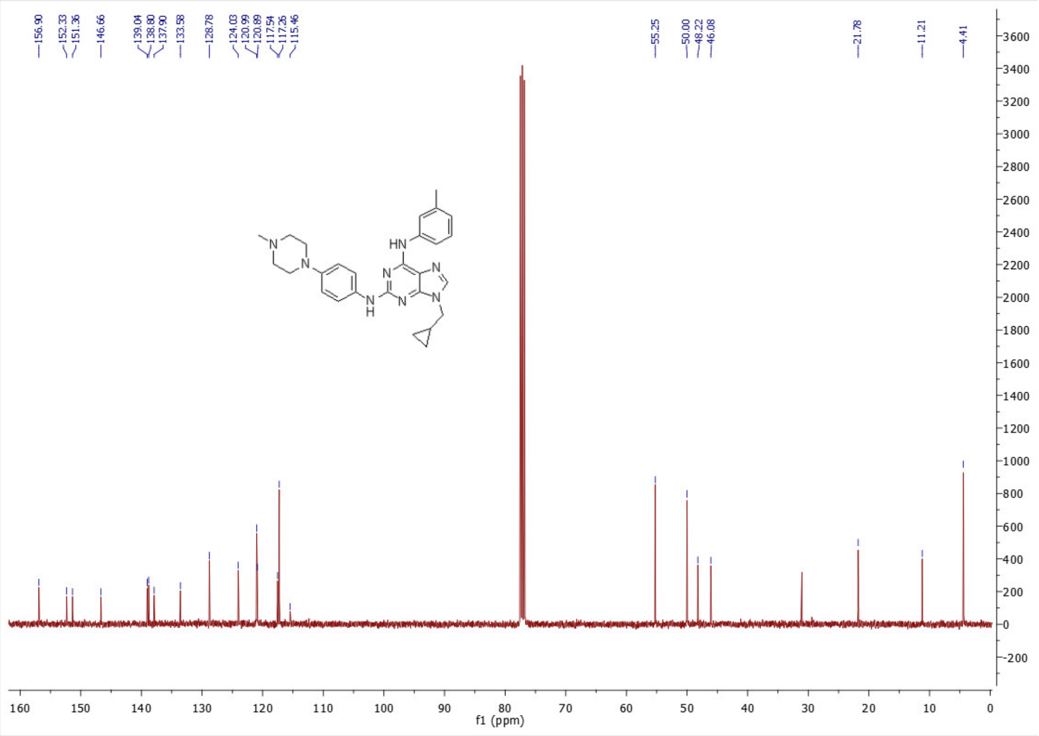

^{1}H NMR spectra of compound **11d**

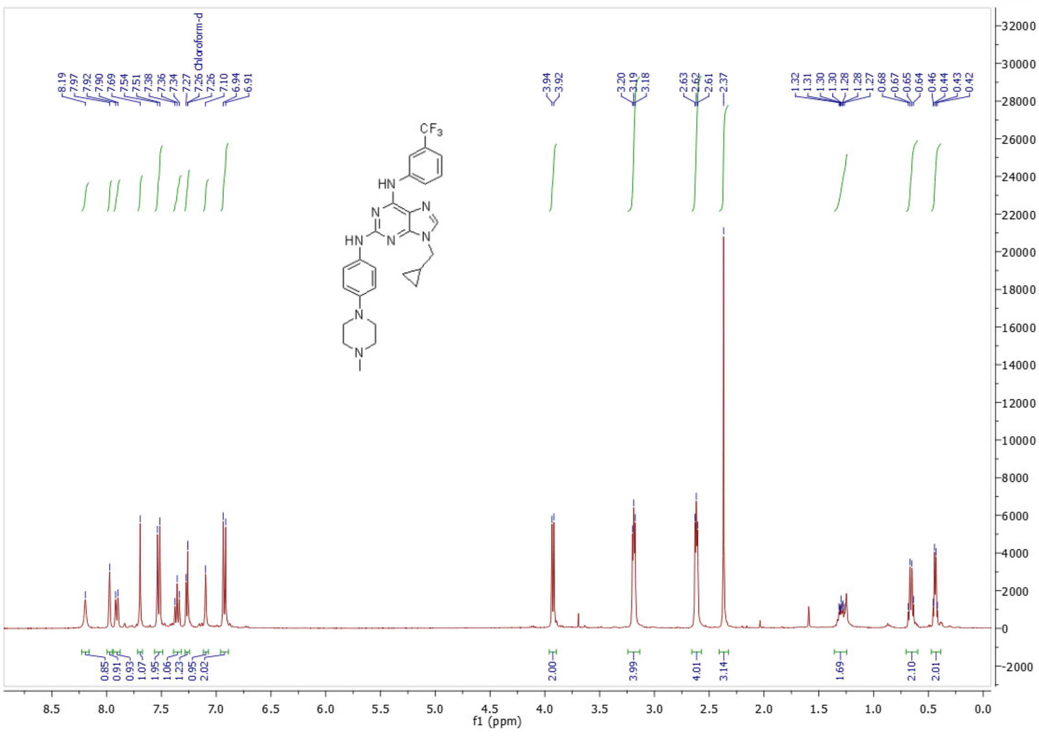

^{13}C NMR spectra of compound **11d**

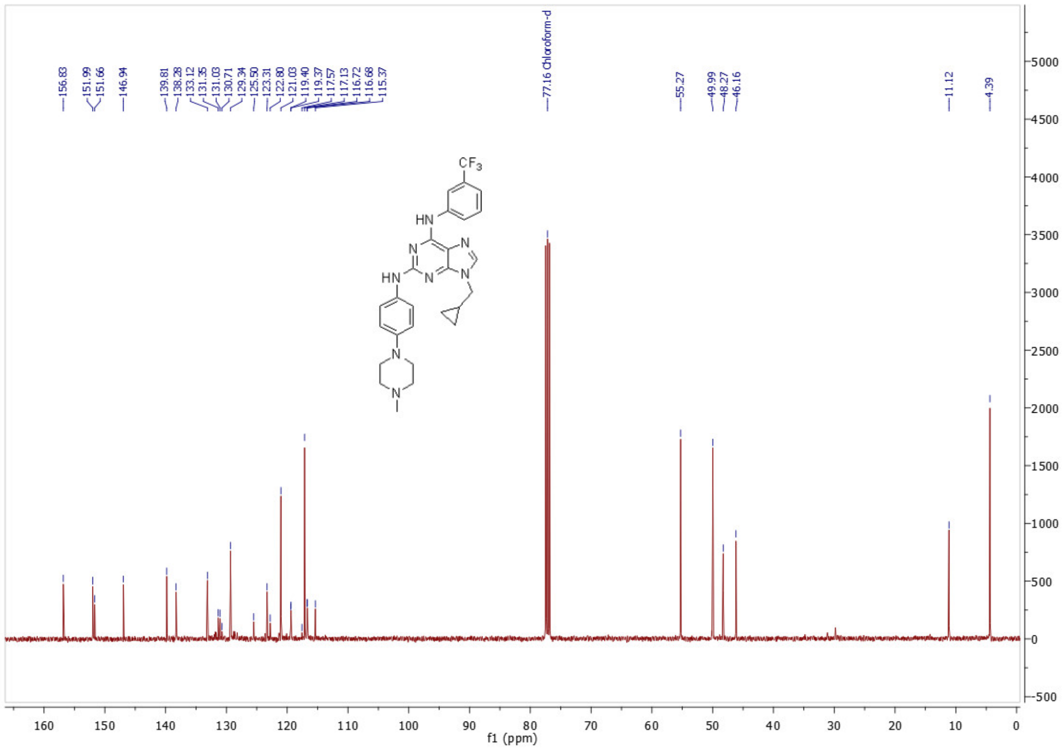

^{19}F NMR spectra of compound **11d**

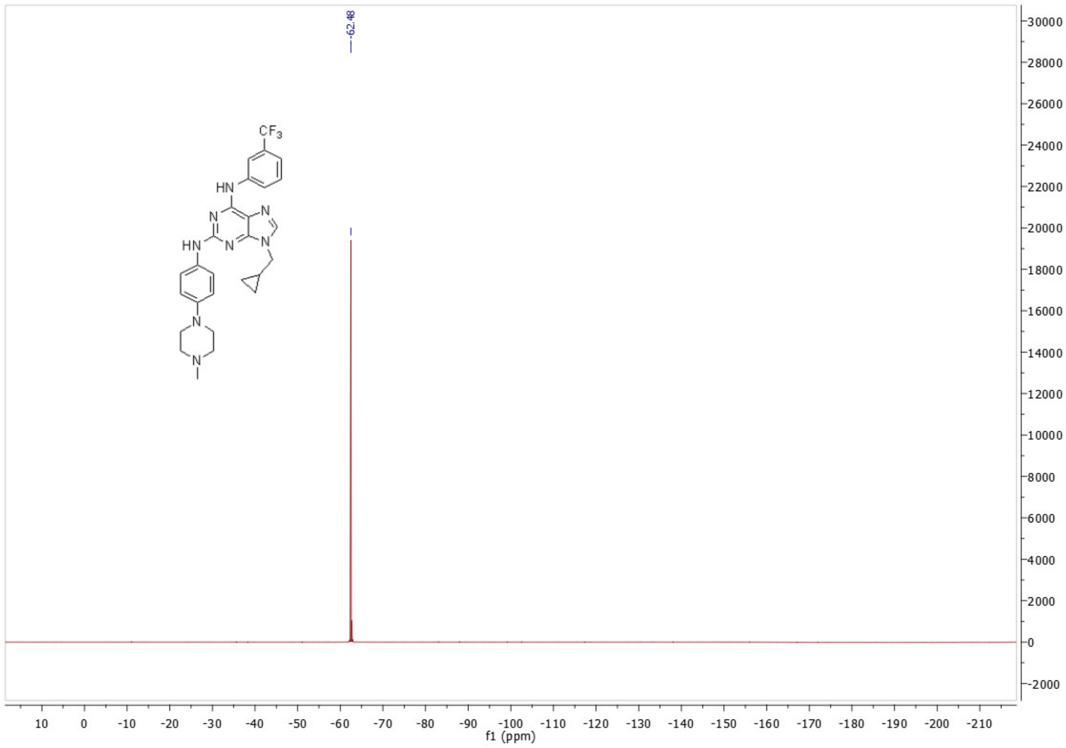

^{1}H NMR spectra of compound **11e**

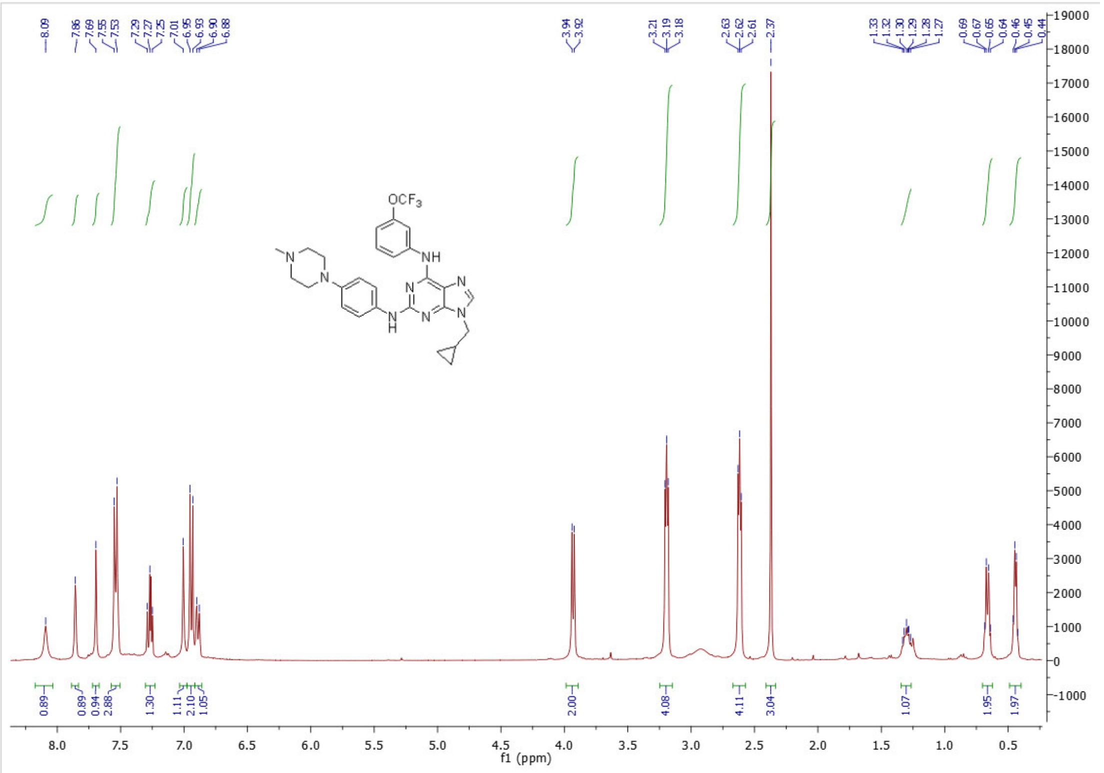

^{13}C NMR spectra of compound **11e**

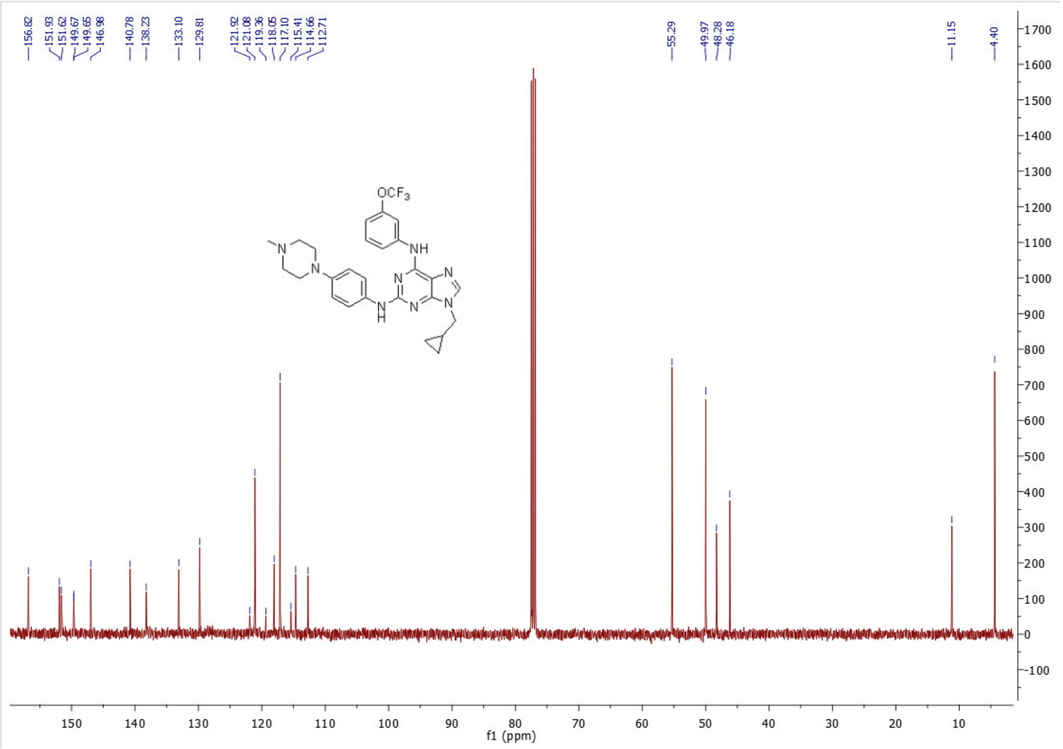

^{19}F NMR spectra of compound **11e**

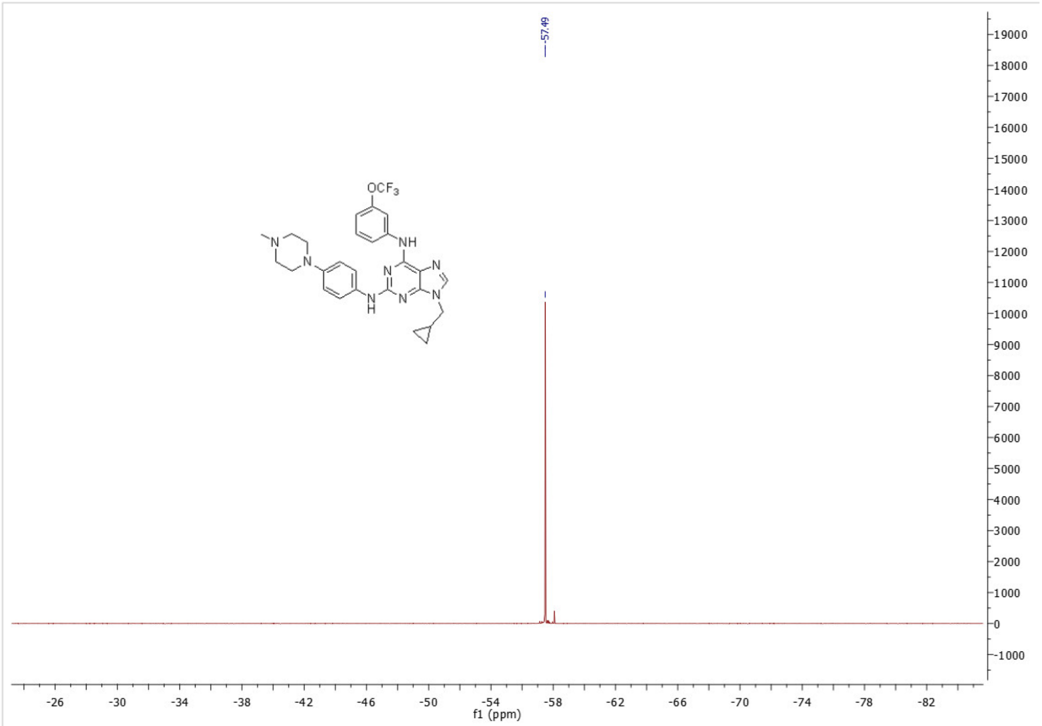

^{1}H NMR spectra of compound **11f**

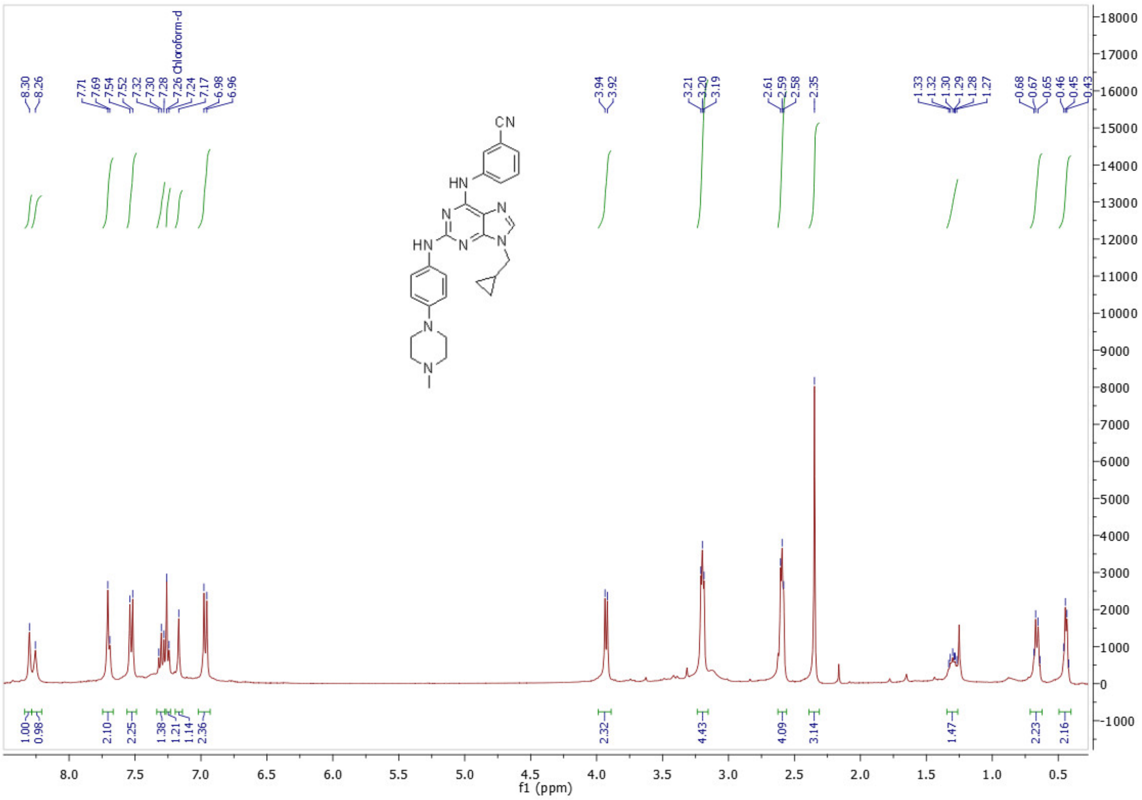

^{13}C NMR spectra of compound **11f**

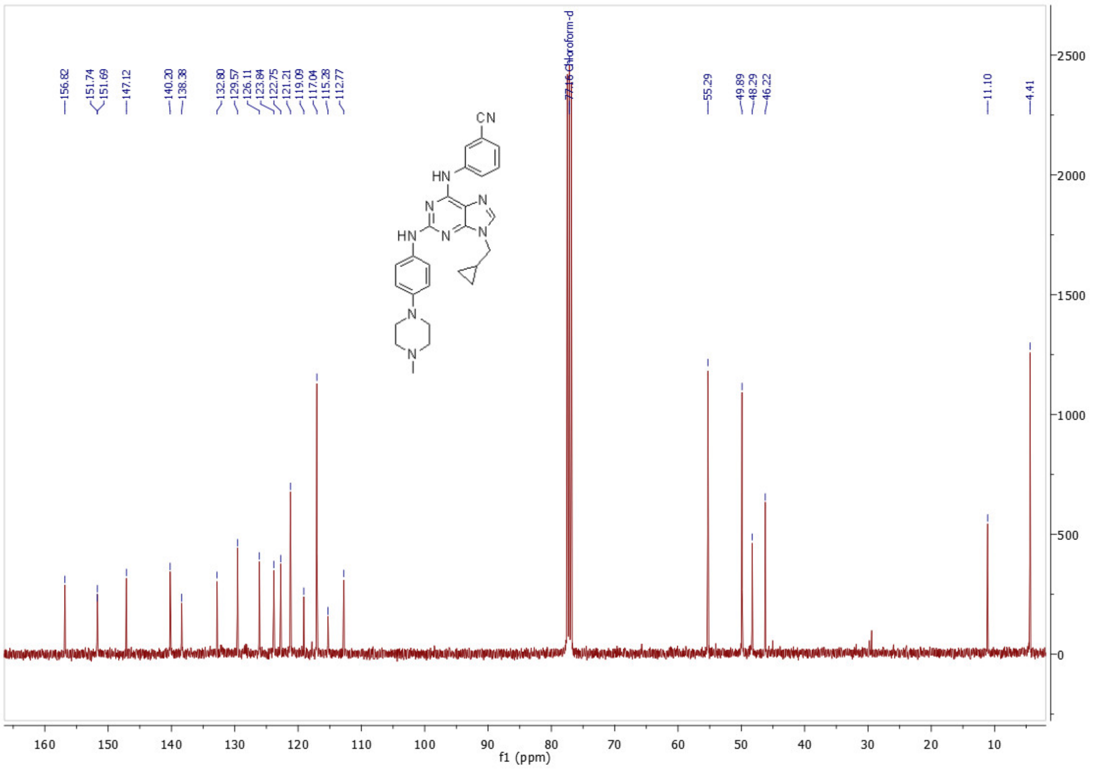

^{1}H NMR spectra of compound **11g**

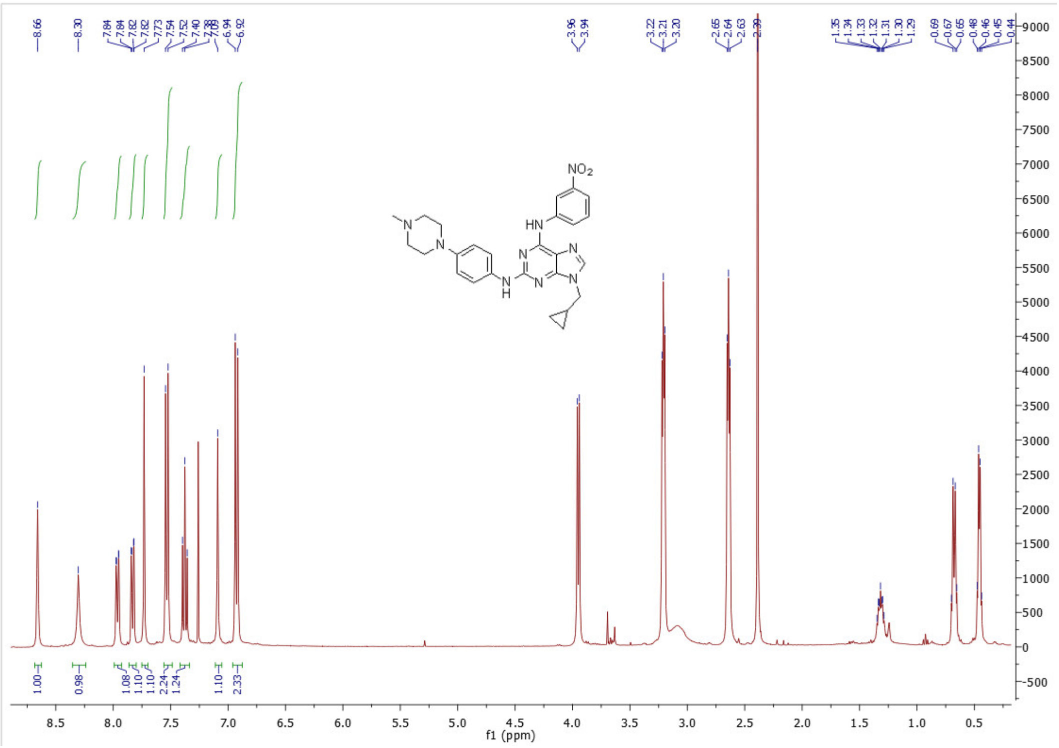

^{13}C NMR spectra of compound **11g**

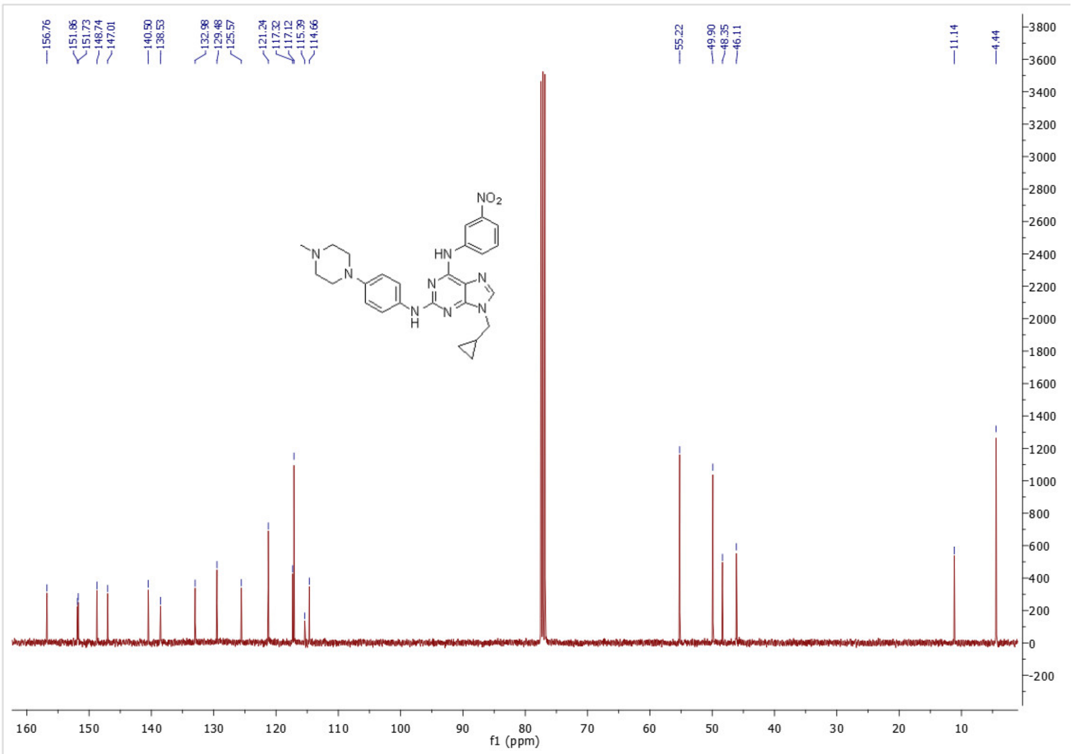

^{1}H NMR spectra of compound **11h**

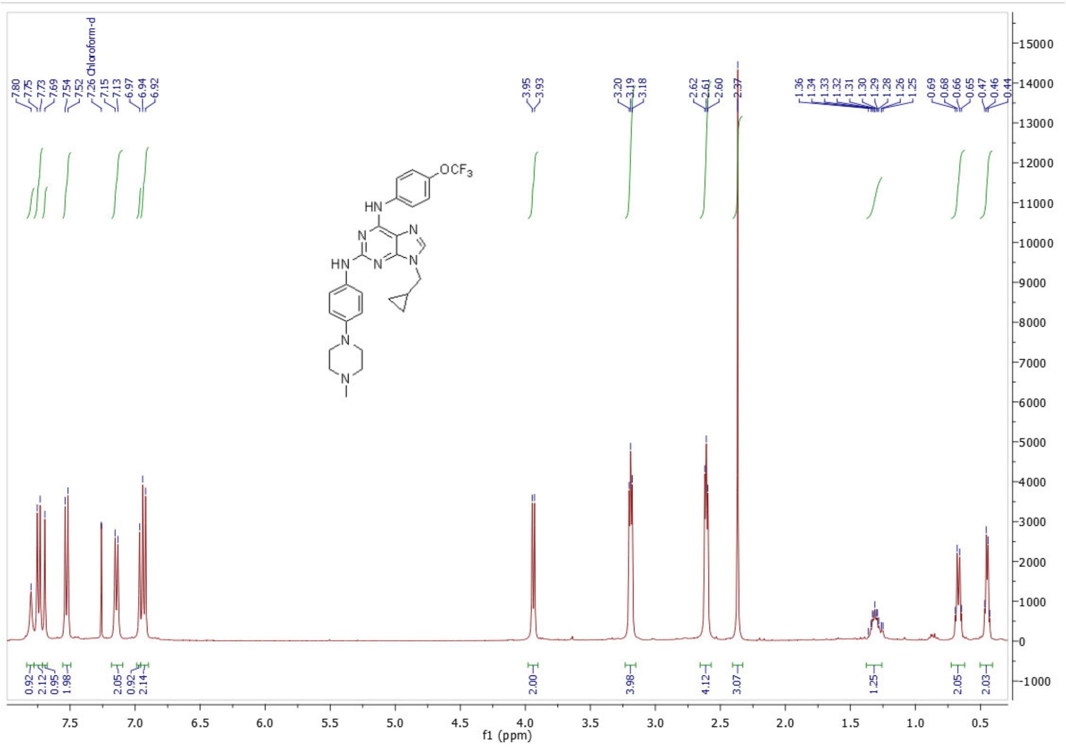

^{13}C NMR spectra of compound **11h**

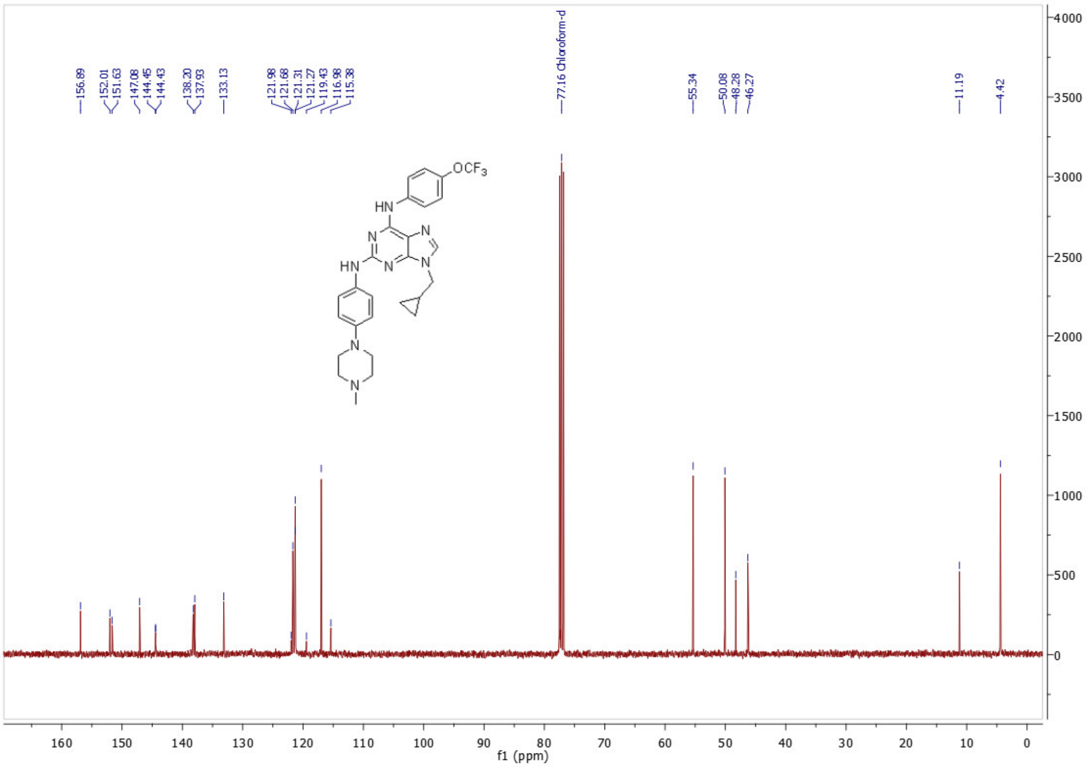

^{19}F NMR spectra of compound **11h**

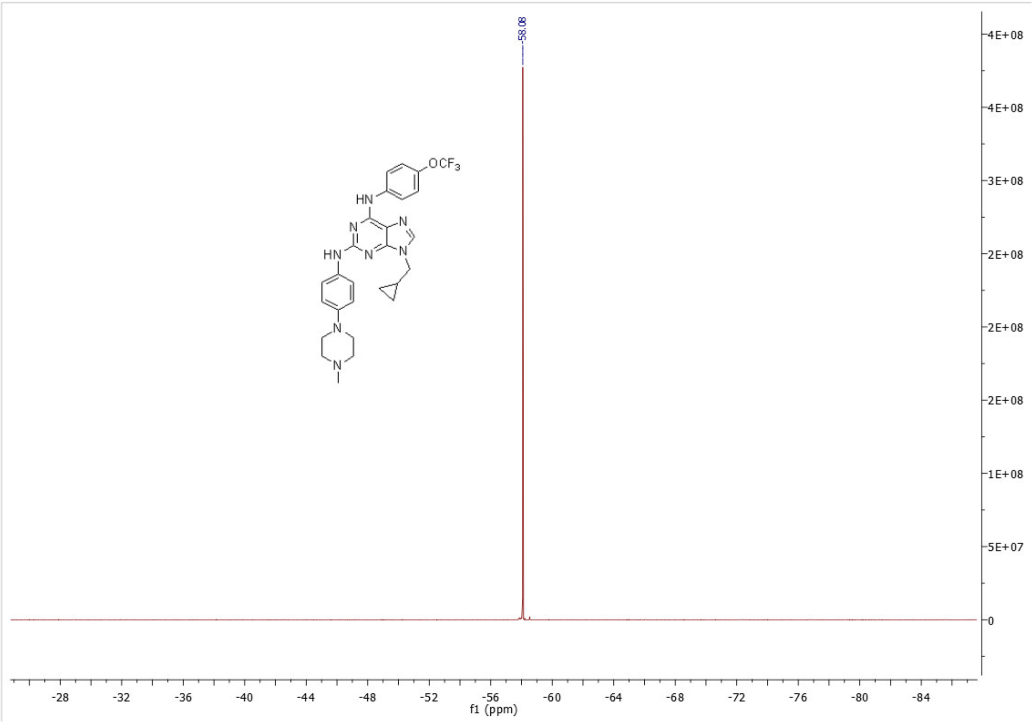

^{1}H NMR spectra of compound **11i**

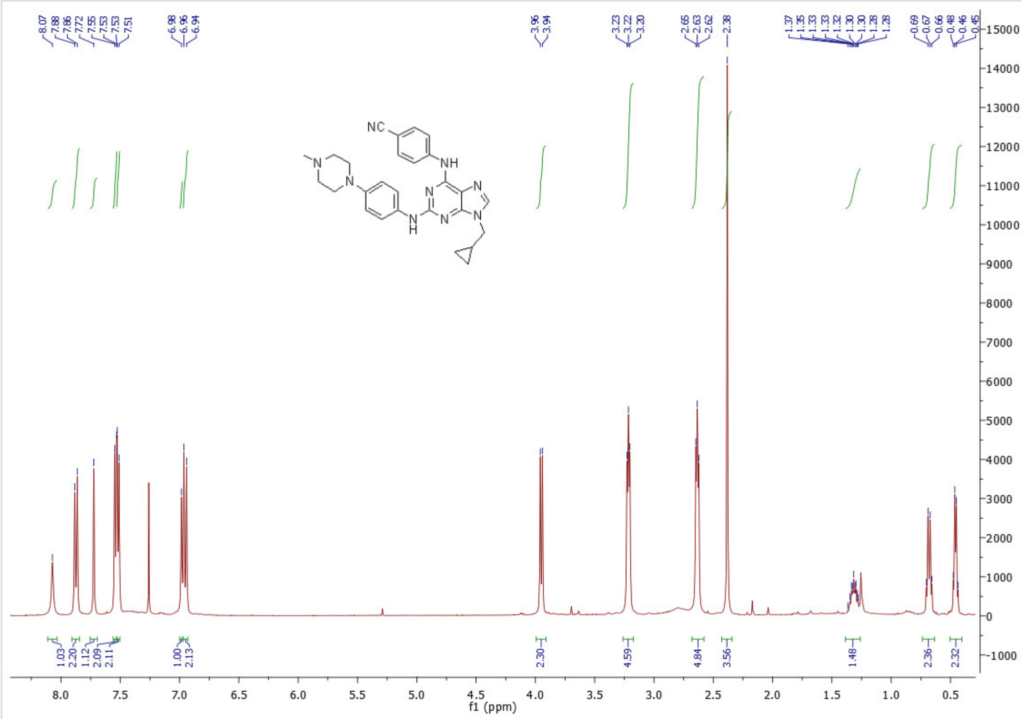

^{13}C NMR spectra of compound **11i**

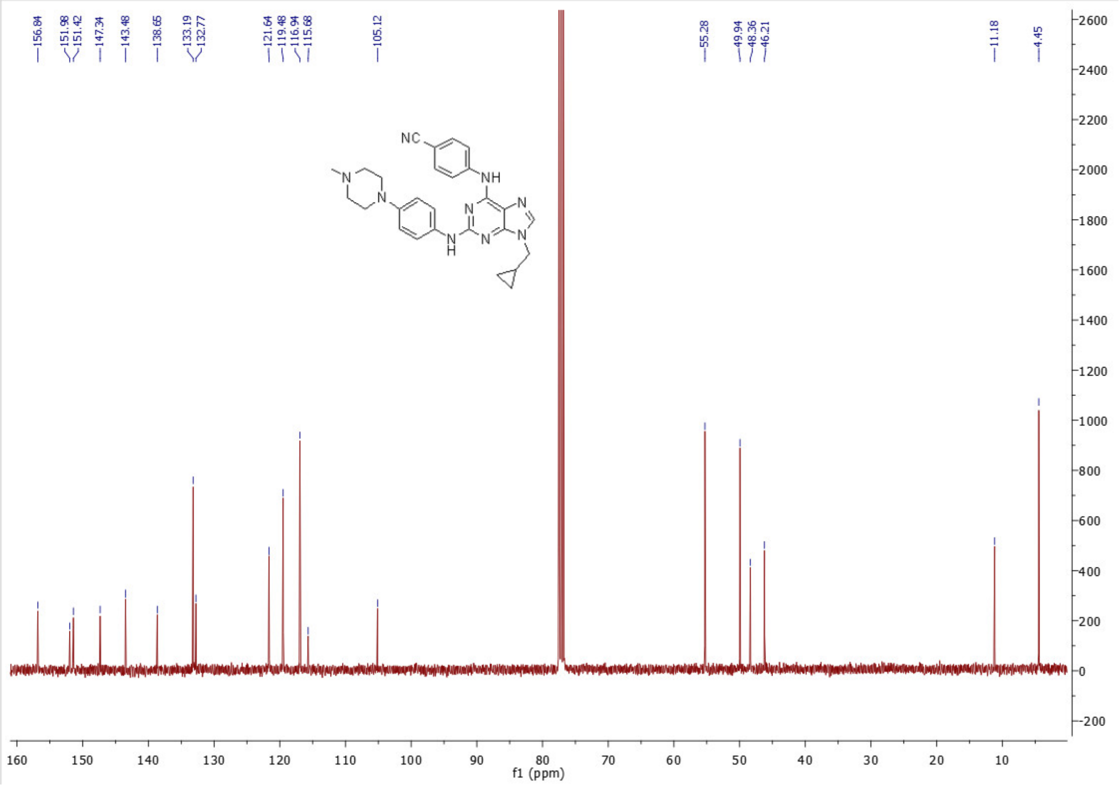

^{1}H NMR spectra of compound **11j**

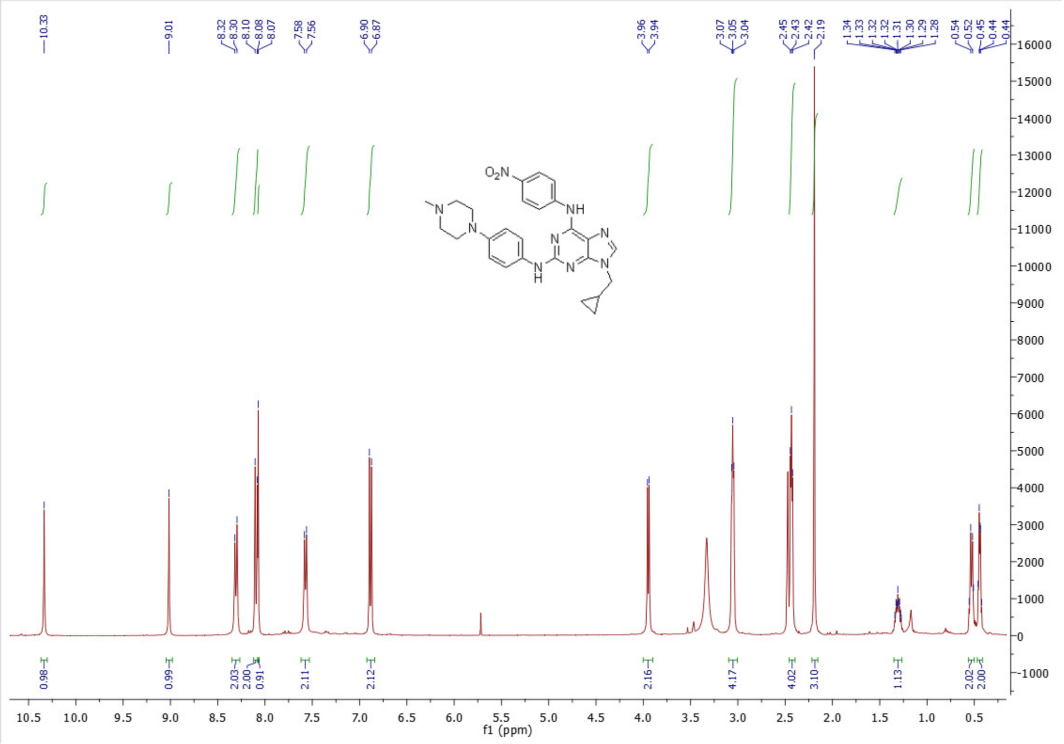

^{13}C NMR spectra of compound **11j**

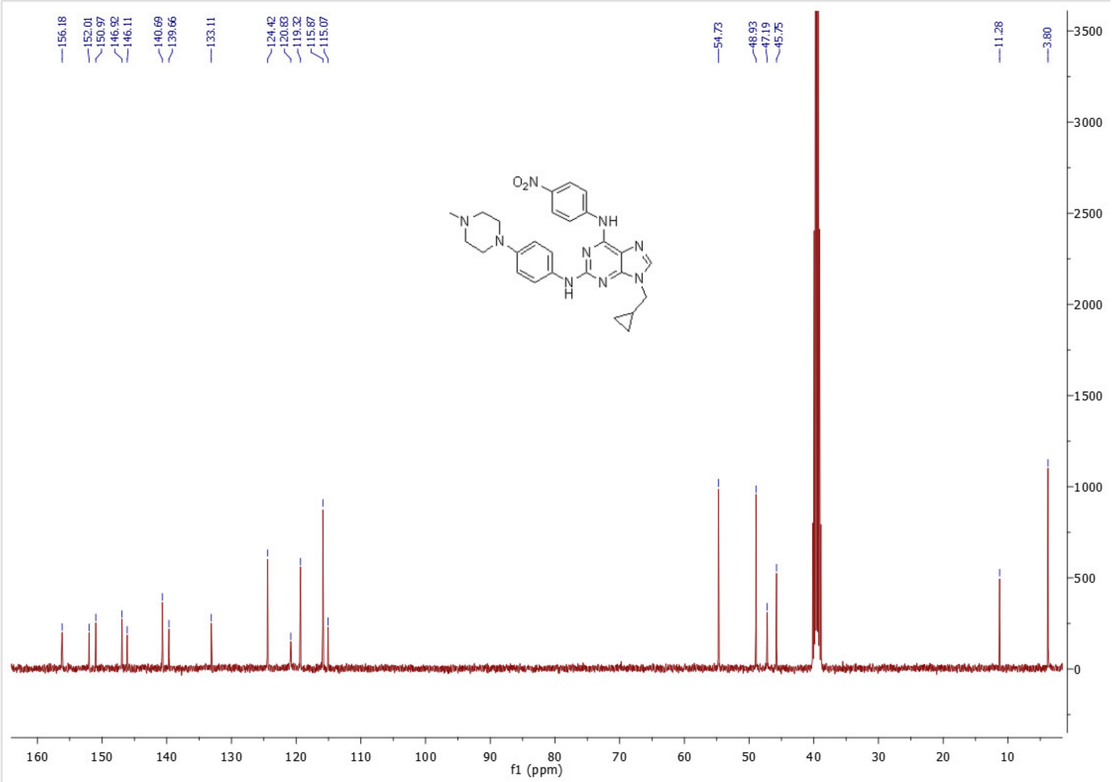

^{1}H NMR spectra of compound **11k**

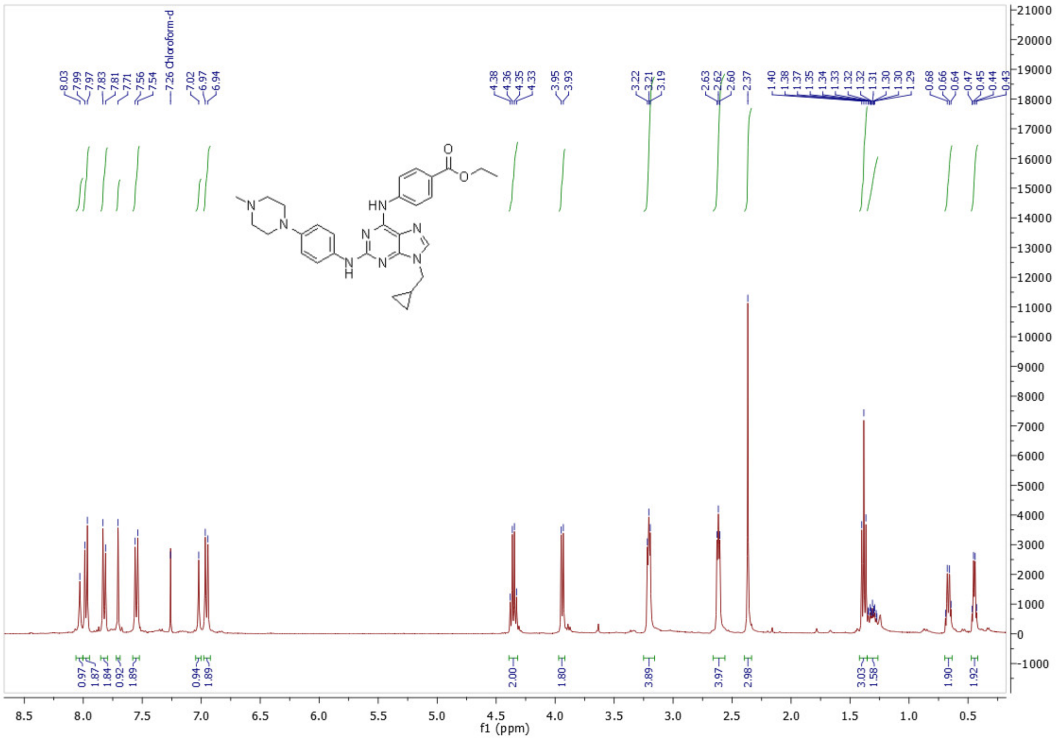

^{13}C NMR spectra of compound **11k**

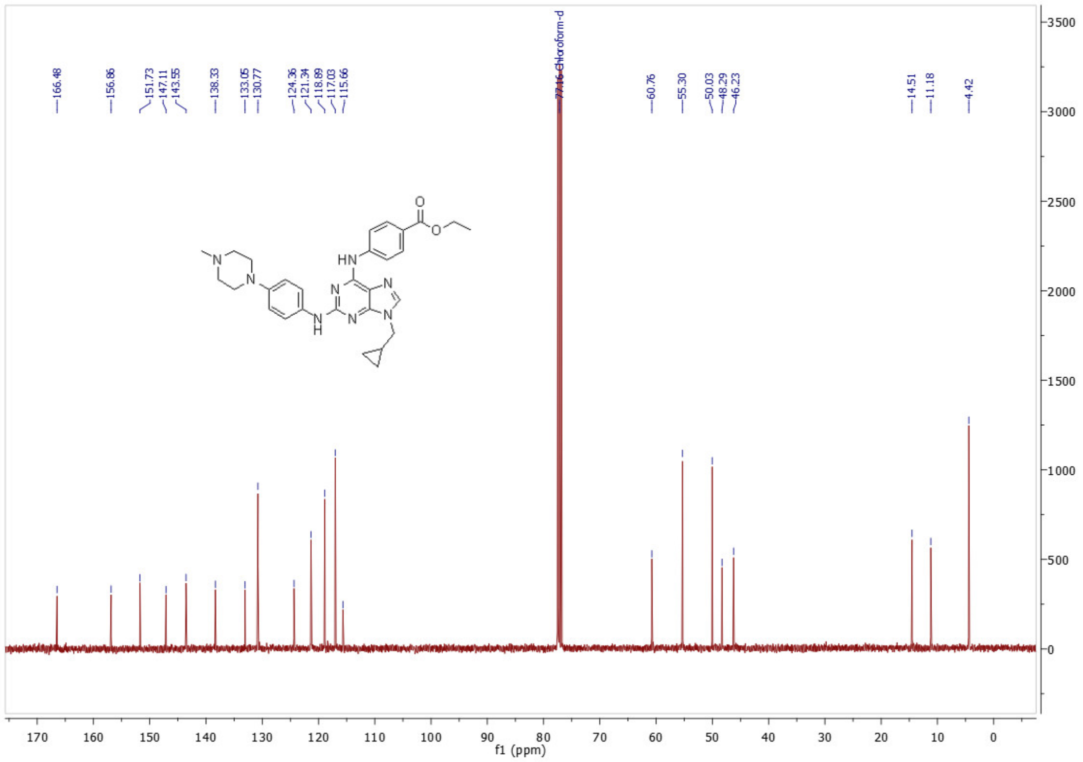

^{1}H NMR spectra of compound **11l**

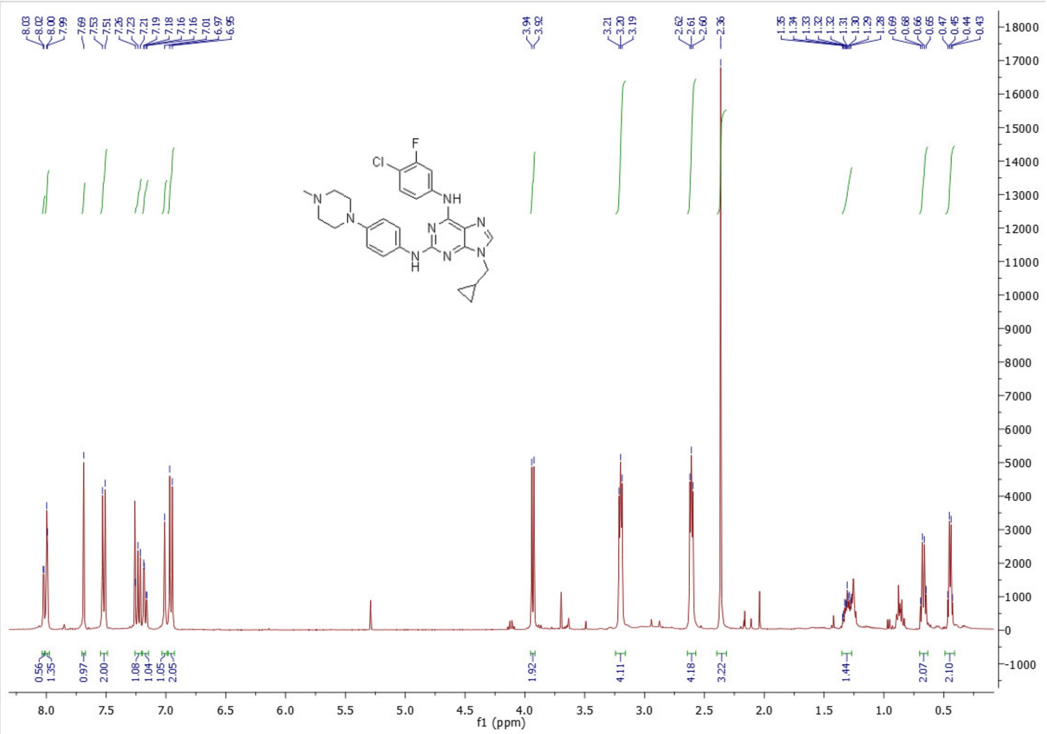

^{13}C NMR spectra of compound **11l**

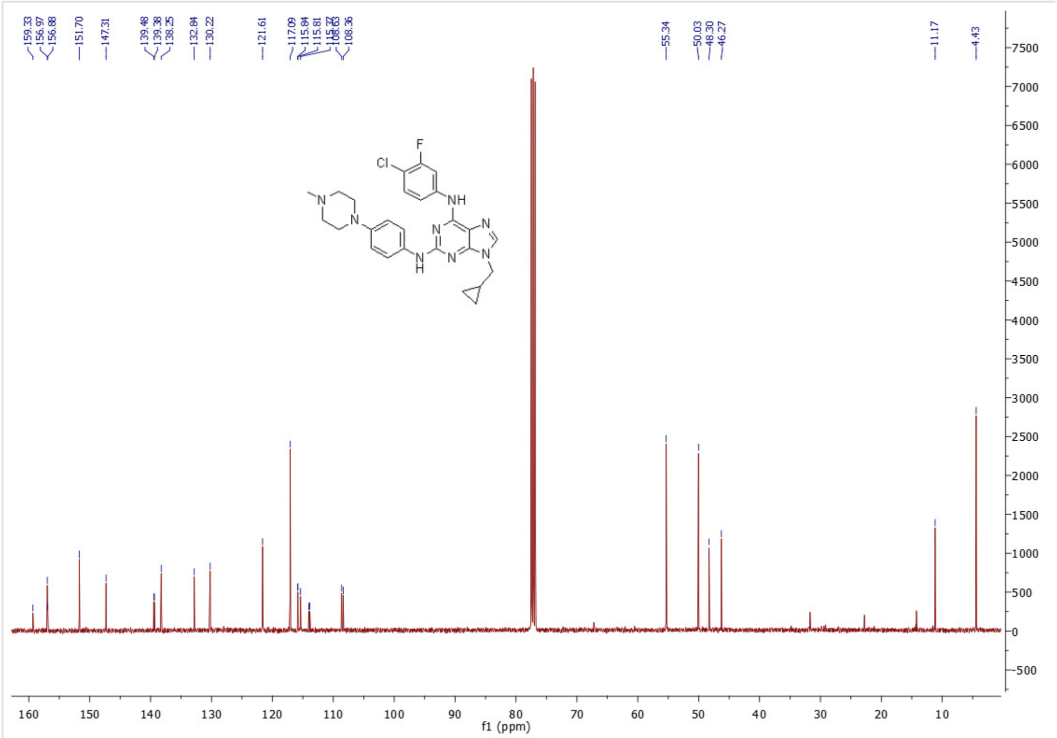

^{19}F NMR spectra of compound **11l**

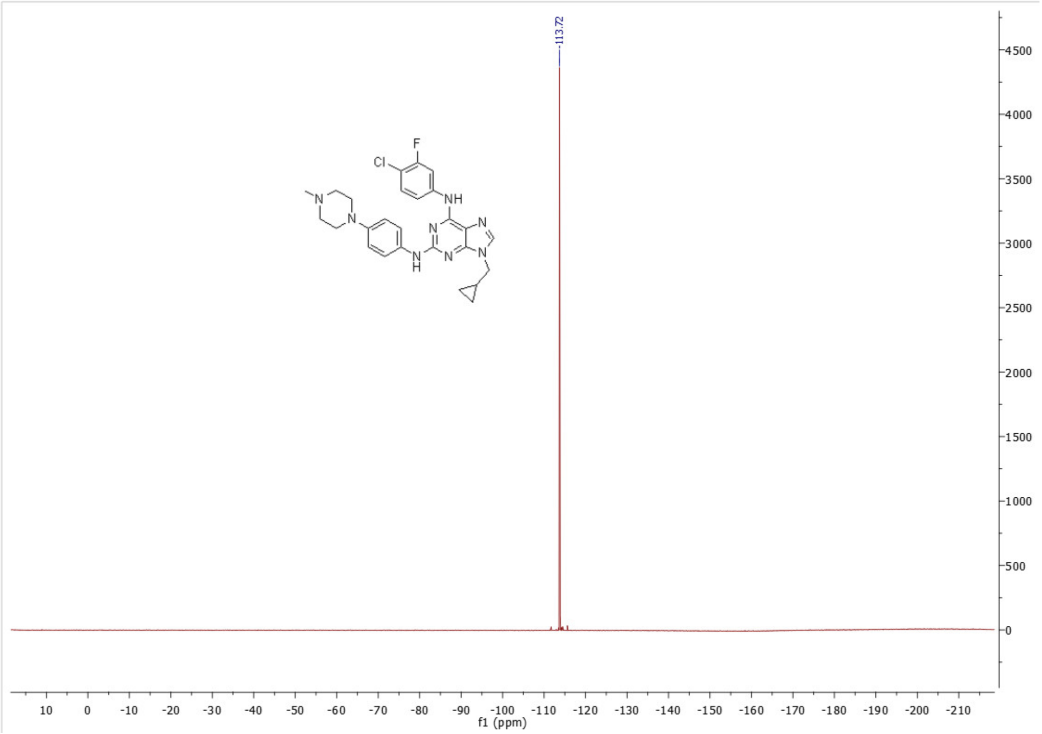

^{1}H NMR spectra of compound **11m**

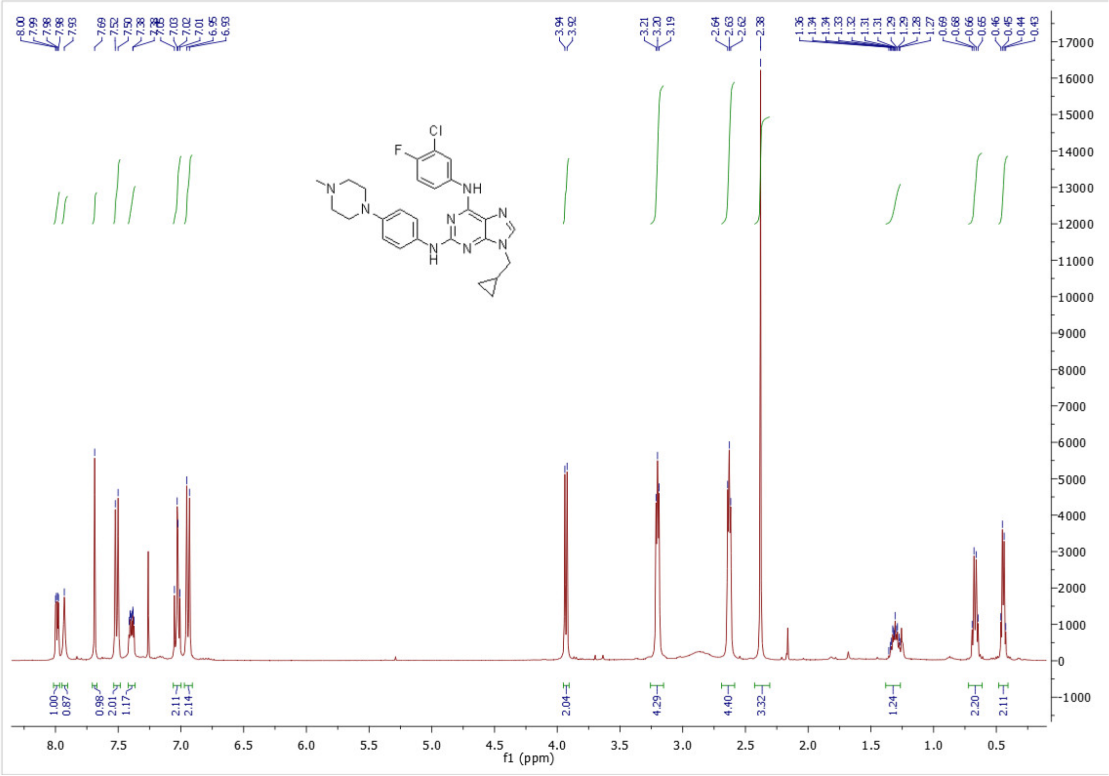

^{13}C NMR spectra of compound **11m**

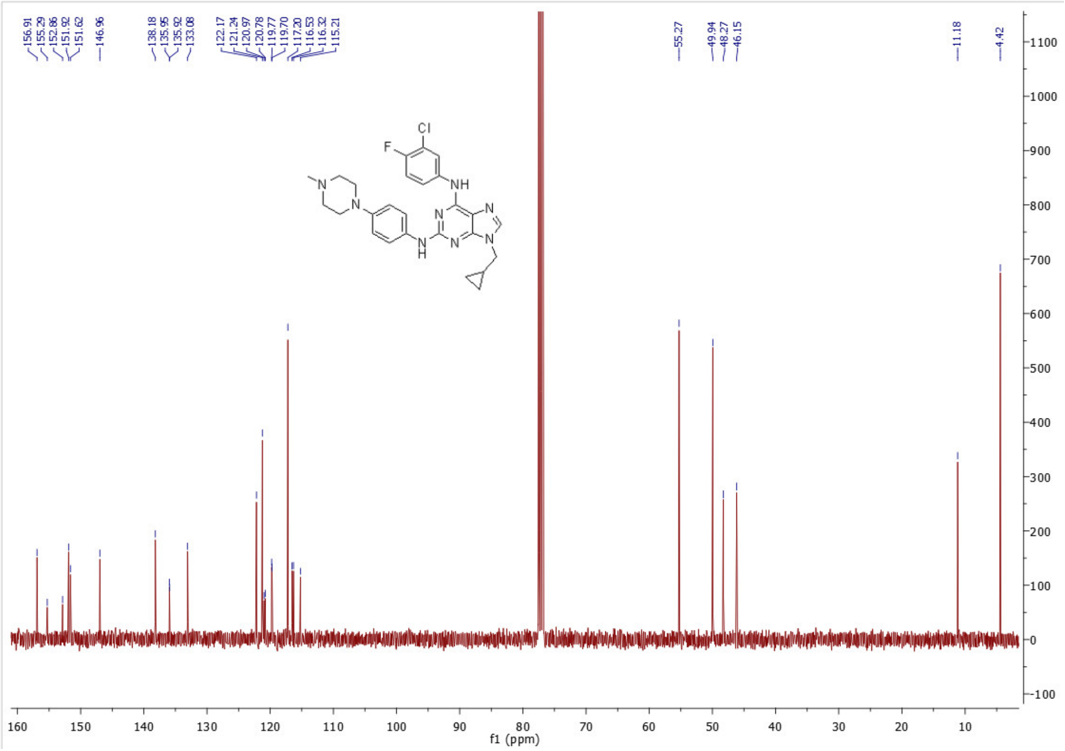

^{19}F NMR spectra of compound **11m**

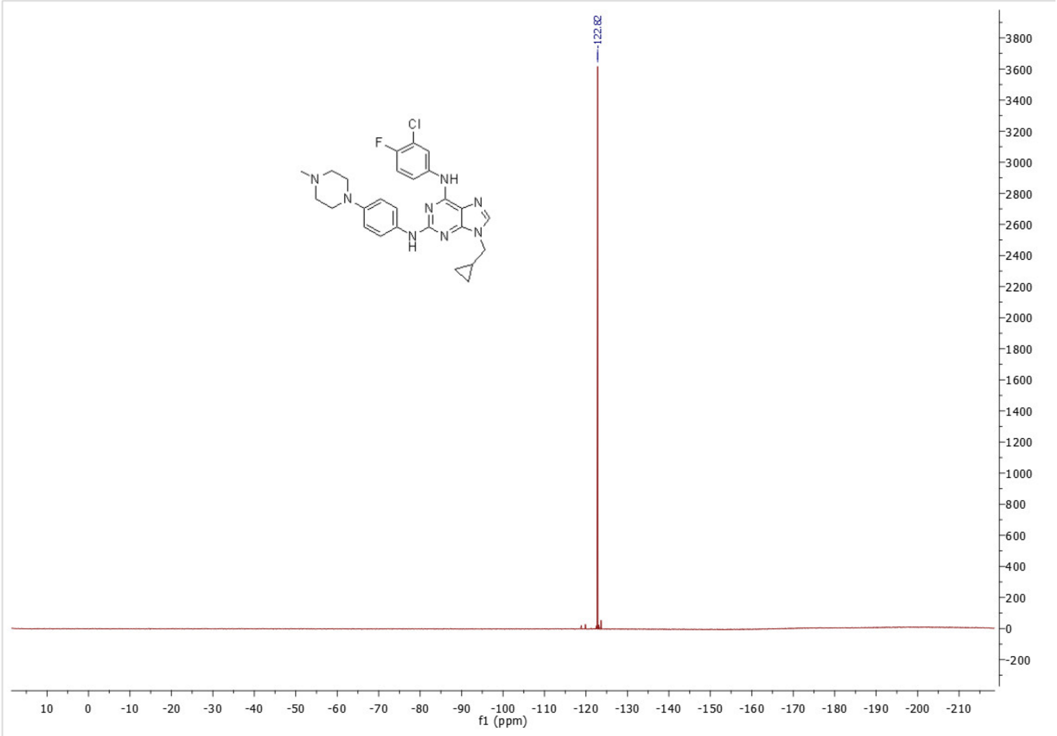

^{1}H NMR spectra of compound **11n**

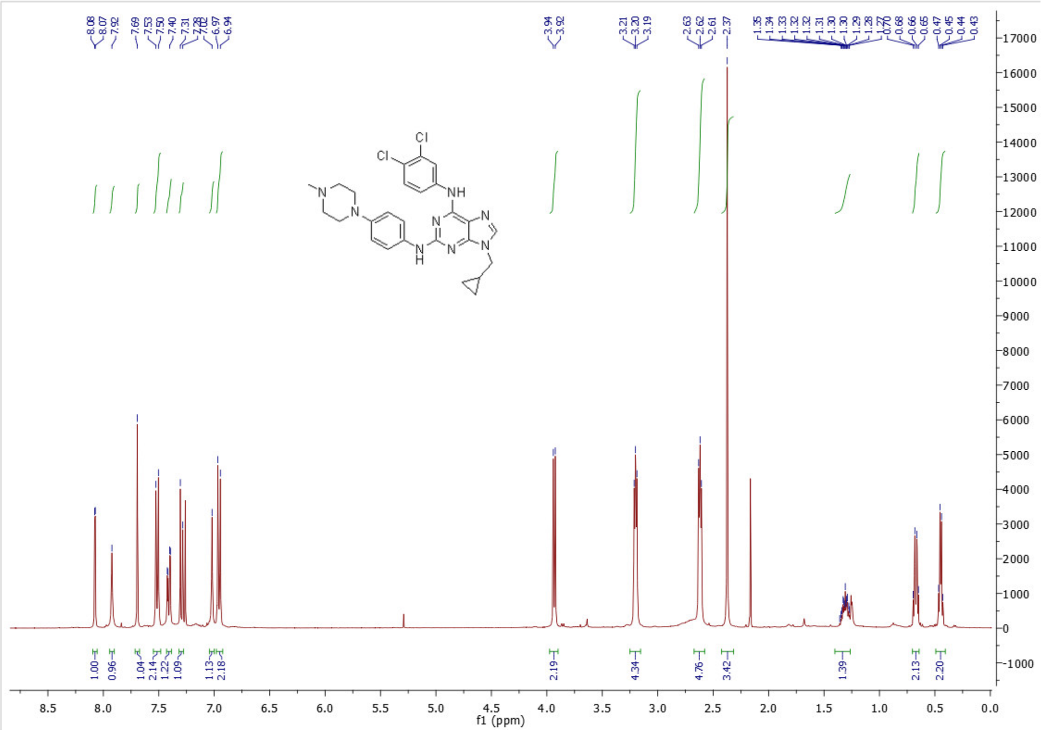

^{13}C NMR spectra of compound **11n**

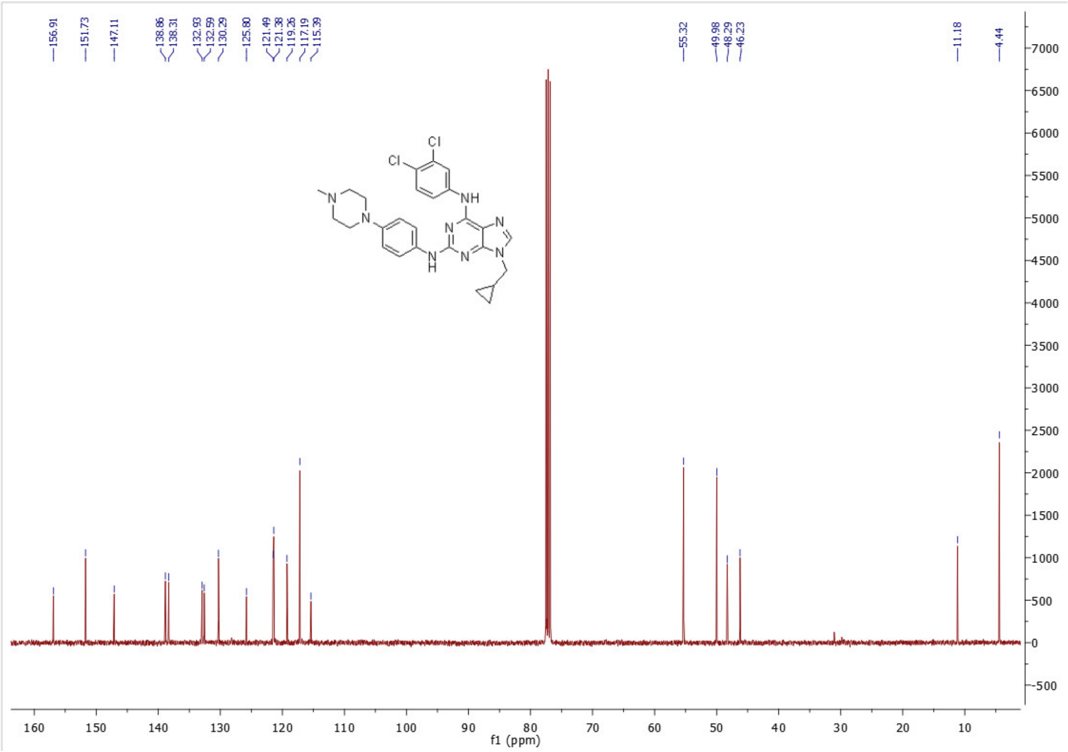

^{1}H NMR spectra of compound **11o**

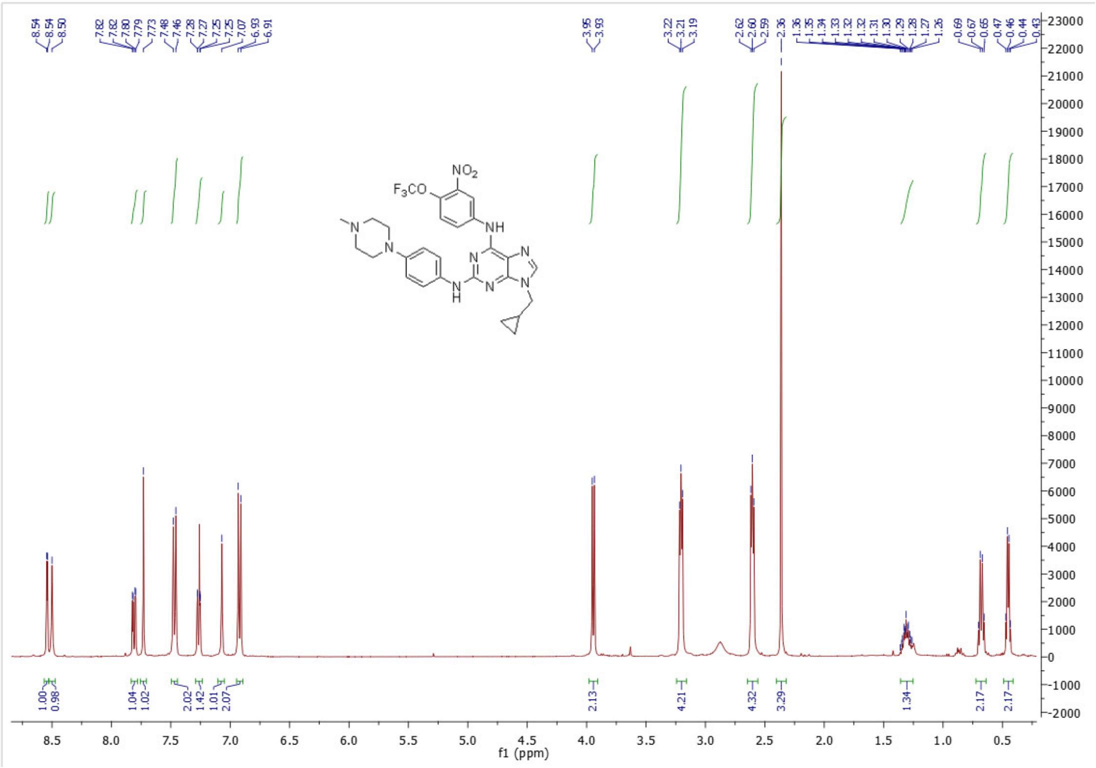

^{13}C NMR spectra of compound **11o**

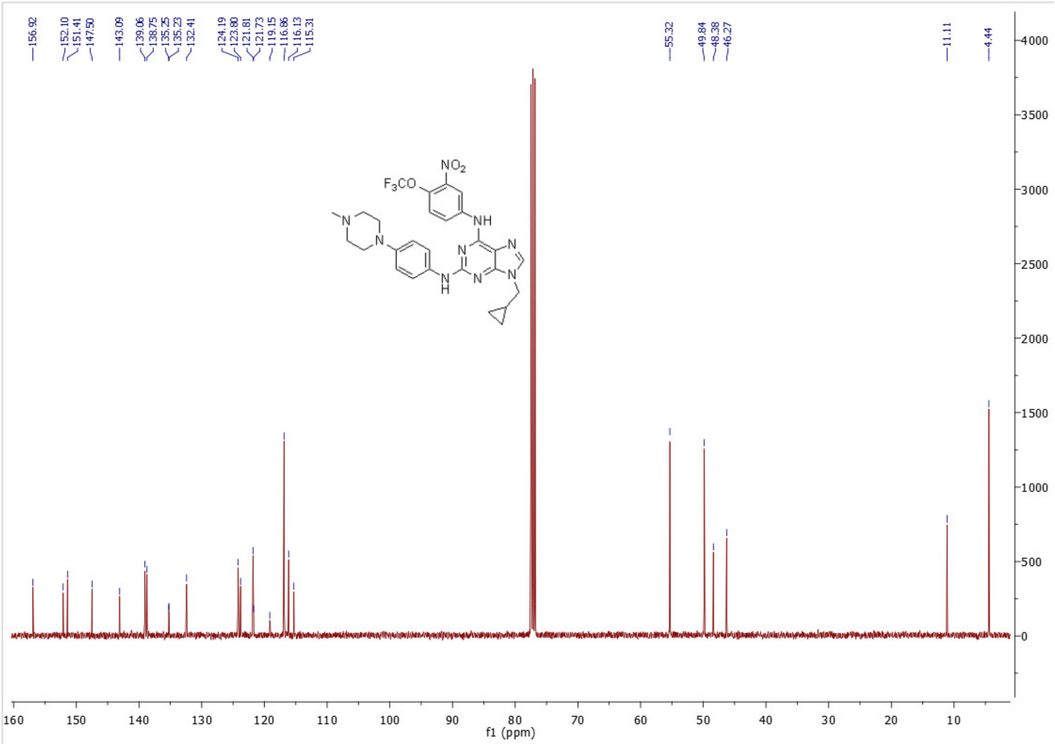

^{19}F NMR spectra of compound **11o**

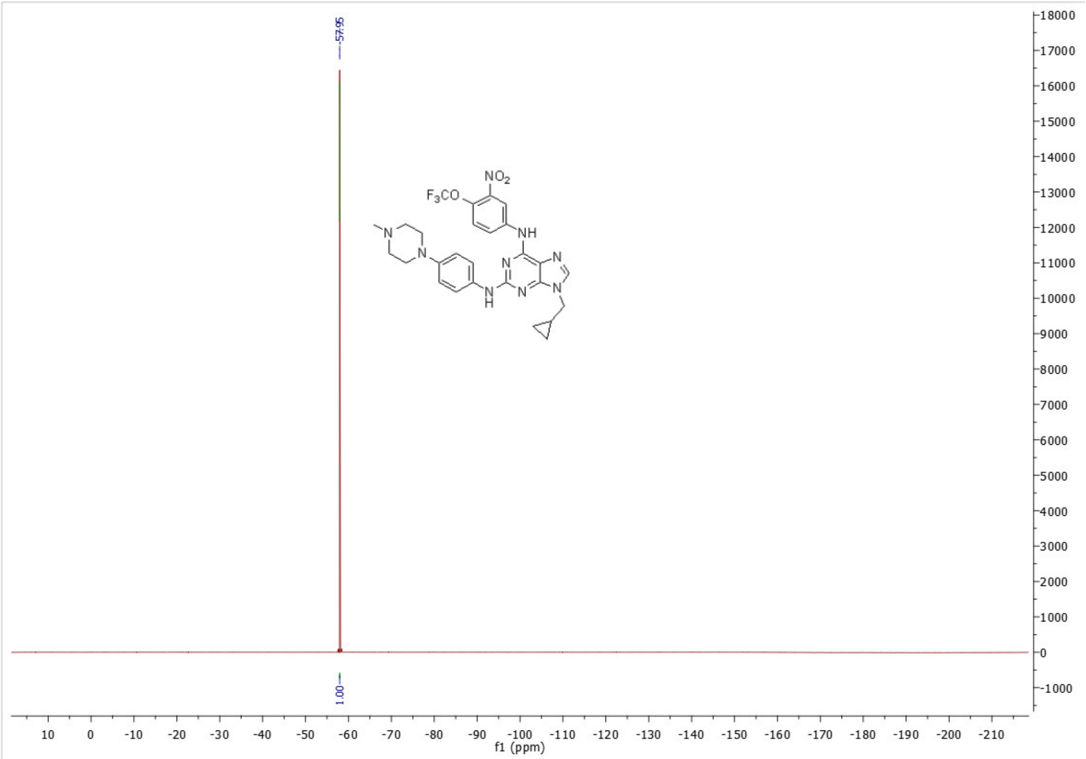

^{1}H NMR spectra of compound **12a**

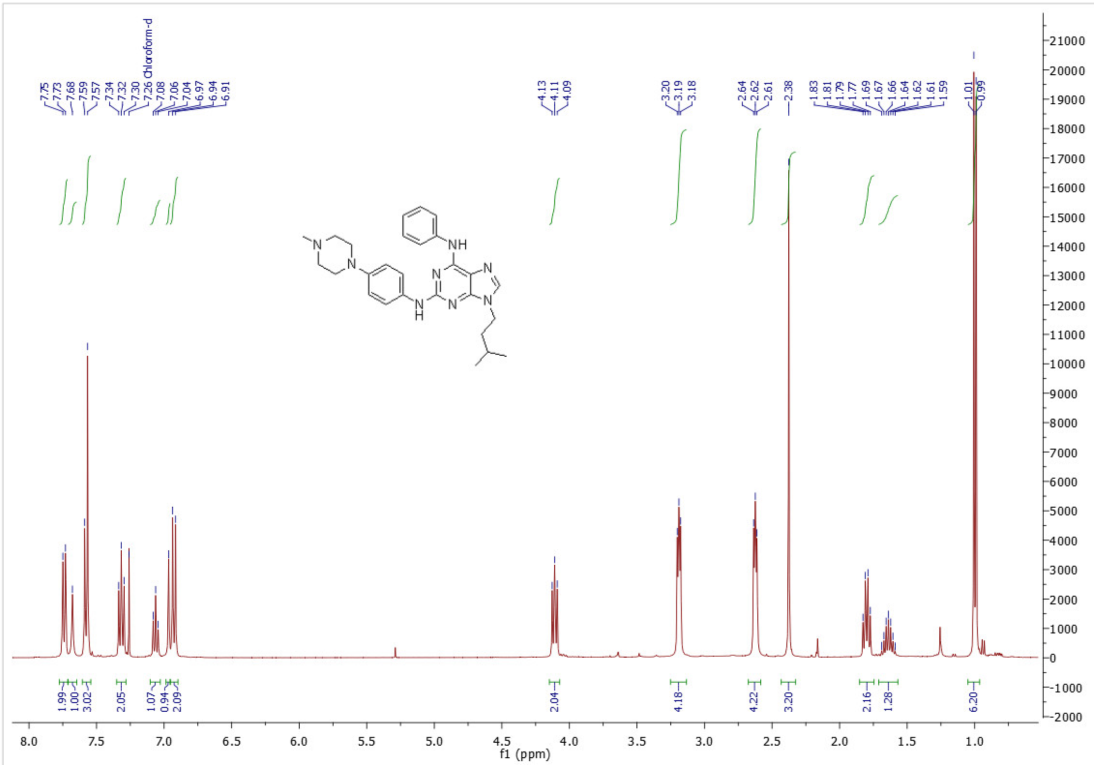

^{13}C NMR spectra of compound **12a**

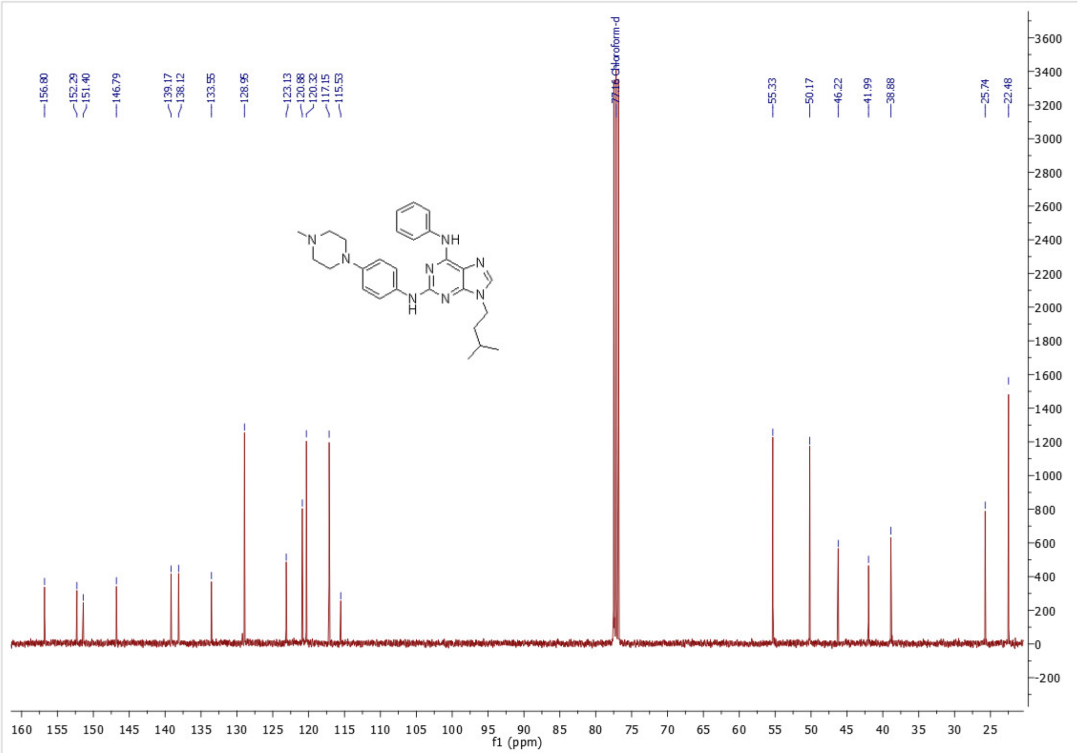

^{1}H NMR spectra of compound **12b**

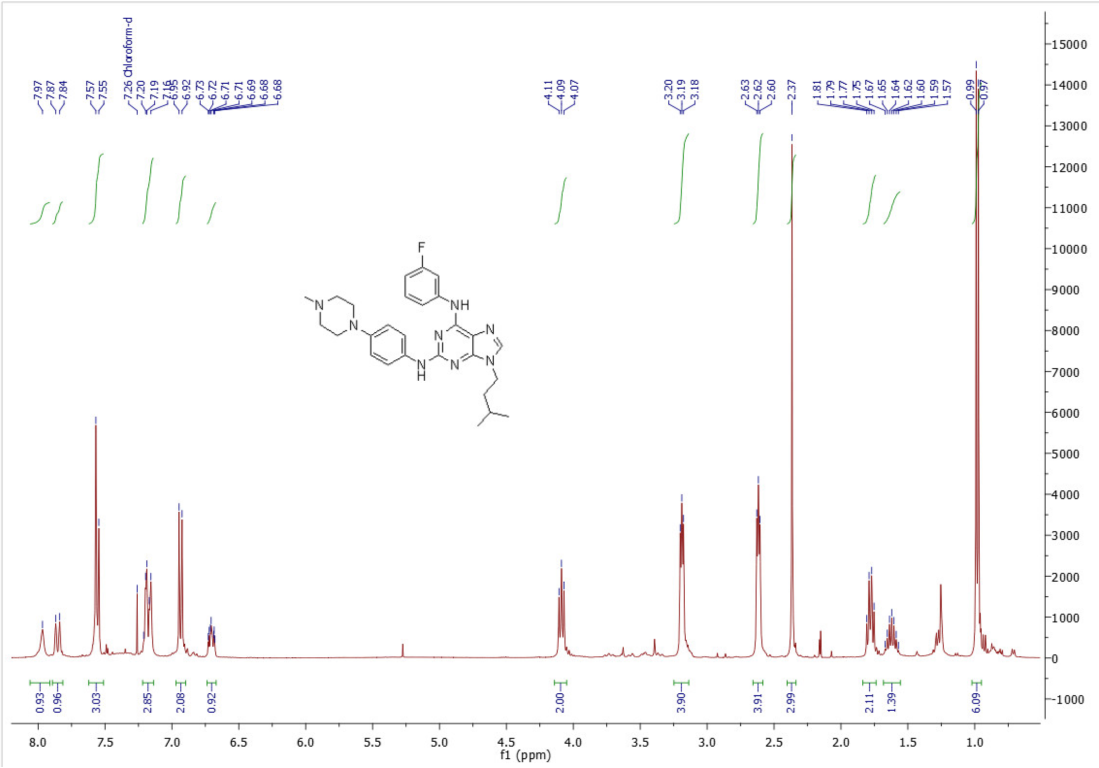

^{13}C NMR spectra of compound **12b**

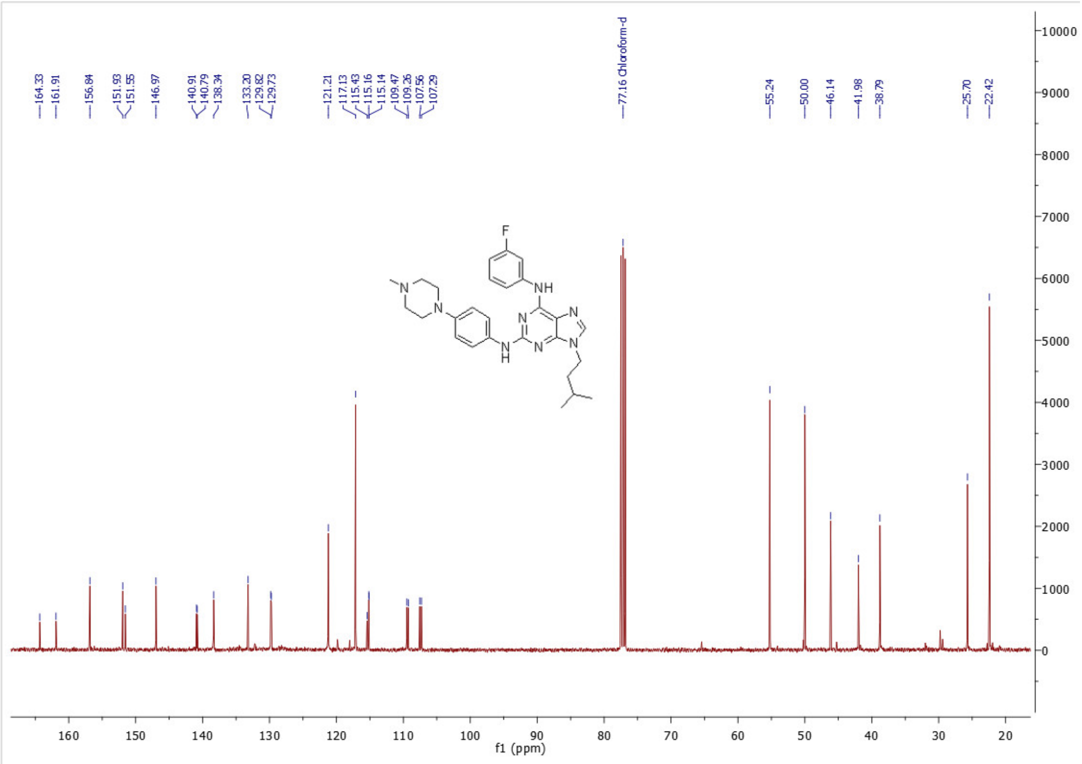

^{19}F NMR spectra of compound **12b**

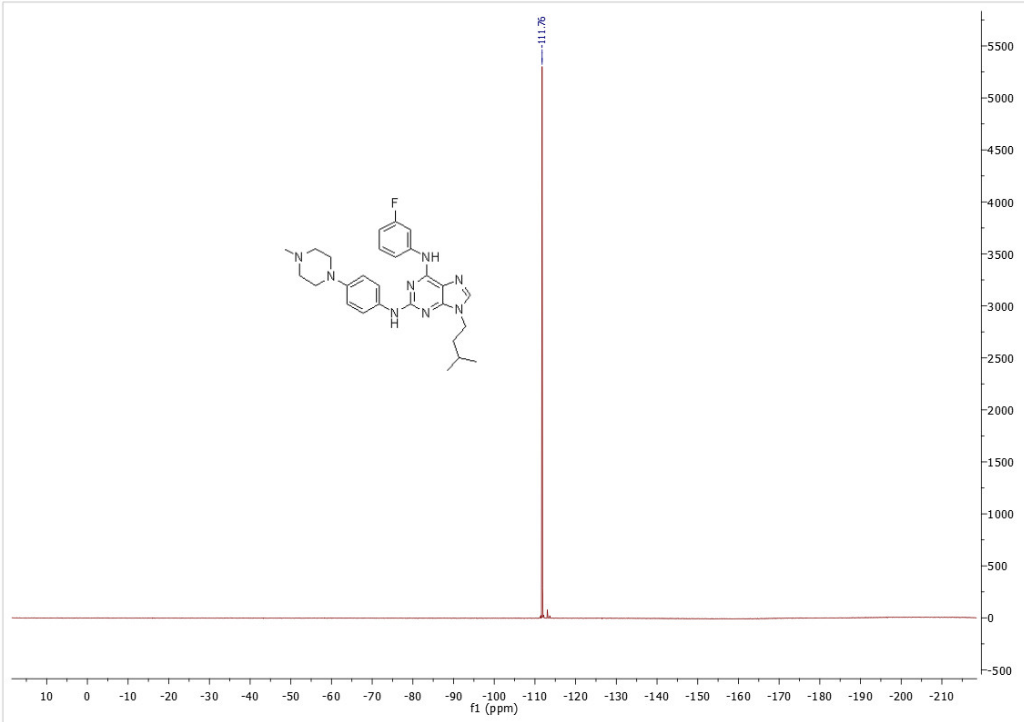

^{1}H NMR spectra of compound **12c**

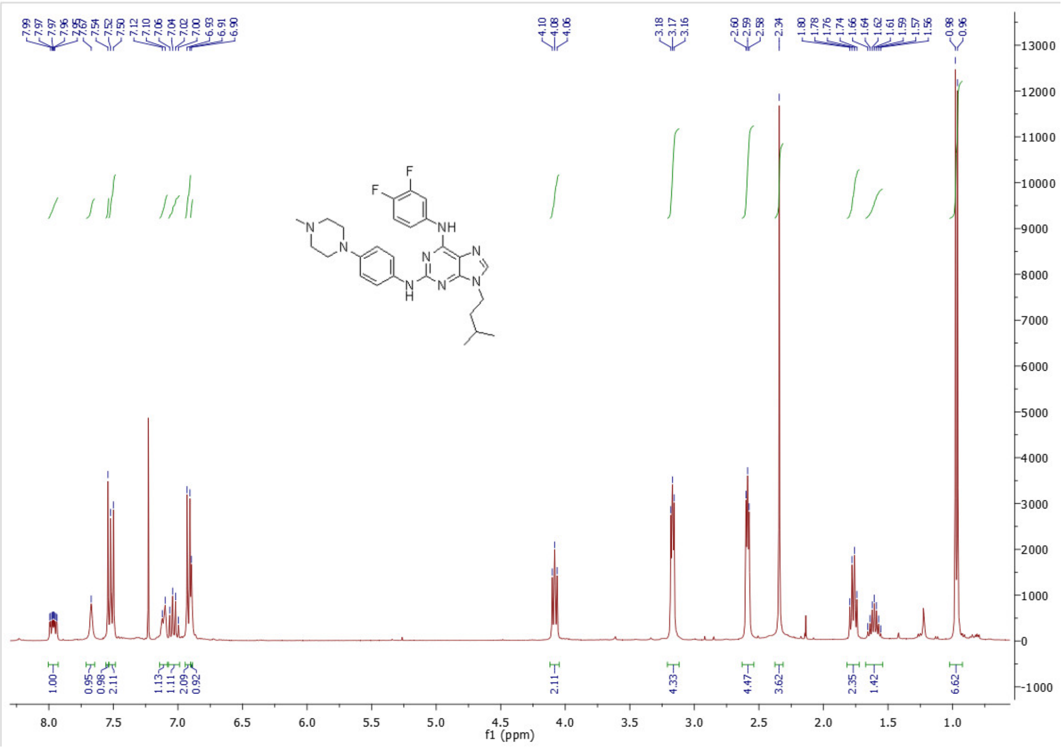

^{13}C NMR spectra of compound **12c**

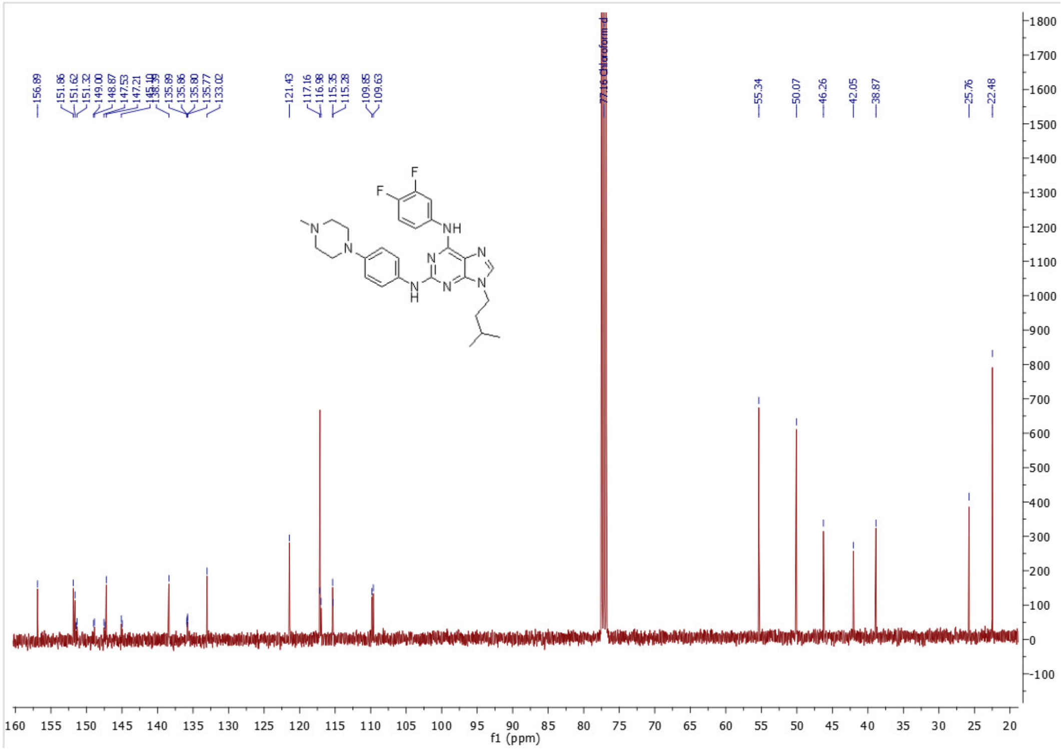

^{19}F NMR spectra of compound **12c**

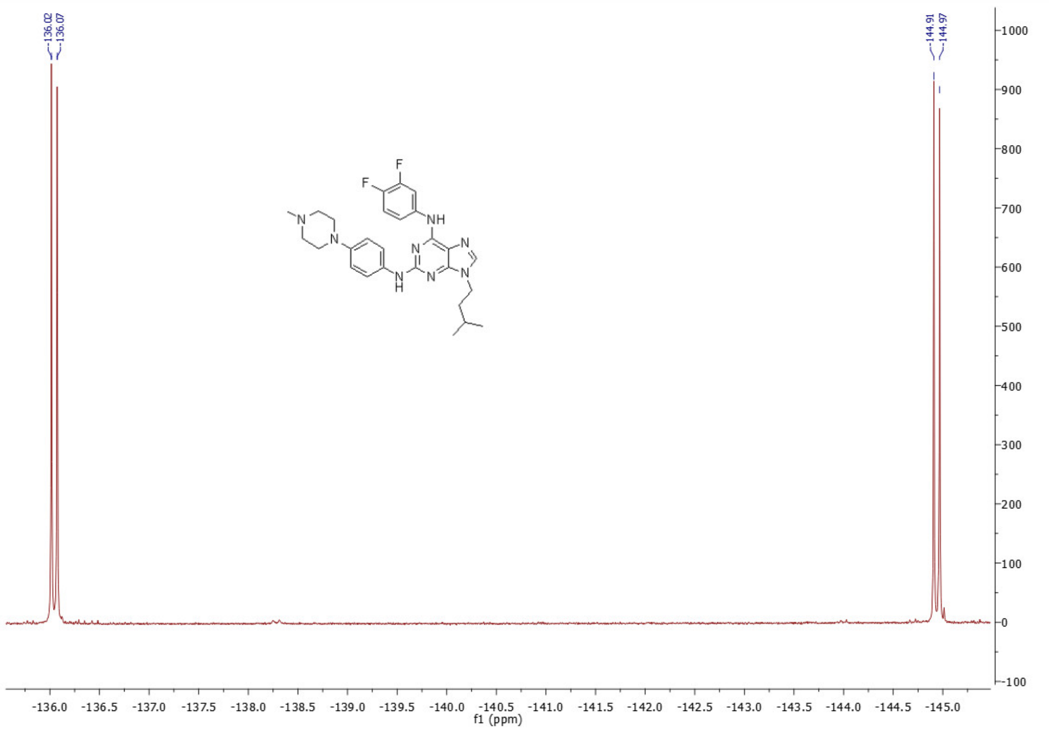

^{1}H NMR spectra of compound **12d**

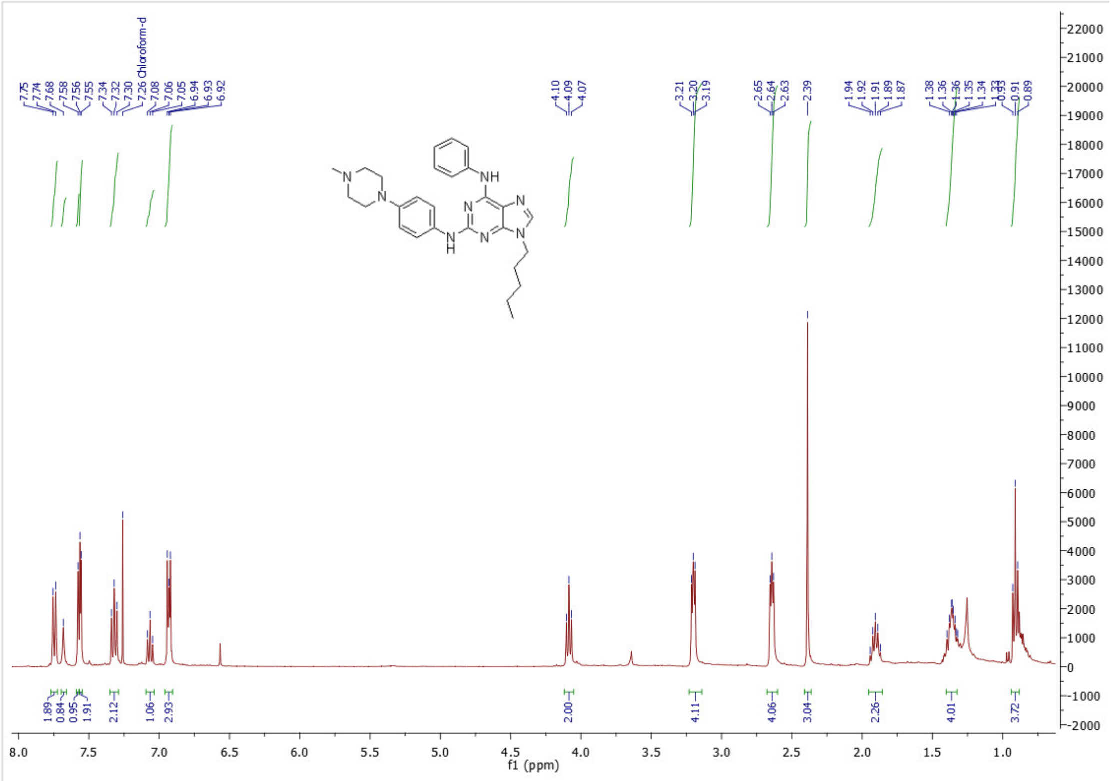

^{13}C NMR spectra of compound **12d**

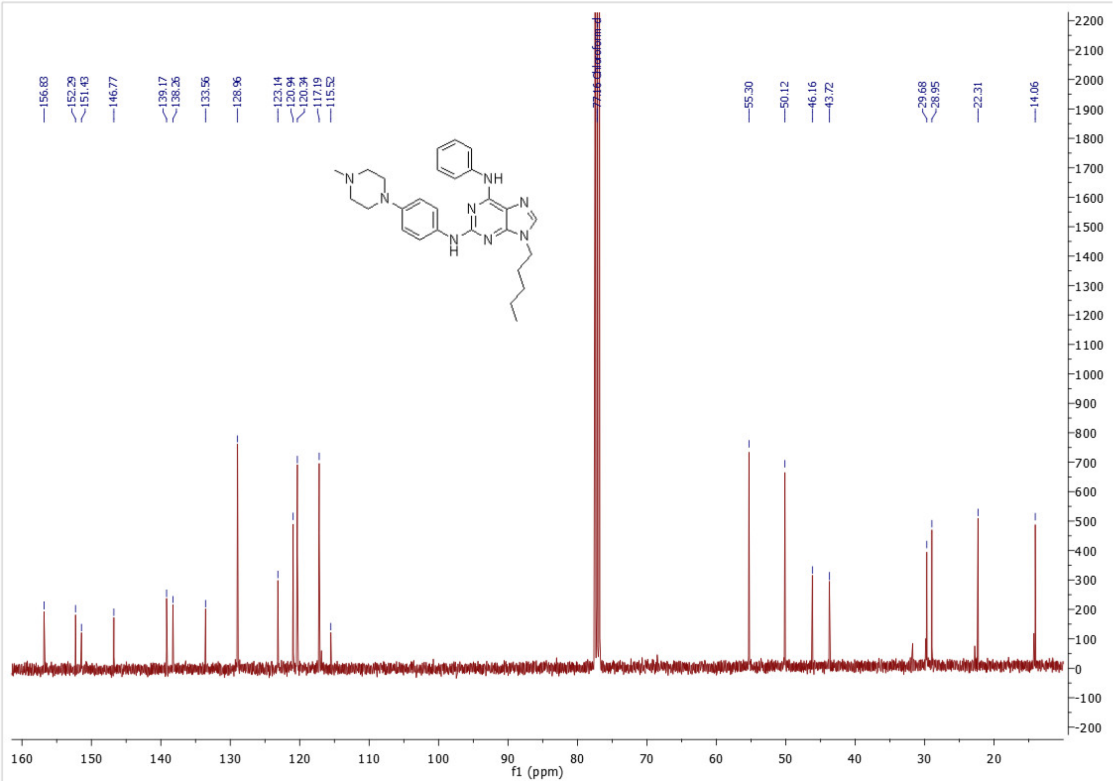

^{11}H NMR spectra of compound **12e**

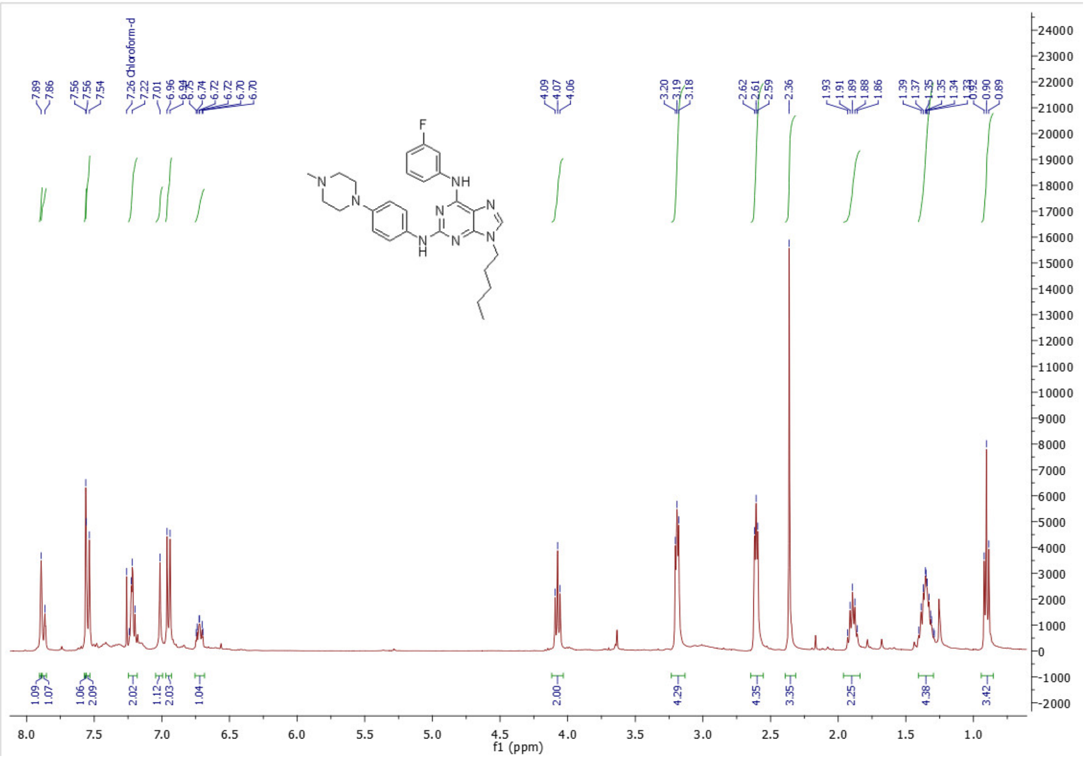

^{13}C NMR spectra of compound **12e**

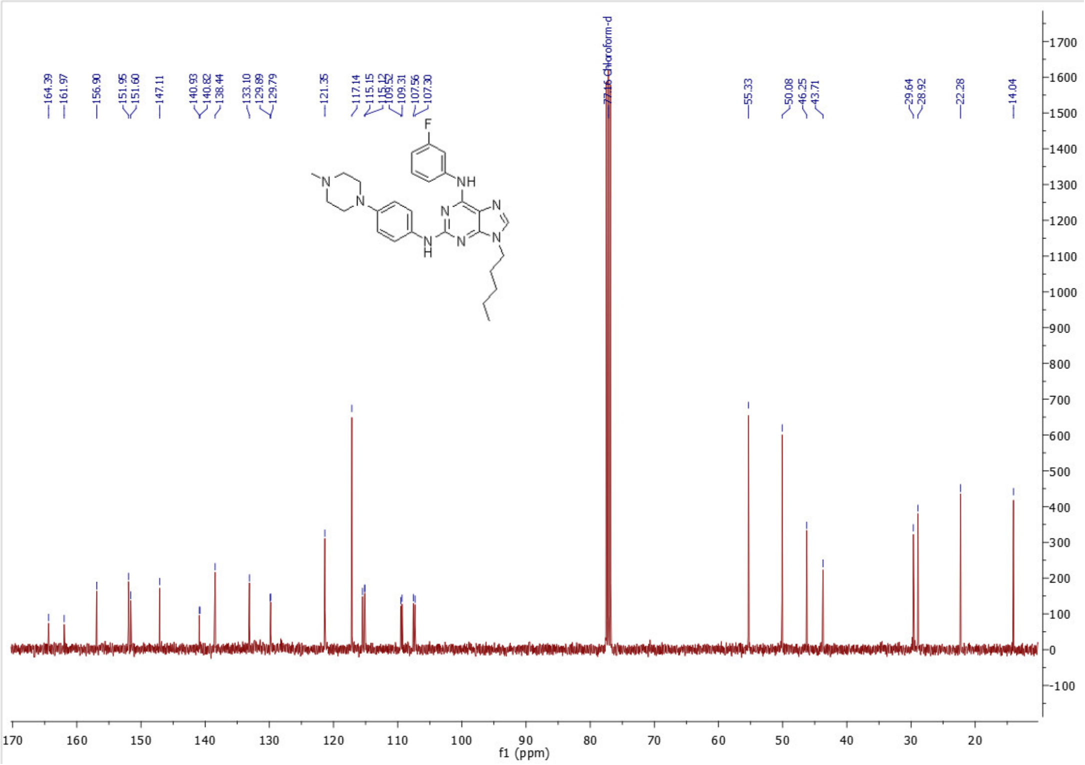

^{19}F NMR spectra of compound **12e**

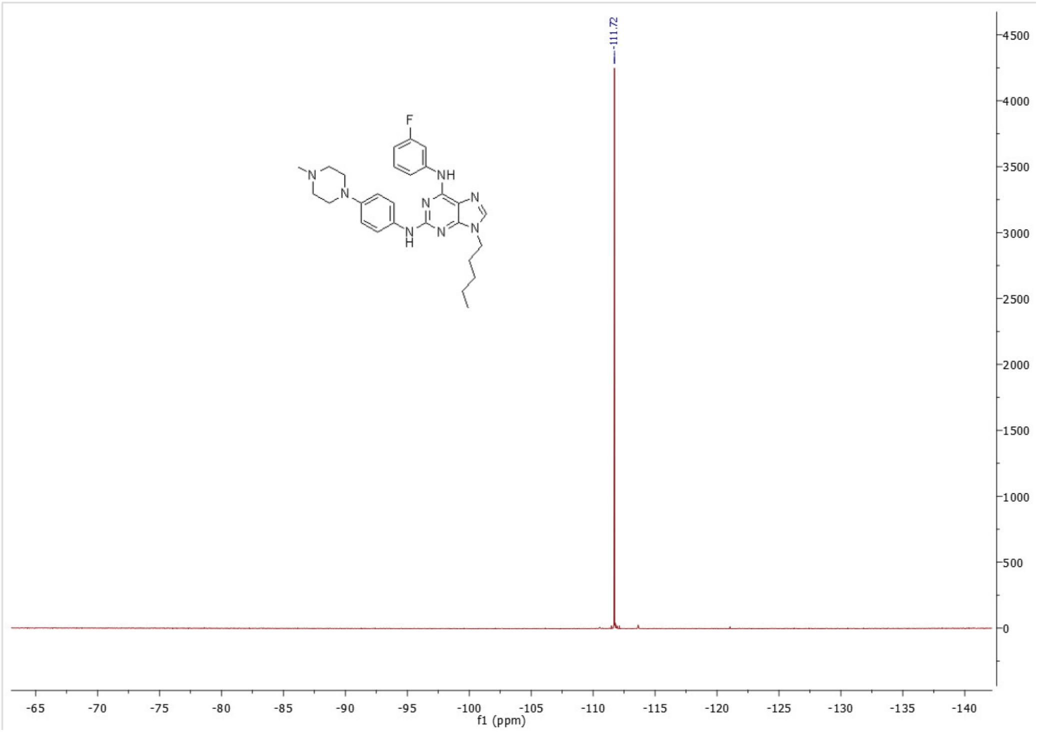

^{1}H NMR spectra of compound **12f**

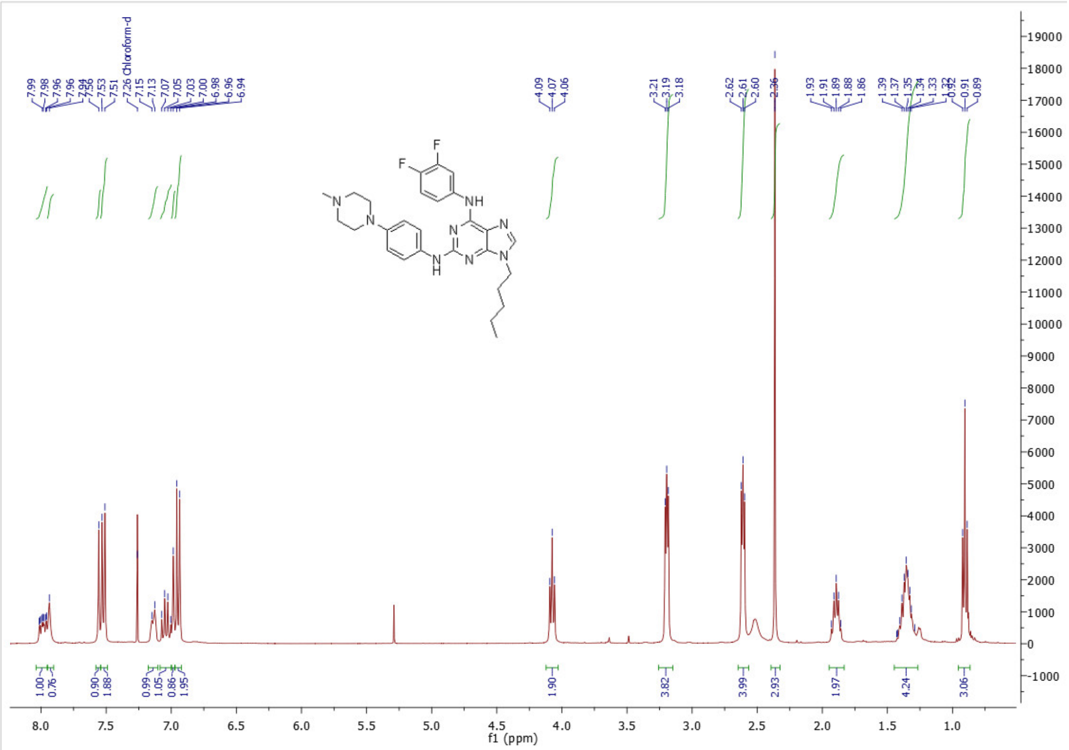

^{13}C NMR spectra of compound **12f**

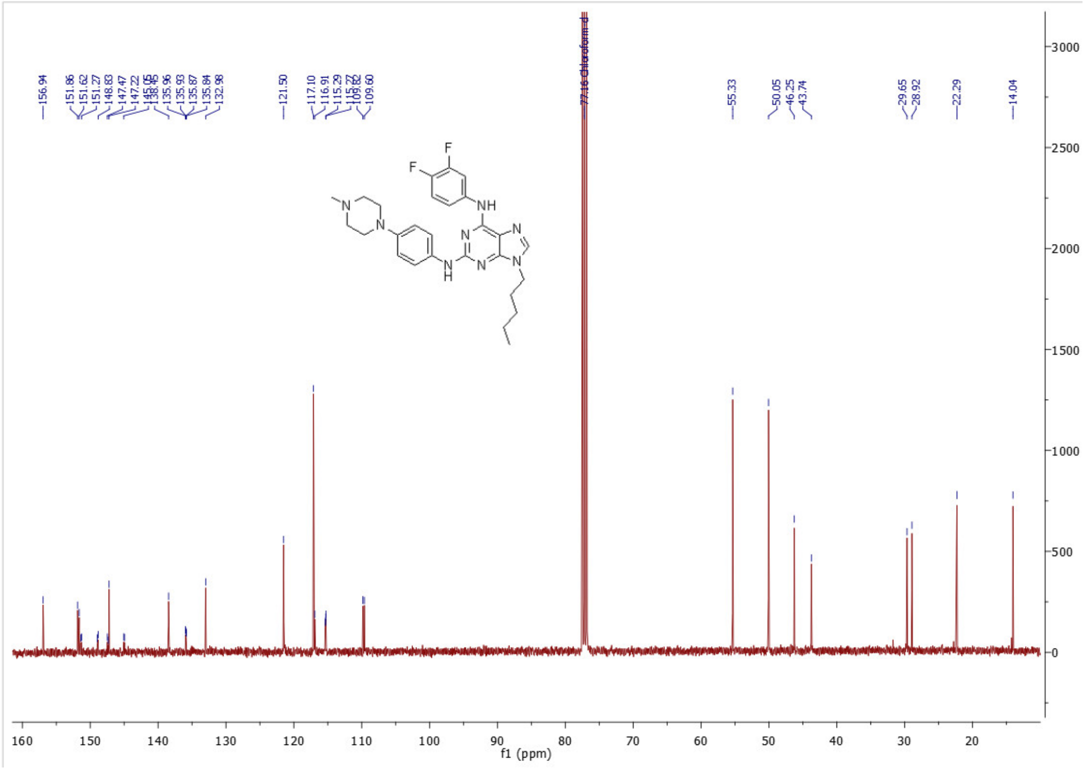

^{19}F NMR spectra of compound **12f**

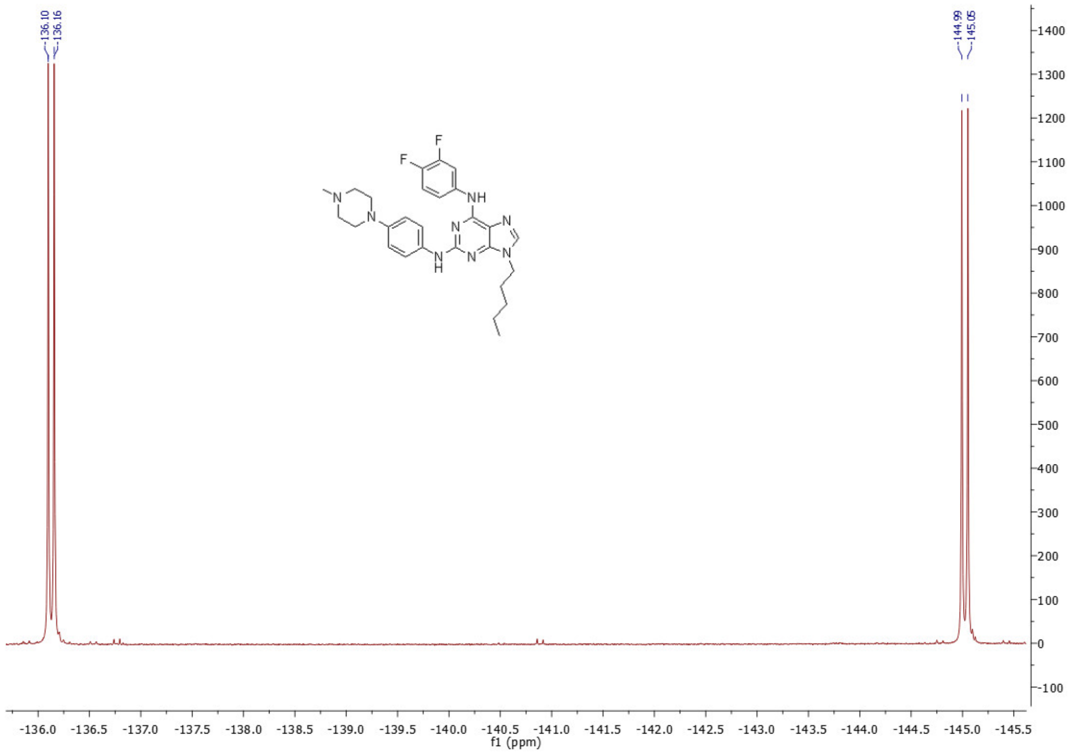

^{1}H NMR spectra of compound **12g**

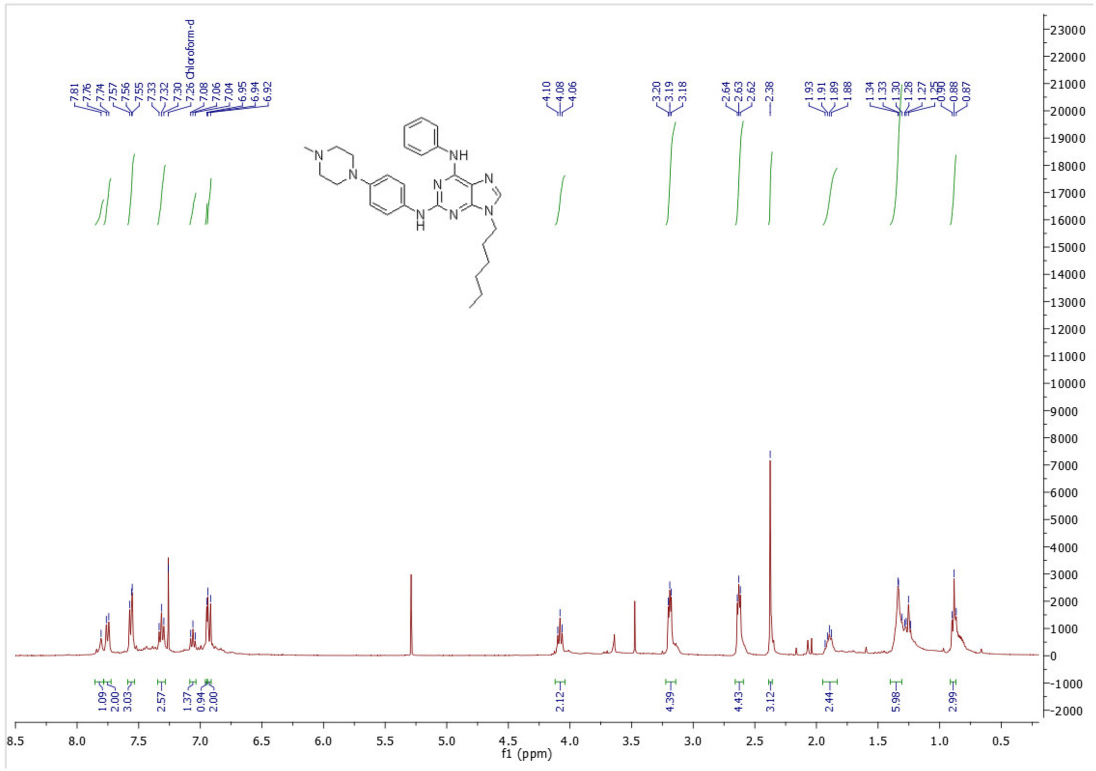

^{13}C NMR spectra of compound **12g**

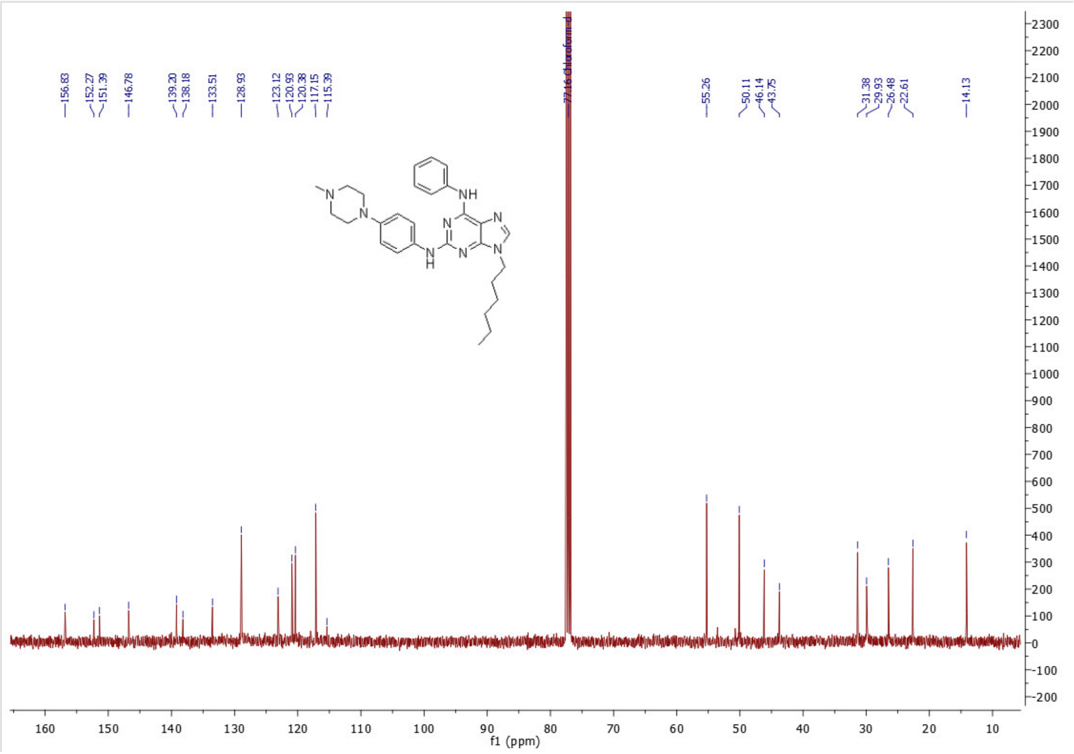

^{1}H NMR spectra of compound **12h**

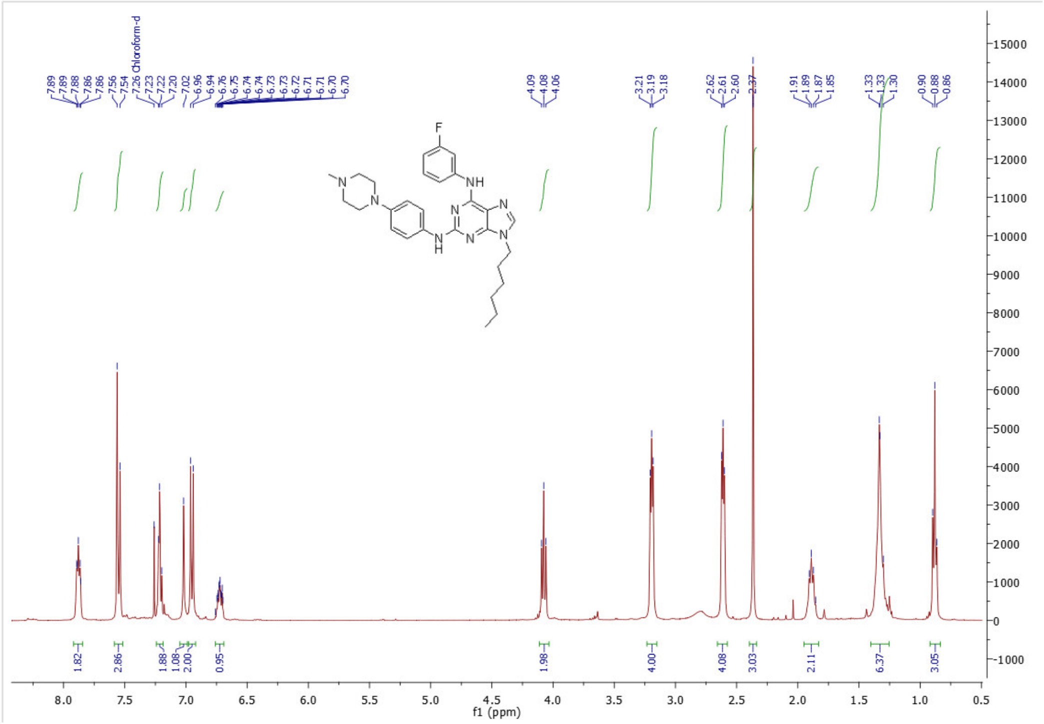

^{13}C NMR spectra of compound **12h**

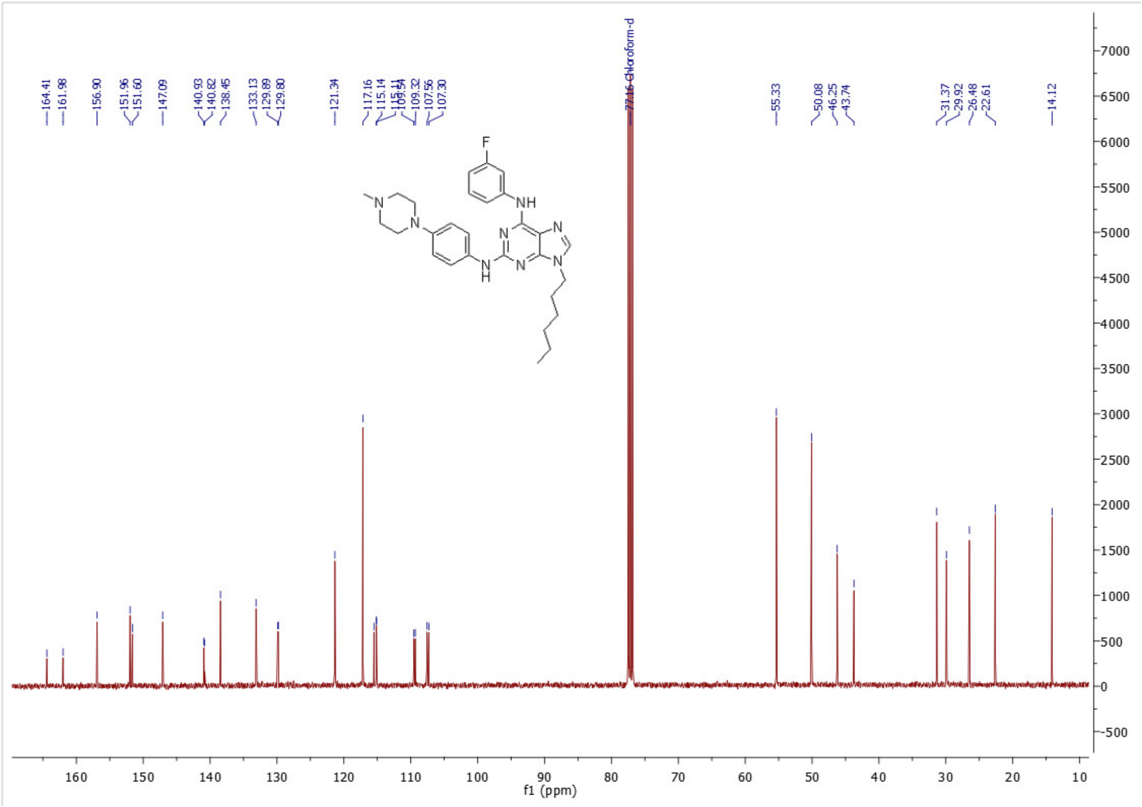

^{19}F NMR spectra of compound **12h**

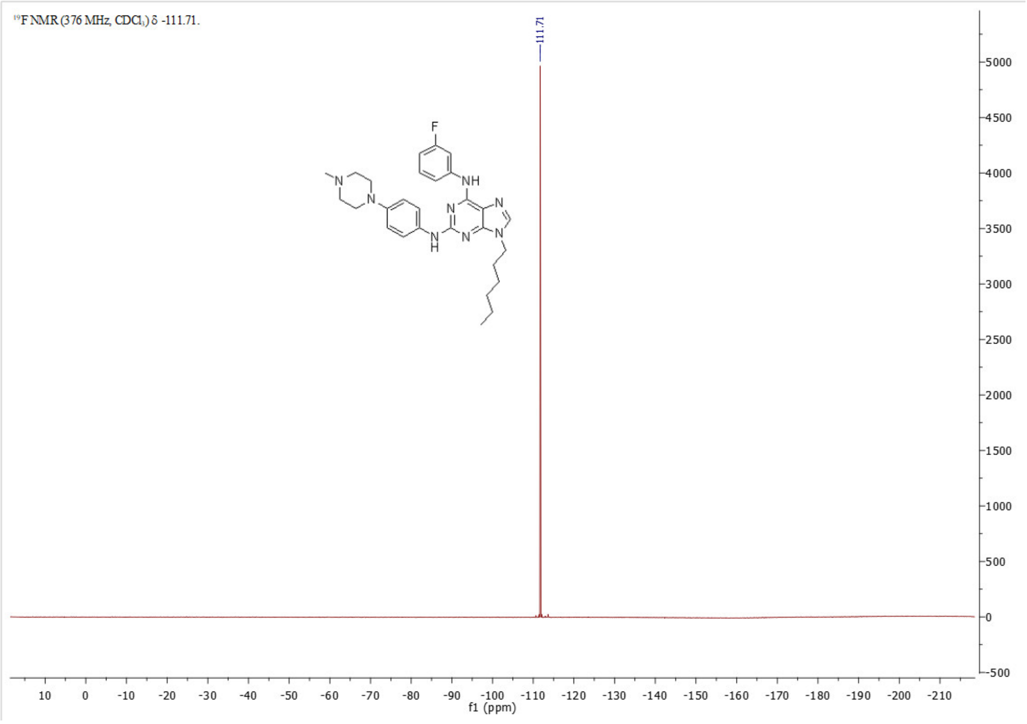

^{1}H NMR spectra of compound **12i**

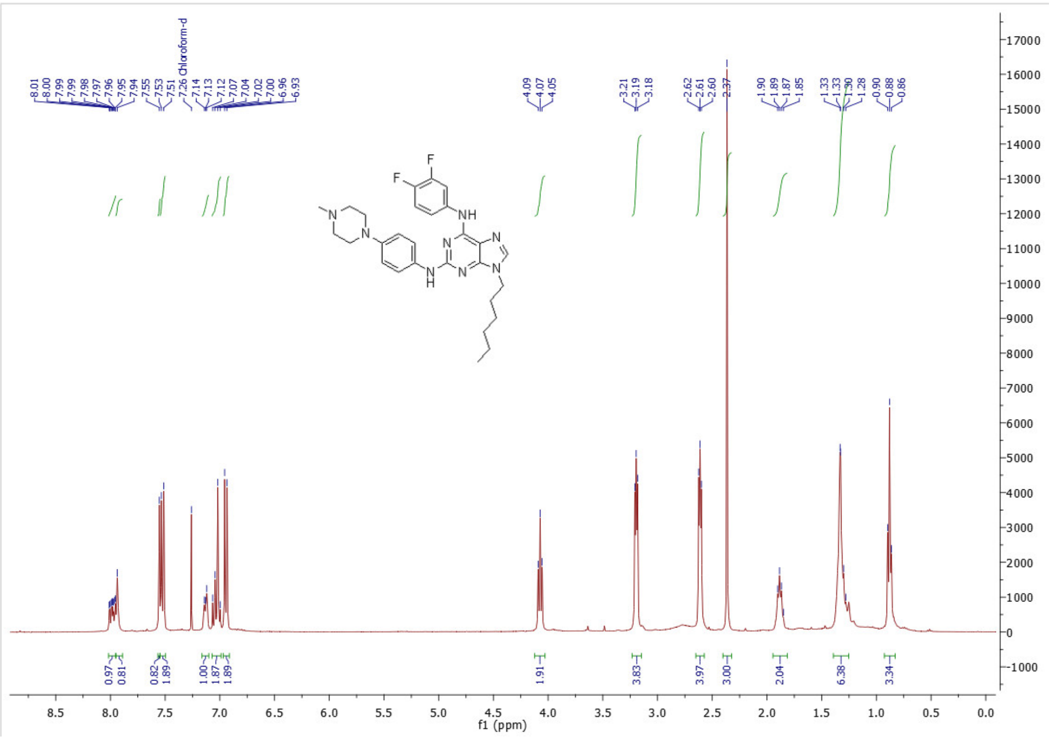

^{13}C NMR spectra of compound **12i**

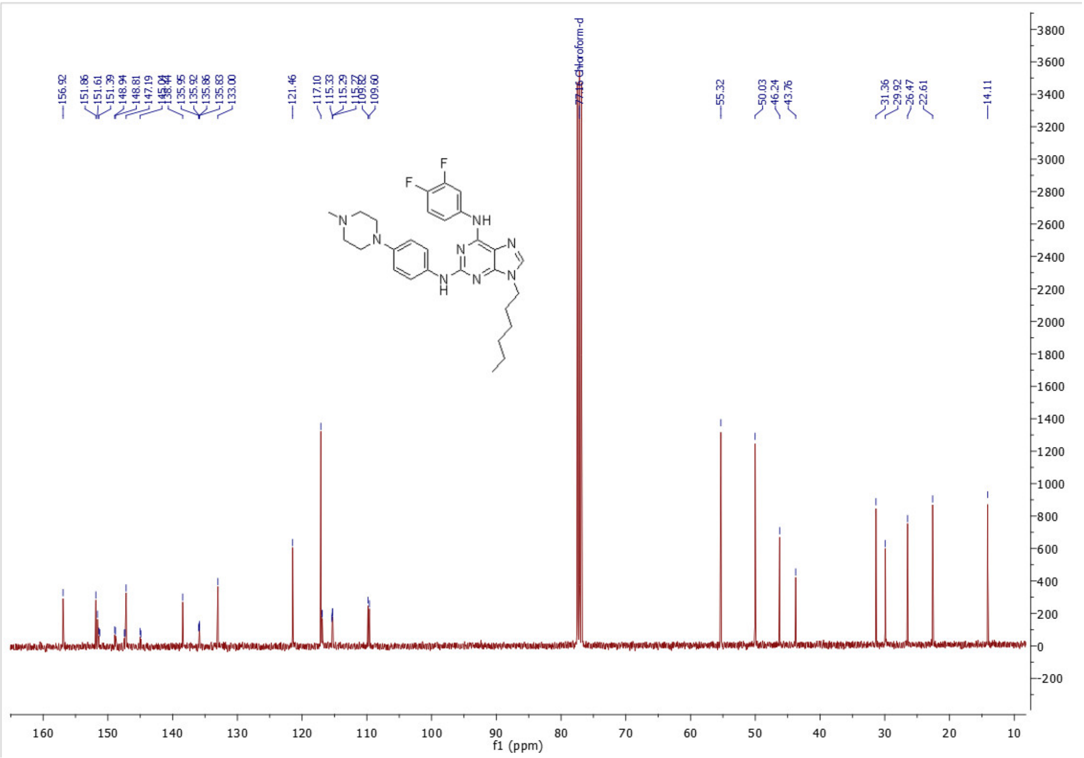

^{19}F NMR spectra of compound **12i**

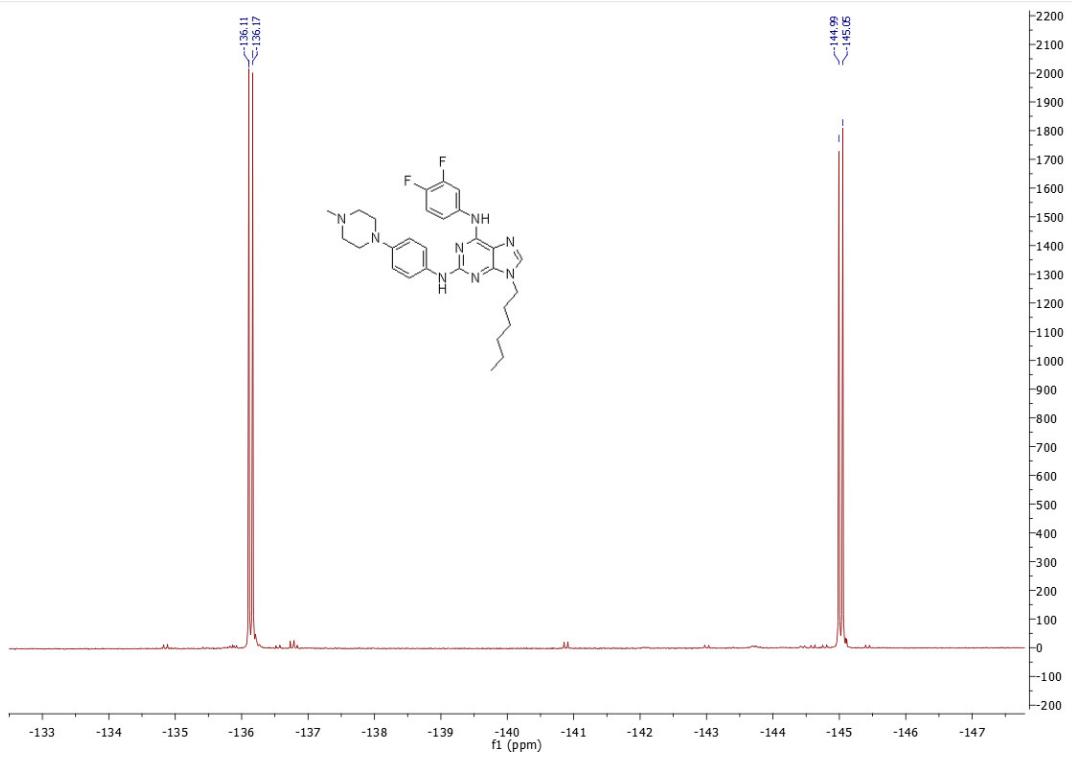

^{1}H NMR spectra of compound **12j**

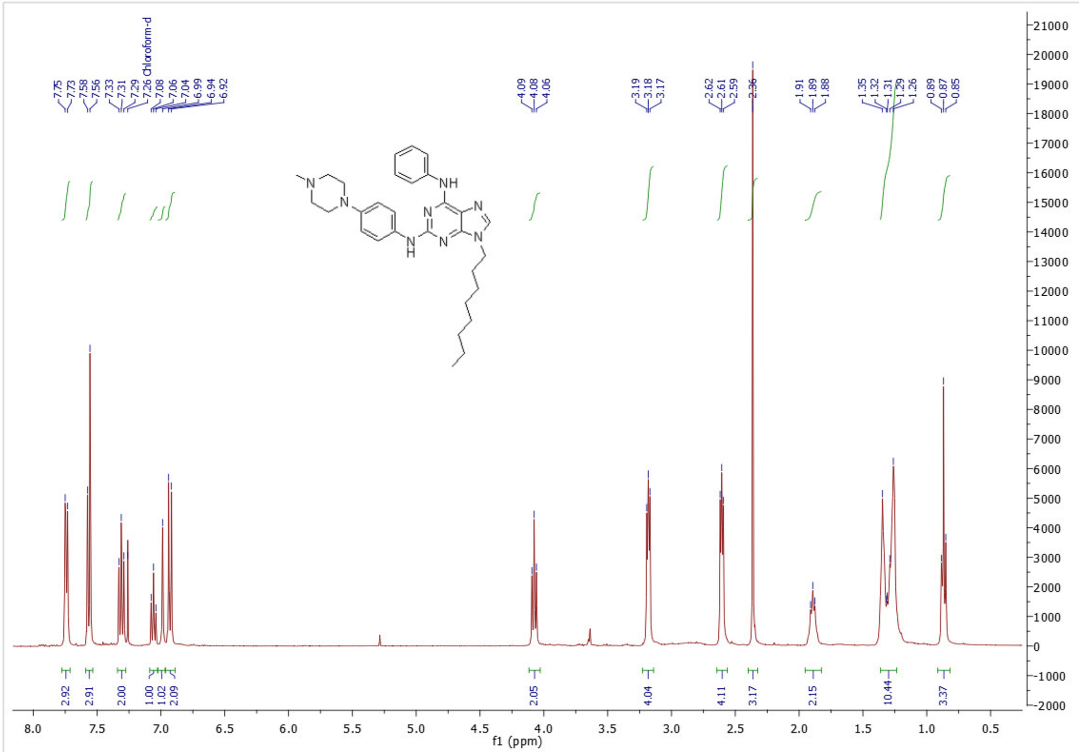

^{13}C NMR spectra of compound **12j**

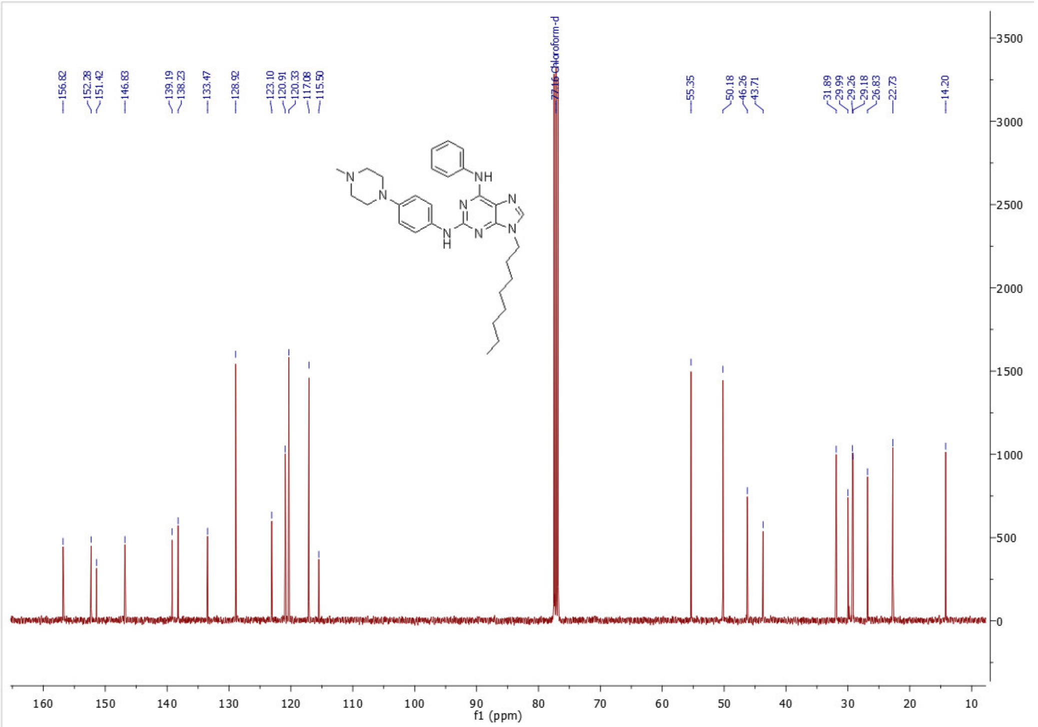

^{1}H NMR spectra of compound **12k**

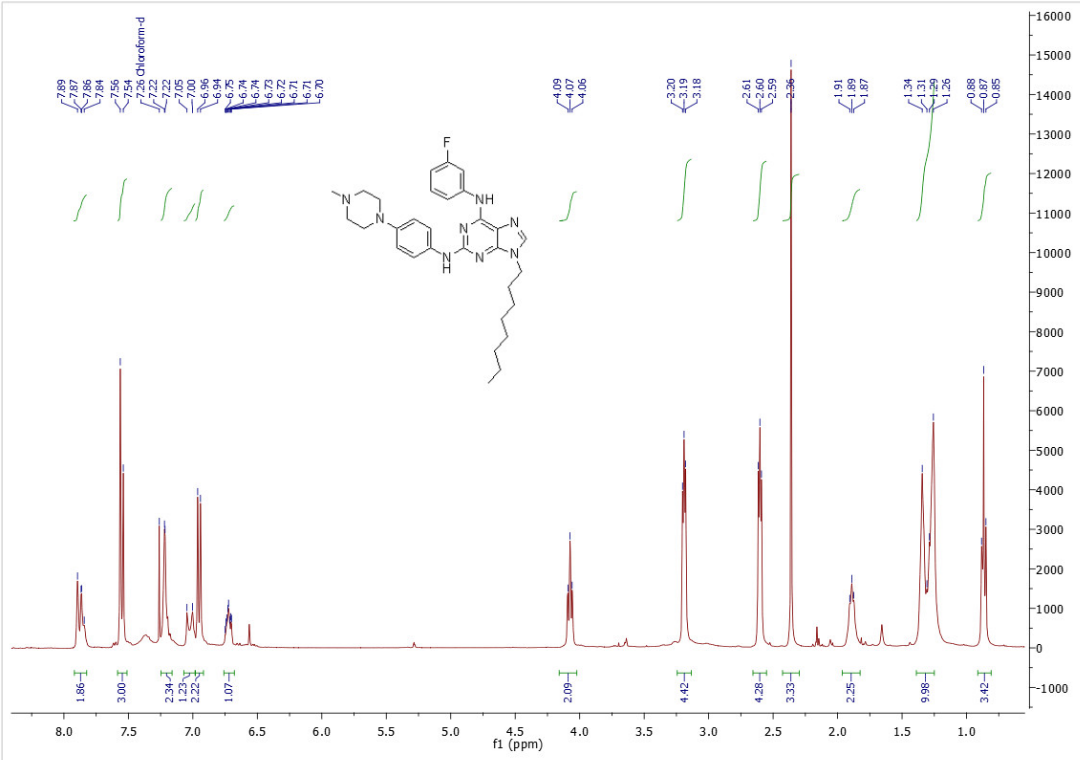

^{13}C NMR spectra of compound **12k**

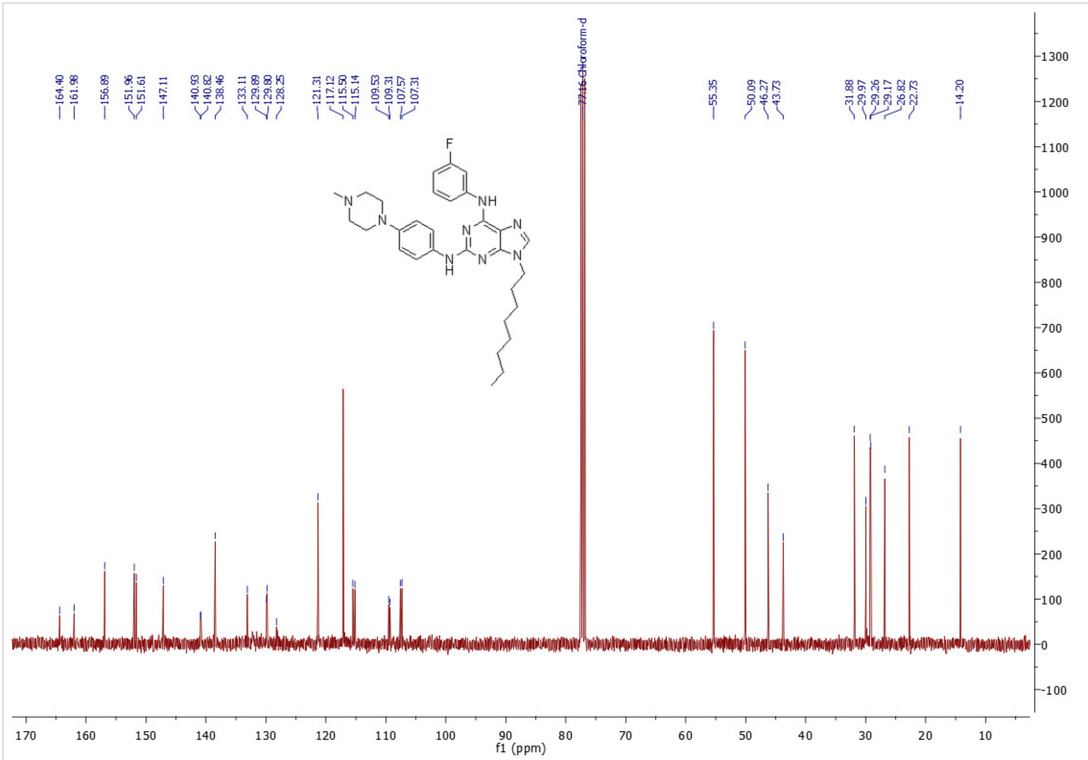

^{19}F NMR spectra of compound **12k**

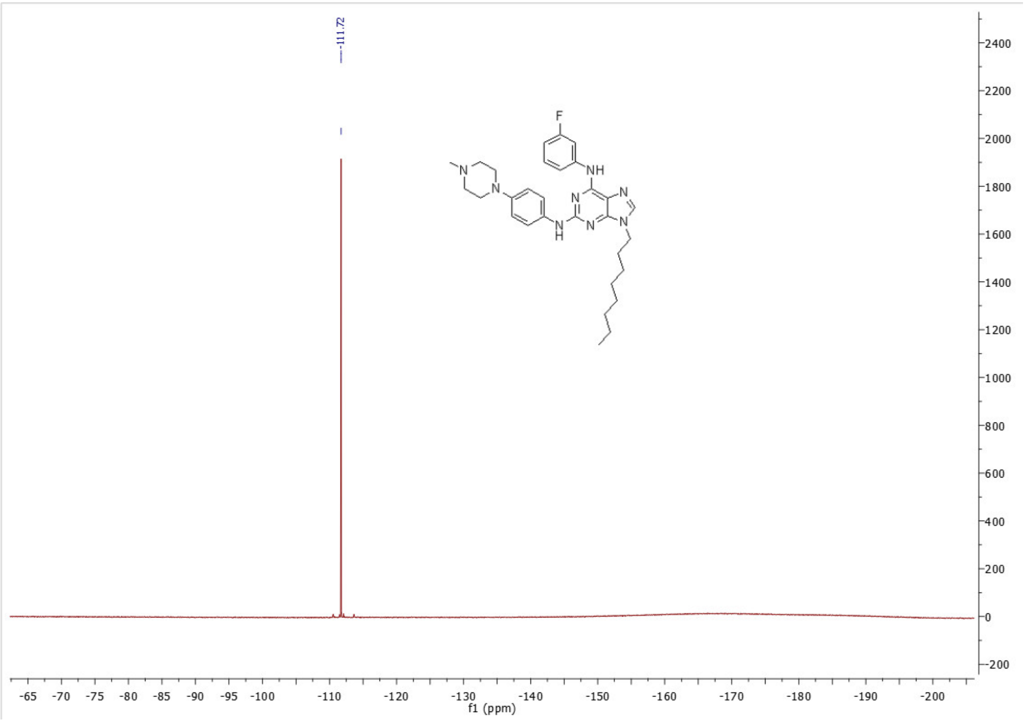

^{1}H NMR spectra of compound **12l**

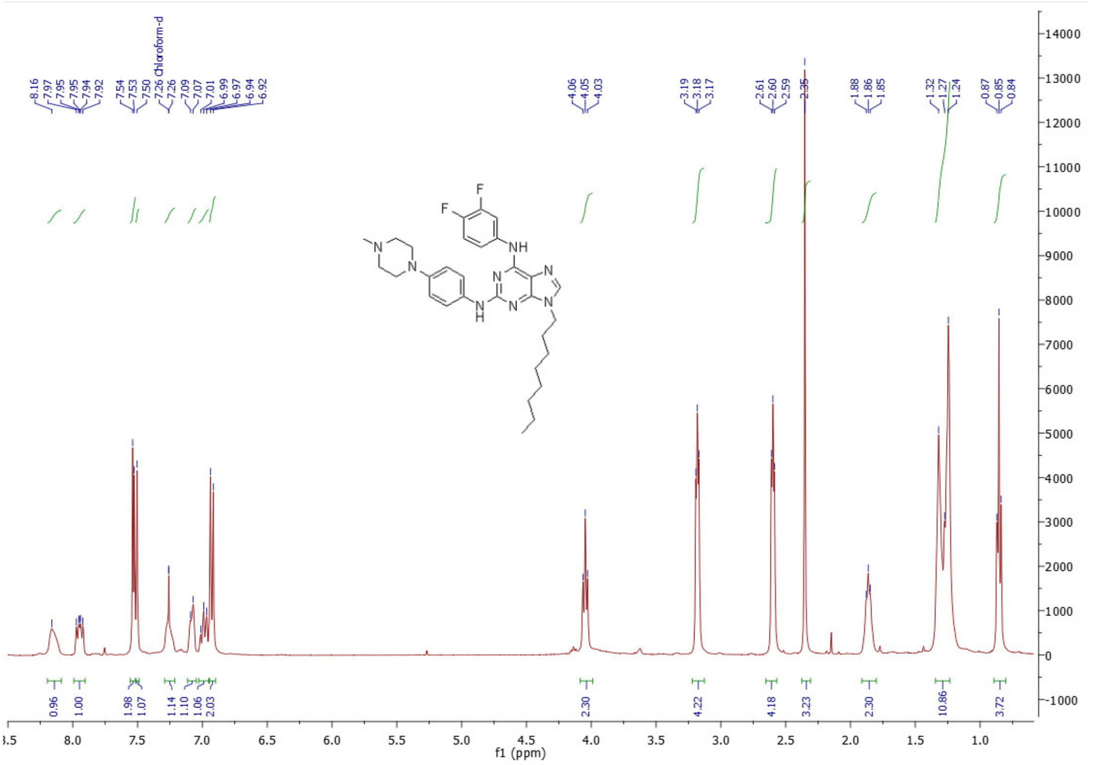

^{13}C NMR spectra of compound **12l**

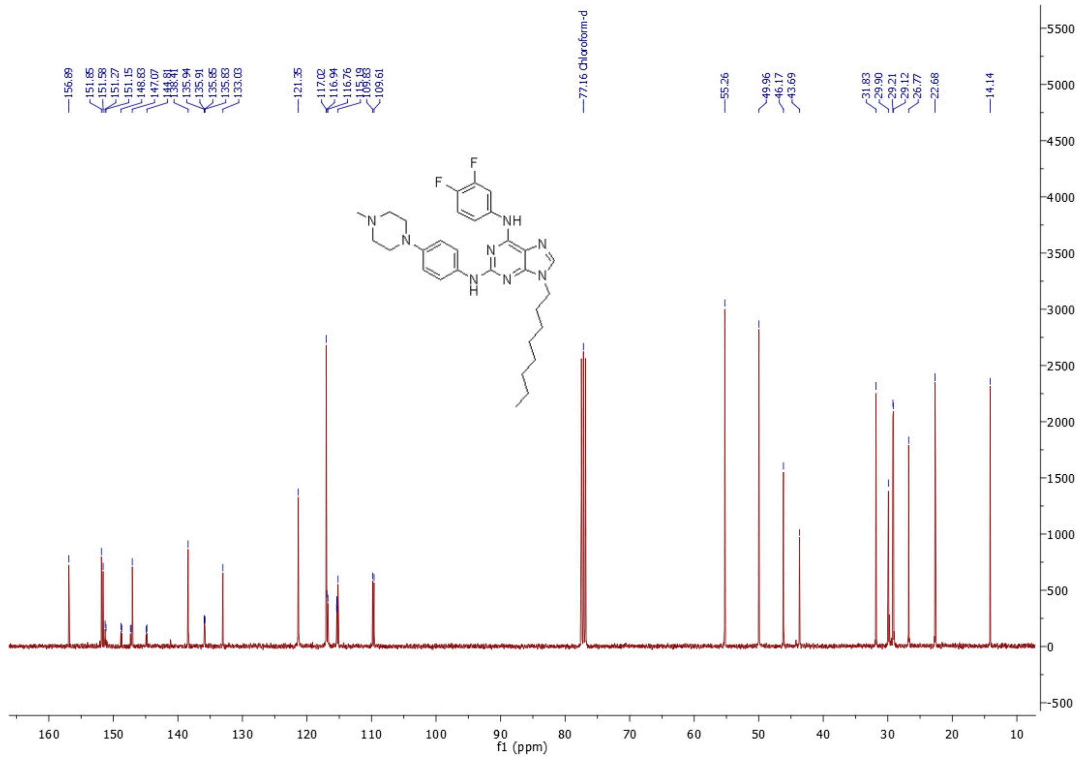

^{19}F NMR spectra of compound **12l**

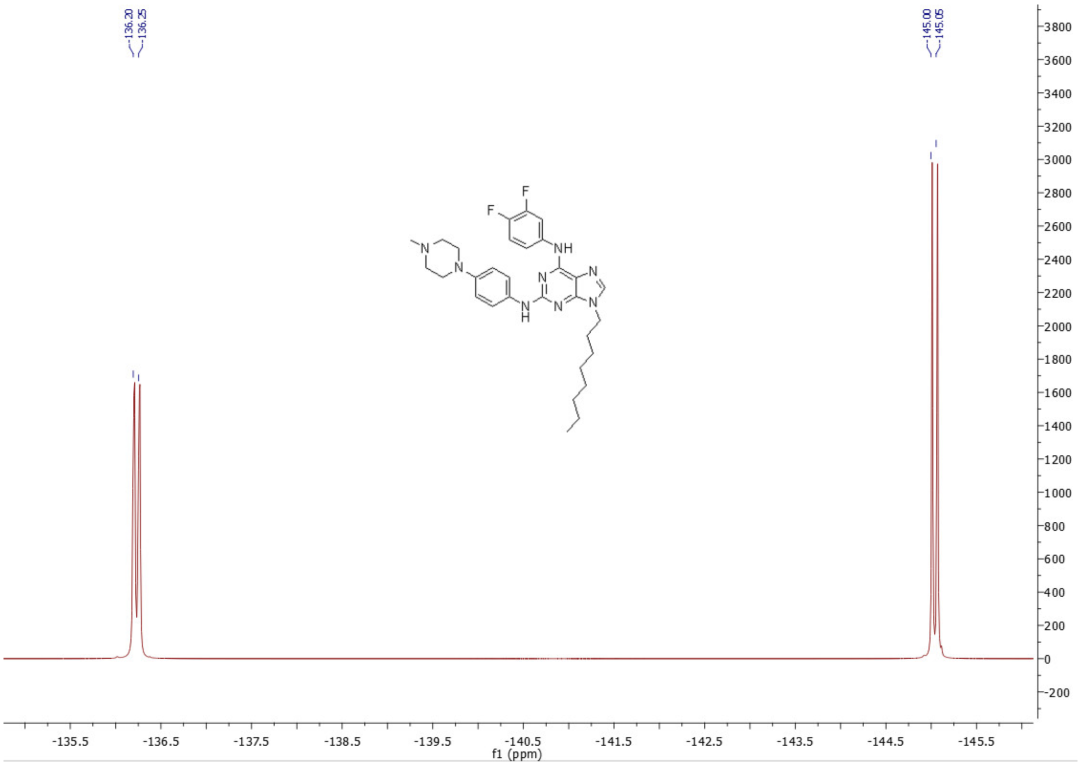

^{1}H NMR spectra of compound **14**

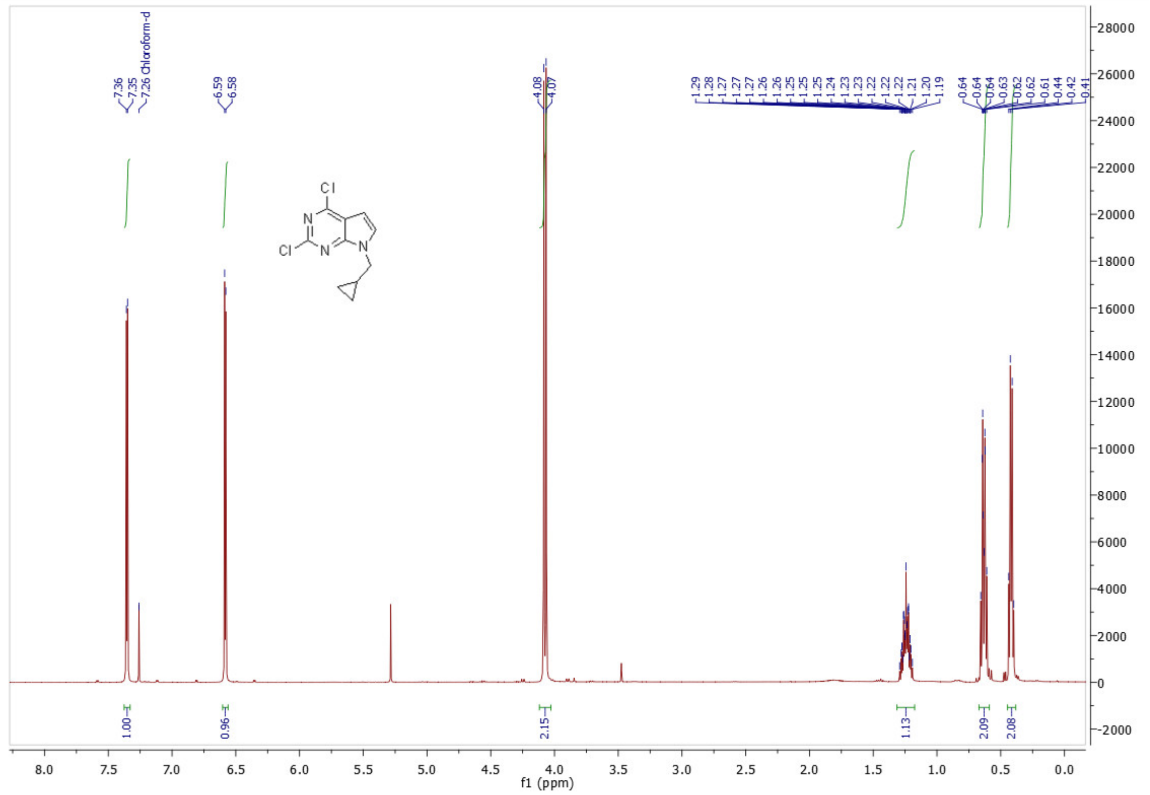

^{13}C NMR spectra of compound **14**

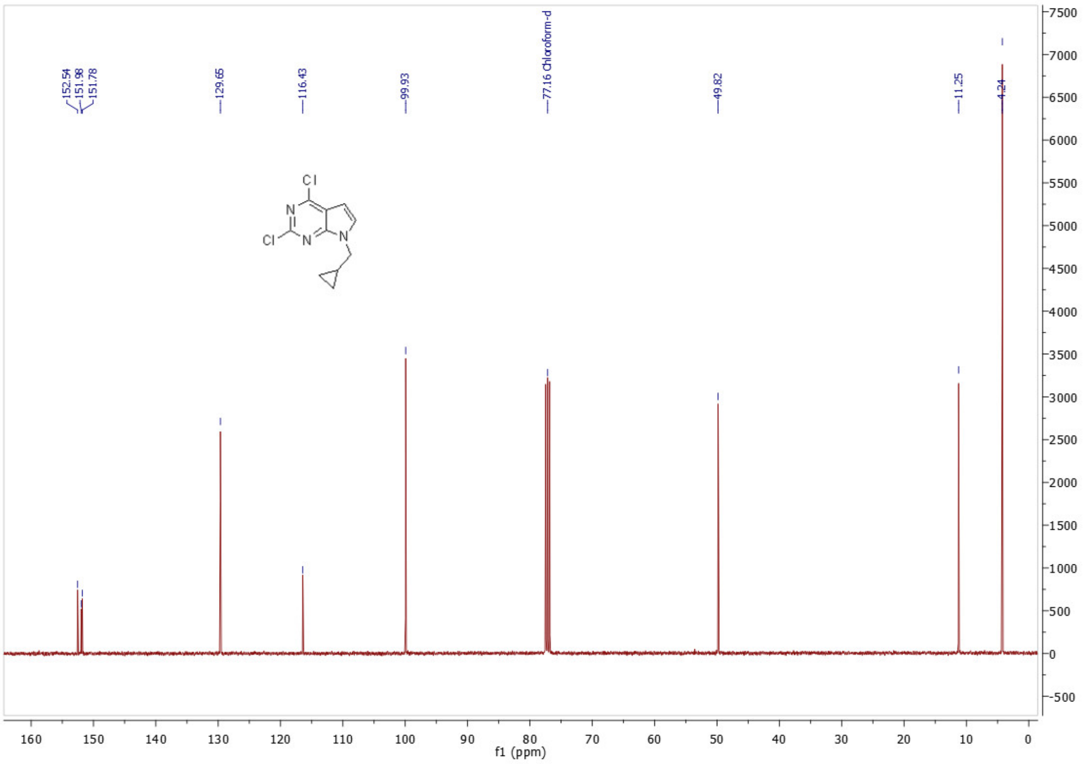

^{1}H NMR spectra of compound **15**

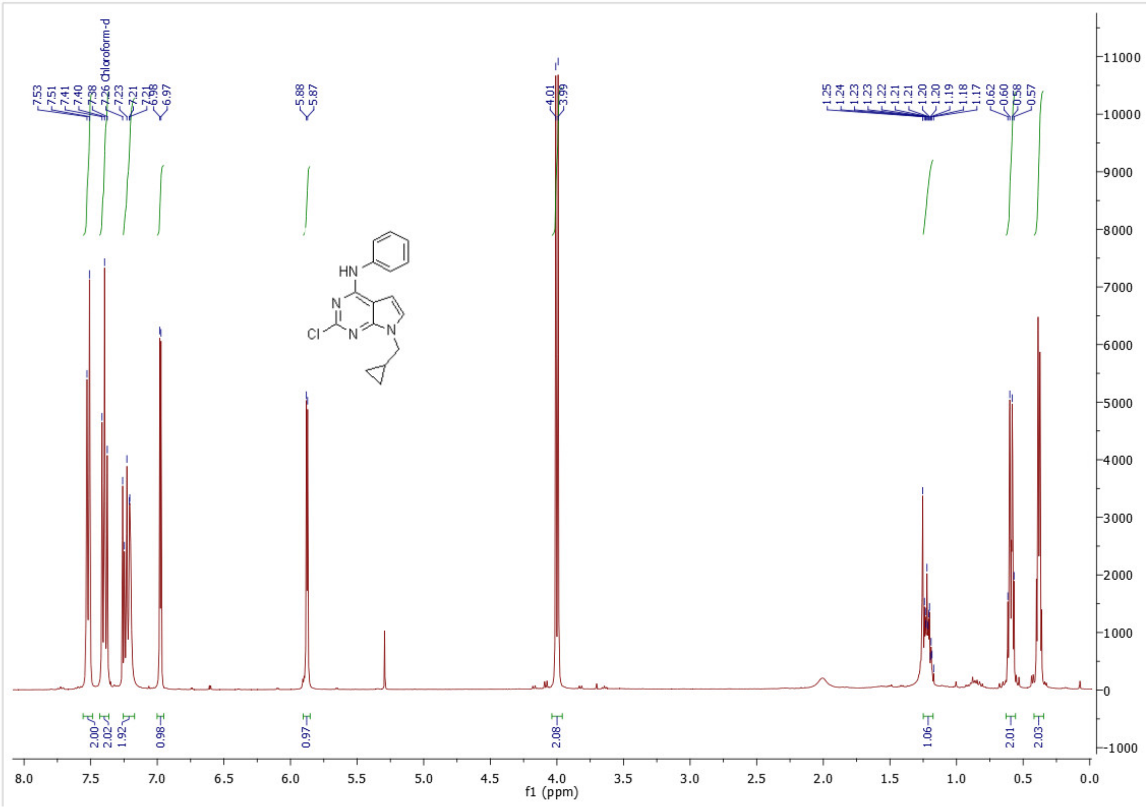

^{13}C NMR spectra of compound **15**

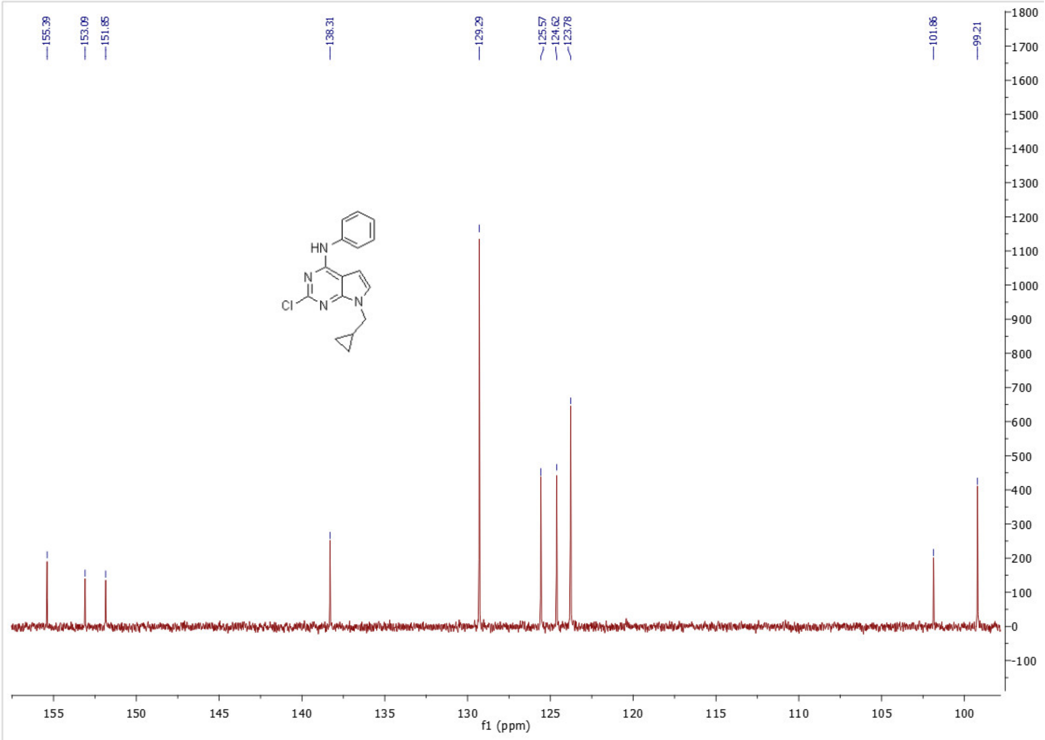

^{1}H NMR spectra of compound **16**

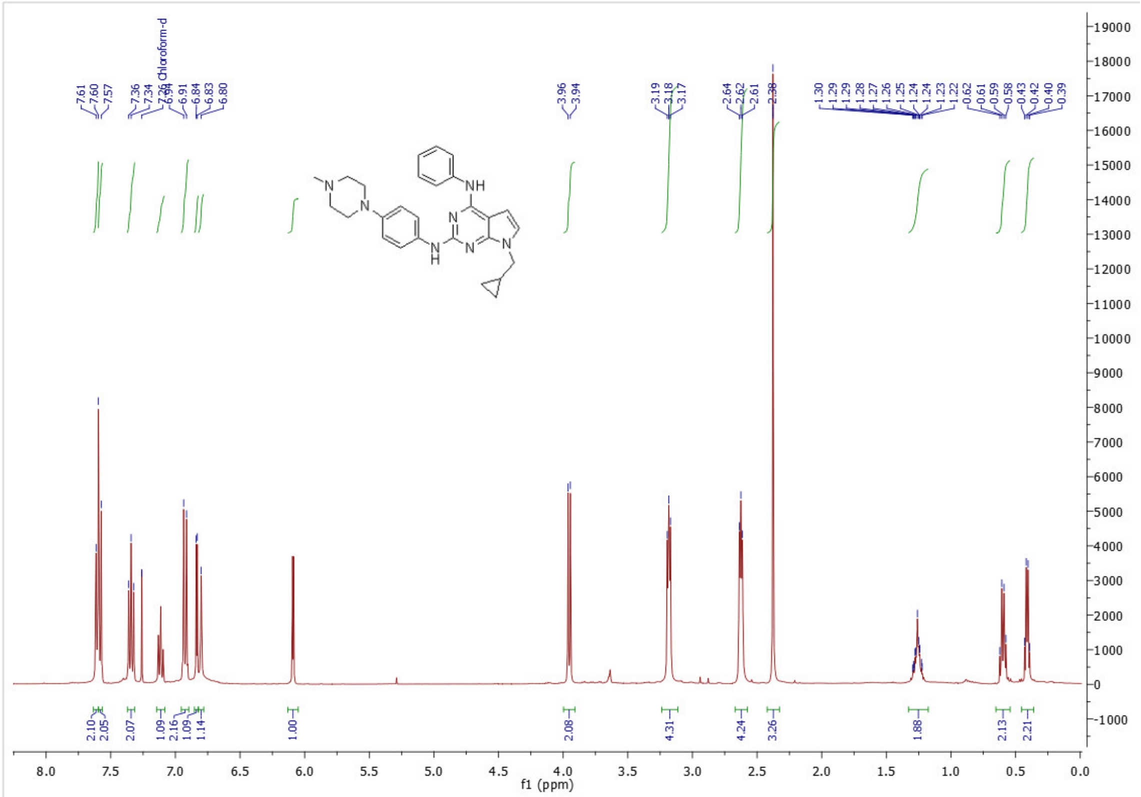

^{13}C NMR spectra of compound **16**

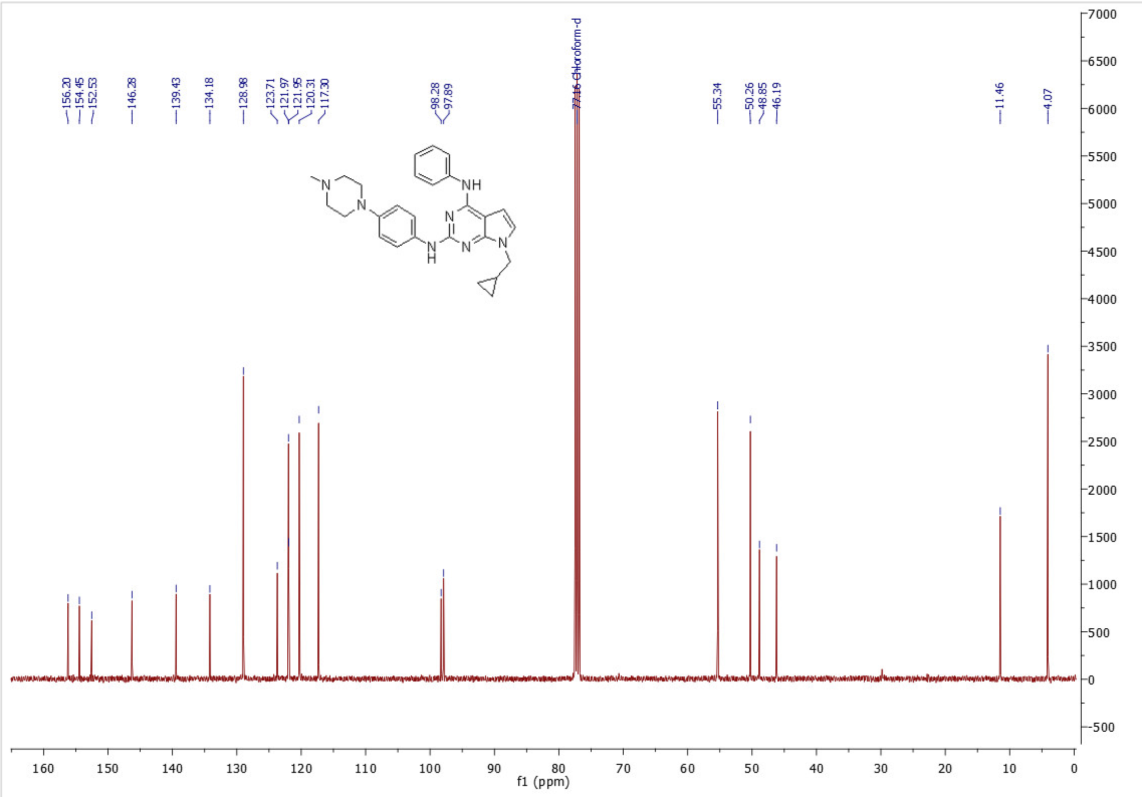

Mass spectra of compound **4a**

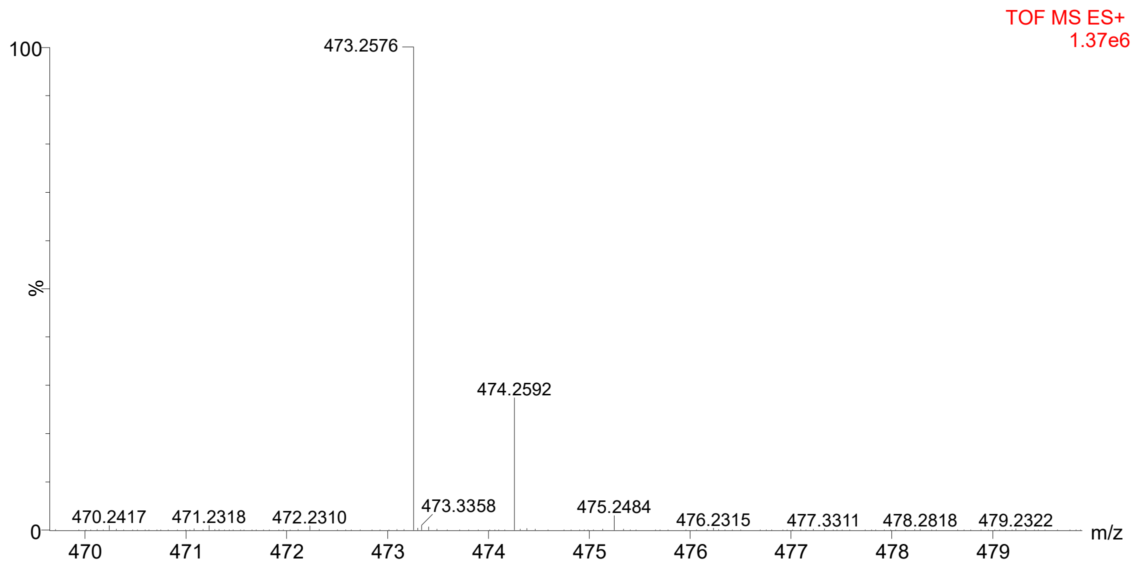

Mass spectra of compound **4b**

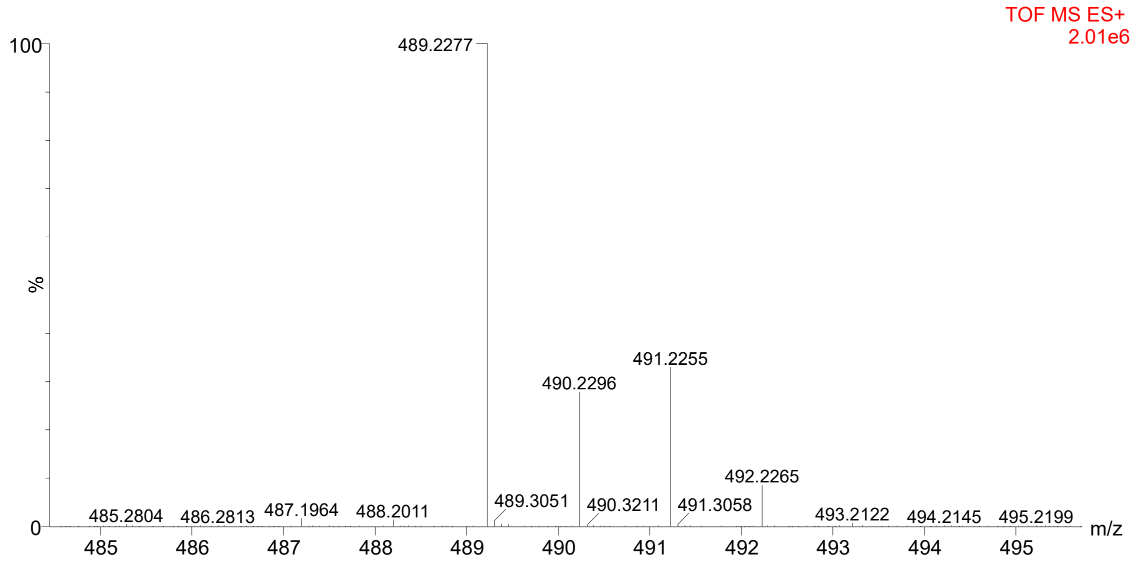

Mass spectra of compound **4c**

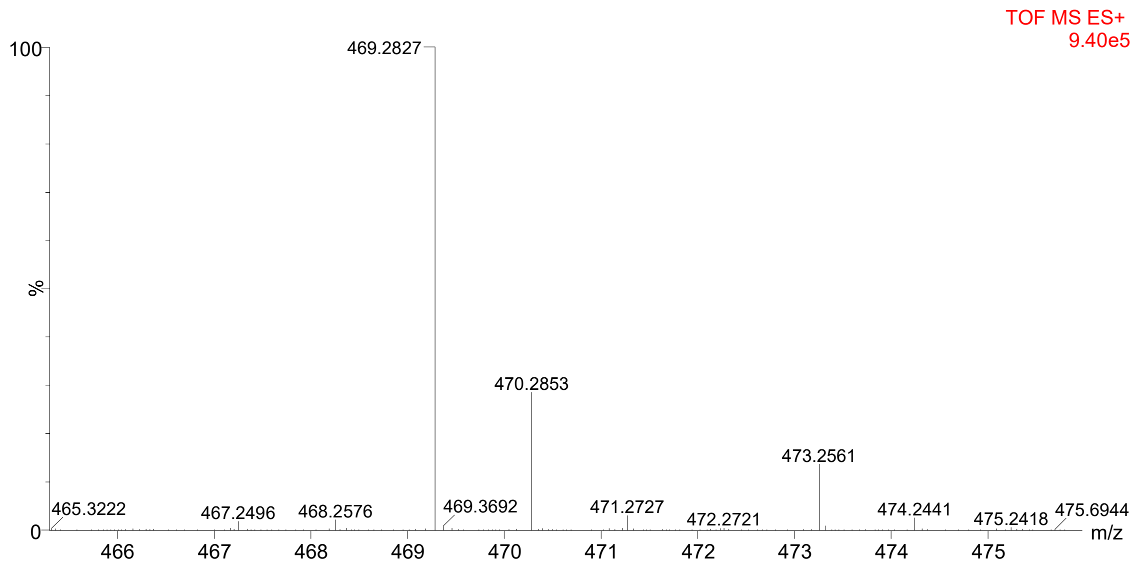

Mass spectra of compound **4d**

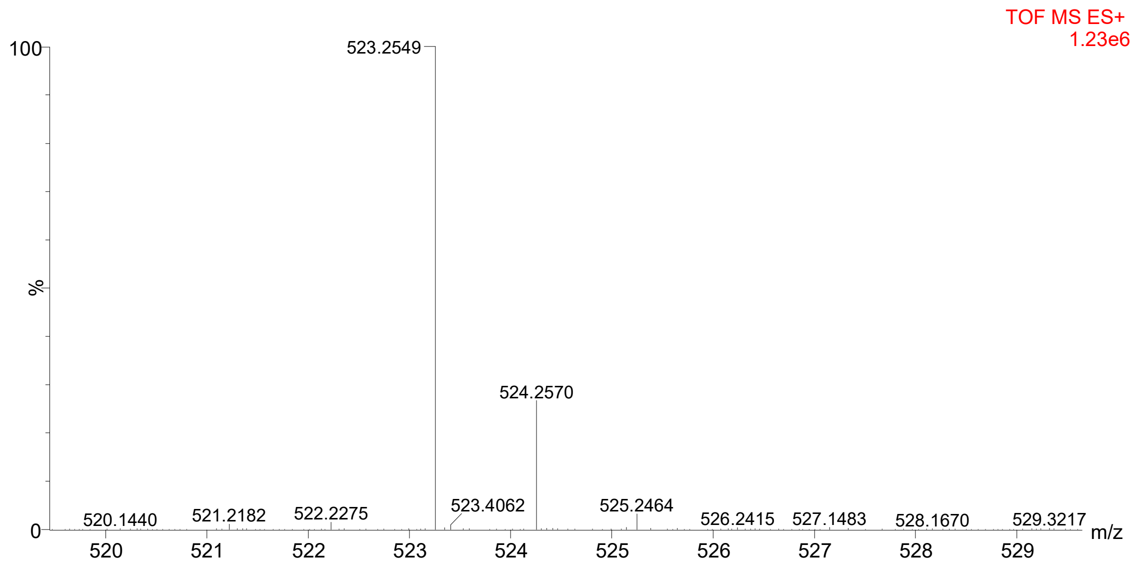

Mass spectra of compound **4e**

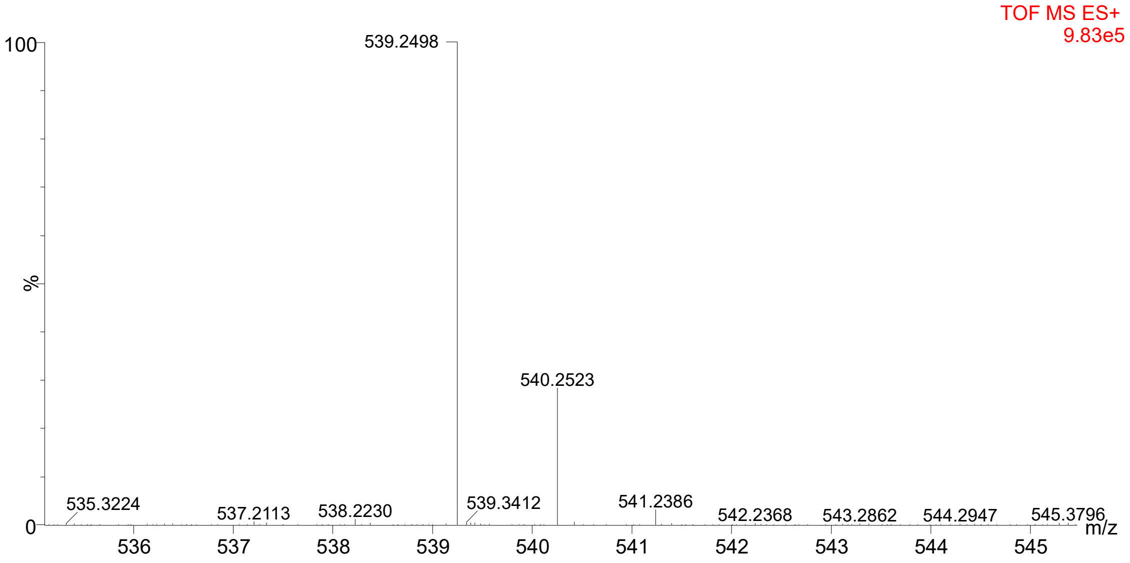

Mass spectra of compound **4f**

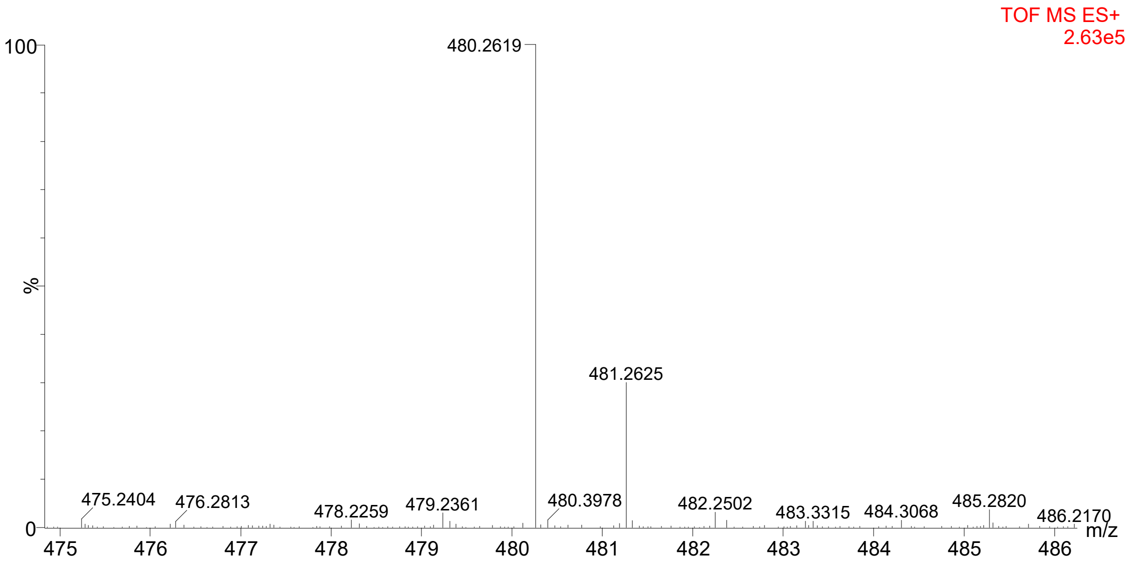

Mass spectra of compound **4g**

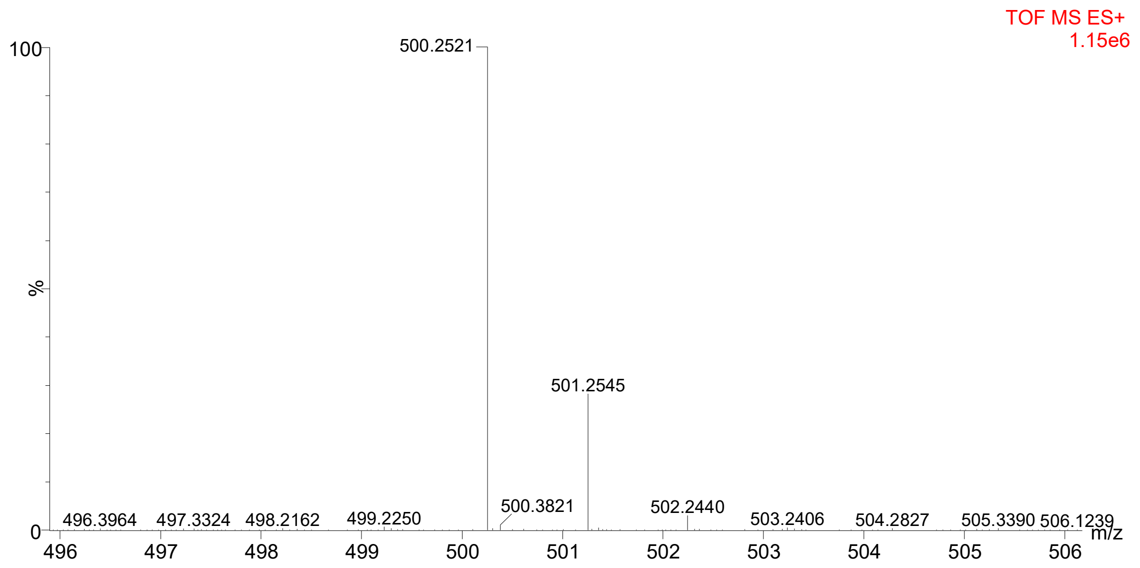

Mass spectra of compound **4h**

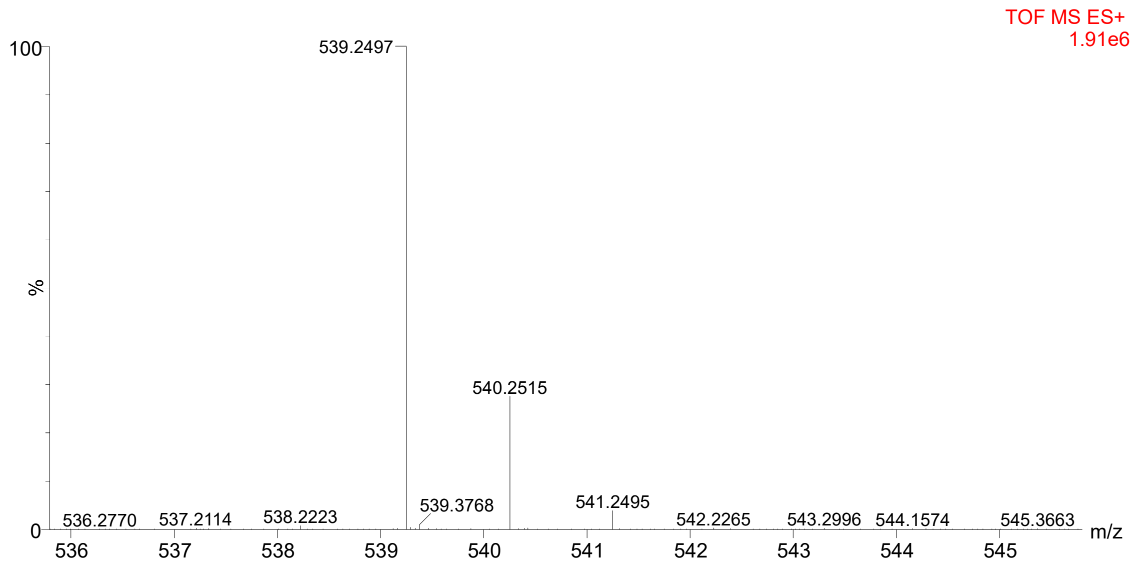

Mass spectra of compound **4i**

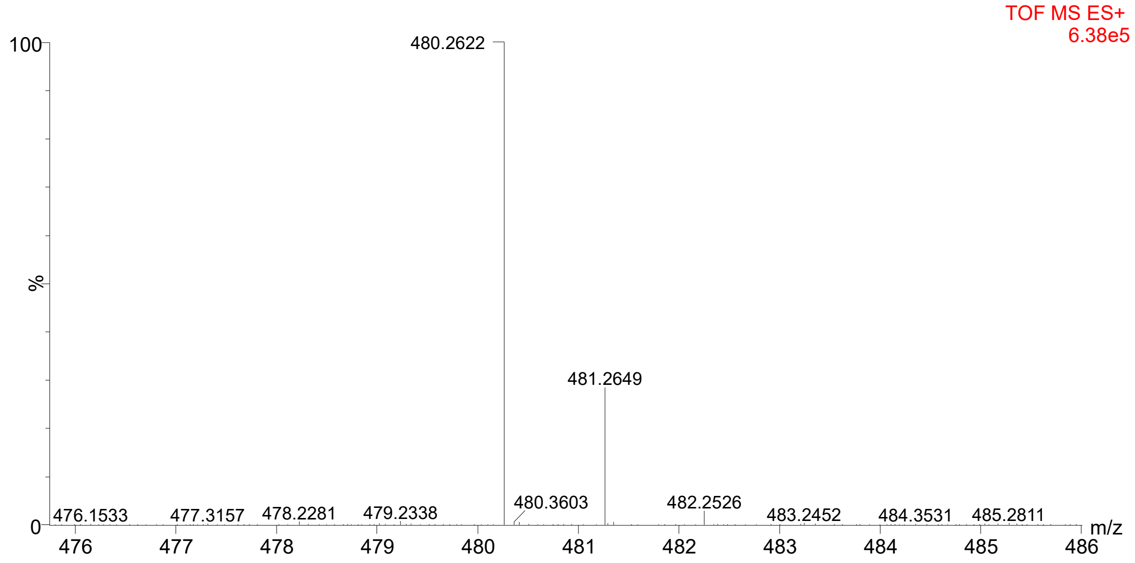

Mass spectra of compound **4j**

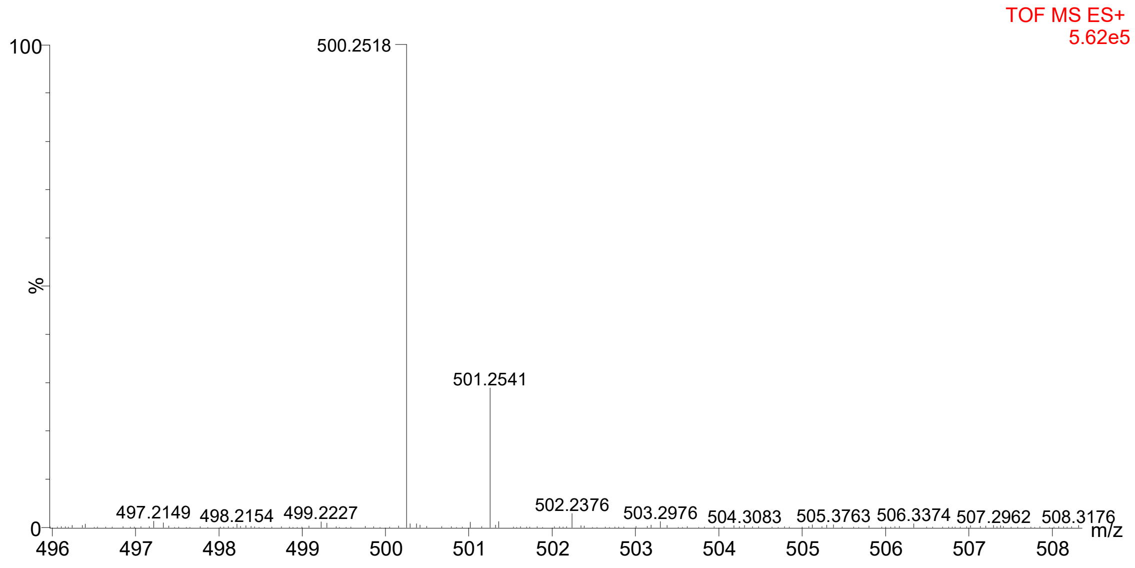

Mass spectra of compound **4k**

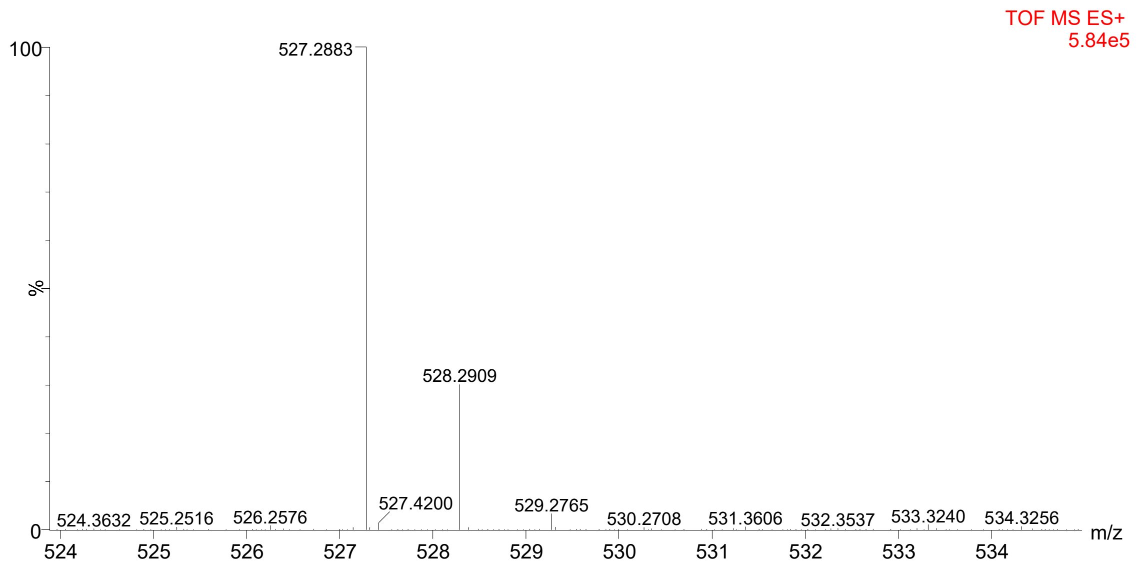

Mass spectra of compound **4l**

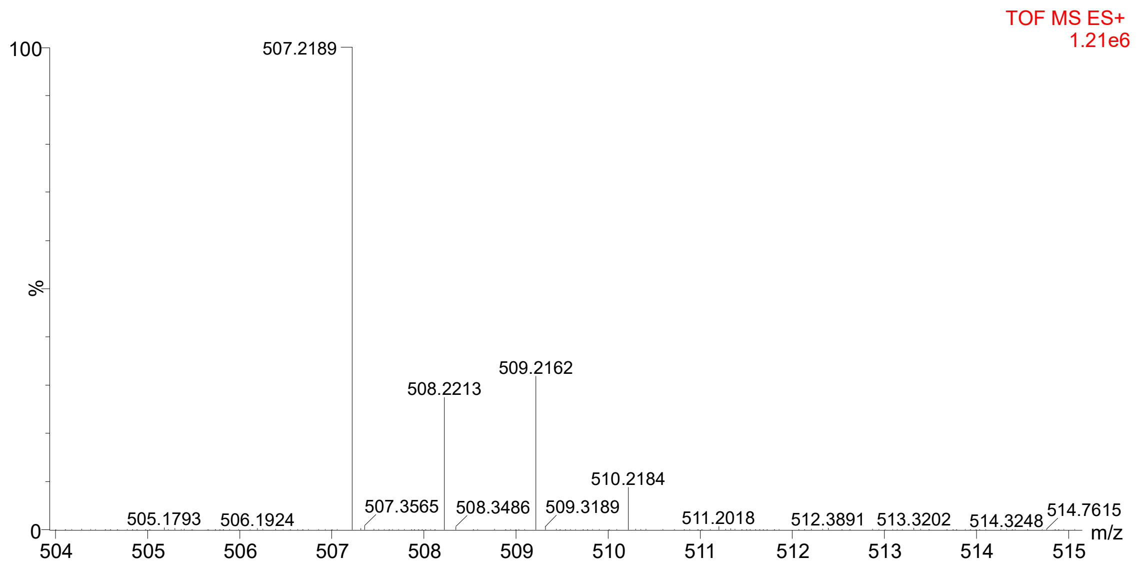

Mass spectra of compound **4m**

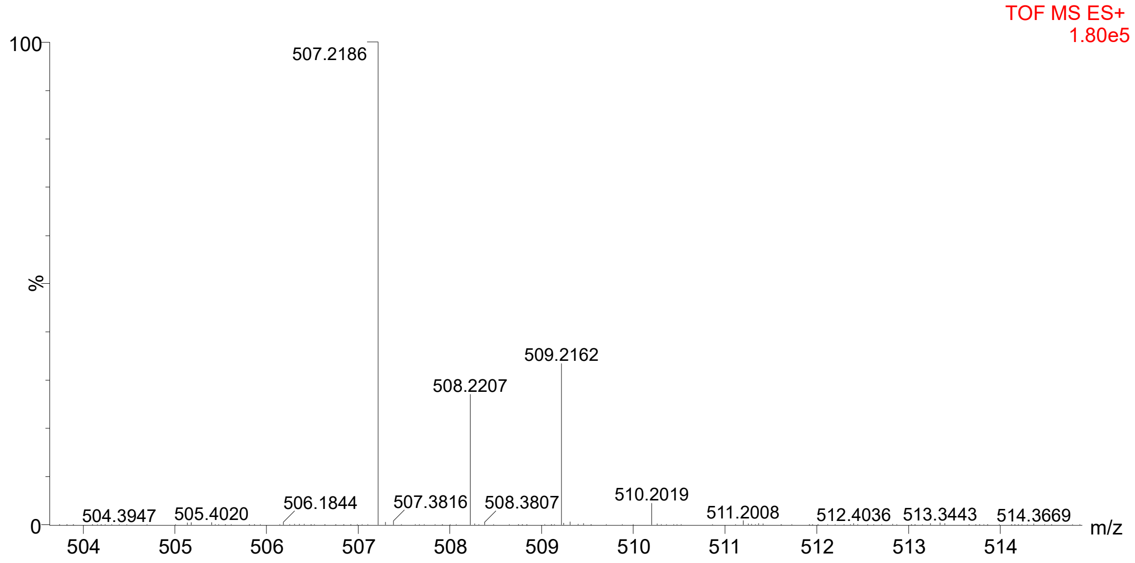

Mass spectra of compound **4n**

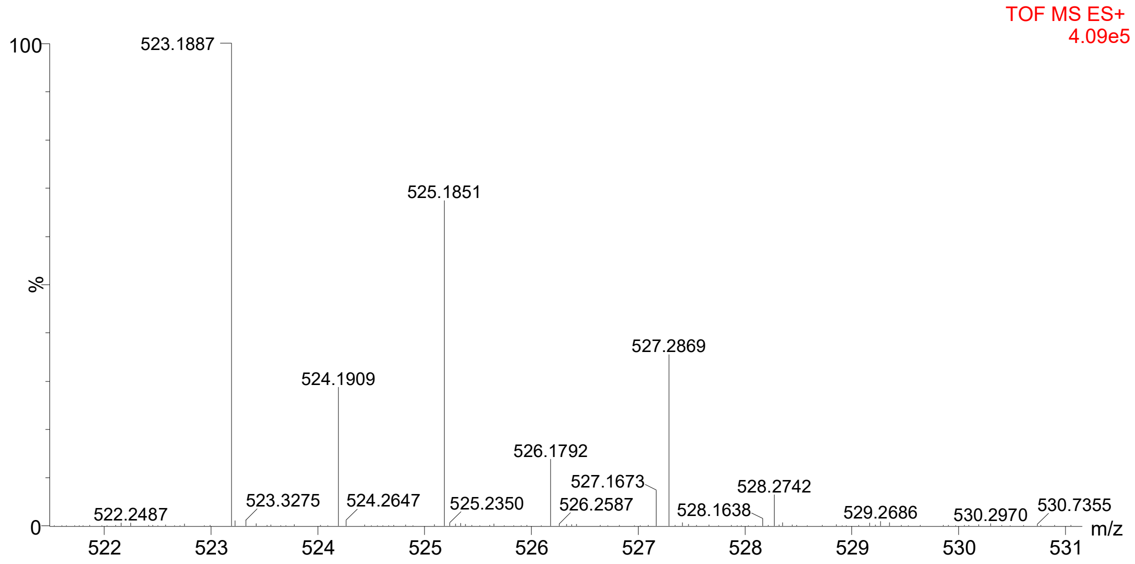

Mass spectra of compound **4o**

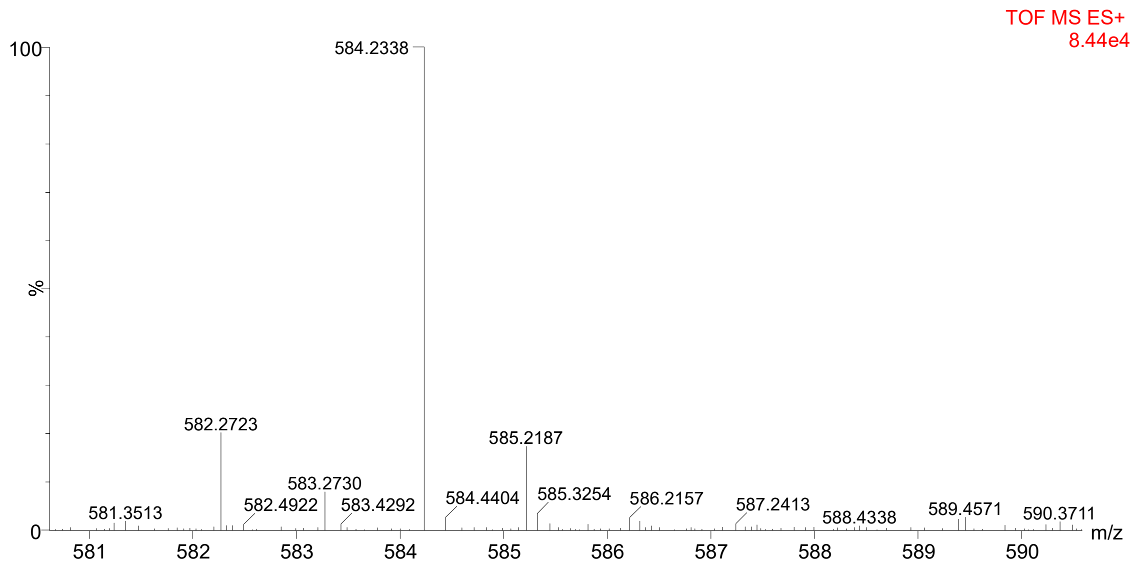

Mass spectra of compound **5a**

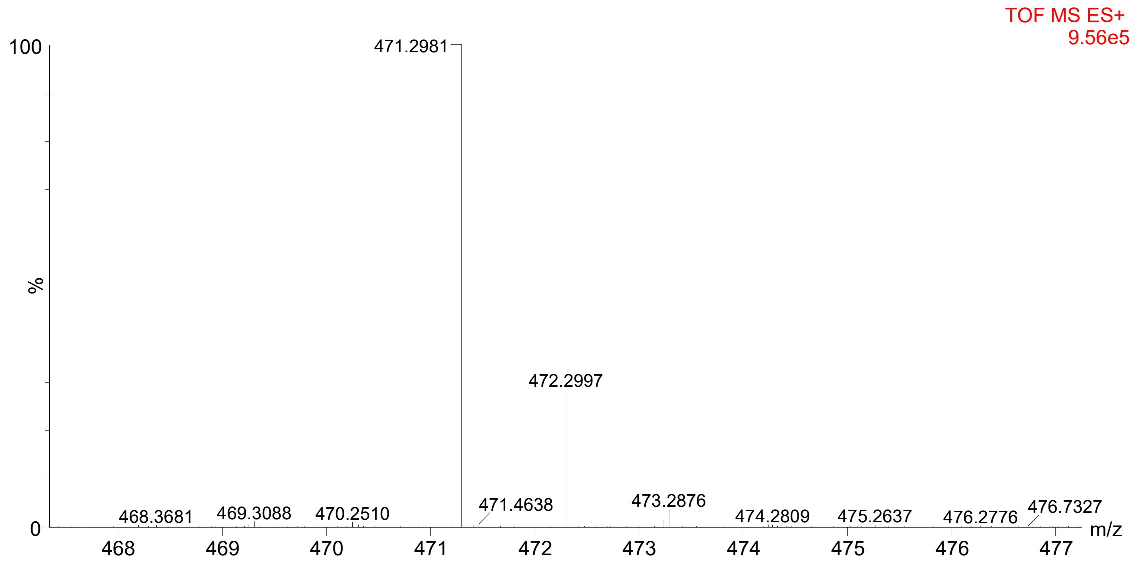

Mass spectra of compound **5b**

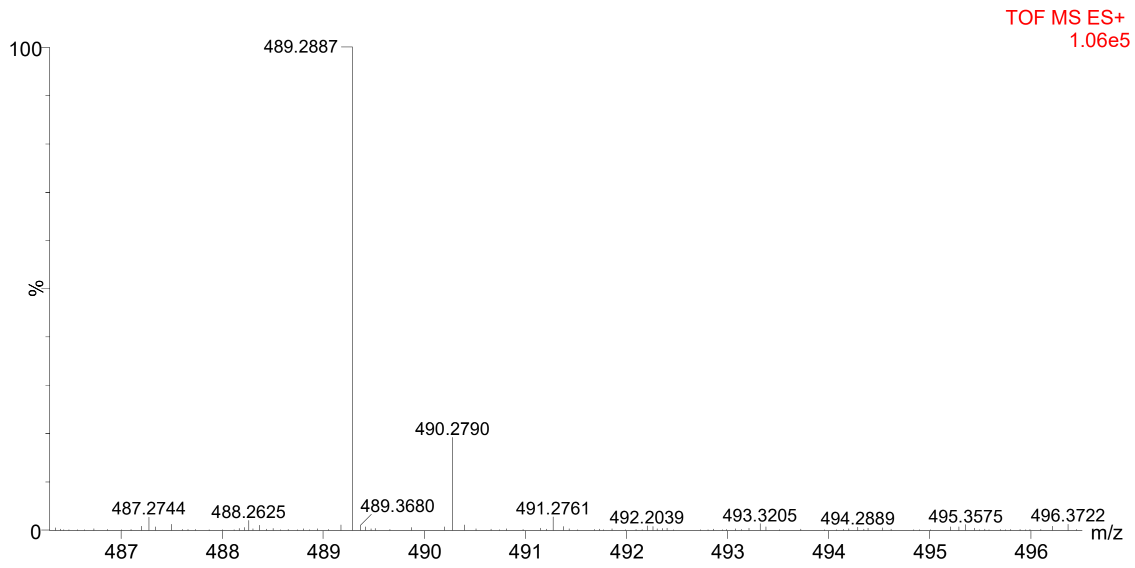

Mass spectra of compound **5c**

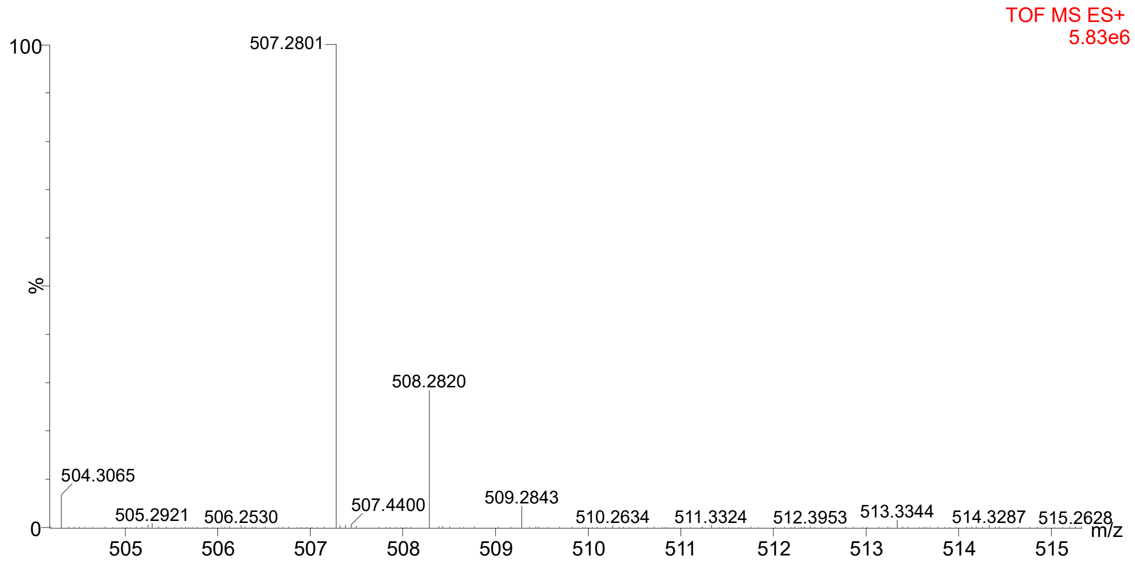

Mass spectra of compound **5d**

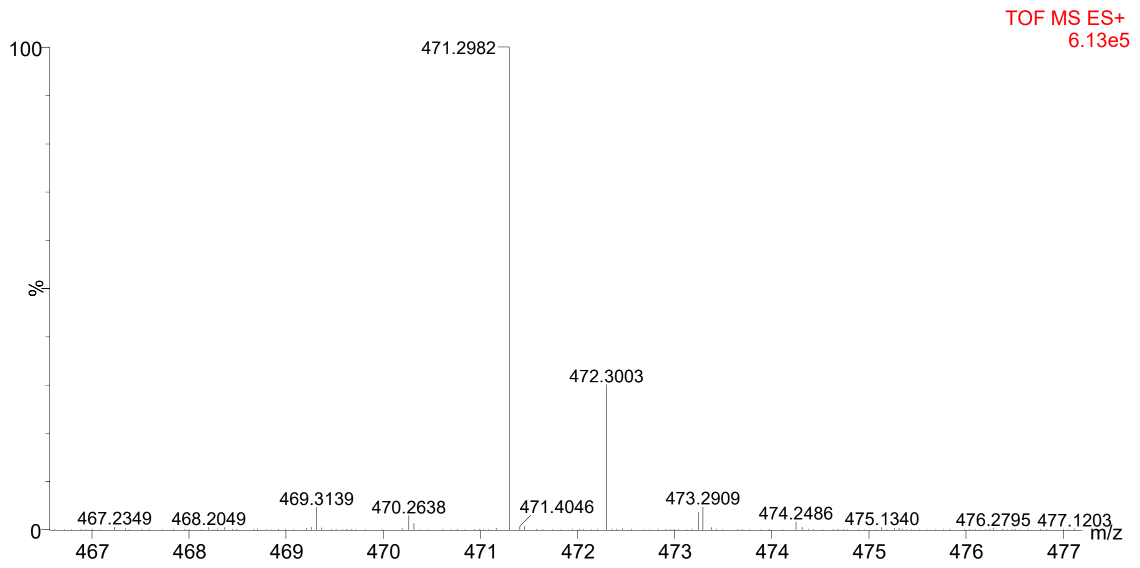

Mass spectra of compound **5e**

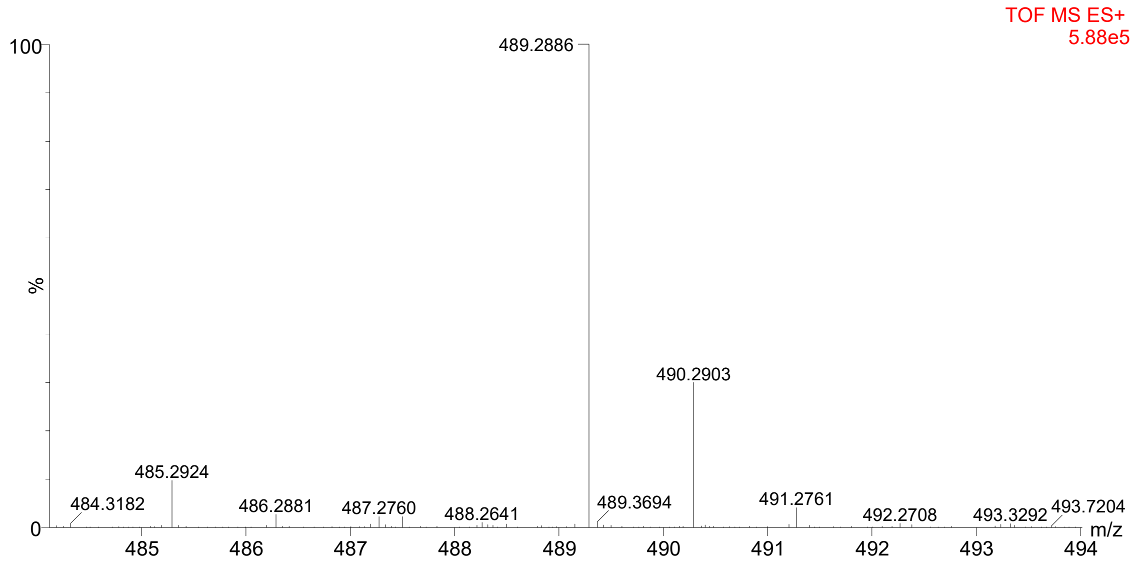

Mass spectra of compound **5f**

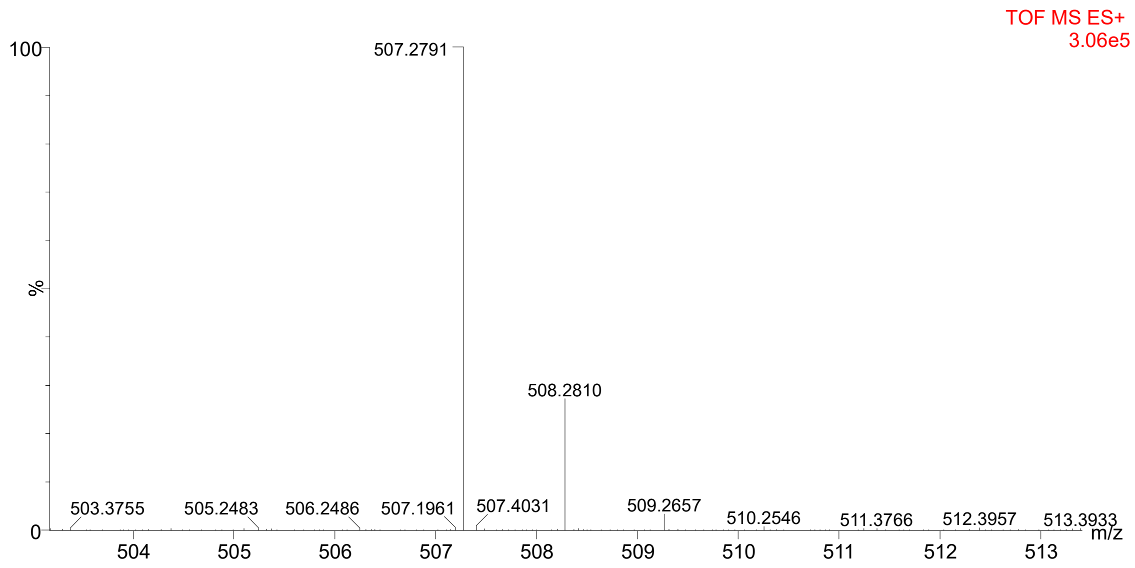

Mass spectra of compound **5g**

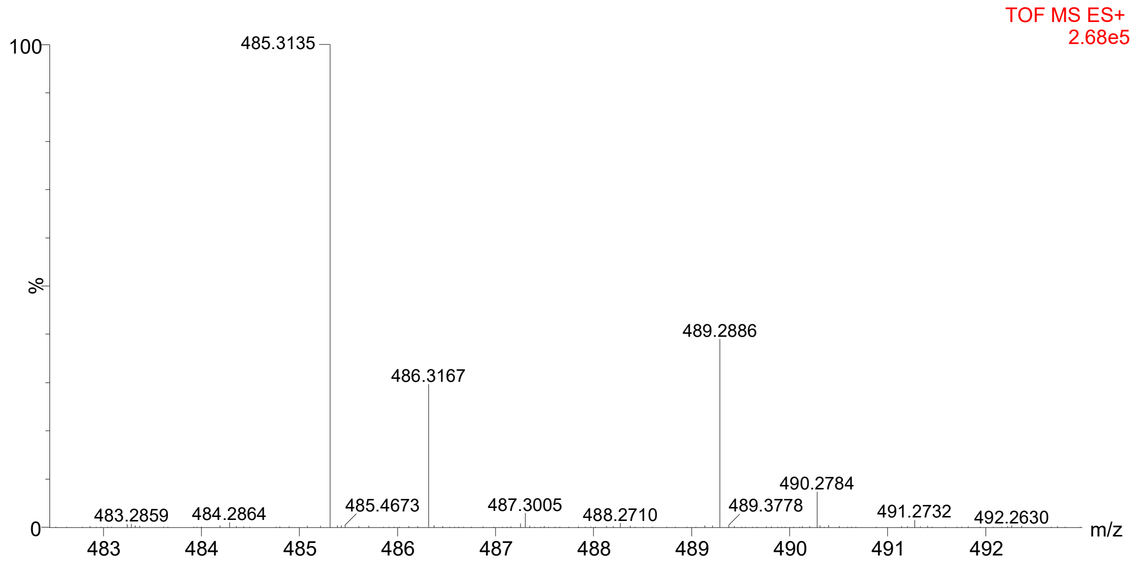

Mass spectra of compound **5h**

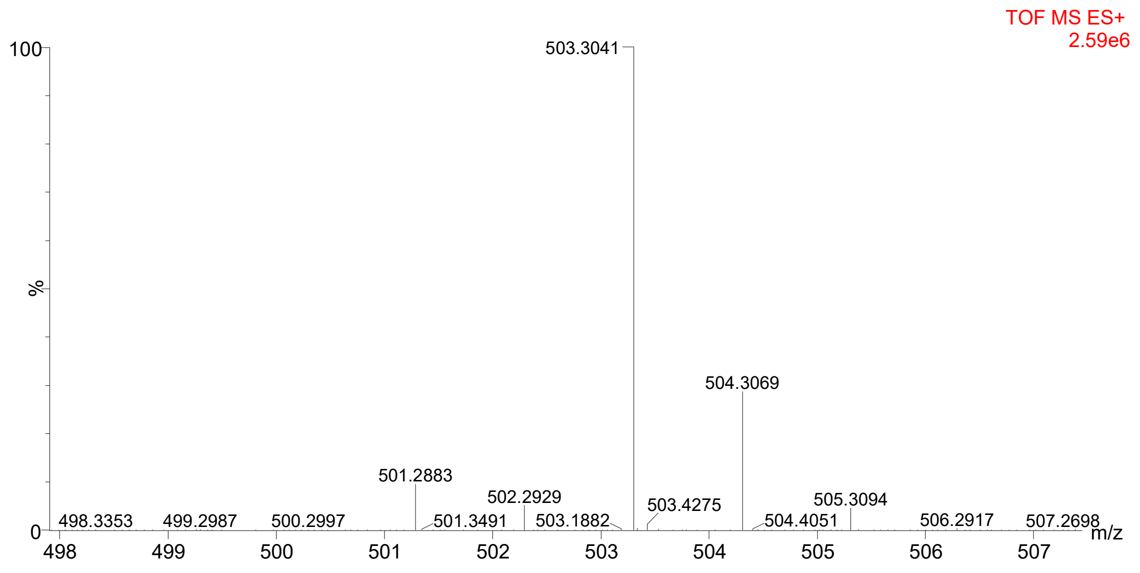

Mass spectra of compound **5i**

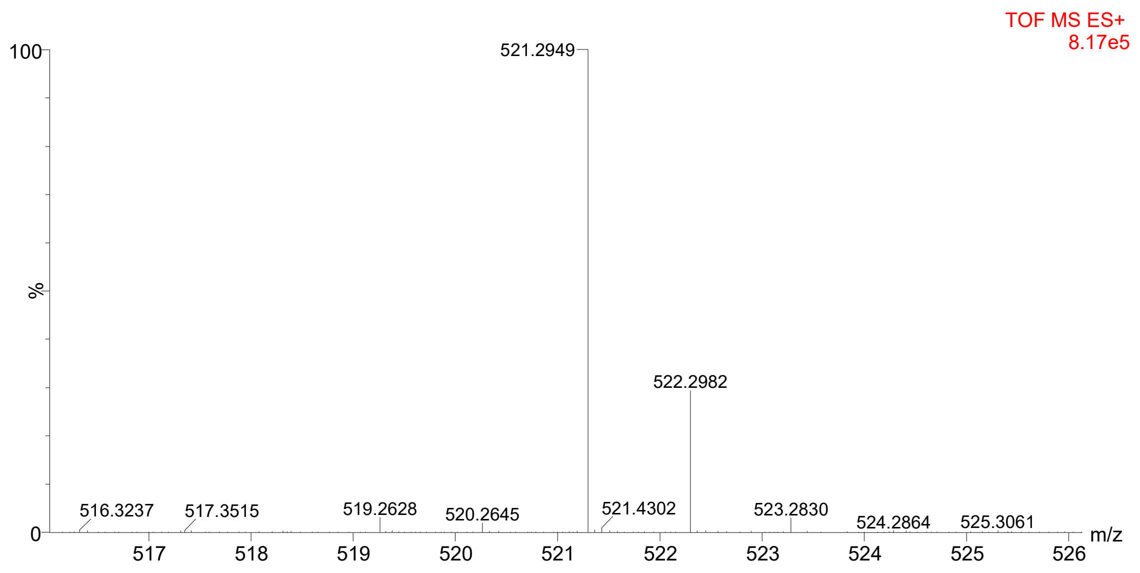

Mass spectra of compound **5j**

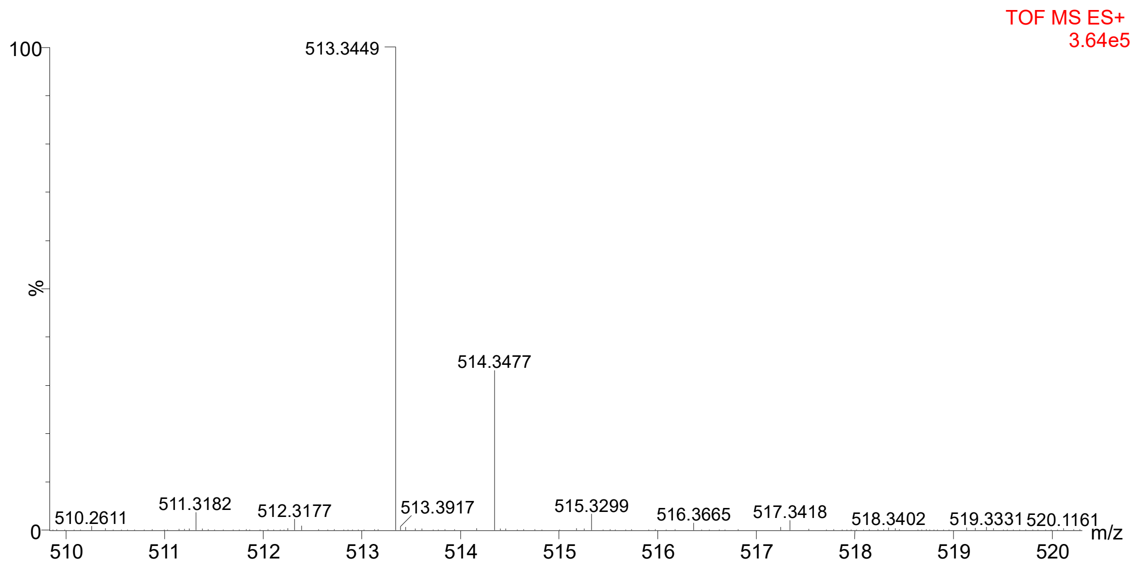

Mass spectra of compound **5k**

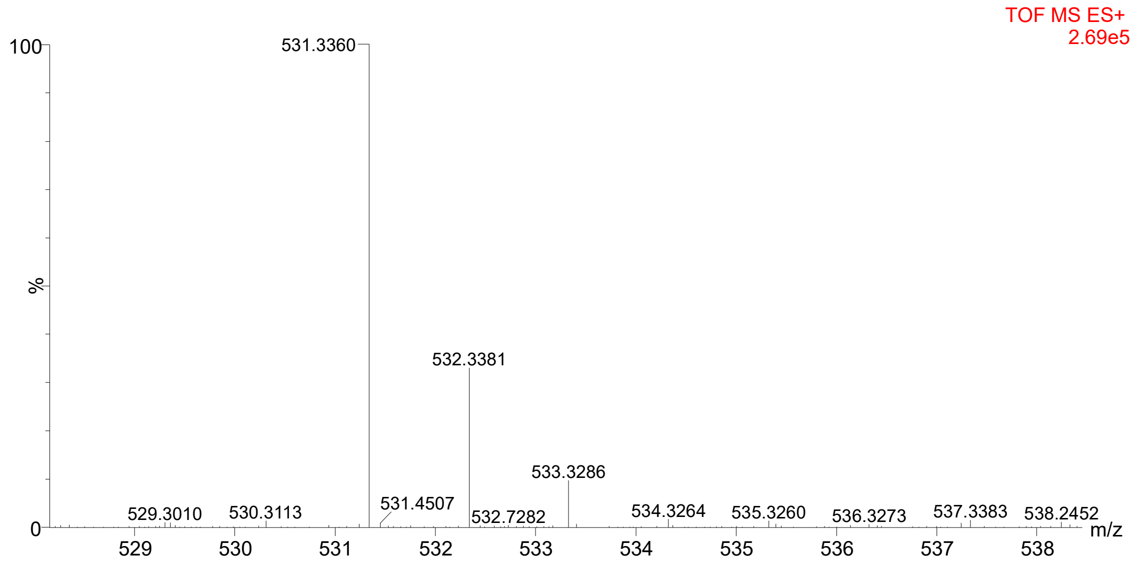

Mass spectra of compound **5l**

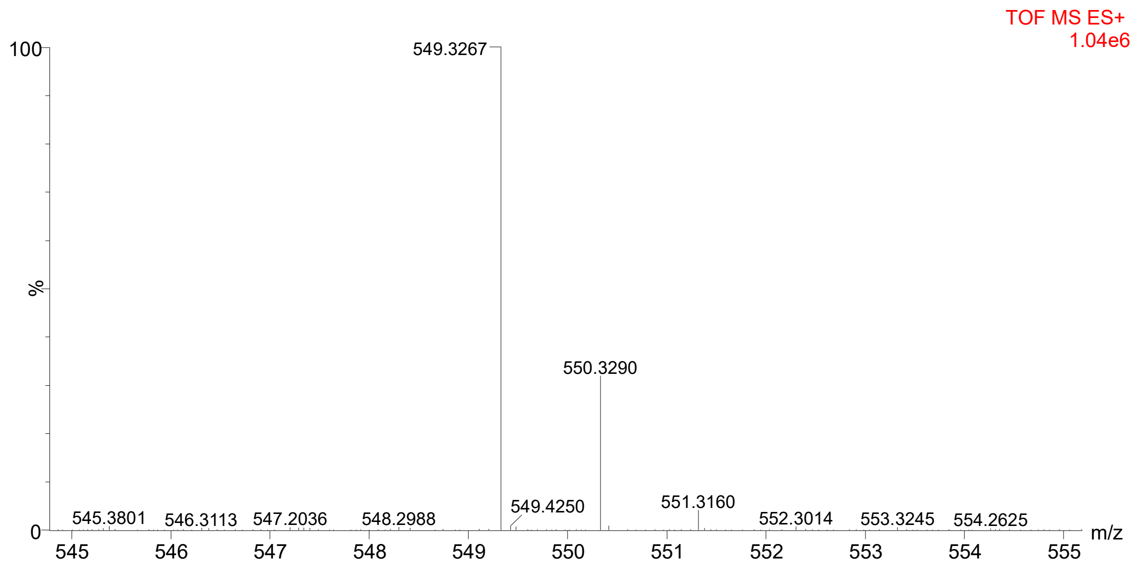

Mass spectra of compound **8a**

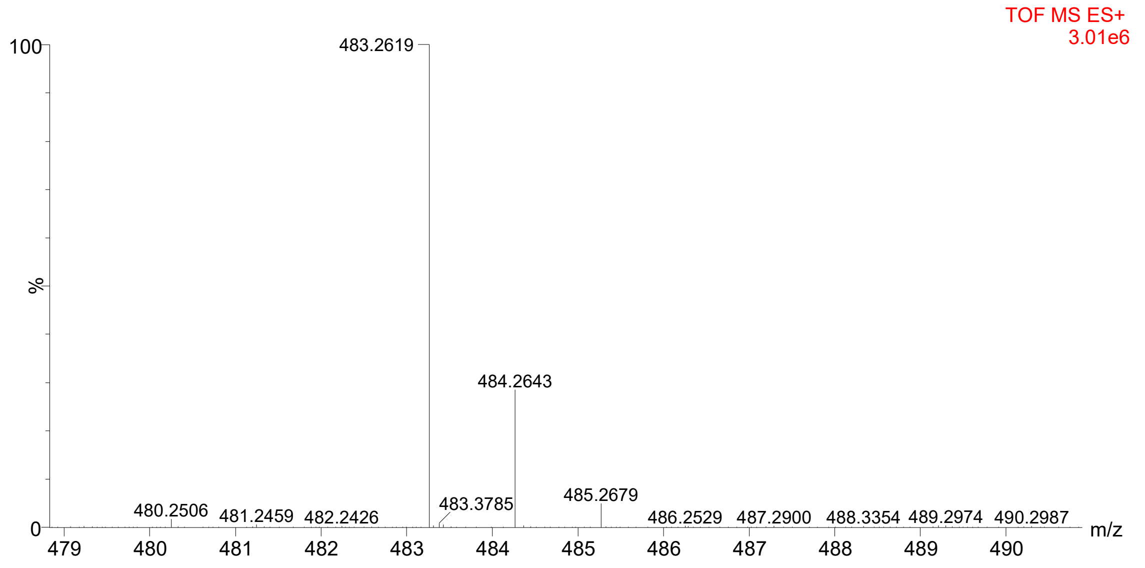

Mass spectra of compound **8b**

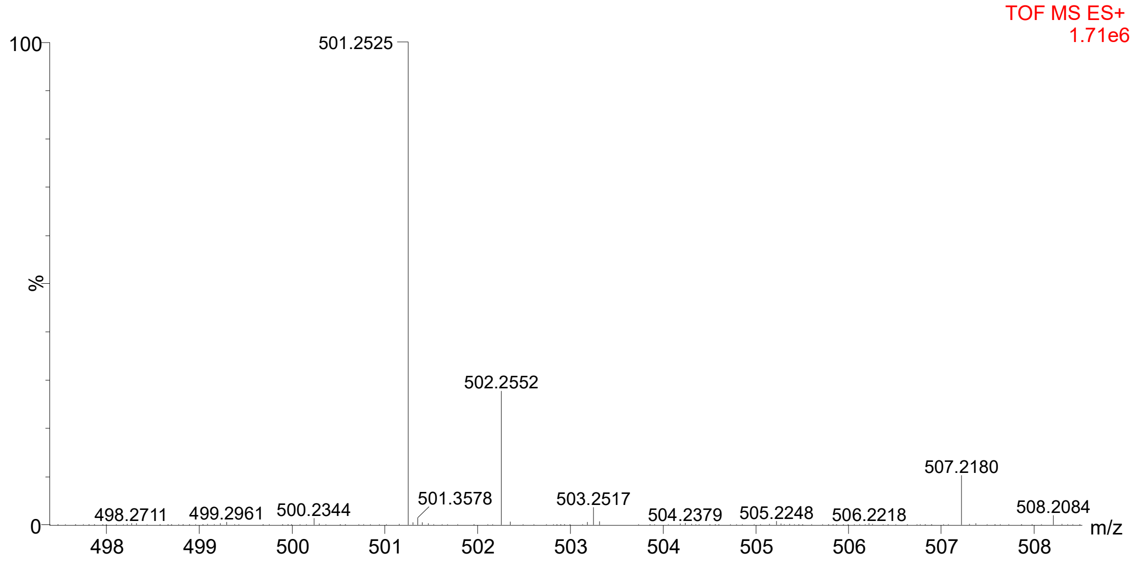

Mass spectra of compound **8c**

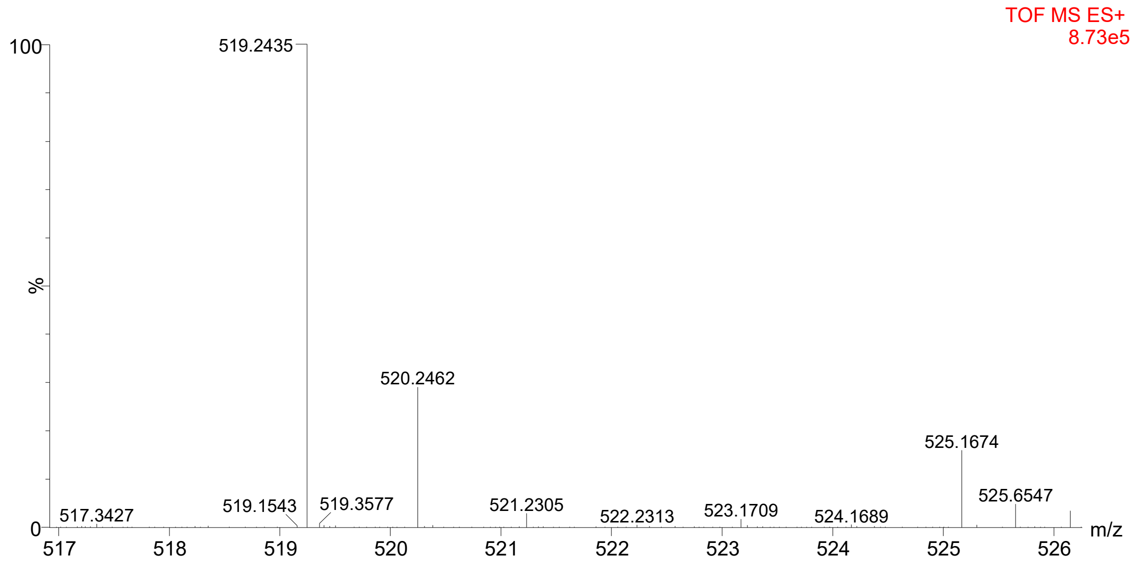

Mass spectra of compound **12**

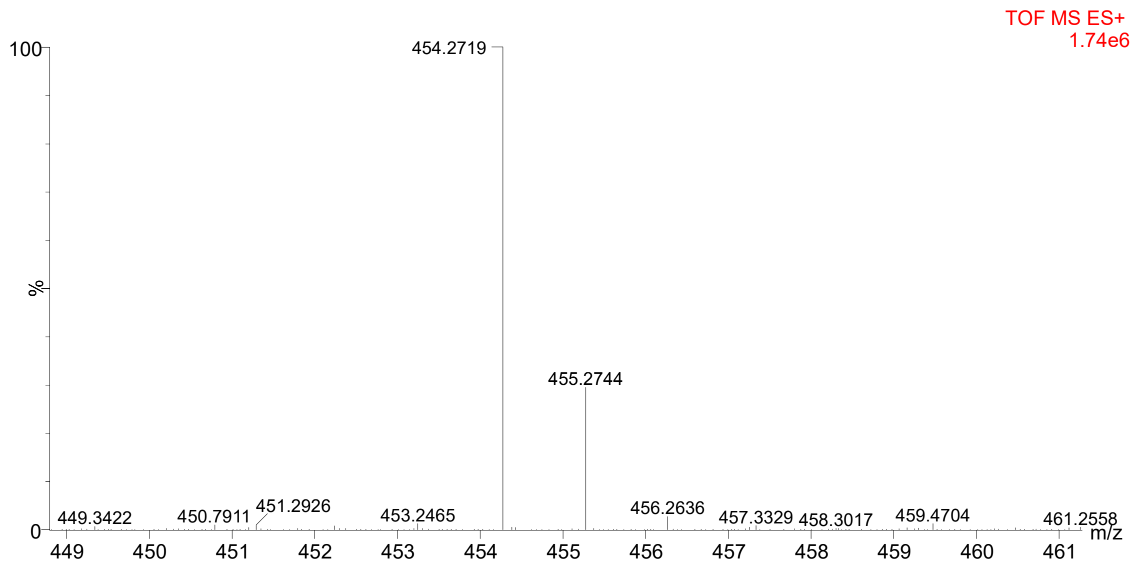

3D-QSAR details

Table S1. Statistical parameters for CoMFA analysis in Bcr-Abl, BTK and FLT3-ITD.

CoMFA	N	SEE	r^2	F	q^2	r^2_{test}	S	E
**Bcr-Abl**	5	0.402	0.902	34.829	0.901	0.885	0.615	0.385
**BTK**	6	0.202	0.874	20.868	0.874	0.625	0.444	0.556
**FLT3-ITD**	5	0.204	0.9	33.728	0.899	0.602	0.605	0.395

[a] N = The optimum number of components; SEE is the standard error of estimation of non-CV analysis; r^2_{ncv} is the square of the non CV coefficient; F is the F-test value; q^2 = the square of the LOO cross-validation (CV) coefficient; r^2_{test}= the regression coefficient for test set; S, and E, are the steric, and electrostatic contributions, respectively.

Table S2. Summary of external validation parameters for CoMFA models.

Condition	Parameters	Threshold value	Bcr-Abl	BTK	FLT3-ITD
1	q^2	>0.5	0.901	0.874	0.899
2	r^2	>0.6	0.885	0.625	0.602
3a	r_0^2	Close to value of r^2	0.999	0.999	0.999
3b	r'^2_0	Close to value of r^2	0.999	0.999	0.999
4a	k	$0.85 < k < 1.15$	1.019	0.949	0.976
4b	k'	$0.85 < k' < 1.15$	0.980	1.053	1.023

q^2 is the same parameter as listed in table 2; r^2 is the regression coefficient for the test set; r_0^2 and k are the correlation coefficient between the *experimental* and *predicted* activities for test set and the respective slope of regression; and $r_0'^2$ and k' are the correlation coefficient between the *predicted* and *experimental* activities for test set and the respective slope of regression.

Table S3. Experimental and predicted pIC_{50} and residual values for analyzed compounds according to CoMFA.[a]

Molecule	CoMFA ABL			CoMFA BTK			CoMFA FLT3		
	Experimental pIC_{50}	Predicted pIC_{50}	Residual	Experimental pIC_{50}	Predicted pIC_{50}	Residual	Experimental pIC_{50}	Predicted pIC_{50}	Residual
**1**	5.3774	5.797	-0.419	4.8328	5.088	-0.256	4.7124	5.181	-0.469
**2**	7.1427	7.064	0.079	5.8459	6.03	-0.184	*6.0313*	*5.768*	*0.26*
**3**	6.983	6.802	0.181	5.8877	5.631	0.257	*5.823*	*5.825*	*0.00*
**4**	6.9066	6.593	0.314	*5.8943*	*5.569*	*0.325*	5.9502	5.706	0.244
**5**	5.7508	6.115	-0.364	4.6445	4.716	-0.072	*5.7442*	*5.849*	*-0.10*
**6**	*5.9402*	*5.781*	*0.16*	5.3854	5.292	0.094	5.6342	5.782	-0.147
**7**	6.7761	6.477	0.299	5.8668	5.583	0.283	5.9041	5.724	0.18
**8**	*6.4067*	*6.59*	*-0.18*	6.2192	6.169	0.05	6.127	5.899	0.228
**9**	6.89	6.991	-0.101	6.0276	6.177	-0.149	6.2725	6.202	0.07
**10**	7.0047	7.211	-0.206	6.040	6.071	-0.031	6.4353	6.275	0.161
**11**	6.8788	6.795	0.084	5.9359	5.776	0.16	5.9355	5.974	-0.038
**12**	6.5662	6.742	-0.176	5.6211	5.595	0.026	5.8129	5.654	0.159
**13**	6.3951	6.905	-0.51	5.4089	5.731	-0.322	5.5967	5.833	-0.236
**14**	6.699	6.183	0.516	6.0577	5.858	0.2	*5.9878*	*5.615*	*0.37*
**15**	*6.3612*	*6.935*	*-0.57*	5.2983	5.372	-0.073	5.5743	5.908	-0.334
**16**	5.9169	6.181	-0.264	5.2213	5.487	-0.266	5.7111	5.768	-0.057
**17**	6.246	5.964	0.282	5.7637	5.566	0.198	5.796	5.78	0.016
**18**	*6.0259*	*6.05*	*-0.02*	5.5634	5.491	0.073	5.7435	5.708	0.035
**19**	4.0733	4.149	-0.076	6.2364	6.056	0.181	4.699	4.654	0.045
**20**	4.000	3.946	0.054	*6.0121*	*5.926*	*0.086*	4.699	4.601	0.098
**21**	4.301	5.084	-0.783	5.423	5.636	-0.213	6.4185	6.277	0.141
**22**	*5.911*	*5.941*	*-0.03*	*6.3747*	*5.853*	*0.52*	6.3377	6.214	0.124
**23**	5.7501	5.249	0.501	5.6533	5.444	0.209	*6.6478*	*6.226*	*0.42*
**24**	*5.8667*	*5.858*	*0.01*	6.0313	6.045	-0.014	5.992	6.166	-0.174
**25**	5.008	4.802	0.206	6.0388	6.228	-0.189	5.7198	5.733	-0.013
**26**	4.000	4.151	-0.151	*6.3915*	*5.96*	*0.43*	4.693	4.782	-0.089
**27**	4.4689	4.357	0.112	6.3391	6.369	-0.03	*5.5624*	*5.71*	*-0.15*
**28**	4.000	4.643	-0.643	6.4942	6.448	0.046	5.636	5.788	-0.152
**29**	6.1898	5.744	0.446	*6.4455*	*6.001*	*0.44*	6.3045	6.209	0.095
**30**	5.6426	5.237	0.406	*5.724*	*5.675*	*0.05*	6.1032	6.206	-0.103
**31**	4.2057	3.992	0.214	4.699	4.677	0.022	4.699	4.483	0.216

[a]Test set compounds for each enzyme are in cursive.

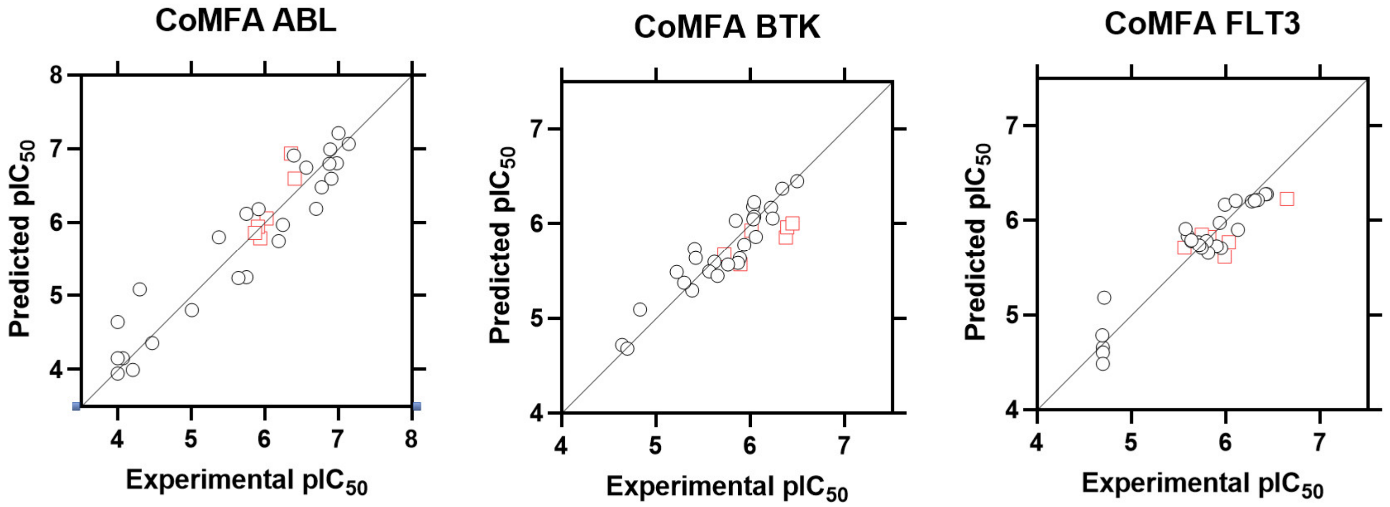

Figure S1. Plots of experimental pIC_{50} versus predicted pIC_{50} for CoMFA in ABL, BTK and FLT3. In black circles the training set. In red squares the test set.

1. Docking protocol

1.1 Co-crystallized ligand preparation

For the self-docking studies, the molecule (inhibitor compounds) that were co-crystallized with the kinases were extracted and prepared for docking by adding hydrogen atoms, protonating the amine of the piperazines, and adding charges using the LigPrep tool implemented in the Schrödinger software (version 2021-1).[1]

1.2 Synthesized ligand preparation

The synthesized ligands were modeled with Maestro software, these compound hydrogens were added, and partial charges were assigned using the OPLS4 force field with LigPrep tool.[1] Then, the ionization state was generated with Epik module.[1]

1.3 Protein preparation

All crystal kinases were imported into Maestro software from the PDB database (www.rcsb.org (Accessed 05 March 2021)). For each crystal complex, all water molecules, salt, and organic solvent were deleted. The Protein Preparation Wizard of Schrödinger software was subsequently used for further preparations of the proteins.[1] Next, bond orders were assigned, and hydrogen atoms were added; additionally, the hydrogen bond lattice was optimized, and the protonation state of the protein was determined at pH 7.0 using PROPKA module. Finally, a constrained energy minimization step was carried out using the OPLS4 force field for each system.[1]

1.4 Grid preparation and molecular docking

The receptor grid for each protein structure was generated using the Receptor Grid Generation module implemented in Schrödinger Suite. In this step, the center of the co-crystallized ligand for each crystal structure corresponded to the grid box centroid. Next, the molecular docking using Glide in the standard precision mode (SP-score) was calculated by redocking the co-crystallized ligand with the binding site of its crystallized protein. Finally, the best poses were filtered with extra precision mode (XP-score) where the best result was the one closest to the pose of the co-crystallized ligand.[2]

An additional step was added to the synthesized ligands (**4i**, **5b** and **5j**). The best with XP-scored poses were refined with MM-GBSA method, considering the flexibility of the nearby amino acids of the docked ligands poses at 6 Å distance (**Figure S2**).

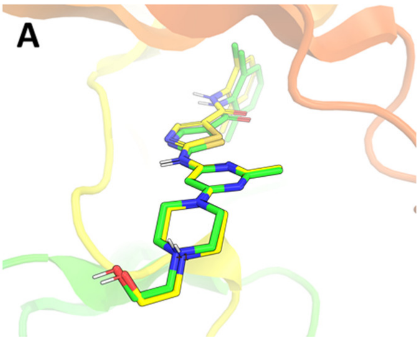

Bcr-Abl protein
PDB ID: 2GQG
RMSD$_{self\text{-}docking}$: 0.76 Å

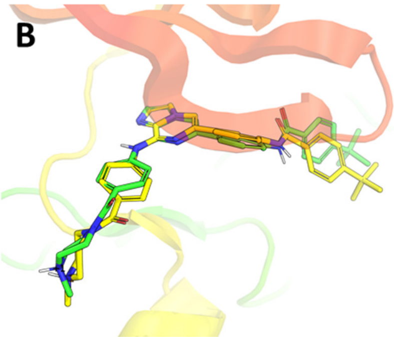

BTK protein
PDB ID: 4OT5
RMSD$_{self\text{-}docking}$: 1.31 Å

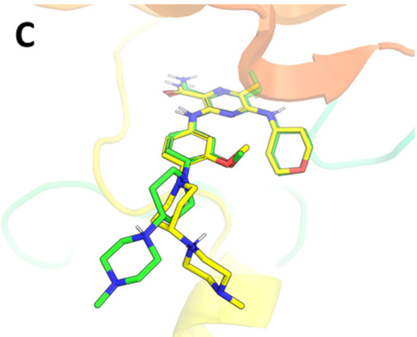

FLT3 protein
PDB ID: 6JQR
RMSD$_{self\text{-}docking}$: 2.27 Å

Figure S2. Co-crystallized ligands with their experimentally determined binding mode are shown in green, while the docking pose of the ligands from our self-docking protocol are shown in yellow.

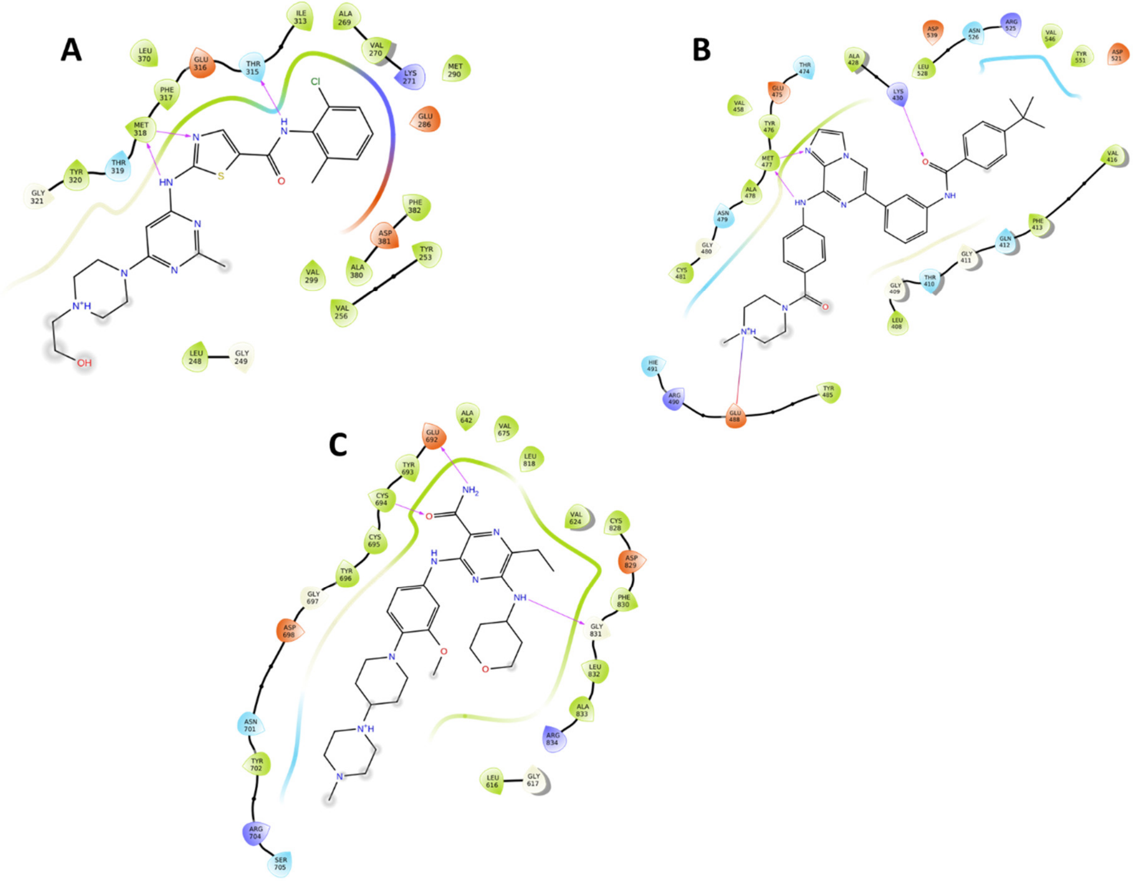

Figure S3. Schemes of the essential interactions reproduced by the self-docking poses. The important hydrogen bonds are amino acids M318, M477 and C694 for Abl (**A**), BTK (**B**), and FLT3 (**C**), respectively.

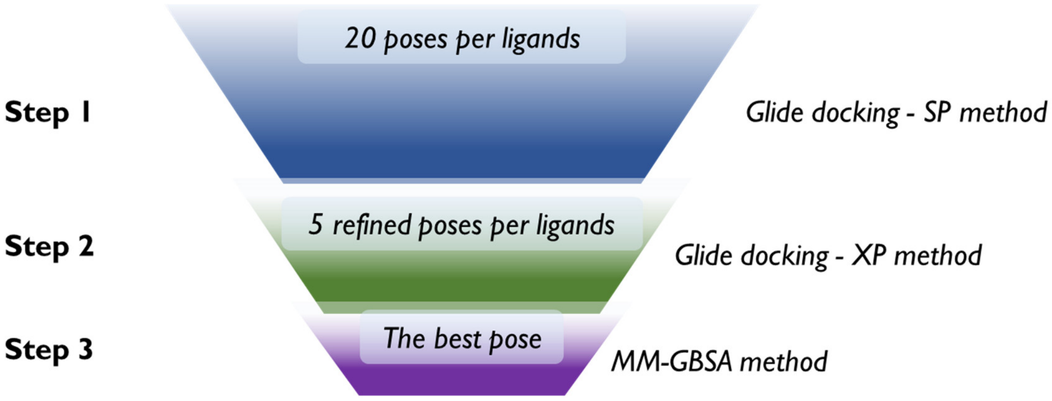

Figure S4. Docking protocol for synthetized ligands.

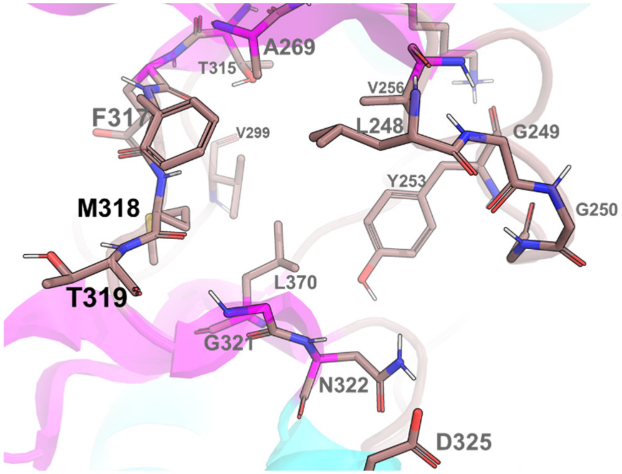

Figure S5. Graphic representation of the active site of Bcr-Abl protein.

Table S4. Main energetic contributions per residues between the active site of Bcr-Abl with the synthesized ligands.

Comp.	XP-score (kcal/mol)	H.-bond (kcal/mol)	van der Waals energetic contributions (kcal/mol)											
			L248	G249	A250	Y253	V256	A269	F317	T319	L370	G321	N322	D325
**4i**	-8.51	-1.61	-5.32	-2.14	-0.37	-4.30	-2.91	-0.97	0.64	-2.18	-2.30	-2.52	-2.34	-0.41
**5b**	-5.96	-0.58	-5.32	-1.72	-0.32	-2.50	-1.79	-1.24	-2.34	-1.99	-1.66	-3.11	-1.54	-0.18
**5j**	-5.63	-1.00	-4.97	-1.81	-0.28	-2.72	-2.01	-1.43	-2.95	-2.72	-2.26	-2.49	-1.32	-0.13

Table S5. Prime MM-GBSA calculation of the docked complexes (ligands-Bcr-Abl).

Comp.	ΔG Bind	ΔG Coul.	ΔG H.-bond	ΔG Lipo	ΔG Solv GB	ΔG vdW
**4i**	-74.52	-45.32	0.37	-24.02	50.1	-54.57
**5b**	-57.32	-37.03	1.00	-27.62	52.86	-44.83
**5j**	-58.47	-37.74	0.71	-32.41	59.61	-50.57

ΔG Bind: MM-GBSA free energy of binding.
ΔG Coul: Coulomb energy of the complex.
ΔG H.-bond: Hydrogen-bonding energy correction of the complex.
ΔG Solv GB: Generalized Born electrostatic solvation energy of the complex.
ΔG Lipo: Lipophilic energy of the complex.
ΔG vdW: van der Waals energy of the complex.

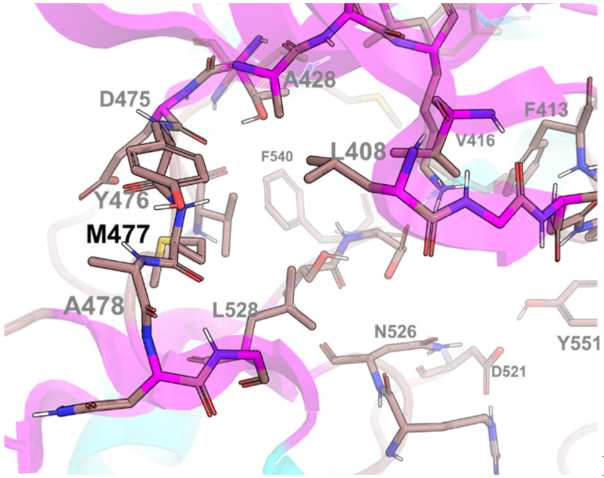

Figure S6. Graphic representation of the active site of BTK protein.

Table S6. Main energetic contributions per residues between the active site of BTK with the synthesized ligands.

**Comp.**	**XP-score (kcal/mol)**	**H.-bond (kcal/mol)**	**van der Waals energetic contributions (kcal/mol)**											
		**M477**	**L408**	**F413**	**V416**	**A428**	**Y476**	**A478**	**D521**	**N526**	**L528**	**F540**	**L542**	**Y551**
**4i**	-6.82	-0.60	-6.22	-1.19	-4.18	-2.65	-3.57	-1.80	-0.71	-2.41	-4.20	-0.12	-0.27	-0.86
**5b**	-6.41	-0.58	-5.90	-1.33	-3.43	-1.63	-3.32	-1.77	-0.81	-2.70	-4.85	-0.44	-0.60	-1.08
**5j**	-6.01	-0.76	-5.85	-1.27	-3.50	-1.63	-3.36	-1.70	-0.80	-2.66	-4.78	-2.26	-1.72	-1.08

Table S7. Prime MM-GBSA calculation of the docked complexes (ligands-BTK).

**Comp.**	**ΔG Bind**	**ΔG Coul.**	**ΔG H.-bond**	**ΔG Lipo**	**ΔG Solv GB**	**ΔG vdW**
**4i**	-73.37	-31.75	-0.63	-20.4	41.58	-66.42
**5b**	-67.34	-29.39	-0.47	-21.65	43.68	-66.36
**5j**	-86.08	-33.12	-1.00	-27.66	45.3	-74.19

ΔG Bind: MM-GBSA free energy of binding.
ΔG Coul: Coulomb energy of the complex.
ΔG H.-bond: Hydrogen-bonding energy correction of the complex.
ΔG Solv GB: Generalized Born electrostatic solvation energy of the complex.
ΔG Lipo: Lipophilic energy of the complex.
ΔG vdW: van der Waals energy of the complex.

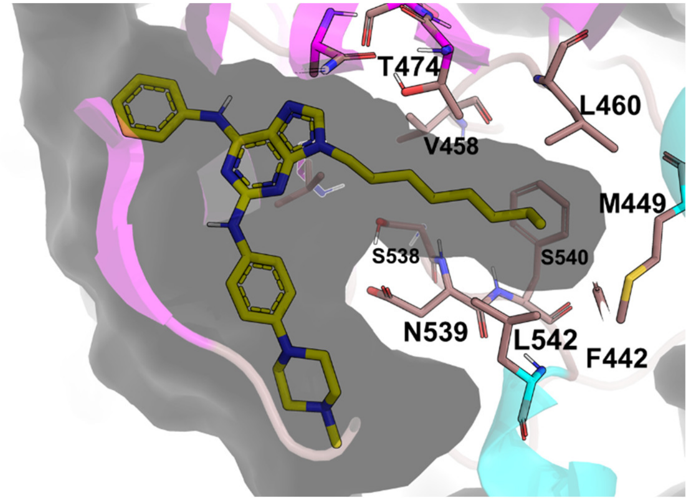

Figure S7. Graphic representation of the hydrophobic pocket of BTK protein and **5j** structure in yellow.

Table S8. Main energetic contributions per residues the hydrophobic pocket of BTK with synthetized ligands.

	van der Waals energetic contributions (kcal/mol)								
**Comp.**	**T474**	**V458**	**L460**	**M449**	**F442**	**N539**	**S538**	**F540**	**L542**
**4i**	-2.32	-1.25	-0.13	-0.02	-0.02	-2.87	-0.98	-0.12	-0.27
**5b**	-2.56	-2.27	-0.30	-0.87	-0.12	-4.24	-1.91	-0.44	-0.60
**5j**	-2.62	-2.41	-1.70	-0.87	-0.12	-4.82	-2.30	-2.26	-1.72

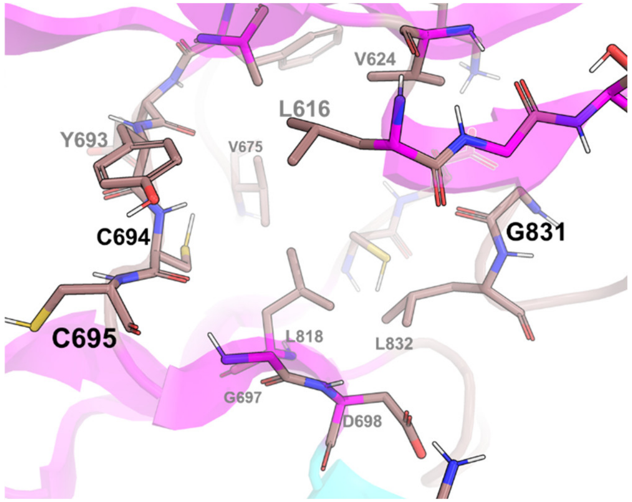

Figure S8. Graphic representation of the active site of FLT3 protein.

Table S9. Main energetic contributions per residues between the active site of FLT3 with the synthesized ligands.

	XP-Score	H.-bond (kcal/mol)			van der Waals energetic contributions (kcal/mol)										
**Comp.**	**(kcal/mol)**	**Y693**	**C694**	**G831**	**L616**	**V624**	**V675**	**Y693**	**C694**	**C695**	**G697**	**D698**	**L818**	**G831**	**L832**
4i	-10.36	0.00	-0.39	-0.87	-7.70	-2.61	-1.06	-3.80	-3.61	-1.04	-1.67	-2.86	-3.57	-2.06	-5.17
5b	-11.38	0.00	-2.00	0.00	-8.08	-3.40	-1.88	-4.39	-3.19	-1.78	-1.99	-3.34	-3.29	-2.12	0.03
5j	-3.21	-0.55	-0.23	0.00	-7.72	-1.83	-1.35	-4.15	-3.46	-2.50	-3.43	-2.16	-1.46	-1.60	-3.97

Table S10. Prime MM-GBSA calculation of the docked complexes (ligand-FLT3).

Comp.	ΔG Bind	ΔG Coul.	ΔG H.-bond	ΔG Lipo	ΔG Solv GB	ΔG vdW
**4i**	-74.22	-40.97	-0.34	-20.29	47.45	-48.12
**5b**	-74.05	-42.12	-0.36	-24.81	55.74	-47.82
**5j**	-61.34	-30.91	-0.06	-23.51	59.65	-60.91

ΔG Bind: MM-GBSA free energy of binding.
ΔG Coul: Coulomb energy of the complex.
ΔG H.-bond: Hydrogen-bonding energy correction of the complex.
ΔG Solv GB: Generalized Born electrostatic solvation energy of the complex.
ΔG Lipo: Lipophilic energy of the complex.
ΔG vdW: van der Waals energy of the complex.

References

(1) Schrödinger Release 2021-1: Protein Preparation Wizard; Schrödinger, LLC, New York, NY, 2021.

(2) Friesner, R. A.; Murphy, R. B.; Repasky, M. P.; Frye, L. L.; Greenwood, J. R.; Halgren, T. A.; Sanschagrin, P. C.; Mainz, D. T. Extra Precision Glide: Docking and Scoring Incorporating a Model of Hydrophobic Enclosure for Protein-Ligand Complexes. *J. Med. Chem.* **2006**, *49* (21), 6177–6196. https://doi.org/10.1021/jm051256o.
